# Bronchial airway gene expression signatures in mouse lung squamous cell carcinoma and their modulation by cancer chemopreventive agents

**DOI:** 10.18632/oncotarget.13806

**Published:** 2016-12-07

**Authors:** Donghai Xiong, Jing Pan, Qi Zhang, Eva Szabo, Mark Steven Miller, Ronald A. Lubet, Ming You, Yian Wang

**Affiliations:** ^1^ Cancer Center, Medical College of Wisconsin, WI 53226, USA; ^2^ Department of Pharmacology and Toxicology, Medical College of Wisconsin, Milwaukee, WI 53226, USA; ^3^ Chemopreventive Agent Development Research Group, Division of Cancer Prevention, National Cancer Institute, Rockville, MD 20850, USA

**Keywords:** AUC (area under the curve), GSEA (gene set enrichment analysis), IPA (Ingenuity Pathway Analysis), PPAR (peroxisome proliferator-activated receptor gamma), SCC (squamous cell carcinoma)

## Abstract

Due to exposure to environmental toxicants, a “field cancerization” effect occurs in the lung resulting in the development of a field of initiated but morphologically normal appearing cells in the damaged epithelium of bronchial airways with dysregulated gene expression patterns. Using a mouse model of lung squamous cell carcinoma (SCC), we performed transcriptome sequencing (RNA-Seq) to profile bronchial airway gene expression and found activation of the PI3K and Myc signaling networks in cytologically normal bronchial airway epithelial cells of mice with preneopastic lung SCC lesions, which was reversed by treatment with the PI3K Inhibitor XL-147 and pioglitazone, respectively. Activated MYC signaling was also present in premalignant and tumor tissues from human lung SCC patients. In addition, we identified a key microRNA, mmu-miR-449c-5p, whose suppression significantly up-regulated Myc expression in the normal bronchial airway epithelial cells of mice with early stage SCC lesions. We developed a novel bronchial genomic classifier in mice and validated it in humans. In the classifier, Ppbp (pro-platelet basic protein) was overexpressed 115 fold in the bronchial airways of mice with preneoplastic lung SCC lesions. This is the first report that demonstrates Ppbp as a novel biomarker in the bronchial airway for lung cancer diagnosis.

## INTRODUCTION

Lung cancer is the leading cause of cancer related death around the world [1]. Lung squamous cell carcinoma (SCC) is a major category of non-small cell lung cancers (NSCLC), accounting for about 25–30% of total lung cancer cases in the US population. Lung SCC is a highly heterogeneous disease that develops via multiple complex steps [2, 3]. Genetic etiology of lung SCC has been studied extensively in human and animal models [4–6]. However, the understanding of lung SCC genetic etiology is still incomplete and there is high heterogeneity in the cause of this cancer according to large sequencing projects utilizing tissue from human patients [7].

Although there has been extensive work examining the development of lung adenocarcinomas in both chemically induced and transgenic models, there is limited examination of squamous cell carcinoma of the lung in rodent models. In 2004 we published on a *N*-nitroso-tris-chloroethylurea (NTCU) induced squamous cell model [8] based on the initial studies of Rehm and Lijinsky [9]. Multiple groups have confirmed the ability of the model to generate lesions in the squamous region of the lung following treatment of various mouse strains with NTCU. However, little is known about whether the gene-expression biomarkers or classifiers from bronchial airway samples of mice in which NTCU is used to induce SCC recapitulate the molecular lesions found in human patients. As a consequence of prolonged exposure to environmental toxicants such as cigarette smoke or air pollution, a “field cancerization” effect occurs in the lung resulting in the development of a field of initiated but morphologically normal appearing cells in the damaged bronchial epithelium that contain molecular lesions such as mutations in an oncogene or tumor suppressor gene [10–14]. Tissue samples from this extended injured area can be collected in a less invasive manner by bronchial brushing and have been used to develop a gene expression–based biomarker signature for distinguishing lung cancer in smokers [15]. Gene-expression classifier from bronchial airway was also developed to improve the diagnosis of lung cancer [16]. We therefore wished to compare RNA expression results in the NTCU mouse model and compare it with human data.

In this study, we therefore conducted RNA-sequencing analysis using bronchial airway samples from mice that were treated with NTCU. Normal appearing bronchial epithelial cells from treated mice that represent the field of damaged cells and contain undetectable preneoplastic lesions of SCC (referred to as preneoplastic or lung SCC early lesions) were isolated. We identified significant activation of oncogenic pathways, especially the PI3K/AKT and Myc pathways as early events during lung SCC development. Modulation of these important pathways would appear to be relevant to prevention or early therapy of SCC. In particular, we found that the PI3K pathway could be modulated by the PI3K inhibitor XL-147 while the Myc pathway was modulated by the peroxisome proliferator-activated receptor gamma (PPAR) agonist - pioglitazone in the bronchial airways of mice containing preneoplastic SCC lesions. XL-147 and pioglitazone have been used as chemopreventive agents against human cancer. Specifically, PI3K pathway inhibition by XL-147 inhibits proliferation, angiogenesis, cellular invasion, and tumor cell growth and survival [17]. Pioglitazone has also been found to be able to prevent carcinogenesis in preclinical model and patients [18, 19]. Our data revealed the molecular mechanisms of how these two chemopreventive agents play their anti-tumor roles in the context of lung cancer. Finally, we performed microRNA-seq (miRNA-seq) and integrative analysis of RNA-seq and miRNA-seq data, which identified a key microRNA, mmu-miR-449c-5p, as a novel bronchial airway biomarker for lung SCC that exhibits tumor suppressor activity through inhibition of *Myc* expression. Finally, we developed a 36-gene-expression classifier from bronchial epithelial cells of NTCU treated mice, which can be extended to the human bronchial airway samples and may aid in the diagnosis of squamous cell lung cancer.

## RESULTS

### PI3K/AKT pathway and Myc signaling network were activated in cytologically normal bronchial airway epithelial cells of preneoplastic lung SCC lesions

**Figure 1:**
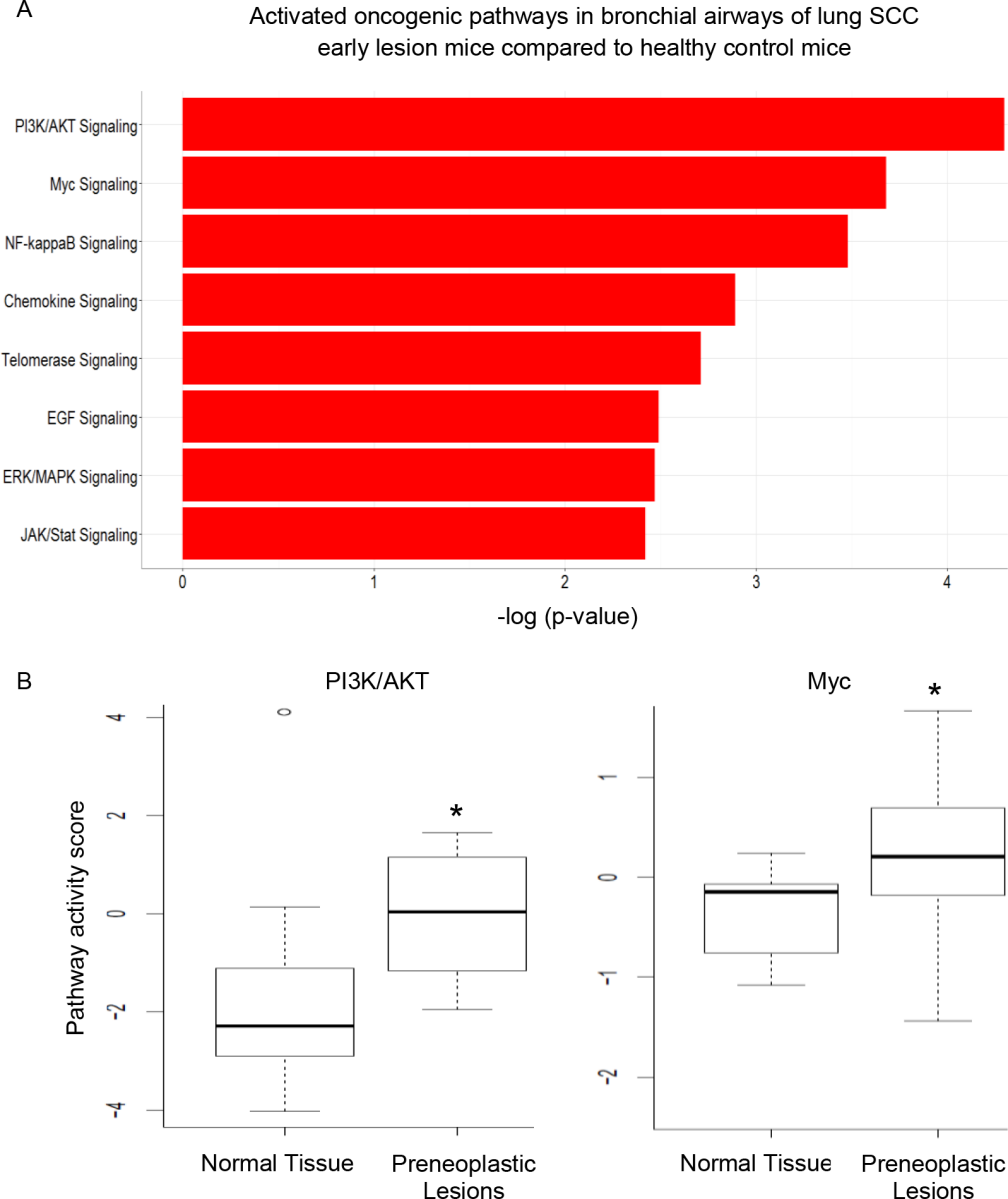
The significantly activated oncogenic pathways in the bronchial airways of mice harboring preneoplastic SCC lesions (**A**) PI3K pathway and Myc signaling network were the top two most significant pathways activated as identified by IPA; (**B**) DART validated the activation status of PI3K/AKT and Myc signaling (*P < 0.001).

We analyzed patterns of pathway deregulation in cytologically normal bronchial airway epithelial cells from NCTU-treated mice obtained from bronchial brushings after 20 weeks of NTCU treatment (lung SCC early-lesion model). First, we filtered out genes with low expression (median expression values < 4 cpm [counts per million mapped reads]). Then we used the statistical R packages *edgeR* and *limma* [20, 21] to generate the list of differentially expressed genes in bronchial airway samples between carcinogen treated and control untreated mice. This was to cross-validate the results generated by different RNA-seq data analysis programs and obtain the most reliable set of differential expression genes for follow-up analyses. A total of 3,016 genes were differentially expressed between, with 1,252 genes up-regulated and 1,764 genes down-regulated in the airway samples of mice harboring preneoplastic SCC lesions. The follow-up IPA (Ingenuity Pathway Analysis) study of the differentially expressed genes suggested that a number of oncogenic pathways/networks, *i.e*., PI3K/AKT, Myc, NF-ĸB, Chemokine, Telomerase, EGF, ERK/MAPK, and JAK/Stat signaling pathways were activated in the bronchial airways of mice harboring preneoplastic SCC lesions. The top two activated pathways identified by IPA were the PI3K pathway and Myc signaling network (Figure 1A). We further used another program, DART [22], to check the activation status of oncogenic pathways in our samples. Only the activation of PI3K/AKT and Myc signaling was further confirmed (Figure 1B).

**Figure 2:**
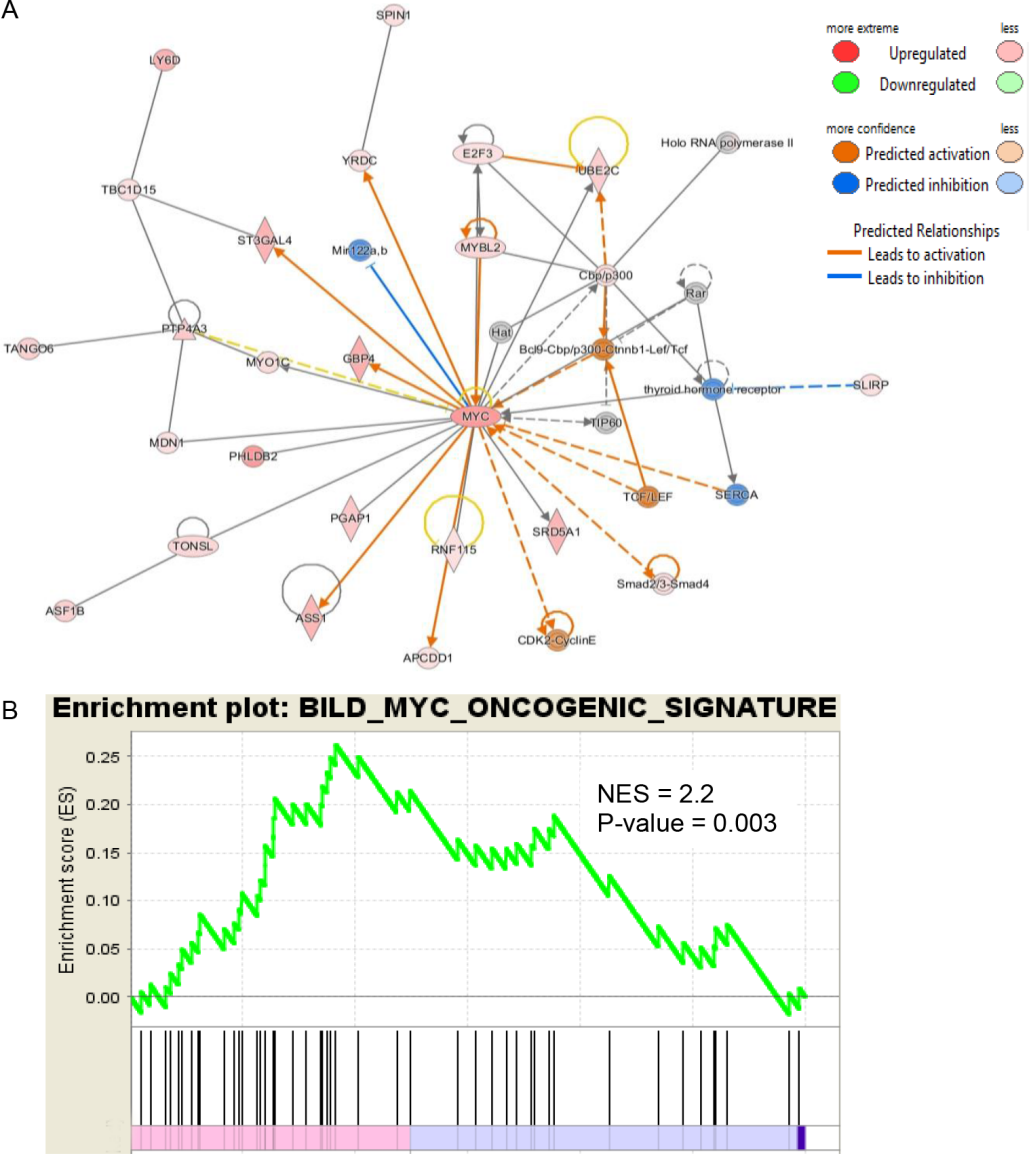
IPA and GSEA identified Myc as a dysregulated transcription factor in the bronchial airway epithelial cells from NCTU-treated mice (**A**) Expression patterns of known downstream targets of Myc identified by IPA suggest the activation of Myc even in the bronchial airway cells of mice with preneoplastic SCC lesions. (**B**) GSEA plot for the Myc targets gene set significantly enriched in the bronchial airway cells of mice with preneoplastic SCC lesions.

**Figure 3:**
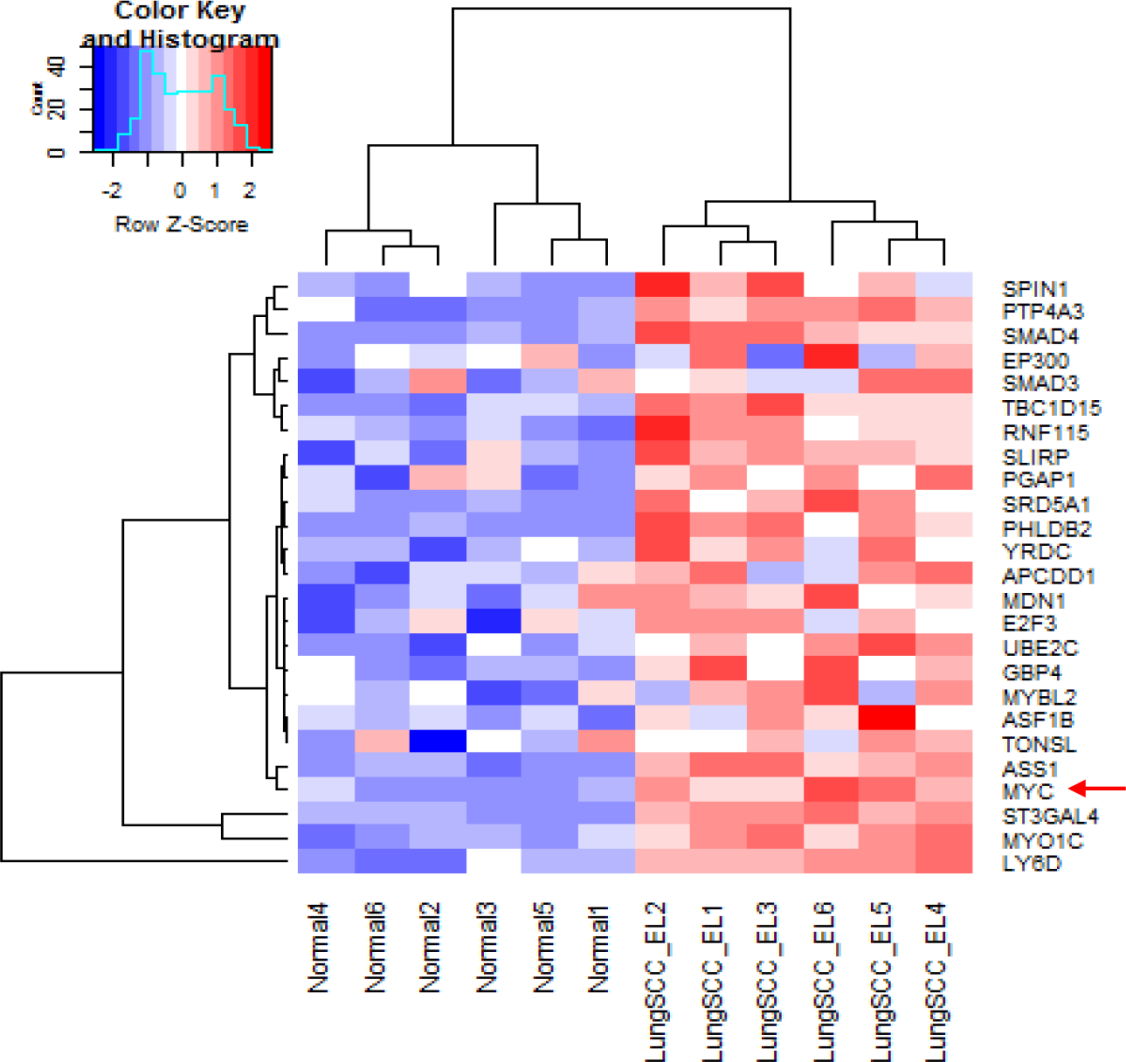
Significantly increased expression of Myc and its downstream targeted genes in the bronchial airway epithelial cells from mice with preneoplastic SCC lesions compared to the normal mice without cancer LungSCC_EL: preneoplastic lung SCC lesions; Normal: normal tissue from control mice without cancer.

IPA was used to determine whether the differentially expressed genes were enriched in known targets of various transcription factors. This approach showed that Myc was a dysregulated transcription factor in the normal appearing airway epithelial cells from NTCU treated mice. The differentially expressed genes in the airways were also enriched in the previously reported targets of Myc (Figure 2A). As Myc is predicted to activate the expression of these target genes, the IPA program showed that Myc activity was significantly induced (*P* = 1.13 E-21, Z-score = 2.152). We also performed pre-ranked gene set enrichment analysis (GSEA) using the well-established oncogenic pathway signatures [23] and further validated that bronchial airway cells from NTCU-treated mice were enriched for genes from the Myc signaling network (Figure 2B). Importantly, we found that the expression levels of both *Myc* and its downstream target oncogenes such as LY6D, ST3GAL4, ASS1, PTP4A3, UBE2C and E2F3 increased in the bronchial airways significantly during progression from normal to preneoplastic SCC lesions (Figure 3, Supplementary Table S1). This suggests that the elevated activity of Myc network genes are due to the increased gene expression in the bronchial airways of the carcinogen-treated mice.

### PI3K/AKT and Myc signaling activation in bronchial airways of lung SCC early-lesion mice can be reversed by XL-147 and pioglitazone treatment

**Figure 4:**
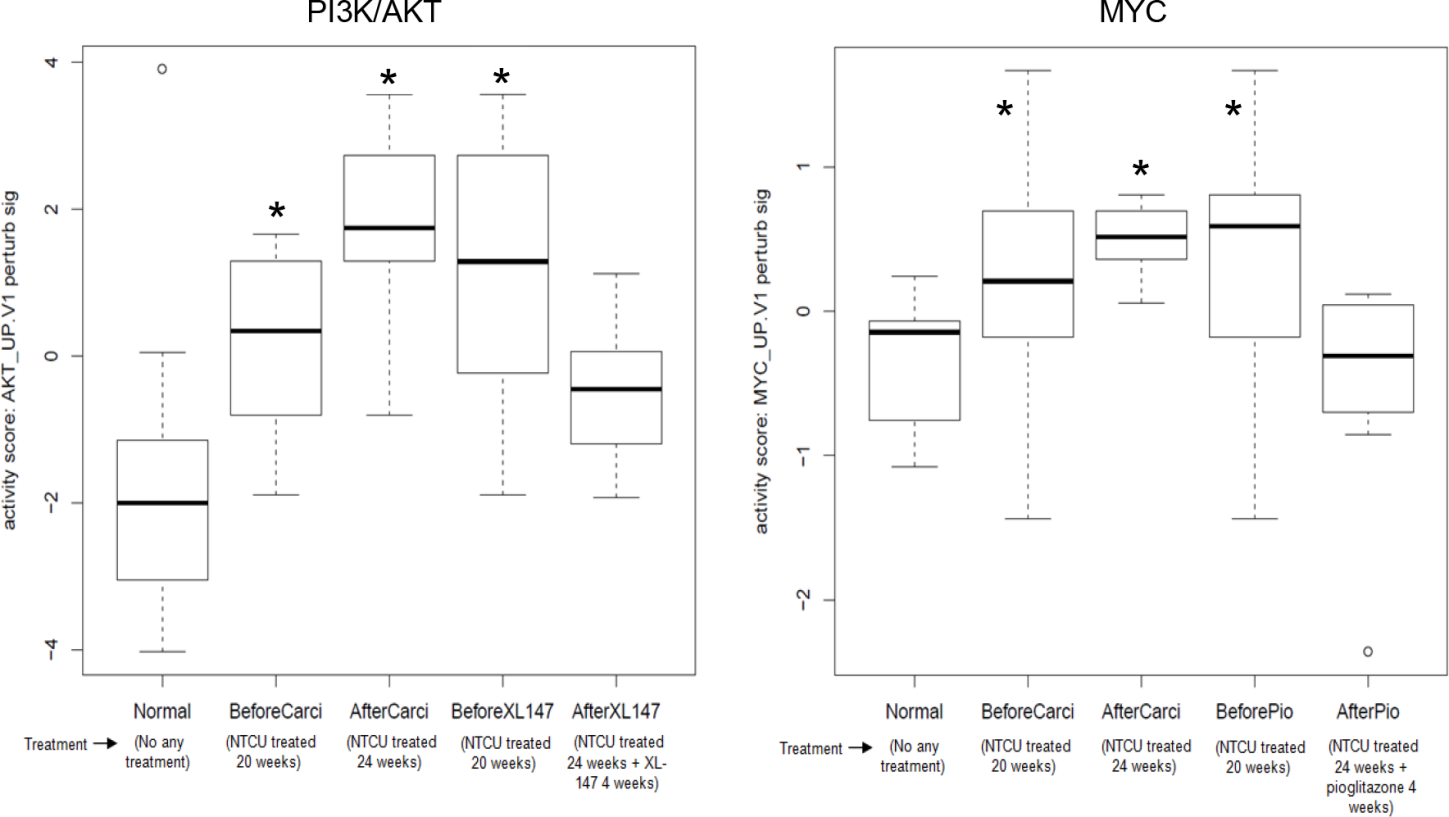
Elevated and reversible PI3K/AKT and Myc pathway activity in the airway samples of the mouse lung SCC early lesion model (**A**) PI3K/AKT pathway activation in the airway samples of the mouse lung SCC early-lesion model treated with NTCU compared to the normal control mice. The elevated PI3K/AKT activity can be inhibited by XL-147 in the neoplastic cells of the mouse lung SCC early-lesion models. *P < 0.01 when comparing each of the 3 designated groups to either “Normal” or “AfterXL147” group; (**B**) Myc pathway activation in the airway samples of the mouse lung SCC early lesion model. The elevated Myc activity can be inhibited by pioglitazone to the normal level in the preneoplastic cells.. *P < 0.01 when comparing each of the 3 designated groups to either “Normal” or “AfterPio” group.

We analyzed the RNA-seq data on the paired bronchial airway samples from 1) lung SCC early lesion mice before and after the extended NTCU treatment for 4 more weeks (i.e., 20 weeks mice vs 24 weeks mice, named “BeforeCarci” and “AfterCarci”); 2) lung SCC early lesion mice before and after the extended 4-week NTCU plus 100 mg/kg XL-147 treatment (i.e., 20 weeks mice vs 24 weeks mice, named “BeforeXL147” and “AfterXL147”); 3) lung SCC early lesion mice before and after the extended 4-week NTCU plus 15 mg/kg pioglitazone treatment (i.e., 20 weeks mice vs 24 weeks mice, named “BeforePio” and “AfterPio”). We also analyzed the RNA-seq data on the bronchial airway samples from the parallel normal control group of mice without any treatment. As expected, the PI3K/AKT and Myc pathway were activated in the airway samples of all the 3 groups of lung SCC early-lesion mice treated with NTCU compared to the normal control mice not exposed to any carcinogen. The elevated PI3K/AKT and Myc pathway activities observed in the mice treated with NTCU for 24 weeks (AfterCarci) can be down-regulated by XL-147 (a PI3K inhibitor) and pioglitazone, respectively, to the similar level as observed in the normal control group (Figure 4). These data suggest that PI3K/AKT and Myc pathway activation are early and reversible events in the normal appearing epithelial cells of the upper bronchial airways during lung SCC development. In total, XL-147 treatment led to 1,135 differential expressed genes consisting of 603 significantly down-regulated genes and 532 significantly up-regulated genes after XL-147 treatment (Supplementary Table S2). Pioglitazone treatment resulted in 3,018 differential expressed genes consisting of 1,491 significantly down-regulated genes and 1,527 significantly up-regulated genes after pioglitazone treatment (Supplementary Table S3).

### Myc signaling activation was also present in the early lesions/tumors of the human and mouse lung SCC

**Figure 5:**
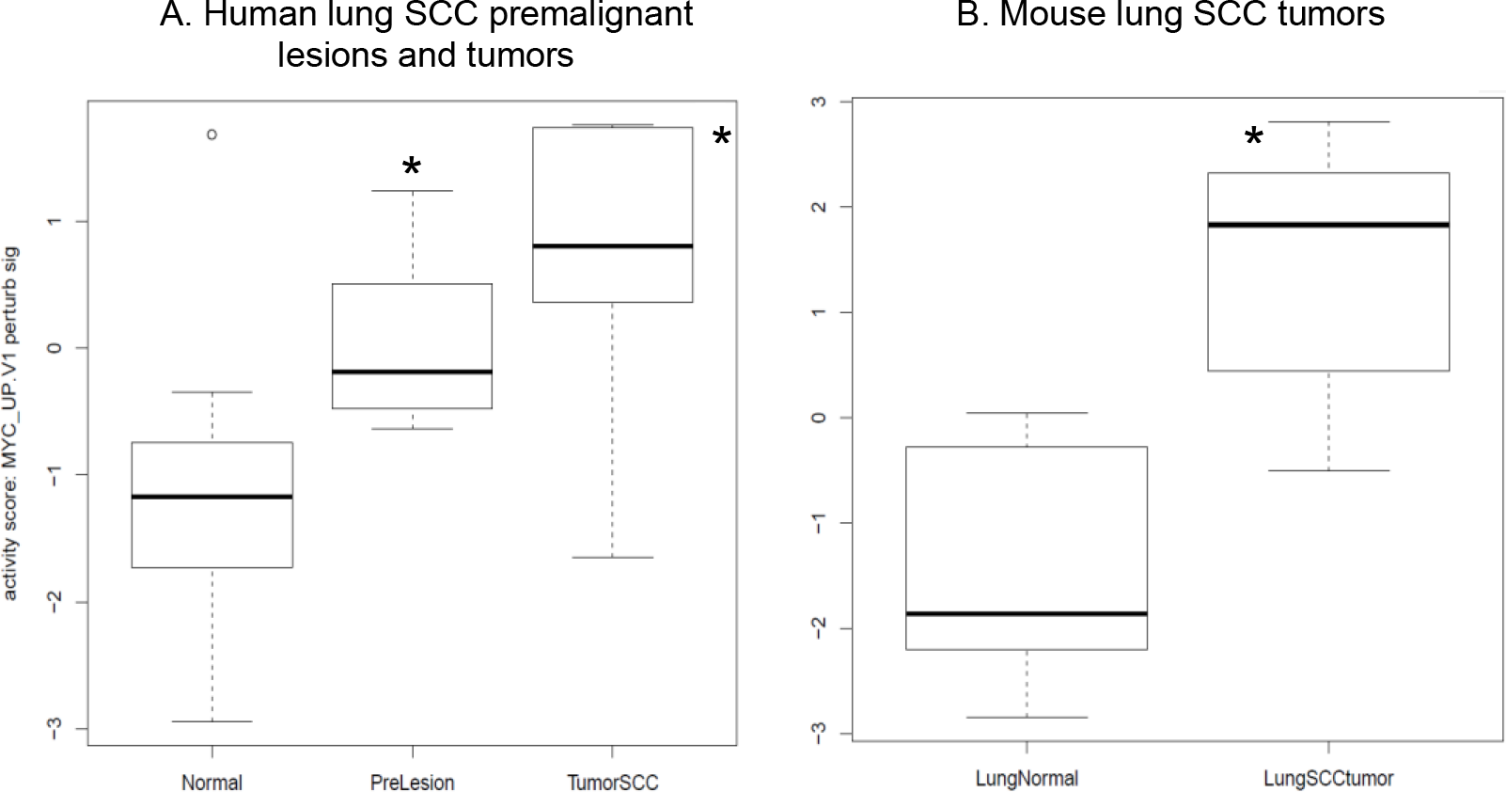
MYC oncogenic pathway is significantly activated in (A) the human lung SCC premalignant lesions and tumors relative to the bronchial airway samples based on the RNA-seq data from [24] and (B) the mouse lung SCC tumors based on our own RNA-seq data *P < 0.01 when comparing each of the designated group to the normal control group.

**Figure 6:**
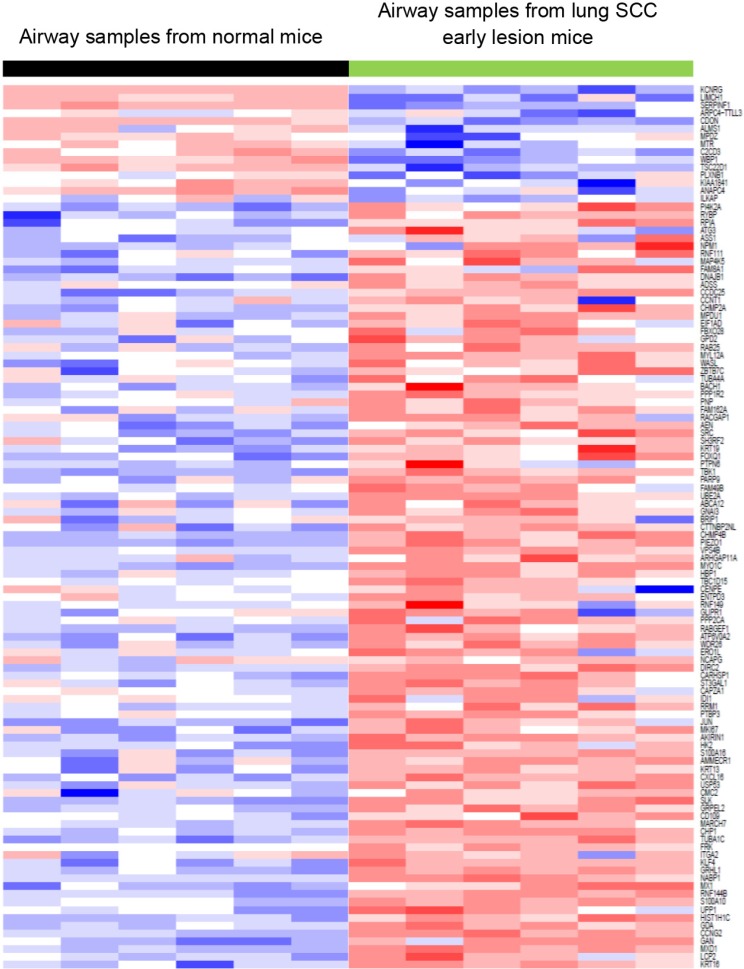
The signature genes for lung SCC premalignant lesion samples were similarly regulated in the bronchial airway samples of lung SCC early-lesion mice This suggested that the gene expressions of bronchial airway samples can be used to recapitulate the molecular changes that occur during the early carcinogenesis of lung SCC, which may improve early diagnosis of lung SCC.

In addition, we downloaded human RNA-seq data on laser-microdissected cell populations along the SCC pathologic continuum of normal basal cells, premalignant lesions, and tumor cells [24]. The oncogenic pathway analysis showed that the Myc pathway was significantly activated in both human lung SCC premalignant lesions and tumors (Figure 5A). We previously did RNA-seq for a sample set consisting of 16 mouse lung SCC tumors and 8 normal lung tissues. By analyzing this dataset, we found that the Myc pathway was also significantly activated in the mouse lung SCC tumors (Figure 5B). These were consistent with the findings from the RNA-seq analysis of the bronchial airway samples of lung SCC early-lesion mice, suggesting that Myc network activation is a common phenomenon present in both the bronchial airways and early lesions/tumors of the human and mouse lung SCC. We also found that the signature genes for human lung SCC premalignant lesion samples [24] were similarly regulated in the normal appearing bronchial airway samples of NTCU treated mice (Figure 6). This suggests that the gene expression alterations observed in murine preneoplastic bronchial airway samples resemble the molecular changes that occur during the early stages of human lung SCC development, which supports the field effect theory that the damage caused by carcinogens is not limited solely to the lung but rather form a “field of injury” throughout the entire respiratory tract [10]. PI3K pathway activity was also elevated in the human lung tumors as has been reported previously [10], hence we did not elaborate on this pathway here.

### Integrative analysis of the RNA-seq and miRNA-seq data for the airway brush samples identified mmu-miR-449c-5p as a novel biomarker and a tumor suppressor

The integrative analysis of the RNA-seq and miRNA-seq data using the R software package MiRComb [25] showed that 2,335 miRNA-mRNA pairs were significantly negative correlated in expression (Supplementary Table S4) and the paired members were predicted by at least one miRNA-mRNA database (microCosm/TargetScan or both) to target each other. Each member of these miRNA-mRNA pairs was deregulated in the airway brush samples of the lung SCC early-lesion mice by at least 3 fold compared to the normal control group.

Following this analysis, we then filtered the list of significant miRNA-mRNA pairs using the following criteria i) the genes in the miRNA-mRNA pairs should be in the compiled list of 1571 cancer genes provided by The Network of Cancer Genes (NCG, http://ncg.kcl.ac.uk/), which is a manually curated repository of cancer genes derived from the scientific literature [26] ii) the marked ‘oncogenes’ by NCG that showed up in our miRNA-mRNA pairs list were up-regulated in the lung SCC early-lesion mice relative to the normal control mice; iii) the marked ‘tumor suppressor genes’ by NCG that showed up in our miRNA-mRNA pairs list were down-regulated in the lung SCC early-lesion mice relative to the normal control mice. The filtering steps gave a short list of six oncogenes - *Myc*, *Lmo1*, *Ccnd2*, *Specc1*, *Notch1*, and *Pim1* as well as two tumor suppressor genes, *Msh2* and *Chek2* along with their partner miRNAs. Table 1 summarizes the statistics of these most significant miRNA-mRNA pairs. The negative correlations of these genes with their paired miRNAs were plotted in Figure S1. It can be seen that the significant decrease of mmu-miR-449c-5p level (~ 14 fold) significantly correlated with the large increase of Myc expression (~ 8 fold) in the normal appearing bronchial airway cells from NTCU treated mice relative to normal tissue from control mice. Given the importance of Myc signaling activation in the early development of SCC in the bronchial airways of NTCU treated mice, it appears that mmu-miR-449c-5p could be a novel bronchial airway biomarker and a tumor suppressor via its inhibition of Myc expression during early tumorigenesis of lung SCC.

**Table 1:** Statistics of the negative correlations of the six oncogenes – Myc, Lmo1, Ccnd2, Specc1, Notch1, Pim1 and two tumor suppressor genes – Msh2 and Chek2 with their most significant correlated miRNAs

mmuMiR	mmuGene	cor	pval	adj.pval	miRNA fold change	mRNA fold change	description
mmu-miR-449c-5p	Myc	−0.89	1.2E-04	1.60E-03	−13.7	7.7	v-myc avian myelocytomatosis viral oncogene homolog
mmu-miR-181a-5p	Lmo1	−0.73	5.8E-03	1.80E-02	−8.1	5.1	LIM domain only 1 (rhombotin 1)
mmu-let-7d-3p	Ccnd2	−0.94	6.0E-06	4.00E-04	−77.7	4.3	cyclin D2
mmu-miR-375-3p	Specc1	−0.83	7.4E-04	4.40E-03	−7.7	3.8	sperm antigen with calponin homology and coiled-coil domains 1
mmu-miR-92b-5p	Notch1	−0.95	3.2E-06	3.10E-04	−108.1	3.5	notch 1
mmu-miR-328-3p	Pim1	−0.9	7.6E-05	1.20E-03	−48.1	3.1	Pim-1 proto-oncogene serine/threonine kinase
mmu-miR-223-3p	Msh2	−0.88	2.1E-04	2.10E-03	567.7	−3.6	mutS homolog 2
mmu-miR-143-3p	Chek2	−0.81	1.3E-03	6.40E-03	6.9	−3.5	checkpoint kinase 2

### A bronchial genomic classifier derived from lung SCC early lesion mice can be used for the diagnostic evaluation of lung cancer in humans

**Figure 7:**
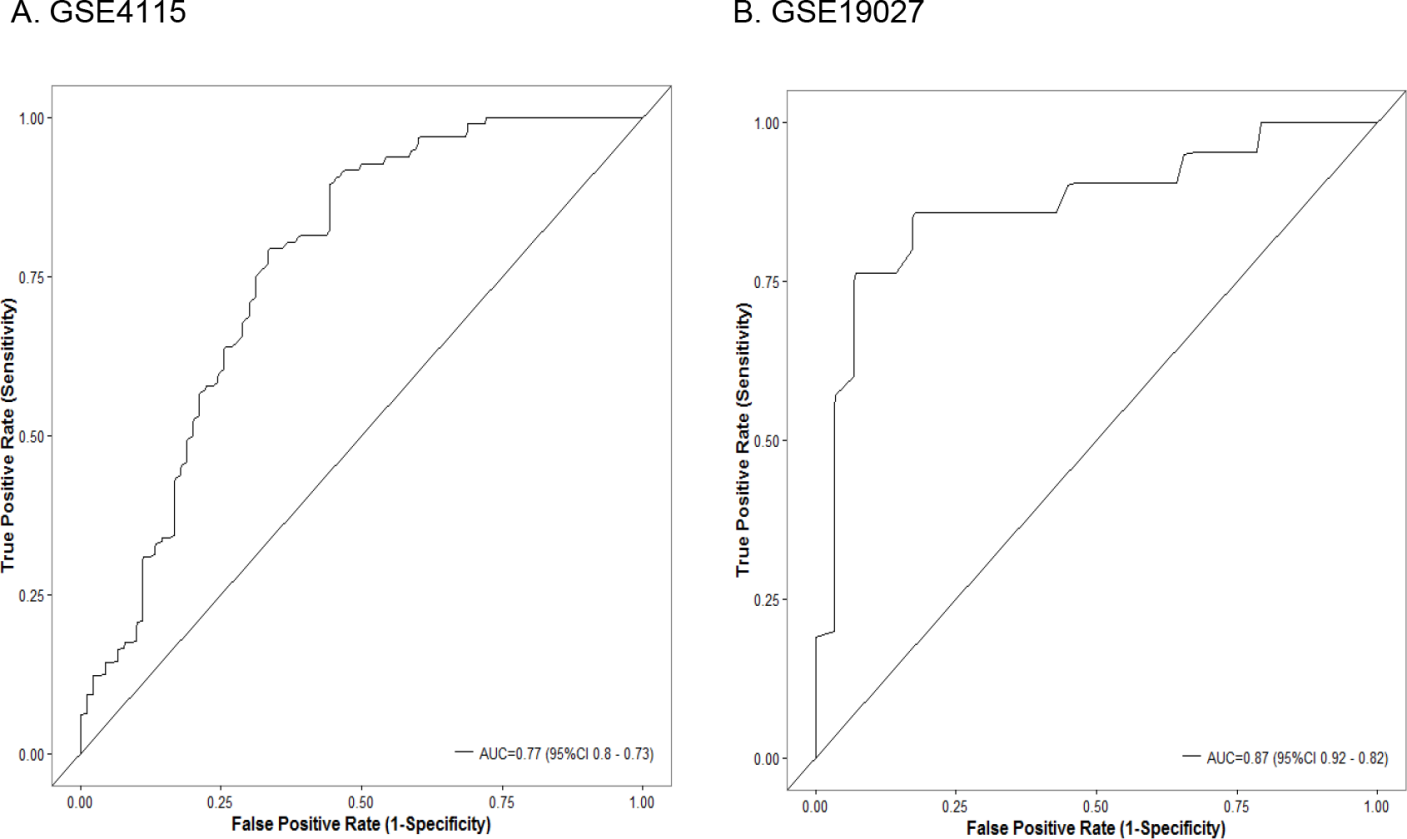
Classifier performance in the (A) Dataset GSE4115 comprising 90 smokers without cancer and 97 smokers with lung cancer; (B) Dataset GSE19027 comprising 29 smokers without cancer and 21 smokers with lung cancer Shown are receiver-operating-characteristic curves. In GSE4115, the area under the curve (AUC) was 0.77 (95% confidence interval [CI], 0.73 to 0.8). In GSE19027, the AUC was 0.87 (95% CI, 0.82 to 0.92).

Due to the similarity of the gene expression patterns, we further investigated whether the bronchial genomic classifier from mice can assist in the diagnosis of lung cancer in humans. We developed a 36-gene classifier from the bronchial airway samples of lung SCC early-lesion mice (Supplementary Figure S2). We tested the effectiveness of this 36-gene classifier from mice in the two human microarray datasets. In GSE4115, the classifier had an area under the receiver-operating-characteristic curve (AUC) of 0.77 (95% confidence interval [CI], 0.73 to 0.8), a sensitivity of 80% (95% CI, 73% to 87%), and a specificity of 63% (95% CI, 54% to 72%). In GSE19027, the classifier had an AUC of 0.87 (95% CI, 0.82 to 0.92), a sensitivity of 86% (95% CI, 67%-96%), and a specificity of 72% (95% CI, 56%–85%). The performance of the classifier in the two human datasets can be seen from Figure 7, which is as good as the previous reported signature in terms of sensitivity (86% vs. 89%) [16] and demonstrated a significant higher specificity (72% vs. 47%). These data suggested that the gene-expression classifier developed from the bronchial airway samples from the mouse lung SCC models may be applied to the accurate diagnosis of squamous lesions in humans and might be used for determining individuals (smokers or ex-smokers) who are at particularly high risk and may be candidates for preventive protocols.

## DISCUSSION

Although gene-expression signatures have been developed from bronchial brush samples to infer early genomic events important to lung cancer etiology in human populations, no corresponding research has been done in mouse models. The goal of this study was to determine the molecular changes in bronchial airways that may reflect the early events associated with lung SCC carcinogenesis in the NTCU mouse model. Furthermore, we compared the gene expression signature of the mouse model with data previously generated in humans. The objectives were to help validate the SCC model as well as to determine gene pathways and gene targets which might prove applicable for prevention/therapy of SCC employing this model. Our data showed that the NTCU mouse model has significant overlap in terms of alterations in oncogenic pathways with human lung SCC, therefore making it a legitimate model for screening novel targeted therapeutic and chemopreventive agents. We demonstrated that the PI3K/AKT and Myc signaling pathways were activated in the normal appearing bronchial airway epithelial cells of NTCU mice. The activation of PI3K signaling was consistent with the findings in human bronchial brush samples, which determined that PI3K is activated in the proximal airway prior to development of clear lung tumors [10]. The activation of Myc network was reported in early stage human lung SCC lesions [24] and tumors [10]. However, our study discovered, for the first time, that Myc signaling is also up-regulated in the bronchial airways of preneoplastic lung SCC lesions in mice, suggesting that Myc network is deregulated early in the progression of lung SCC lesions and and is overexpressed in SCCs as well.

We found that Myc network activation in the bronchial airways of mice with preneoplastic lung SCC lesions is mediated through the upregulation of Myc gene expression. This is different from what has been found in the premalignant lesions and malignant tumor samples in human lung SCC, where it was reported that the Myc network was activated by nuclear translocation of the Myc transcriptional factor rather than Myc expression change [24]. Our data suggested that the initiation stage of lung SCC already demonstrates activation of the Myc network (Figures 1, 2, 3) and this appears to be mediated by upregulating the expression levels of the Myc gene in bronchial airways relative to the original ‘healthy’ state of the epithelial cells in untreated control mice (Figure 3). The elevated expression of Myc remains constant across the bronchial airways, early lesions, and tumors of human lung SCC patients [24]. However, Myc signaling activity can be further elevated in the early lesion/tumor tissues relative to the bronchial airway samples by using non-transcriptional regulation mechanisms such as Myc nuclear translocation [24]. These data indicated that the activation of Myc signaling can be realized through different mechanisms during the stepwise progression of lung SCC, which has not been reported previously. Corresponding to Figure 2A, the overexpressed genes that are downstream effectors of the Myc signaling network were listed in Supplementary Table S2. Of note, LY6D, ST3GAL4, ASS1, PTP4A3 (also named PRL-3), UBE2C (also named UBCH10), and E2F3 are novel oncogenes and biomarkers whose overexpression are related to metastases, migration, or proliferation of NSCLC and other cancers [27–39]. Our data indicated that Myc overexpression resulted in the overall upregulation of a bunch of oncogenes subjected to Myc regulation, which presumably contribute to the enhanced carcinogenesis across the lung and upper bronchial airways.

It has been reported that the basal cells in human prostate cancer use Myc signaling networks in the initiation of tumorigenesis [40]. Moreover, Myc signaling is responsible for the similarities between ESCs (embryonic stem cells) and cancer [41–43] and Myc can induce a basal stem cell/ESC-like transcriptional profile when transduced into keratinocytes [44]. In this study, we also found that the basal stem cell/ESC signature genes characterized previously [45] were upregulated in the airway brush samples from the lung SCC early lesion mice (Figure S3-S4), which could be caused by the upregulation of Myc expression and subsequent activation of Myc network signaling (Figures 1, 2, 3). These results suggest that the bronchial brush airway samples from lung SCC early-lesion mice have more of the basal stem cell characteristics than the non-lesion control mice, which could be an important feature of the initialization phase of the development of lung SCC premalignant lesions and tumors.

As expected, the activated PI3K/AKT signaling was inhibited in bronchial brush samples of lung SCC early-lesion mice by the treatment of XL-147 that is a potent PI3K inhibitor. This is similar to what was found in the human airway brush samples of NSCLC patients, where the elevated PI3K activity was inhibited by myo-inositol, another PI3K inhibitor [10]. We also found that Myc activation can be inhibited by pioglitazone, a PPARγ agonist. Interestingly, the PI3K/AKT pathway activity was not significantly reduced by the treatment of pioglitazone, and Myc pathway activity was not significantly downregulated by the treatment of XL-147. This suggested that the combination therapy using both XL-147 and pioglitazone treatment may complement each other and thus achieve better efficacy in preventing lung SCC. Our previous research showed that pioglitazone significantly inhibited progression of both lung adenocarcinoma and SCC in the two mouse model systems [46]. Specifically, pioglitazone significantly inhibited tumor load (sum of tumor volume per lung in average) by 50%–64% (*P* < 0.05) in the lung adenocarcinoma mouse model and caused a 35% decrease (*P* < 0.05) in lung SCC. However, the underlying molecular mechanisms remain elusive. This study provides data suggesting that inhibition of Myc network activity could be a key route for pioglitazone to play its anti-tumor roles in lung SCC.

Our work also revealed a number of significant miRNA-mRNA interactions that can be identified in the bronchial airway samples of lung SCC early-lesion mice, which may play a key role in the early tumorigenesis of lung SCC. In particular, we detected that mmu-miR-449c-5p targeted the *Myc* gene and that mmu-miR-449c-5p expression was negatively correlated with *Myc* expression. The human counterpart of mouse mmu-miR-449c-5p, *i.e*., miR-449c, had been reported to be down-regulated in human cancers, including nasopharyngeal carcinoma [47], gastric carcinoma [48], and NSCLC [49]. *In vitro* cell culture studies showed that miR-449c inhibited gastric carcinoma growth by targeting *MET* [48] and inhibited NSCLC cell progression by targeting *c-Myc* [49]. Our studied support these findings at the *in vivo* level. Furthermore, our results show that miR-449c downregulation and the associated *Myc* activation were early events important to lung SCC development, and can be detected in the normal appearing bronchial airway epithelial cells. Thus, miR-449c expression may be a novel bronchial airway biomarker that can be used for the diagnosis of lung SCC based on the use of non-invasive bronchial brushing samples.

We derived a 36-gene classifier from the bronchial airway samples of lung SCC early-lesion mice and found that this classifier can detect lung cancer cases based on both mice and human bronchial brush samples. The information of the genes from the 36-gene based mouse biomarker panels are given in Table 2. In this classifier, some genes have been reported previously as novel biomarkers for NSCLC. For example, *PPBP* (pro-platelet basic protein, also known as “CTAP-III”) was previously known as a novel blood marker for the NSCLC diagnosis in humans [50, 51]. When comparing the bronchial airways of lung SCC early lesion-mice to the normal mice, the PPBP gene was overexpressed 115 fold (Table 2). This is the first report to demonstrate that PPBP is a novel biomarker in the bronchial airway for NSCLC diagnosis. *Mcl-1* is an antiapoptotic member of the Bcl-2 family frequently upregulated in NSCLC and promotes lung cancer cell migration [52]. *FOSL2* belongs to the AP-1 transcription factor family and facilitates TGF-β1-induced migration of NSCLC tumors [53]. *MARCKS* is significantly overexpressed in human lung SCC relative to adjacent non-cancer tissue and has been identified as a prognostic biomarker in human primary lung SCC [54]. *MARCKS* was also reported to play a crucial role in potentiating lung cancer cell migration/metastasis [55]. *MAP4K4* expression is closely associated with NSCLC progression and has an independent prognostic value in lung adenocarcinoma [56]. *CLCA2* has been found to be a novel blood biomarker for NSCLC diagnosis and prognosis [57, 58]. A number of established lung cancer oncogenes have also been included in the classifier, such as *IL1R2*, *JUNB*, *EGFR*, *SOX9*, *FOSB*, and *JUND*.

**Table 2:** Description of the 36-gene classifier derived from the mouse lung SCC early lesion model that are used for the diagnosis of human lung cancer by bronchial airway samples

Gene	Name	KEGG_PATHWAY	FoldChange mouseLCvsNormal	*P* Value
PPBP	pro-platelet basic protein (chemokine (C-X-C motif) ligand 7)	hsa04062:Chemokine signaling pathway	115.0	3.4E-22
IL1R2	interleukin 1 receptor, type II	hsa04010:MAPK signaling pathway	7.6	2.8E-12
HILPDA	hypoxia inducible lipid droplet associated		6.6	5.1E-15
CWH43	hypothetical protein FLJ21511		5.9	2.5E-24
JUNB	jun B proto-oncogene		5.6	1.2E-13
EGR1	early growth response 1	hsa05020:Prion diseases	5.4	2.8E-08
MCL1	myeloid cell leukemia sequence 1 (BCL2-related)		4.8	5.0E-15
FOSL2	FOS-like antigen 2		3.7	1.0E-21
MARCKS	myristoylated alanine-rich protein kinase C substrate	hsa04666:Fc gamma R-mediated phagocytosis	3.6	1.2E-07
SIDT2	SID1 transmembrane family, member 2		3.3	3.1E-14
BHLHE41	basic helix-loop-helix family, member e41	hsa04710:Circadian rhythm	3.3	7.3E-04
PDLIM4	PDZ and LIM domain 4		3.1	8.2E-04
HTRA1	HtrA serine peptidase 1		3.1	1.1E-05
MAP4K4	mitogen-activated protein kinase kinase kinase kinase 4	hsa04010:MAPK signaling pathway	2.7	9.2E-15
EGFR	epidermal growth factor receptor (erythroblastic leukemia viral (v-erb-b) oncogene homolog, avian)	hsa04010:MAPK signaling pathway	2.5	6.5E-08
CYR61	cysteine-rich, angiogenic inducer, 61		2.4	1.2E-03
SOX9	SRY (sex determining region Y)- box 9		2.4	1.2E-04
CALML3	calmodulin-like 3	hsa04070:Phosphatidylinositol signaling systempathway	2.4	1.8E-05
CLCA2	chloride channel accessory 2	hsa04740:Olfactory transduction	2.4	2.4E-03
IQSEC1	IQ motif and Sec7 domain 1	hsa04144:Endocytosis	2.3	6.1E-05
FOSB	FBJ murine osteosarcoma viral oncogene homolog B		2.1	2.5E-02
IER2	immediate early response 2		2.0	3.8E-05
SMC2	structural maintenance of chromosomes 2		1.9	5.5E-03
CA12	carbonic anhydrase XII	hsa00910:Nitrogen metabolism	1.9	9.1E-05
CPE	carboxypeptidase E	hsa04940:Type I diabetes mellitus	1.9	3.0E-05
AVPI1	arginine vasopressin-induced 1		1.7	2.0E-06
TMEM45A	transmembrane protein 45A		1.7	2.0E-03
JUND	jun D proto-oncogene	hsa04010:MAPK signaling pathway	1.7	8.5E-04
CHST15	carbohydrate (N-acetylgalactosamine 4-sulfate 6-O) sulfotransferase 15	hsa00532:Chondroitin sulfate biosynthesis	1.7	9.5E-05
ATG101	autophagy related 101		1.6	1.2E-04
CLTB	clathrin light chain B	hsa04142:Lysosome	1.5	4.3E-04
EFNB2	ephrin-B2	hsa04360:Axon guidance	1.5	7.1E-03
IDS	iduronate 2-sulfatase	hsa00531:Glycosaminoglycan degradation	−1.8	1.6E-07
GCLC	glutamate-cysteine ligase, catalytic subunit	hsa00480:Glutathione metabolism	−2.1	5.7E-07
SERPING1	serpin peptidase inhibitor, clade G (C1 inhibitor), member 1	hsa04610:Complement and coagulation cascades	−2.3	1.1E-05
ANK3	ankyrin 3, node of Ranvier (ankyrin G)		−2.4	3.0E-16

In summary, we detected the activation of the critical oncogenic pathways like PI3K and Myc signaling networks in cytologically normal bronchial airway epithelial cells of mice with preneopastic lung SCC lesions, which can be reversed by treatment with XL-147 and pioglitazone, respectively. We also identified a key microRNA, mmu-miR-449c-5p, whose suppression significantly up-regulated *myc* expression in the normal bronchial airway epithelial cells of mice with early stage SCC lesions. Finally, we developed a novel bronchial genomic classifier in mice that can be used to assist in the diagnosis of human lung cancer. Our findings supported the “field cancerization” theory and suggested key pathways and genes critical to the initiation of lung SCC.

## MATERIALS AND METHODS

### Mice populations and bronchial brush sample collection

For comparison, we set up a negative control group of Swiss mice that were not subjected to any treatment (called ‘Normal’ group). In parallel, the positive control group (only treated with carcinogen) and several drug treatment groups (carcinogene plus drug treatment) of mice were all treated with the carcinogen - NTCU at the same dose and duration of time (treated biweekly with topical 40 mmol/L NTCU for 24 weeks for SCC early-lesion [SCC-EL] model). For sample collection, we did bronchial brushing twice for the carcinogen group (positive control), one at 20 weeks (called ‘BeforeCarci’) and another at 24 weeks (called ‘AfterCarci’). We also collected brush samples for the drug treatment groups of Swiss mice at the same time points. The first drug group samples are composed of the brush samples obtained from the lung SCC early lesion mice before and after the extended 4-week NTCU plus XL-147 treatment (i.e., 20 weeks mice [called ‘BeforeXL147′] vs 24 weeks mice [called ‘AfterXL147′], XL-147 dosage is 100 mg/Kg BW/day). The second drug group samples consist of the brush samples obtained from the lung SCC early lesion mice before and after the extended 4-week NTCU plus pioglitazone treatment (i.e., 20 weeks mice [called ‘BeforePio’] vs 24 weeks mice [called ‘AfterPio’], pioglitazone dosage is 15 mg/kg body weight via oral gavage). Therefore, there are a total of 7 groups of brush airways samples, i.e., ‘Normal’, ‘BeforeCarci’, ‘AfterCarci’, ‘BeforeXL147′, ‘AfterXL147′, ‘BeforePio’, ‘AfterPio’ groups, with each group having 6 samples subjected to RNA-sequencing.

### RNA processing, RNA-seq and miRNA-seq experimentation

Total RNA samples were extracted from the airway brush samples using Qiagen (Valencia, CA) RNeasy® Mini Kit. The quality of the total RNA samples obtained was very high, with RIN (RNA integrity number) values in the range of 9-10. Total RNA samples were divided into two aliquots for RNA-seq and miRNA-seq experiments separately. We used TruSeq RNA Library Preparation Kit v2 and TruSeq Small RNA Library Preparation Kits from Illumina (San Diego, CA) to construct the RNA-seq and miRNA-seq libraries, respectively. The sequencing of these RNA-seq and miRNA-seq library samples was performed in The Medical College of Wisconsin (MCW) Human and Molecular Genetics Center (HMGC) Sequencing Core using the HiSeq 2500 platforms (Illumina, San Diego, CA). The reads generated were single-end with 50 nucleotides in length. The qualities of the RNA-seq and miRNA-seq reads were analyzed using the FastQC program (http://www.bioinformatics.babraham.ac.uk/projects/fastqc/). The coverages ranged from 15 million to 32 million reads per RNA-seq sample and 3 million to 5 million reads per miRNA-seq sample. The quality scores of > 95.3% of all the bases of each sample are > 30, averaging around 40, greatly exceeding the threshold of 20.

### RNA-seq and miRNA-seq reads alignment and differential expression analysis

The pre-processed sample RNA-seq reads were aligned to the mm9 mouse genome (UCSC version, July 2007) using Bowtie-TopHat (version 2.0.4, segment length 29nt, 1 mismatch in segment permitted, for maximum sensitivity, coverage search performed [59, 60]). Read counts were obtained using HTseq [61]. Batch effects were adjusted using the R package - RUVSeq [62]. Data normalization and differential expression analysis were performed using the statistical algorithms implemented in the statistical R packages - edgeR and limma [20, 21]. FDR (False discovery rate) corrected *p*-values of less than 0.05 were used as criteria for significantly regulated genes. We used FastQC [63] to check the quality of raw sequences after the miRNA sequencing based on the HiSeq 2500 platform. Cutadapt [64] was used to remove adapter and unwanted sequences from raw data for miRNA-seq reads. Then FastQC was used again to recheck the quality of the preprocessed reads. The mapping of the trimmed high quality miRNA-seq reads was performed using Chimira [65], which is a fast and robust system for the cleaning, filtering, quality control, and mapping of miRNA-Seq data. The initially mapped miRNA-seq reads were further adjusted for the confounding factors, like batch effects, and normalized by the individual library sizes using the software programs RUVSeq [62] and edgeR [21]. Similar to RNA-seq, the differential expression analysis of miRNA was performed using the statistical algorithms implemented in the statistical R packages edgeR and limma [20, 21].

### Oncogenic pathway analysis and activation analysis

The list of differentially expressed genes in the bronchial airway samples between lung SCC early-lesion mice and non-lesion control mice was analyzed by IPA (http://www.ingenuity.com). The two major modules in IPA, *i.e*., ‘Canonical Pathways’ and ‘Networks’, were adopted for pathway and network enrichment analysis. GSEA [66] analysis was performed for the pre-ranked differentially expressed genes using the option ‘GseaPreranked’. In GSEA, 1000 permutations were used to calculate significance. A gene set was considered to be significantly enriched in one of the two groups when the *P value* was lower than 0.05 and the FDR was lower than 0.25 for the corresponding gene set. For inferring and comparing the activity of the oncogenic pathways across different groups, we utilized the statistical R package DART [22, 67]. This software uses a perturbation expression signature database encompassing perturbations of over 90 cancer genes, in combination with a novel statistical denoising algorithm, to help discern perturbations of the oncogenic pathways.

### Integrative analysis of the RNA-seq and miRNA-seq data for the airway brush samples from the lung SCC early-lesion mice

As the starting point we used three basic assumptions in the miRNA-mRNA combination analysis [25]: i) miRNAs negatively regulate expression of their mRNA targets; ii) miRNA/mRNA interactions, as they are based on RNA hybridization, can be predicted with bioinformatic approaches; iii) miRNAs and mRNAs that play a role in a specific diseases are deregulated in that disease. We used an R software package - MiRComb [25] - to conduct the analysis of miRNA-mRNA interactions based on the RNA-seq and miRNA-seq data we generated for the airway brush samples from the lung SCC early-lesion mice and the normal control group.

### Development and validation of a bronchial genomic classifier from lung SCC early lesion mice

We first developed a 36-gene classifier using the *cancerclass* R package [68] based on the analysis of the RNA-seq data of the bronchial airway samples from a mouse cohort comprising 12 lung SCC mice and 11 normal mice available from this study. Then we validated the accuracy of this classifier for the diagnosis of lung cancer using the two downloaded human microarray datasets of bronchial airway samples obtained from smokers with and without lung cancer. These two datasets are deposited in the GEO database with Accession # of GSE4115 [10] and GSE19027 [69]. Dataset GSE4115 comprised 90 smokers without cancer and 97 smokers with lung cancer. Dataset GSE19027 comprised 29 smokers without cancer and 21 smokers with lung cancer. We conducted preprocessing of these raw microarray data, data normalization and filtering, and differential expression analyses using the *limma* R package [20]. The performance of the mouse-derived classifier was evaluated with the use of receiver-operating-characteristic curves, calculation of AUC [70], and estimates of sensitivity and specificity implemented in the *cancerclass* R package [71]. This classification protocol starts with a feature selection step and continues with nearest-centroid classification. The accurarcy of the predictor can be evaluated using training and test set validation, leave-one-out cross-validation or in a multiple random validation protocol. Fisher's exact test was used for categorical variables. All confidence intervals are reported as two-sided binomial 95% confidence intervals. Statistical analysis was performed with R software, version 3.1.2 (R Project for Statistical Computing).

## SUPPLEMENTARY MATERIALS FIGURES AND TABLES








