# Supplementary material for: Bronchial airway gene expression signatures in mouse lung squamous cell carcinoma and their modulation by cancer chemopreventive agents
Source: Oncotarget. 2016 Dec 7;8(12):18885–900. doi: 10.18632/oncotarget.13806 (PMC5386655; doi:10.18632/oncotarget.13806)
Supplement: Supplementary file 1 [file oncotarget-08-18885-s001.pdf]

Bronchial airway gene expression signatures in mouse lung squamous cell carcinoma and their modulation by cancer chemopreventive agents

Supplementary Materials

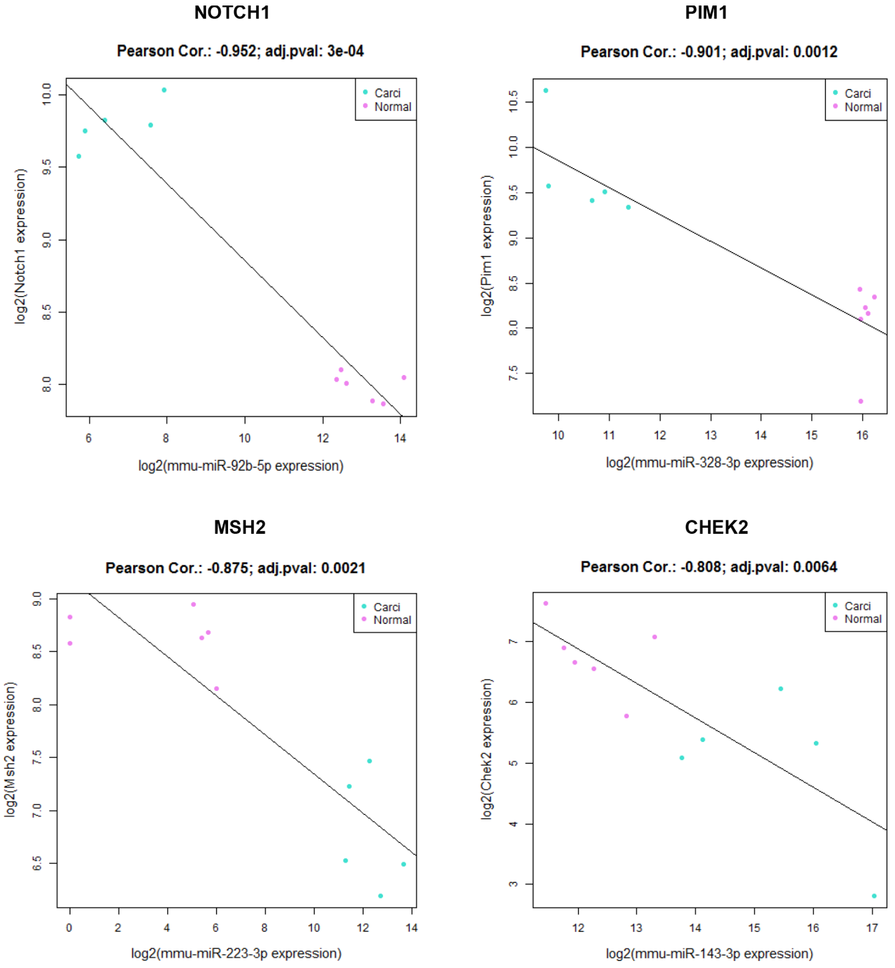

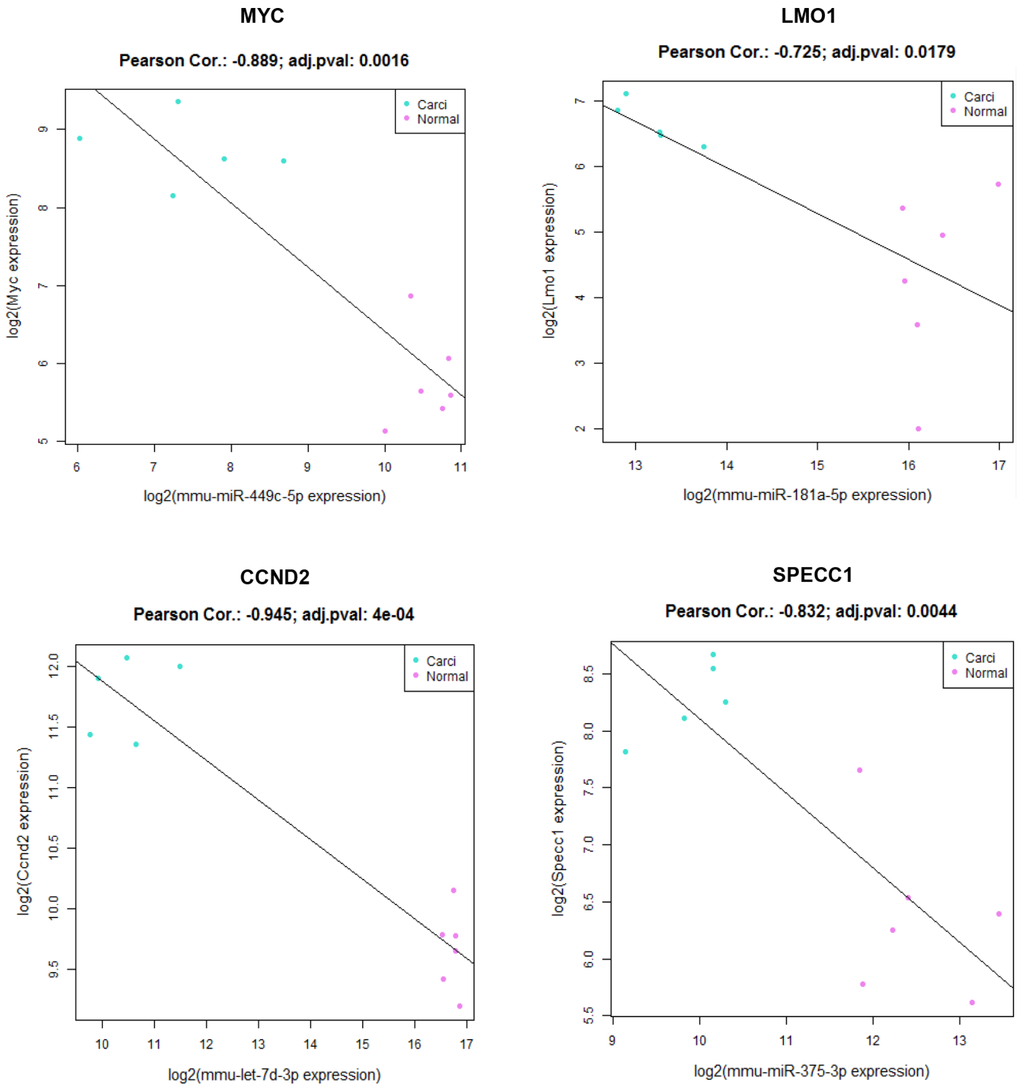

Supplementary Figure S1: Plot of the negative correlations of the six oncogenes – Myc, Lmo1, Ccnd2, Specc1, Notch1, Pim1 and two tumor suppressor genes – Msh2 and Chek2 with their most significant correlated miRNAs. Plot of the negative correlations of the six oncogenes – Myc, Lmo1, Ccnd2, Specc1, Notch1, Pim1 and two tumor suppressor genes – Msh2 and Chek2 with their most significant correlated miRNAs.

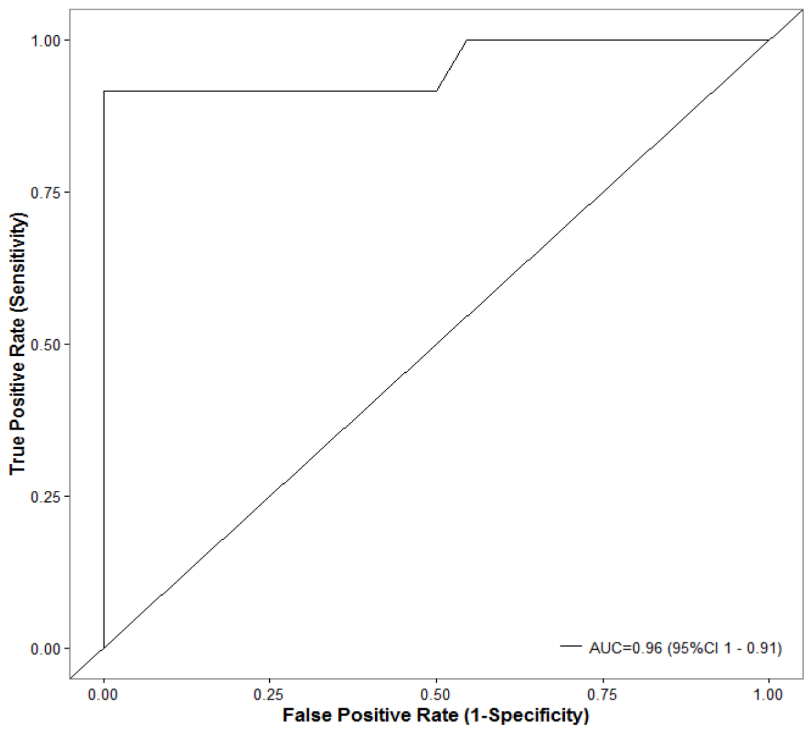

Supplementary Figure S2: ROC curve analysis of the training set cohort of lung SCC mice using the finalized gene expression classifier for Figure 7. The AUC was calculated as 0.96 (95% CI, 0.91-1) for the NSCLC mice cohort (12 airway samples from mice with preneoplastic lung SCC lesions + 11 airway samples from healthy mice without any lesions or tumors).

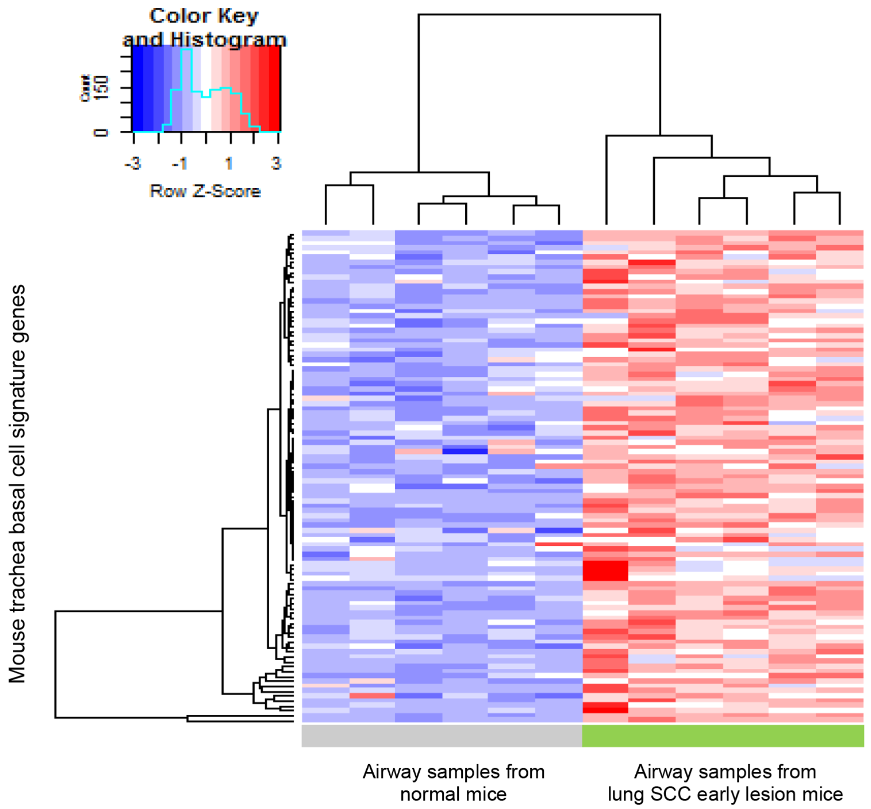

Supplementary Figure S3: Unsupervised hierarchial cluster analysis of mouse airway brush samples from mice with preneoplastic SCC lesions versus no-lesion control mice based on the expression of the mouse trachea basal cell-signature genes previously detected [45].

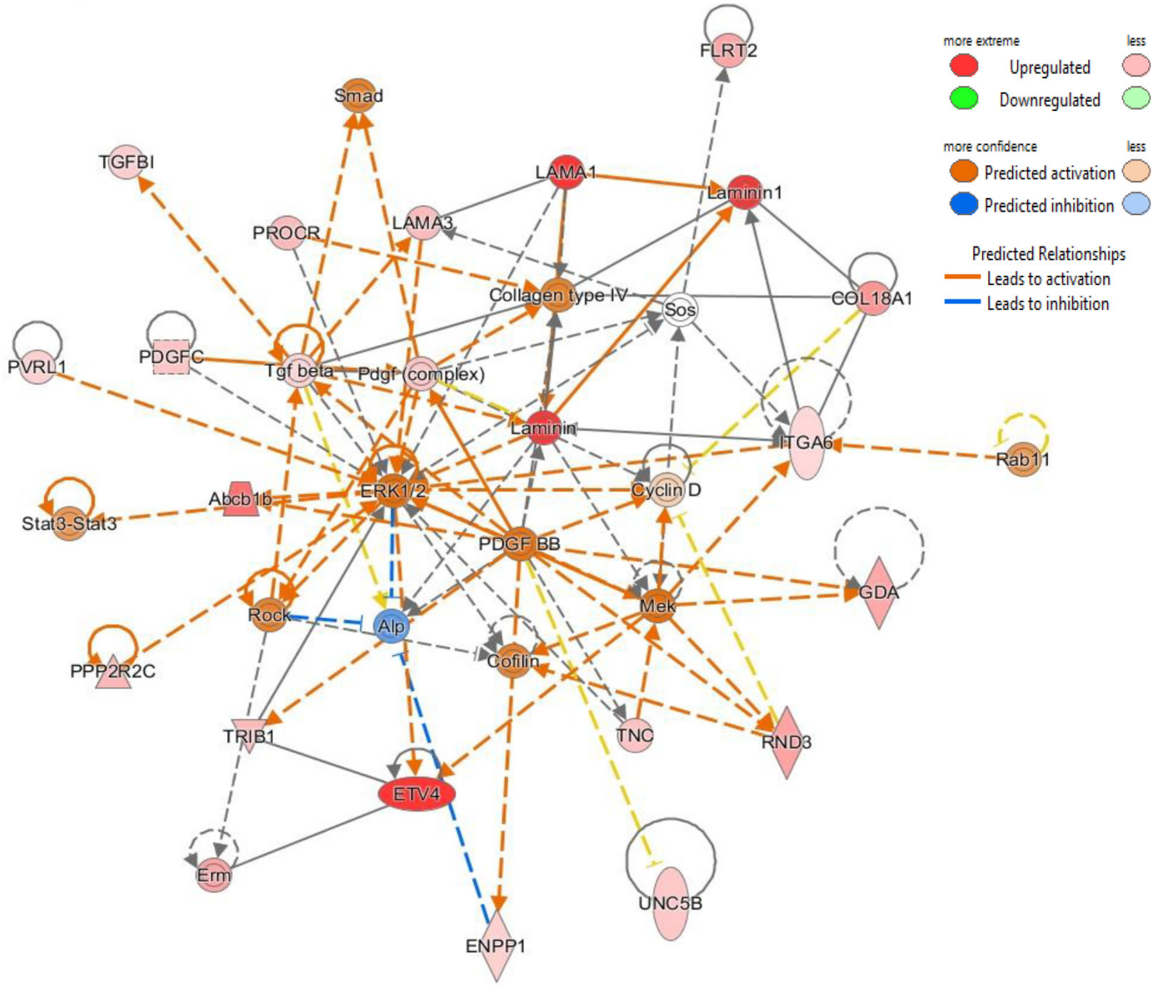

Supplementary Figure S4: Network analysis of the upregulated basal cell signature genes [45] in the bronchial airway brush samples from murine preneoplastic lung SCC lesions showed that the ESC network was predicted to be activated. The analysis was performed using the IPA software.

Supplementary Table S1: The information of the overexpressed genes in the bronchial airway epithelial cells from NCTU-treated mice that are downstream effectors of the Myc signaling network

Human gene symbol	Entrez Gene Name	Fold change	*P* value	Location
PHLDB2	pleckstrin homology-like domain, family B, member 2	5.2	5.07E-07	Cytoplasm
GBP4	guanylate binding protein 4	3.7	1.03E-03	Cytoplasm
LY6D	lymphocyte antigen 6 complex, locus D	3.6	9.80E-11	Plasma Membrane
ST3GAL4	ST3 beta-galactoside alpha-2,3-sialyltransferase 4	3.4	5.07E-19	Cytoplasm
SRD5A1	steroid-5-alpha-reductase, alpha polypeptide 1 (3-oxo-5 alpha-steroid delta 4-dehydrogenase alpha 1)	3.3	8.40E-05	Cytoplasm
ASS1	argininosuccinate synthase 1	3.2	3.05E-12	Cytoplasm
PGAP1	post-GPI attachment to proteins 1	2.4	1.97E-03	Cytoplasm
PTP4A3	protein tyrosine phosphatase type IVA, member 3	2.4	2.68E-09	Plasma Membrane
UBE2C	ubiquitin conjugating enzyme E2C	2.2	9.11E-03	Cytoplasm
TANGO6	transport and golgi organization 6 homolog	2.2	1.60E-02	Other
ASF1B	anti-silencing function 1B histone chaperone	2.2	5.25E-03	Nucleus
MYBL2	v-myb avian myeloblastosis viral oncogene homolog-like 2	2.0	2.06E-02	Nucleus
TONSL	tonsoku-like, DNA repair protein	1.9	1.66E-02	Cytoplasm
MYO1C	myosin IC	1.8	6.77E-09	Cytoplasm
TBC1D15	TBC1 domain family member 15	1.6	4.71E-05	Cytoplasm
APCDD1	adenomatosis polyposis coli down-regulated 1	1.6	6.60E-03	Plasma Membrane
SLIRP	SRA stem-loop interacting RNA binding protein	1.6	1.16E-04	Cytoplasm
E2F3	E2F transcription factor 3	1.6	5.87E-03	Nucleus
SPIN1	spindlin 1	1.5	2.66E-06	Nucleus
RNF115	ring finger protein 115	1.5	2.37E-05	Cytoplasm
YRDC	yrdC N(6)-threonylcarbamoyltransferase domain containing	1.5	3.50E-03	Other
MDN1	midasin AAA ATPase 1	1.5	2.00E-02	Nucleus

Supplementary Table S2: Detailed information of the 1,135 differential expressed genes caused by XL-147 treatment, which consisted of 603 significantly down-regulated genes and 532 significantly up-regulated genes after XL-147 treatment. See Supplymentary_Table_S2

Supplementary Table S3:Detailed information of the 3,018 differential expressed genes caused by pioglitazone treatment, which consisted of 1,491 significantly down-regulated genes and 1,527 significantly up-regulated genes after pioglitazone treatment. See Supplymentary_Table_S3

Supplementary Table S4: Detailed information of the 2,335 miRNA-mRNA pairs that were significantly negative correlated in expression. See Supplymentary_Table_S4
