# Supplementary material for: Bronchial airway gene expression signatures in mouse lung squamous cell carcinoma and their modulation by cancer chemopreventive agents
Source: Oncotarget. 2016 Dec 7;8(12):18885–900. doi: 10.18632/oncotarget.13806 (PMC5386655; doi:10.18632/oncotarget.13806)
Supplement: Supplementary file 2 [file oncotarget-08-18885-s002.docx]

Table S2. Detailed information of the 1,135 differential expressed genes caused by XL-147 treatment, which consisted of 603 significantly down-regulated genes and 532 significantly up-regulated genes after XL-147 treatment. logFC (i.e., log2 Fold change in RNA-seq data comparing XL-147 treated samples vs non-treated samples), P value and FDR (false discovery rate) were given for each gene. All the genes had FDR < 0.05 to be considered significant to adjust for multiple testing issue. MGIsymbol and HGNCsymbol mean the mouse gene name and corresponding human gene name. The mouse genes without corresponding human homolog genes were labeled ‘NA’ in the “HGNCsymbol” column.

| MGIsymbol | HGNCsymbol | logFC | P value | FDR |
| --- | --- | --- | --- | --- |
| Car3 | CA3 | -4.97 | 1.40E-05 | 7.81E-04 |
| 2310057J18Rik | C6orf58 | -4.77 | 8.34E-05 | 3.16E-03 |
| Slc5a7 | SLC5A7 | -4.68 | 8.60E-07 | 9.04E-05 |
| Wnt2b | WNT2B | -4.53 | 2.03E-12 | 2.52E-09 |
| Adipoq | ADIPOQ | -4.52 | 4.49E-09 | 1.27E-06 |
| Pck1 | PCK1 | -4.14 | 1.69E-06 | 1.52E-04 |
| 1190003K10Rik | NA | -4.13 | 1.53E-05 | 8.35E-04 |
| Scgb2b12 | NA | -4.12 | 3.02E-03 | 4.43E-02 |
| Lipf | LIPF | -4.09 | 3.79E-04 | 1.01E-02 |
| Pgr | PGR | -3.98 | 2.77E-05 | 1.32E-03 |
| Cfd | CFD | -3.97 | 1.03E-05 | 6.25E-04 |
| Scgb2b19 | NA | -3.88 | 1.88E-03 | 3.20E-02 |
| Sox10 | SOX10 | -3.85 | 5.07E-05 | 2.17E-03 |
| Epha5 | EPHA5 | -3.84 | 1.84E-05 | 9.40E-04 |
| Arhgap36 | ARHGAP36 | -3.76 | 7.65E-06 | 4.96E-04 |
| Mcidas | MCIDAS | -3.73 | 2.57E-07 | 3.31E-05 |
| Crisp3 | NA | -3.72 | 1.05E-14 | 2.71E-11 |
| Bricd5 | BRICD5 | -3.64 | 1.19E-04 | 4.10E-03 |
| Plin1 | PLIN1 | -3.64 | 1.27E-07 | 1.98E-05 |
| Dcpp2 | NA | -3.60 | 2.25E-05 | 1.10E-03 |
| Tusc5 | TUSC5 | -3.59 | 1.29E-05 | 7.29E-04 |
| Svopl | SVOPL | -3.59 | 1.74E-07 | 2.48E-05 |
| 6330403K07Rik | NA | -3.57 | 2.16E-06 | 1.80E-04 |
| Lrrc3b | LRRC3B | -3.57 | 1.72E-05 | 9.05E-04 |
| Fam198a | FAM198A | -3.53 | 8.42E-08 | 1.41E-05 |
| Ccno | CCNO | -3.52 | 1.07E-08 | 2.57E-06 |
| Tdo2 | TDO2 | -3.52 | 1.11E-04 | 3.90E-03 |
| Cdc20b | CDC20B | -3.47 | 6.66E-06 | 4.48E-04 |
| Frmpd4 | FRMPD4 | -3.45 | 7.53E-04 | 1.69E-02 |
| Shox2 | SHOX2 | -3.43 | 2.06E-04 | 6.29E-03 |
| Phex | PHEX | -3.43 | 1.48E-08 | 3.19E-06 |
| Agtr2 | AGTR2 | -3.43 | 2.39E-05 | 1.16E-03 |
| Igj | NA | -3.31 | 8.19E-04 | 1.80E-02 |
| Agbl1 | AGBL1 | -3.30 | 3.87E-04 | 1.03E-02 |
| Dcaf12l1 | DCAF12L2 | -3.30 | 1.46E-04 | 4.82E-03 |
| Asb10 | ASB10 | -3.27 | 2.39E-05 | 1.16E-03 |
| Edn3 | EDN3 | -3.27 | 1.19E-03 | 2.38E-02 |
| Cidec | CIDEC | -3.26 | 2.57E-04 | 7.53E-03 |
| Myoc | MYOC | -3.25 | 8.24E-06 | 5.21E-04 |
| Foxn4 | FOXN4 | -3.23 | 1.07E-05 | 6.39E-04 |
| Myh13 | MYH13 | -3.22 | 2.18E-06 | 1.80E-04 |
| Lrrc4b | LRRC4B | -3.22 | 8.13E-05 | 3.11E-03 |
| Dmbt1 | DMBT1 | -3.21 | 1.00E-04 | 3.60E-03 |
| Klhl33 | KLHL33 | -3.21 | 2.03E-04 | 6.21E-03 |
| Mstn | MSTN | -3.20 | 4.94E-04 | 1.25E-02 |
| Epha3 | EPHA3 | -3.19 | 3.05E-03 | 4.45E-02 |
| Scgb2b7 | NA | -3.19 | 4.87E-04 | 1.23E-02 |
| Nell2 | NELL2 | -3.18 | 5.36E-04 | 1.32E-02 |
| Scg3 | SCG3 | -3.18 | 1.99E-04 | 6.16E-03 |
| Kank4 | KANK4 | -3.11 | 7.39E-06 | 4.83E-04 |
| Klhl4 | KLHL4 | -3.11 | 4.81E-04 | 1.22E-02 |
| Crispld1 | CRISPLD1 | -3.10 | 2.24E-04 | 6.70E-03 |
| Tmem132c | TMEM132C | -3.09 | 2.19E-06 | 1.80E-04 |
| Susd5 | SUSD5 | -3.07 | 1.30E-04 | 4.40E-03 |
| Dcpp3 | NA | -3.06 | 9.59E-06 | 5.90E-04 |
| Adcyap1r1 | ADCYAP1R1 | -3.05 | 6.89E-09 | 1.82E-06 |
| Sorcs1 | SORCS1 | -3.05 | 4.49E-06 | 3.22E-04 |
| Myadml2 | MYADML2 | -3.04 | 2.09E-03 | 3.46E-02 |
| Cplx1 | CPLX1 | -3.04 | 2.54E-04 | 7.48E-03 |
| Sln | NA | -3.01 | 1.47E-03 | 2.74E-02 |
| Frem1 | FREM1 | -2.99 | 2.92E-03 | 4.34E-02 |
| Kbtbd12 | KBTBD12 | -2.99 | 5.44E-04 | 1.33E-02 |
| Bpifb1 | BPIFB1 | -2.98 | 1.62E-03 | 2.91E-02 |
| Drd1a | NA | -2.97 | 1.41E-08 | 3.14E-06 |
| Ptgfr | PTGFR | -2.96 | 7.22E-05 | 2.85E-03 |
| Fbxo40 | FBXO40 | -2.96 | 1.56E-03 | 2.85E-02 |
| 4930444F02Rik | NA | -2.95 | 1.73E-03 | 3.02E-02 |
| Chodl | CHODL | -2.91 | 8.61E-05 | 3.21E-03 |
| Epyc | EPYC | -2.91 | 1.17E-06 | 1.17E-04 |
| Tnnt1 | TNNT1 | -2.90 | 7.20E-07 | 7.89E-05 |
| Dcpp1 | NA | -2.89 | 6.68E-08 | 1.17E-05 |
| Pkhd1l1 | PKHD1L1 | -2.89 | 1.59E-03 | 2.88E-02 |
| Scgb2b20 | NA | -2.88 | 7.29E-04 | 1.66E-02 |
| Ckmt2 | CKMT2 | -2.88 | 3.17E-03 | 4.56E-02 |
| Bhlha15 | BHLHA15 | -2.87 | 8.60E-11 | 4.78E-08 |
| Gja3 | GJA3 | -2.85 | 4.82E-05 | 2.08E-03 |
| AI848285 | NA | -2.84 | 1.09E-08 | 2.57E-06 |
| Ngb | NGB | -2.82 | 1.26E-06 | 1.23E-04 |
| Inmt | INMT-FAM188B | -2.80 | 1.38E-09 | 5.23E-07 |
| Prr32 | PRR32 | -2.79 | 2.28E-03 | 3.68E-02 |
| Tril | TRIL | -2.77 | 6.86E-07 | 7.62E-05 |
| Kcna1 | KCNA1 | -2.77 | 7.32E-04 | 1.66E-02 |
| Myoz3 | MYOZ3 | -2.75 | 2.04E-03 | 3.39E-02 |
| Pde3a | PDE3A | -2.74 | 6.07E-04 | 1.46E-02 |
| Tbx15 | TBX15 | -2.73 | 9.44E-04 | 1.99E-02 |
| Pax1 | PAX1 | -2.71 | 1.47E-05 | 8.11E-04 |
| Gdf10 | GDF10 | -2.71 | 2.35E-04 | 7.01E-03 |
| Runx1t1 | RUNX1T1 | -2.70 | 1.26E-04 | 4.31E-03 |
| B4galnt2 | B4GALNT2 | -2.70 | 4.37E-06 | 3.16E-04 |
| Tmem182 | TMEM182 | -2.69 | 2.12E-03 | 3.48E-02 |
| Spon1 | SPON1 | -2.67 | 7.37E-07 | 8.02E-05 |
| Slc26a7 | SLC26A7 | -2.66 | 3.40E-03 | 4.77E-02 |
| Egf | EGF | -2.66 | 2.33E-07 | 3.07E-05 |
| Mamstr | MAMSTR | -2.66 | 3.10E-03 | 4.50E-02 |
| Myog | MYOG | -2.66 | 1.99E-03 | 3.34E-02 |
| Tbxa2r | TBXA2R | -2.66 | 7.43E-04 | 1.68E-02 |
| A2m | A2M | -2.65 | 3.25E-03 | 4.64E-02 |
| Olfml1 | OLFML1 | -2.65 | 7.89E-06 | 5.09E-04 |
| Frzb | FRZB | -2.65 | 1.45E-06 | 1.37E-04 |
| Cd209f | NA | -2.64 | 7.76E-04 | 1.72E-02 |
| Snph | SNPH | -2.64 | 3.24E-05 | 1.49E-03 |
| Ccdc42 | CCDC42 | -2.62 | 1.62E-03 | 2.90E-02 |
| Ido1 | IDO1 | -2.61 | 2.74E-05 | 1.31E-03 |
| Lmod3 | LMOD3 | -2.59 | 3.52E-03 | 4.87E-02 |
| Rasd2 | RASD2 | -2.57 | 4.52E-08 | 8.79E-06 |
| Prdm6 | PRDM6 | -2.57 | 1.08E-04 | 3.80E-03 |
| Thrsp | THRSP | -2.56 | 7.95E-09 | 2.06E-06 |
| Ccna1 | CCNA1 | -2.56 | 1.96E-04 | 6.07E-03 |
| Myocd | MYOCD | -2.55 | 6.54E-05 | 2.60E-03 |
| Stxbp5l | STXBP5L | -2.55 | 6.02E-05 | 2.49E-03 |
| Musk | MUSK | -2.54 | 1.88E-03 | 3.20E-02 |
| Abca8b | ABCA8 | -2.54 | 1.61E-06 | 1.47E-04 |
| BC006965 | NA | -2.53 | 2.95E-04 | 8.44E-03 |
| Twist2 | TWIST2 | -2.48 | 5.95E-06 | 4.13E-04 |
| Gabra4 | GABRA4 | -2.47 | 2.71E-03 | 4.15E-02 |
| Hhip | HHIP | -2.45 | 1.15E-04 | 4.01E-03 |
| 2810468N07Rik | NA | -2.43 | 2.16E-03 | 3.53E-02 |
| Pitx2 | PITX2 | -2.43 | 1.23E-03 | 2.43E-02 |
| A530099J19Rik | NA | -2.43 | 3.41E-03 | 4.77E-02 |
| Nags | NAGS | -2.42 | 2.51E-04 | 7.42E-03 |
| Colq | COLQ | -2.40 | 5.16E-04 | 1.28E-02 |
| Srpx | SRPX | -2.40 | 1.69E-03 | 2.97E-02 |
| Mfap4 | MFAP4 | -2.39 | 3.22E-07 | 4.05E-05 |
| Btn1a1 | BTN1A1 | -2.38 | 1.21E-03 | 2.41E-02 |
| Gprasp2 | GPRASP2 | -2.36 | 1.31E-03 | 2.52E-02 |
| Iigp1 | NA | -2.36 | 3.08E-04 | 8.67E-03 |
| Aoc3 | AOC3 | -2.36 | 2.08E-06 | 1.76E-04 |
| Cntfr | CNTFR | -2.36 | 6.63E-06 | 4.48E-04 |
| Retnla | RETNLB | -2.36 | 9.82E-04 | 2.05E-02 |
| Ripply1 | RIPPLY1 | -2.35 | 1.84E-03 | 3.16E-02 |
| Zfp366 | ZNF366 | -2.34 | 2.01E-03 | 3.36E-02 |
| Adora1 | ADORA1 | -2.33 | 8.58E-06 | 5.34E-04 |
| Slc25a34 | SLC25A34 | -2.32 | 1.72E-03 | 3.01E-02 |
| Retn | RETN | -2.32 | 2.84E-03 | 4.26E-02 |
| Cyp2e1 | CYP2E1 | -2.30 | 4.85E-04 | 1.23E-02 |
| Cys1 | CYS1 | -2.30 | 7.81E-04 | 1.73E-02 |
| C1qtnf2 | C1QTNF2 | -2.29 | 2.63E-04 | 7.65E-03 |
| Cyp2d9 | CYP2D7 | -2.29 | 5.17E-04 | 1.28E-02 |
| Opcml | OPCML | -2.28 | 7.96E-04 | 1.76E-02 |
| Mme | MME | -2.28 | 9.73E-07 | 1.00E-04 |
| Tmem255a | TMEM255A | -2.27 | 1.35E-03 | 2.59E-02 |
| Derl3 | DERL3 | -2.26 | 1.26E-05 | 7.21E-04 |
| Chrm2 | CHRM2 | -2.25 | 8.02E-04 | 1.77E-02 |
| Robo4 | ROBO4 | -2.24 | 4.55E-04 | 1.17E-02 |
| Slc2a13 | SLC2A13 | -2.23 | 2.28E-03 | 3.68E-02 |
| Cyp4a12a | CYP4A11 | -2.22 | 3.10E-10 | 1.54E-07 |
| Fam13c | FAM13C | -2.21 | 5.14E-04 | 1.28E-02 |
| Pax7 | PAX7 | -2.21 | 3.63E-03 | 4.98E-02 |
| Actg2 | ACTG2 | -2.21 | 1.12E-04 | 3.91E-03 |
| Zcchc5 | ZCCHC5 | -2.21 | 2.19E-03 | 3.56E-02 |
| Avpr1a | AVPR1A | -2.20 | 6.34E-04 | 1.51E-02 |
| Gfra3 | GFRA3 | -2.20 | 3.64E-03 | 4.98E-02 |
| Cxcl12 | CXCL12 | -2.19 | 2.32E-07 | 3.07E-05 |
| Lman1l | LMAN1L | -2.17 | 2.81E-11 | 1.90E-08 |
| F2rl3 | F2RL3 | -2.14 | 2.34E-03 | 3.74E-02 |
| Apol6 | APOL6 | -2.14 | 3.93E-05 | 1.76E-03 |
| Dmrta1 | DMRTA1 | -2.12 | 2.88E-05 | 1.36E-03 |
| Dchs1 | DCHS1 | -2.12 | 2.96E-05 | 1.39E-03 |
| Nkx3-1 | NKX3-1 | -2.12 | 4.68E-08 | 8.87E-06 |
| C1qtnf7 | C1QTNF7 | -2.12 | 6.77E-04 | 1.58E-02 |
| Perm1 | PERM1 | -2.12 | 1.82E-04 | 5.71E-03 |
| Prss2 | PRSS1 | -2.11 | 1.72E-05 | 9.05E-04 |
| Kcnma1 | KCNMA1 | -2.11 | 8.97E-05 | 3.28E-03 |
| Asb16 | ASB16 | -2.10 | 3.30E-03 | 4.67E-02 |
| Rcan2 | RCAN2 | -2.10 | 2.81E-04 | 8.07E-03 |
| Sbk2 | SBK2 | -2.10 | 1.46E-03 | 2.74E-02 |
| Gpr124 | NA | -2.09 | 8.29E-06 | 5.22E-04 |
| Dpt | DPT | -2.08 | 1.07E-05 | 6.39E-04 |
| Adcy5 | ADCY5 | -2.08 | 2.36E-04 | 7.01E-03 |
| Txlnb | TXLNB | -2.08 | 1.42E-03 | 2.69E-02 |
| Tlr12 | NA | -2.07 | 1.16E-03 | 2.33E-02 |
| Fbln7 | FBLN7 | -2.07 | 1.44E-05 | 7.98E-04 |
| Upk3a | UPK3A | -2.07 | 1.79E-05 | 9.29E-04 |
| Slc12a8 | SLC12A8 | -2.07 | 8.04E-06 | 5.17E-04 |
| Atp1a2 | ATP1A2 | -2.07 | 2.79E-03 | 4.21E-02 |
| Ptgdr | PTGDR | -2.07 | 7.26E-06 | 4.81E-04 |
| Slc38a5 | SLC38A5 | -2.07 | 1.81E-03 | 3.12E-02 |
| Pycr1 | PYCR1 | -2.06 | 3.13E-04 | 8.80E-03 |
| Tff2 | TFF2 | -2.05 | 3.96E-06 | 2.93E-04 |
| Cnn1 | CNN1 | -2.05 | 9.01E-05 | 3.29E-03 |
| Tagln | TAGLN | -2.04 | 7.48E-05 | 2.93E-03 |
| Cox7a1 | COX7A1 | -2.03 | 6.03E-05 | 2.49E-03 |
| Chrdl1 | CHRDL1 | -2.03 | 2.75E-04 | 7.94E-03 |
| Klhl13 | KLHL13 | -2.02 | 4.54E-05 | 1.99E-03 |
| Pgm5 | PGM5 | -2.00 | 4.93E-06 | 3.50E-04 |
| Fcrls | FCRL2 | -2.00 | 9.19E-05 | 3.34E-03 |
| 1810041L15Rik | KIAA1644 | -2.00 | 3.44E-04 | 9.51E-03 |
| Cyp2d22 | CYP2D6 | -1.98 | 2.28E-08 | 4.60E-06 |
| Gxylt2 | GXYLT2 | -1.98 | 3.60E-04 | 9.81E-03 |
| Fmo1 | FMO1 | -1.98 | 1.37E-06 | 1.33E-04 |
| Abat | ABAT | -1.97 | 7.61E-05 | 2.95E-03 |
| Aass | AASS | -1.97 | 4.51E-05 | 1.98E-03 |
| Scube1 | SCUBE1 | -1.96 | 3.80E-06 | 2.88E-04 |
| Mfap2 | MFAP2 | -1.96 | 1.60E-04 | 5.11E-03 |
| Rgs7 | RGS7 | -1.95 | 7.72E-04 | 1.71E-02 |
| Pdgfd | PDGFD | -1.94 | 3.12E-03 | 4.51E-02 |
| Aifm3 | AIFM3 | -1.94 | 3.35E-03 | 4.73E-02 |
| Ank2 | ANK2 | -1.93 | 2.08E-04 | 6.33E-03 |
| Rasl12 | RASL12 | -1.92 | 5.34E-04 | 1.32E-02 |
| Myh11 | MYH11 | -1.92 | 4.02E-05 | 1.79E-03 |
| Sfrp2 | SFRP2 | -1.92 | 1.70E-05 | 9.03E-04 |
| Hspb2 | HSPB2 | -1.91 | 1.27E-03 | 2.48E-02 |
| Gmnc | GMNC | -1.91 | 8.81E-05 | 3.25E-03 |
| Maob | MAOB | -1.91 | 1.06E-04 | 3.74E-03 |
| Acnat1 | NA | -1.90 | 8.23E-05 | 3.13E-03 |
| Olfml3 | OLFML3 | -1.90 | 2.03E-05 | 1.01E-03 |
| Tmtc1 | TMTC1 | -1.89 | 6.84E-06 | 4.56E-04 |
| Sh3bgr | SH3BGR | -1.89 | 2.05E-03 | 3.40E-02 |
| Abca8a | NA | -1.88 | 2.59E-06 | 2.08E-04 |
| Ogn | OGN | -1.86 | 2.73E-09 | 9.05E-07 |
| Slc4a5 | SLC4A5 | -1.86 | 1.08E-05 | 6.39E-04 |
| Dner | DNER | -1.86 | 1.19E-04 | 4.12E-03 |
| Kcnb1 | KCNB1 | -1.85 | 2.45E-03 | 3.87E-02 |
| Ndn | NDN | -1.85 | 1.33E-04 | 4.49E-03 |
| Ppp1r3c | PPP1R3C | -1.85 | 2.02E-04 | 6.21E-03 |
| Abi3bp | ABI3BP | -1.84 | 5.75E-09 | 1.54E-06 |
| Rcn3 | RCN3 | -1.83 | 8.85E-06 | 5.46E-04 |
| Lum | LUM | -1.83 | 1.39E-08 | 3.13E-06 |
| Mrvi1 | MRVI1 | -1.82 | 1.71E-05 | 9.05E-04 |
| Foxf2 | FOXF2 | -1.82 | 2.58E-04 | 7.53E-03 |
| Zfp385c | ZNF385C | -1.82 | 1.91E-04 | 5.95E-03 |
| Cpa3 | CPA3 | -1.82 | 1.88E-03 | 3.20E-02 |
| Scn4b | SCN4B | -1.81 | 2.59E-03 | 4.03E-02 |
| Aplnr | APLNR | -1.81 | 1.46E-03 | 2.74E-02 |
| Acta2 | ACTA2 | -1.81 | 1.25E-05 | 7.14E-04 |
| Mylk | MYLK | -1.81 | 5.92E-06 | 4.13E-04 |
| Prkg1 | PRKG1 | -1.81 | 1.44E-03 | 2.72E-02 |
| Aldh1a2 | ALDH1A2 | -1.80 | 8.09E-05 | 3.10E-03 |
| Fgf16 | FGF16 | -1.80 | 1.36E-03 | 2.60E-02 |
| Sgce | SGCE | -1.79 | 3.67E-04 | 9.94E-03 |
| Inpp5j | INPP5J | -1.79 | 3.33E-04 | 9.26E-03 |
| Ces1g | CES1 | -1.77 | 7.47E-05 | 2.93E-03 |
| Kcnk3 | KCNK3 | -1.76 | 2.66E-03 | 4.11E-02 |
| B3gnt9 | B3GNT9 | -1.76 | 1.74E-03 | 3.03E-02 |
| 1700019D03Rik | C2orf88 | -1.76 | 1.84E-03 | 3.17E-02 |
| Plin4 | PLIN4 | -1.76 | 1.99E-03 | 3.34E-02 |
| Synpo2 | SYNPO2 | -1.75 | 1.40E-04 | 4.69E-03 |
| Actc1 | ACTC1 | -1.74 | 9.89E-04 | 2.06E-02 |
| Sfrp4 | SFRP4 | -1.73 | 3.97E-04 | 1.05E-02 |
| 5330417C22Rik | KIAA1324 | -1.73 | 1.47E-06 | 1.38E-04 |
| Sntg2 | SNTG2 | -1.73 | 3.20E-03 | 4.59E-02 |
| Tet1 | TET1 | -1.71 | 2.65E-03 | 4.10E-02 |
| Pdgfra | PDGFRA | -1.71 | 1.68E-06 | 1.51E-04 |
| Bche | BCHE | -1.71 | 1.58E-06 | 1.44E-04 |
| Csdc2 | CSDC2 | -1.70 | 1.45E-05 | 8.03E-04 |
| Fxyd6 | FXYD6-FXYD2 | -1.70 | 4.20E-06 | 3.08E-04 |
| Lama2 | LAMA2 | -1.69 | 1.96E-04 | 6.07E-03 |
| Rspo3 | RSPO3 | -1.69 | 2.52E-03 | 3.95E-02 |
| Prrx1 | PRRX1 | -1.69 | 2.77E-05 | 1.32E-03 |
| Sardh | SARDH | -1.69 | 2.16E-04 | 6.51E-03 |
| Creb3l1 | CREB3L1 | -1.69 | 3.51E-07 | 4.30E-05 |
| Igf2 | IGF2 | -1.68 | 8.98E-05 | 3.28E-03 |
| Lrrc17 | LRRC17 | -1.68 | 6.16E-04 | 1.47E-02 |
| Scn7a | SCN7A | -1.67 | 2.43E-03 | 3.85E-02 |
| Mpped2 | MPPED2 | -1.67 | 1.64E-03 | 2.93E-02 |
| Kcnk2 | KCNK2 | -1.67 | 2.18E-05 | 1.07E-03 |
| Mcpt4 | NA | -1.67 | 3.27E-03 | 4.65E-02 |
| Itga1 | ITGA1 | -1.67 | 1.28E-04 | 4.35E-03 |
| Muc5b | MUC5B | -1.67 | 4.90E-06 | 3.50E-04 |
| Asgr1 | ASGR1 | -1.66 | 8.83E-04 | 1.89E-02 |
| Foxa3 | FOXA3 | -1.65 | 1.58E-03 | 2.86E-02 |
| Col15a1 | COL15A1 | -1.65 | 2.44E-07 | 3.16E-05 |
| Atp2a3 | ATP2A3 | -1.65 | 2.59E-07 | 3.31E-05 |
| Nrxn1 | NRXN1 | -1.65 | 1.11E-03 | 2.26E-02 |
| Slc6a17 | SLC6A17 | -1.64 | 1.45E-03 | 2.74E-02 |
| Smgc | NA | -1.62 | 5.14E-04 | 1.28E-02 |
| Gp2 | GP2 | -1.62 | 5.50E-05 | 2.32E-03 |
| Tmem47 | TMEM47 | -1.62 | 1.24E-03 | 2.45E-02 |
| Tesc | TESC | -1.61 | 1.57E-04 | 5.05E-03 |
| Igsf10 | IGSF10 | -1.61 | 1.24E-03 | 2.45E-02 |
| Casq2 | CASQ2 | -1.61 | 7.54E-04 | 1.69E-02 |
| D10Bwg1379e | NA | -1.61 | 7.01E-04 | 1.62E-02 |
| Tmem200b | TMEM200B | -1.60 | 1.20E-03 | 2.39E-02 |
| Twist1 | TWIST1 | -1.60 | 2.03E-03 | 3.39E-02 |
| Myrip | MYRIP | -1.59 | 2.33E-03 | 3.72E-02 |
| Prss32 | NA | -1.59 | 1.16E-07 | 1.83E-05 |
| Hmcn1 | HMCN1 | -1.59 | 1.85E-03 | 3.17E-02 |
| Ism1 | ISM1 | -1.58 | 2.18E-04 | 6.54E-03 |
| Flnc | FLNC | -1.58 | 3.58E-03 | 4.94E-02 |
| Cx3cr1 | CX3CR1 | -1.58 | 1.14E-04 | 3.99E-03 |
| Col4a6 | COL4A6 | -1.58 | 2.78E-11 | 1.90E-08 |
| Dkk2 | DKK2 | -1.58 | 6.69E-05 | 2.65E-03 |
| Scd1 | SCD | -1.58 | 4.23E-04 | 1.11E-02 |
| Myl9 | MYL9 | -1.57 | 1.16E-04 | 4.01E-03 |
| Creb3l4 | CREB3L4 | -1.57 | 1.46E-04 | 4.82E-03 |
| C1s2 | C1S | -1.57 | 1.48E-03 | 2.75E-02 |
| Dpp6 | DPP6 | -1.56 | 3.46E-03 | 4.81E-02 |
| Gng7 | GNG7 | -1.55 | 1.63E-03 | 2.92E-02 |
| Muc19 | NA | -1.54 | 5.74E-04 | 1.39E-02 |
| Chad | CHAD | -1.54 | 1.15E-05 | 6.72E-04 |
| Sema3d | SEMA3D | -1.54 | 1.54E-04 | 5.00E-03 |
| Wscd2 | WSCD2 | -1.53 | 1.05E-03 | 2.16E-02 |
| Isg15 | ISG15 | -1.53 | 1.80E-03 | 3.12E-02 |
| Ntrk2 | NTRK2 | -1.53 | 9.30E-05 | 3.37E-03 |
| Ablim2 | ABLIM2 | -1.53 | 1.65E-03 | 2.94E-02 |
| Ddr2 | DDR2 | -1.52 | 1.27E-03 | 2.49E-02 |
| Lifr | LIFR | -1.52 | 5.35E-07 | 6.21E-05 |
| Dhtkd1 | DHTKD1 | -1.52 | 2.80E-03 | 4.21E-02 |
| Ncald | NCALD | -1.51 | 6.08E-06 | 4.17E-04 |
| Ddx60 | DDX60 | -1.51 | 3.44E-04 | 9.51E-03 |
| Padi2 | PADI2 | -1.50 | 2.62E-03 | 4.07E-02 |
| Shisa2 | SHISA2 | -1.49 | 1.57E-03 | 2.86E-02 |
| Gprin3 | GPRIN3 | -1.49 | 3.08E-03 | 4.50E-02 |
| Col14a1 | COL14A1 | -1.48 | 1.50E-04 | 4.90E-03 |
| Ccdc129 | CCDC129 | -1.47 | 4.22E-08 | 8.31E-06 |
| Lims2 | LIMS2 | -1.47 | 3.76E-04 | 1.01E-02 |
| Dact3 | DACT3 | -1.47 | 2.52E-04 | 7.42E-03 |
| Scgb3a2 | SCGB3A2 | -1.46 | 3.59E-04 | 9.80E-03 |
| Lmod1 | LMOD1 | -1.46 | 4.25E-05 | 1.88E-03 |
| Mmp11 | MMP11 | -1.46 | 1.53E-04 | 4.99E-03 |
| Nynrin | NYNRIN | -1.45 | 4.51E-04 | 1.16E-02 |
| Ankrd1 | ANKRD1 | -1.45 | 3.35E-03 | 4.73E-02 |
| Reln | RELN | -1.45 | 3.41E-03 | 4.77E-02 |
| Aox3 | NA | -1.45 | 7.08E-08 | 1.22E-05 |
| Trim46 | TRIM46 | -1.45 | 4.93E-05 | 2.11E-03 |
| Bmpr1b | BMPR1B | -1.45 | 2.47E-04 | 7.32E-03 |
| Pygb | PYGB | -1.45 | 2.25E-09 | 7.77E-07 |
| Stxbp6 | STXBP6 | -1.45 | 1.34E-04 | 4.53E-03 |
| Lgi2 | LGI2 | -1.43 | 2.04E-11 | 1.67E-08 |
| Gnb3 | GNB3 | -1.42 | 2.85E-03 | 4.26E-02 |
| Fhod3 | FHOD3 | -1.42 | 1.28E-03 | 2.49E-02 |
| Clip3 | CLIP3 | -1.42 | 3.19E-03 | 4.58E-02 |
| Ptprn2 | PTPRN2 | -1.41 | 7.26E-04 | 1.65E-02 |
| Armcx2 | ARMCX2 | -1.41 | 8.49E-04 | 1.84E-02 |
| Acsm3 | ACSM3 | -1.40 | 4.45E-06 | 3.20E-04 |
| Dcn | DCN | -1.40 | 6.34E-07 | 7.20E-05 |
| Epas1 | EPAS1 | -1.40 | 1.14E-06 | 1.15E-04 |
| Upk3b | UPK3B | -1.40 | 3.10E-03 | 4.50E-02 |
| Smad9 | SMAD9 | -1.39 | 1.35E-03 | 2.59E-02 |
| Ern2 | ERN2 | -1.39 | 3.86E-04 | 1.03E-02 |
| Eln | ELN | -1.39 | 4.97E-04 | 1.25E-02 |
| Wnk2 | WNK2 | -1.38 | 2.01E-04 | 6.19E-03 |
| Npr2 | NPR2 | -1.38 | 3.12E-03 | 4.51E-02 |
| Prelp | PRELP | -1.38 | 3.54E-05 | 1.61E-03 |
| Acacb | ACACB | -1.38 | 6.27E-04 | 1.49E-02 |
| Olfml2a | OLFML2A | -1.37 | 1.54E-06 | 1.43E-04 |
| Trabd2b | TRABD2B | -1.37 | 9.55E-04 | 2.01E-02 |
| Sod3 | SOD3 | -1.37 | 1.46E-04 | 4.82E-03 |
| Rarres2 | RARRES2 | -1.36 | 3.45E-05 | 1.57E-03 |
| Adhfe1 | ADHFE1 | -1.36 | 1.50E-03 | 2.78E-02 |
| Jam2 | JAM2 | -1.35 | 6.59E-04 | 1.55E-02 |
| Gata3 | GATA3 | -1.34 | 3.11E-03 | 4.51E-02 |
| Irgm2 | IRGM | -1.34 | 5.20E-04 | 1.29E-02 |
| Cldn8 | CLDN8 | -1.34 | 1.84E-06 | 1.59E-04 |
| Slc25a23 | SLC25A23 | -1.34 | 6.40E-07 | 7.22E-05 |
| Fbxl16 | FBXL16 | -1.33 | 2.37E-03 | 3.77E-02 |
| Galnt16 | GALNT16 | -1.33 | 9.65E-04 | 2.02E-02 |
| Frem2 | FREM2 | -1.33 | 3.42E-03 | 4.78E-02 |
| C1ra | C1R | -1.33 | 6.08E-06 | 4.17E-04 |
| Cacna2d1 | CACNA2D1 | -1.33 | 2.13E-03 | 3.49E-02 |
| Rad51b | RAD51B | -1.33 | 7.34E-05 | 2.88E-03 |
| Rerg | RERG | -1.33 | 3.06E-06 | 2.41E-04 |
| Myh10 | MYH10 | -1.32 | 4.79E-04 | 1.22E-02 |
| Enpp2 | ENPP2 | -1.32 | 1.66E-04 | 5.25E-03 |
| Slc25a21 | SLC25A21 | -1.32 | 1.69E-03 | 2.98E-02 |
| Cldn10 | CLDN10 | -1.31 | 2.11E-04 | 6.41E-03 |
| Gpm6b | GPM6B | -1.31 | 1.67E-03 | 2.96E-02 |
| Lgr6 | LGR6 | -1.30 | 3.16E-04 | 8.85E-03 |
| Celf3 | CELF3 | -1.30 | 2.63E-03 | 4.07E-02 |
| Sspn | SSPN | -1.29 | 1.45E-04 | 4.82E-03 |
| Bmp4 | BMP4 | -1.29 | 4.09E-05 | 1.82E-03 |
| Smoc2 | SMOC2 | -1.29 | 8.00E-05 | 3.08E-03 |
| Pdgfrb | PDGFRB | -1.29 | 2.09E-03 | 3.46E-02 |
| Slc10a5 | SLC10A5 | -1.28 | 1.73E-05 | 9.08E-04 |
| Acsf2 | ACSF2 | -1.27 | 3.75E-10 | 1.64E-07 |
| Serpinf1 | SERPINF1 | -1.27 | 4.86E-05 | 2.09E-03 |
| Mtss1l | MTSS1L | -1.26 | 1.06E-05 | 6.38E-04 |
| Lama4 | LAMA4 | -1.25 | 7.10E-04 | 1.63E-02 |
| Snhg18 | NA | -1.25 | 7.61E-04 | 1.70E-02 |
| Amigo2 | AMIGO2 | -1.25 | 2.74E-03 | 4.17E-02 |
| Sparcl1 | SPARCL1 | -1.25 | 8.51E-05 | 3.19E-03 |
| Islr | ISLR | -1.25 | 4.27E-04 | 1.11E-02 |
| Acsm1 | ACSM1 | -1.24 | 1.00E-06 | 1.02E-04 |
| Kazald1 | KAZALD1 | -1.24 | 2.74E-03 | 4.17E-02 |
| Fcgbp | FCGBP | -1.24 | 3.22E-04 | 8.99E-03 |
| C1s1 | C1S | -1.24 | 1.11E-04 | 3.89E-03 |
| Adamtsl1 | ADAMTSL1 | -1.23 | 1.57E-04 | 5.05E-03 |
| Copz2 | COPZ2 | -1.23 | 8.50E-04 | 1.84E-02 |
| Pcp4l1 | PCP4L1 | -1.23 | 6.44E-04 | 1.52E-02 |
| Nsg1 | NA | -1.23 | 2.17E-03 | 3.54E-02 |
| Plscr4 | PLSCR4 | -1.23 | 5.14E-04 | 1.28E-02 |
| Scara5 | SCARA5 | -1.22 | 4.46E-04 | 1.16E-02 |
| Paqr8 | PAQR8 | -1.21 | 1.26E-04 | 4.31E-03 |
| Hectd2 | HECTD2 | -1.21 | 3.27E-03 | 4.65E-02 |
| Igfbp4 | IGFBP4 | -1.20 | 8.34E-08 | 1.41E-05 |
| Etv1 | ETV1 | -1.19 | 6.82E-04 | 1.59E-02 |
| Itga8 | ITGA8 | -1.18 | 1.65E-03 | 2.94E-02 |
| C4b | C4A | -1.18 | 4.30E-04 | 1.12E-02 |
| Gbp3 | GBP4 | -1.17 | 5.44E-04 | 1.33E-02 |
| Ltbp4 | LTBP4 | -1.17 | 9.05E-09 | 2.27E-06 |
| Mrc2 | MRC2 | -1.17 | 3.43E-03 | 4.78E-02 |
| Gpr153 | GPR153 | -1.17 | 2.75E-03 | 4.18E-02 |
| Id3 | ID3 | -1.16 | 2.95E-05 | 1.39E-03 |
| Adck3 | ADCK3 | -1.16 | 5.64E-04 | 1.38E-02 |
| Arhgef25 | ARHGEF25 | -1.16 | 3.25E-03 | 4.64E-02 |
| Col4a5 | COL4A5 | -1.15 | 1.87E-06 | 1.61E-04 |
| Csrp2 | CSRP2 | -1.14 | 7.41E-06 | 4.83E-04 |
| Fabp3 | FABP3 | -1.14 | 1.72E-03 | 3.01E-02 |
| Ppm1k | PPM1K | -1.14 | 1.55E-04 | 5.02E-03 |
| Edaradd | EDARADD | -1.13 | 8.26E-04 | 1.81E-02 |
| Fmo3 | FMO3 | -1.13 | 2.23E-03 | 3.61E-02 |
| Slco4c1 | SLCO4C1 | -1.12 | 2.96E-03 | 4.37E-02 |
| Nrep | NREP | -1.12 | 2.49E-03 | 3.91E-02 |
| Mpdz | MPDZ | -1.12 | 8.07E-04 | 1.78E-02 |
| Sytl3 | SYTL3 | -1.12 | 3.02E-03 | 4.43E-02 |
| Mturn | NA | -1.11 | 5.88E-04 | 1.42E-02 |
| Tnfrsf19 | TNFRSF19 | -1.11 | 7.71E-05 | 2.98E-03 |
| Muc1 | MUC1 | -1.11 | 2.71E-05 | 1.30E-03 |
| Hspg2 | HSPG2 | -1.11 | 5.92E-05 | 2.46E-03 |
| Itih5 | ITIH5 | -1.11 | 3.62E-03 | 4.97E-02 |
| Ptpru | PTPRU | -1.10 | 7.05E-07 | 7.78E-05 |
| Ptprd | PTPRD | -1.10 | 3.20E-03 | 4.59E-02 |
| Slc9a7 | SLC9A7 | -1.10 | 1.65E-03 | 2.94E-02 |
| Khdrbs3 | KHDRBS3 | -1.10 | 2.77E-03 | 4.20E-02 |
| Gabrp | GABRP | -1.10 | 2.97E-04 | 8.46E-03 |
| Dmpk | DMPK | -1.09 | 3.04E-04 | 8.61E-03 |
| Igfbp5 | IGFBP5 | -1.09 | 2.56E-04 | 7.51E-03 |
| Wfdc1 | WFDC1 | -1.09 | 7.18E-04 | 1.64E-02 |
| Sned1 | SNED1 | -1.09 | 9.52E-04 | 2.00E-02 |
| Slc1a2 | SLC1A2 | -1.09 | 1.50E-03 | 2.78E-02 |
| Tnfsf10 | TNFSF10 | -1.09 | 1.23E-05 | 7.08E-04 |
| Plcb4 | PLCB4 | -1.08 | 2.13E-05 | 1.05E-03 |
| Slc2a4 | SLC2A4 | -1.08 | 8.92E-04 | 1.91E-02 |
| Ano1 | ANO1 | -1.08 | 2.24E-08 | 4.58E-06 |
| Spats2l | SPATS2L | -1.07 | 2.11E-03 | 3.47E-02 |
| Mamdc2 | MAMDC2 | -1.06 | 3.36E-03 | 4.73E-02 |
| AW112010 | NA | -1.06 | 1.25E-04 | 4.27E-03 |
| Foxp2 | FOXP2 | -1.06 | 7.87E-07 | 8.44E-05 |
| Acss1 | ACSS1 | -1.06 | 1.42E-06 | 1.37E-04 |
| Plk4 | PLK4 | -1.06 | 7.61E-04 | 1.70E-02 |
| Nr3c2 | NR3C2 | -1.06 | 1.89E-04 | 5.91E-03 |
| Dleu2 | NA | -1.05 | 1.70E-03 | 2.98E-02 |
| Faah | FAAH | -1.05 | 1.40E-03 | 2.66E-02 |
| Medag | MEDAG | -1.05 | 3.46E-04 | 9.54E-03 |
| Ifit1 | IFIT1B | -1.05 | 1.61E-03 | 2.90E-02 |
| Rbms3 | RBMS3 | -1.04 | 3.83E-04 | 1.02E-02 |
| Fmo5 | FMO5 | -1.04 | 1.62E-05 | 8.66E-04 |
| Igdcc4 | IGDCC4 | -1.04 | 1.39E-03 | 2.64E-02 |
| Thy1 | THY1 | -1.04 | 2.94E-05 | 1.39E-03 |
| Sntb1 | SNTB1 | -1.03 | 1.49E-04 | 4.89E-03 |
| Ahcyl2 | AHCYL2 | -1.03 | 2.25E-05 | 1.10E-03 |
| Fgfr3 | FGFR3 | -1.03 | 1.46E-07 | 2.17E-05 |
| Mycl | MYCL | -1.01 | 1.60E-07 | 2.30E-05 |
| Tmem211 | TMEM211 | -1.01 | 1.81E-03 | 3.12E-02 |
| Hdac5 | HDAC5 | -1.00 | 9.80E-06 | 6.00E-04 |
| C3 | C3 | -1.00 | 1.37E-04 | 4.60E-03 |
| H2-T23 | HLA-E | -0.99 | 4.15E-04 | 1.09E-02 |
| Tfcp2l1 | TFCP2L1 | -0.98 | 1.09E-05 | 6.45E-04 |
| Cd34 | CD34 | -0.98 | 1.86E-03 | 3.19E-02 |
| Isl1 | ISL1 | -0.97 | 1.60E-05 | 8.57E-04 |
| Tbc1d8b | TBC1D8B | -0.96 | 4.56E-05 | 1.99E-03 |
| Lpar1 | LPAR1 | -0.95 | 1.91E-03 | 3.22E-02 |
| Tc2n | TC2N | -0.95 | 2.76E-04 | 7.94E-03 |
| Cldn2 | CLDN2 | -0.95 | 1.46E-03 | 2.74E-02 |
| Adcy2 | ADCY2 | -0.94 | 1.57E-03 | 2.86E-02 |
| Paqr4 | PAQR4 | -0.94 | 5.29E-06 | 3.74E-04 |
| Fgfr1 | FGFR1 | -0.94 | 4.24E-04 | 1.11E-02 |
| Dmd | DMD | -0.94 | 2.96E-03 | 4.37E-02 |
| Tns1 | TNS1 | -0.94 | 1.01E-04 | 3.62E-03 |
| Kank2 | KANK2 | -0.93 | 6.69E-05 | 2.65E-03 |
| Bach2 | BACH2 | -0.92 | 3.61E-03 | 4.96E-02 |
| Pcolce | PCOLCE | -0.92 | 1.64E-04 | 5.22E-03 |
| Gpc3 | GPC3 | -0.91 | 1.49E-03 | 2.77E-02 |
| Fam46a | FAM46A | -0.91 | 5.44E-04 | 1.33E-02 |
| Tgm2 | TGM2 | -0.91 | 1.63E-04 | 5.18E-03 |
| Atoh8 | ATOH8 | -0.90 | 2.94E-03 | 4.36E-02 |
| Asb13 | ASB13 | -0.90 | 6.87E-06 | 4.56E-04 |
| Cx3cl1 | CX3CL1 | -0.90 | 1.83E-05 | 9.38E-04 |
| Ntn1 | NTN1 | -0.90 | 4.47E-04 | 1.16E-02 |
| Pcca | PCCA | -0.90 | 1.58E-04 | 5.08E-03 |
| Map1lc3a | MAP1LC3A | -0.90 | 5.86E-05 | 2.44E-03 |
| Myb | MYB | -0.90 | 2.59E-03 | 4.03E-02 |
| Postn | POSTN | -0.90 | 2.30E-03 | 3.70E-02 |
| Uba7 | UBA7 | -0.90 | 4.23E-04 | 1.11E-02 |
| Ngfr | NGFR | -0.89 | 1.78E-05 | 9.26E-04 |
| Wdr72 | WDR72 | -0.89 | 1.95E-05 | 9.83E-04 |
| Lzts3 | NA | -0.89 | 1.21E-03 | 2.41E-02 |
| Nnt | NNT | -0.89 | 4.34E-05 | 1.92E-03 |
| Tmtc4 | TMTC4 | -0.89 | 5.85E-04 | 1.42E-02 |
| Plxna3 | PLXNA3 | -0.89 | 1.87E-03 | 3.19E-02 |
| Vipr1 | VIPR1 | -0.89 | 3.24E-06 | 2.49E-04 |
| Ntn4 | NTN4 | -0.89 | 6.47E-05 | 2.59E-03 |
| Chd3 | CHD3 | -0.88 | 1.76E-06 | 1.56E-04 |
| Hdac11 | HDAC11 | -0.88 | 3.53E-03 | 4.89E-02 |
| Sertad4 | SERTAD4 | -0.88 | 6.92E-04 | 1.61E-02 |
| Lypd2 | LYPD2 | -0.88 | 5.04E-04 | 1.27E-02 |
| Lhfp | LHFP | -0.87 | 3.11E-03 | 4.50E-02 |
| Arhgap29 | ARHGAP29 | -0.87 | 9.40E-05 | 3.39E-03 |
| Hid1 | HID1 | -0.87 | 2.18E-03 | 3.56E-02 |
| Arhgap8 | ARHGAP8 | -0.87 | 2.31E-03 | 3.71E-02 |
| Bgn | BGN | -0.87 | 7.22E-04 | 1.65E-02 |
| Cand2 | CAND2 | -0.87 | 1.62E-03 | 2.91E-02 |
| Gstk1 | GSTK1 | -0.87 | 6.22E-05 | 2.53E-03 |
| Efemp1 | EFEMP1 | -0.87 | 3.42E-06 | 2.62E-04 |
| Nmi | NMI | -0.86 | 2.20E-04 | 6.60E-03 |
| Scara3 | SCARA3 | -0.86 | 1.61E-03 | 2.90E-02 |
| Ifih1 | IFIH1 | -0.86 | 1.65E-03 | 2.94E-02 |
| Rac3 | RAC3 | -0.86 | 1.04E-04 | 3.69E-03 |
| Il6st | IL6ST | -0.85 | 1.23E-05 | 7.09E-04 |
| Asap3 | ASAP3 | -0.85 | 2.76E-04 | 7.95E-03 |
| Ovca2 | OVCA2 | -0.85 | 2.71E-03 | 4.15E-02 |
| Galnt5 | GALNT5 | -0.84 | 3.36E-03 | 4.73E-02 |
| Akr1c18 | AKR1C4 | -0.83 | 9.71E-04 | 2.03E-02 |
| Tshz2 | TSHZ2 | -0.83 | 8.52E-06 | 5.32E-04 |
| Ocm | OCM | -0.83 | 1.18E-03 | 2.37E-02 |
| Rbbp9 | RBBP9 | -0.83 | 9.55E-04 | 2.01E-02 |
| Scnn1g | SCNN1G | -0.82 | 1.15E-03 | 2.33E-02 |
| Sel1l3 | SEL1L3 | -0.81 | 1.42E-04 | 4.74E-03 |
| Zfp827 | ZNF827 | -0.81 | 1.23E-03 | 2.43E-02 |
| Setbp1 | SETBP1 | -0.81 | 3.05E-04 | 8.61E-03 |
| Ccdc3 | CCDC3 | -0.81 | 1.10E-04 | 3.89E-03 |
| Kctd14 | KCTD14 | -0.81 | 5.07E-04 | 1.27E-02 |
| Csrp1 | CSRP1 | -0.80 | 7.63E-04 | 1.70E-02 |
| Slc16a13 | SLC16A13 | -0.80 | 8.46E-05 | 3.18E-03 |
| St8sia6 | ST8SIA6 | -0.80 | 1.57E-03 | 2.86E-02 |
| Afap1 | AFAP1 | -0.80 | 8.20E-05 | 3.13E-03 |
| Tln2 | TLN2 | -0.80 | 3.61E-04 | 9.81E-03 |
| Helz2 | HELZ2 | -0.80 | 2.06E-03 | 3.42E-02 |
| Ephb2 | EPHB2 | -0.80 | 1.36E-04 | 4.58E-03 |
| Gprc5a | GPRC5A | -0.80 | 1.92E-03 | 3.24E-02 |
| P2rx7 | P2RX7 | -0.79 | 6.95E-04 | 1.61E-02 |
| Rasef | RASEF | -0.78 | 3.32E-04 | 9.25E-03 |
| Oat | OAT | -0.78 | 6.72E-04 | 1.58E-02 |
| Cnn2 | CNN2 | -0.78 | 8.82E-05 | 3.25E-03 |
| Adh1 | ADH1B | -0.78 | 3.54E-04 | 9.67E-03 |
| Cpq | CPQ | -0.77 | 9.43E-04 | 1.99E-02 |
| Tspan12 | TSPAN12 | -0.77 | 6.14E-04 | 1.47E-02 |
| Pam | PAM | -0.77 | 1.99E-03 | 3.34E-02 |
| Mettl7a1 | METTL7A | -0.76 | 3.30E-03 | 4.67E-02 |
| Ppap2b | PPAP2B | -0.76 | 4.61E-04 | 1.18E-02 |
| Tst | TST | -0.76 | 8.73E-04 | 1.88E-02 |
| Fndc3a | FNDC3A | -0.75 | 9.08E-04 | 1.93E-02 |
| Nme3 | NME3 | -0.75 | 1.25E-03 | 2.46E-02 |
| Hoxb2 | HOXB2 | -0.75 | 3.67E-04 | 9.94E-03 |
| Psmb8 | PSMB8 | -0.74 | 4.16E-04 | 1.09E-02 |
| Creb3l2 | CREB3L2 | -0.74 | 7.22E-04 | 1.65E-02 |
| Fam114a1 | FAM114A1 | -0.74 | 5.87E-04 | 1.42E-02 |
| Mgat3 | MGAT3 | -0.74 | 8.63E-04 | 1.86E-02 |
| Mfge8 | MFGE8 | -0.74 | 3.50E-03 | 4.86E-02 |
| Cadps2 | CADPS2 | -0.74 | 5.51E-04 | 1.35E-02 |
| Srgap3 | SRGAP3 | -0.73 | 1.38E-03 | 2.63E-02 |
| Sidt1 | SIDT1 | -0.71 | 2.17E-03 | 3.54E-02 |
| Myh14 | MYH14 | -0.71 | 3.36E-03 | 4.73E-02 |
| Psmb10 | PSMB10 | -0.71 | 1.09E-03 | 2.24E-02 |
| Tbc1d4 | TBC1D4 | -0.71 | 1.17E-03 | 2.34E-02 |
| Mapk12 | MAPK12 | -0.71 | 1.26E-03 | 2.48E-02 |
| Cyp39a1 | CYP39A1 | -0.70 | 2.11E-04 | 6.41E-03 |
| Gga2 | GGA2 | -0.70 | 2.39E-03 | 3.79E-02 |
| Cav1 | CAV1 | -0.70 | 2.06E-03 | 3.42E-02 |
| Dixdc1 | DIXDC1 | -0.70 | 1.68E-03 | 2.96E-02 |
| Pex6 | PEX6 | -0.70 | 1.47E-03 | 2.74E-02 |
| Mov10 | MOV10 | -0.69 | 2.38E-03 | 3.78E-02 |
| Frat2 | FRAT2 | -0.69 | 3.36E-03 | 4.73E-02 |
| Tns3 | TNS3 | -0.69 | 8.64E-04 | 1.86E-02 |
| Parva | PARVA | -0.69 | 2.10E-03 | 3.46E-02 |
| Fam13b | FAM13B | -0.69 | 2.50E-03 | 3.92E-02 |
| Grasp | GRASP | -0.68 | 9.99E-04 | 2.08E-02 |
| Adcy6 | ADCY6 | -0.68 | 7.19E-04 | 1.64E-02 |
| Mlph | MLPH | -0.68 | 6.42E-04 | 1.52E-02 |
| Aldh6a1 | ALDH6A1 | -0.68 | 5.76E-04 | 1.40E-02 |
| Galnt10 | GALNT10 | -0.67 | 1.10E-03 | 2.25E-02 |
| Eif4a2 | EIF4A2 | -0.67 | 6.40E-04 | 1.52E-02 |
| Ppm1l | PPM1L | -0.67 | 1.84E-03 | 3.17E-02 |
| Slco2a1 | SLCO2A1 | -0.67 | 3.54E-03 | 4.89E-02 |
| Tle1 | TLE1 | -0.67 | 3.37E-03 | 4.73E-02 |
| Csad | CSAD | -0.66 | 1.29E-03 | 2.50E-02 |
| Gpt | GPT | -0.66 | 9.15E-04 | 1.94E-02 |
| Arsb | ARSB | -0.66 | 3.10E-03 | 4.50E-02 |
| Acsl1 | ACSL1 | -0.66 | 2.46E-03 | 3.88E-02 |
| Slc9a4 | SLC9A4 | -0.66 | 2.12E-03 | 3.48E-02 |
| Vill | VILL | -0.66 | 1.55E-03 | 2.84E-02 |
| Hes1 | HES1 | -0.66 | 1.47E-03 | 2.75E-02 |
| 4930523C07Rik | KIAA0040 | -0.65 | 2.75E-03 | 4.18E-02 |
| Nbea | NBEA | -0.64 | 1.54E-03 | 2.82E-02 |
| Decr1 | DECR1 | -0.64 | 2.31E-03 | 3.71E-02 |
| Syne2 | SYNE2 | -0.64 | 2.53E-03 | 3.96E-02 |
| Calcoco1 | CALCOCO1 | -0.64 | 1.52E-03 | 2.80E-02 |
| St6galnac2 | ST6GALNAC2 | -0.64 | 9.81E-04 | 2.05E-02 |
| Rab3ip | NA | -0.64 | 2.09E-03 | 3.46E-02 |
| Ptprs | PTPRS | -0.64 | 1.75E-03 | 3.04E-02 |
| Itpr1 | ITPR1 | -0.64 | 2.43E-03 | 3.84E-02 |
| Slc25a35 | SLC25A35 | -0.63 | 2.51E-03 | 3.94E-02 |
| Met | MET | -0.62 | 1.67E-03 | 2.95E-02 |
| Paics | PAICS | -0.62 | 3.47E-03 | 4.82E-02 |
| Nfia | NFIA | -0.62 | 3.04E-03 | 4.44E-02 |
| Nr2c1 | NR2C1 | -0.62 | 2.68E-03 | 4.12E-02 |
| Pla2g16 | PLA2G16 | -0.62 | 8.83E-04 | 1.89E-02 |
| Tenc1 | NA | -0.61 | 2.03E-03 | 3.38E-02 |
| Itpr3 | ITPR3 | -0.61 | 1.36E-03 | 2.61E-02 |
| Bcat2 | BCAT2 | -0.61 | 1.31E-03 | 2.53E-02 |
| Pbx1 | PBX1 | -0.61 | 8.32E-04 | 1.82E-02 |
| Clstn1 | CLSTN1 | -0.61 | 2.91E-03 | 4.33E-02 |
| Myo10 | MYO10 | -0.60 | 2.91E-03 | 4.34E-02 |
| 2610002M06Rik | CHMP1B | -0.60 | 3.08E-03 | 4.50E-02 |
| Ces1e | CES1 | -0.60 | 2.74E-03 | 4.17E-02 |
| Efcab4a | NA | -0.59 | 9.16E-04 | 1.94E-02 |
| Vtcn1 | VTCN1 | -0.58 | 3.20E-03 | 4.58E-02 |
| Neo1 | NEO1 | -0.57 | 1.51E-03 | 2.78E-02 |
| Mylip | MYLIP | -0.57 | 2.80E-03 | 4.21E-02 |
| Smo | SMO | -0.57 | 1.91E-03 | 3.22E-02 |
| Tanc2 | TANC2 | -0.56 | 3.55E-03 | 4.90E-02 |
| Pkp4 | PKP4 | -0.55 | 2.99E-03 | 4.39E-02 |
| Nr2f2 | NR2F2 | -0.55 | 1.96E-03 | 3.30E-02 |
| Htatip2 | HTATIP2 | 0.53 | 3.09E-03 | 4.50E-02 |
| Pgs1 | PGS1 | 0.56 | 3.34E-03 | 4.72E-02 |
| Serpinb6b | SERPINB6 | 0.56 | 3.37E-03 | 4.73E-02 |
| Slc43a2 | SLC43A2 | 0.57 | 3.40E-03 | 4.76E-02 |
| Pgam1 | PGAM1 | 0.58 | 2.83E-03 | 4.25E-02 |
| Gars | GARS | 0.58 | 2.14E-03 | 3.51E-02 |
| Smap2 | SMAP2 | 0.59 | 2.10E-03 | 3.46E-02 |
| Phlda3 | PHLDA3 | 0.59 | 1.62E-03 | 2.90E-02 |
| Shb | SHB | 0.61 | 1.61E-03 | 2.90E-02 |
| Cbr3 | CBR3 | 0.61 | 3.29E-03 | 4.67E-02 |
| Ccrn4l | CCRN4L | 0.62 | 1.67E-03 | 2.95E-02 |
| Sema4d | SEMA4D | 0.62 | 3.17E-03 | 4.56E-02 |
| Hk2 | HK2 | 0.62 | 1.42E-03 | 2.69E-02 |
| Sbno2 | SBNO2 | 0.63 | 2.83E-03 | 4.25E-02 |
| Map4k4 | MAP4K4 | 0.63 | 1.70E-03 | 2.99E-02 |
| Btg2 | BTG2 | 0.63 | 1.48E-03 | 2.75E-02 |
| Ak2 | AK2 | 0.63 | 1.25E-03 | 2.46E-02 |
| Dusp7 | DUSP7 | 0.63 | 2.19E-03 | 3.56E-02 |
| Slc25a17 | SLC25A17 | 0.63 | 8.57E-04 | 1.86E-02 |
| Marcks | MARCKS | 0.64 | 8.38E-04 | 1.82E-02 |
| Rgs12 | RGS12 | 0.65 | 1.91E-03 | 3.23E-02 |
| Smpd1 | SMPD1 | 0.65 | 1.65E-03 | 2.94E-02 |
| 4833423E24Rik | NA | 0.66 | 3.54E-03 | 4.90E-02 |
| Gba | GBA | 0.67 | 3.03E-03 | 4.43E-02 |
| Plekho2 | PLEKHO2 | 0.68 | 3.12E-03 | 4.51E-02 |
| Itprip | NA | 0.68 | 1.14E-03 | 2.30E-02 |
| Tiparp | TIPARP | 0.68 | 1.36E-03 | 2.60E-02 |
| Grina | GRINA | 0.68 | 2.80E-03 | 4.21E-02 |
| Msmo1 | MSMO1 | 0.69 | 2.74E-03 | 4.17E-02 |
| Fdps | FDPS | 0.69 | 3.09E-03 | 4.50E-02 |
| Pde12 | PDE12 | 0.71 | 8.92E-04 | 1.91E-02 |
| Tgfbi | TGFBI | 0.71 | 8.64E-04 | 1.86E-02 |
| Rab31 | RAB31 | 0.71 | 6.18E-04 | 1.47E-02 |
| Rilpl2 | RILPL2 | 0.71 | 3.21E-03 | 4.59E-02 |
| Hif1a | HIF1A | 0.71 | 3.20E-04 | 8.96E-03 |
| Ctnnbip1 | CTNNBIP1 | 0.71 | 2.57E-03 | 4.01E-02 |
| Fam43a | FAM43A | 0.71 | 3.41E-04 | 9.46E-03 |
| Col18a1 | COL18A1 | 0.71 | 2.57E-03 | 4.01E-02 |
| Dennd4a | DENND4A | 0.72 | 1.51E-03 | 2.79E-02 |
| Gadd45a | GADD45A | 0.72 | 1.55E-03 | 2.83E-02 |
| Mdm2 | MDM2 | 0.73 | 2.14E-04 | 6.47E-03 |
| Gclc | GCLC | 0.73 | 2.90E-03 | 4.33E-02 |
| Lama3 | LAMA3 | 0.74 | 3.92E-04 | 1.04E-02 |
| Prnp | PRNP | 0.74 | 3.31E-03 | 4.68E-02 |
| Fbxw9 | FBXW9 | 0.74 | 1.75E-03 | 3.04E-02 |
| Rnf128 | RNF128 | 0.75 | 2.72E-03 | 4.16E-02 |
| Nucb2 | NUCB2 | 0.75 | 8.46E-05 | 3.18E-03 |
| Galc | GALC | 0.75 | 3.10E-03 | 4.50E-02 |
| Il1rap | IL1RAP | 0.75 | 1.47E-03 | 2.74E-02 |
| Esd | ESD | 0.76 | 1.00E-03 | 2.08E-02 |
| Mpp1 | MPP1 | 0.77 | 1.56E-04 | 5.05E-03 |
| Rab8b | RAB8B | 0.77 | 1.68E-04 | 5.32E-03 |
| Tubb6 | TUBB6 | 0.77 | 1.85E-03 | 3.17E-02 |
| Marcksl1 | MARCKSL1 | 0.77 | 2.36E-03 | 3.76E-02 |
| Dhrs9 | DHRS9 | 0.78 | 6.52E-04 | 1.54E-02 |
| Syne1 | SYNE1 | 0.78 | 2.29E-03 | 3.69E-02 |
| Sqle | SQLE | 0.78 | 1.71E-04 | 5.40E-03 |
| Slc19a2 | SLC19A2 | 0.78 | 7.84E-04 | 1.73E-02 |
| Zfp385a | ZNF385A | 0.79 | 4.65E-04 | 1.19E-02 |
| Il22ra1 | IL22RA1 | 0.79 | 1.01E-03 | 2.09E-02 |
| Fam213b | FAM213B | 0.79 | 2.60E-03 | 4.05E-02 |
| Sipa1l2 | SIPA1L2 | 0.79 | 1.09E-03 | 2.24E-02 |
| Rnf144b | RNF144B | 0.79 | 3.46E-03 | 4.81E-02 |
| Akr1b8 | AKR1B10 | 0.79 | 5.05E-04 | 1.27E-02 |
| Dbi | DBI | 0.79 | 5.66E-04 | 1.38E-02 |
| Fermt3 | FERMT3 | 0.79 | 7.17E-04 | 1.64E-02 |
| Degs1 | DEGS1 | 0.80 | 6.37E-05 | 2.57E-03 |
| Mocos | MOCOS | 0.80 | 1.79E-04 | 5.65E-03 |
| Egln3 | EGLN3 | 0.80 | 2.97E-03 | 4.38E-02 |
| Slc39a6 | SLC39A6 | 0.81 | 1.08E-05 | 6.39E-04 |
| Trp53inp1 | TP53INP1 | 0.81 | 3.06E-04 | 8.65E-03 |
| Rab24 | RAB24 | 0.81 | 3.04E-04 | 8.61E-03 |
| Gadd45g | GADD45G | 0.81 | 9.63E-04 | 2.02E-02 |
| Gda | GDA | 0.82 | 6.63E-04 | 1.56E-02 |
| Ccdc64 | CCDC64 | 0.82 | 2.78E-03 | 4.21E-02 |
| Entpd3 | ENTPD3 | 0.82 | 3.45E-05 | 1.57E-03 |
| Lss | LSS | 0.82 | 1.44E-03 | 2.72E-02 |
| Acat2 | ACAT2 | 0.82 | 1.18E-03 | 2.37E-02 |
| Ppp1r18 | PPP1R18 | 0.82 | 3.13E-04 | 8.80E-03 |
| Sh3bp5 | SH3BP5 | 0.82 | 2.97E-03 | 4.37E-02 |
| Ddit4 | DDIT4 | 0.83 | 4.62E-04 | 1.18E-02 |
| Dcun1d3 | DCUN1D3 | 0.83 | 1.87E-03 | 3.19E-02 |
| Rbp1 | RBP1 | 0.83 | 2.95E-03 | 4.37E-02 |
| Homer3 | HOMER3 | 0.84 | 7.11E-04 | 1.63E-02 |
| Apobr | APOBR | 0.84 | 1.75E-03 | 3.04E-02 |
| C130026I21Rik | SP140 | 0.84 | 1.50E-03 | 2.78E-02 |
| Lsp1 | LSP1 | 0.84 | 2.20E-04 | 6.60E-03 |
| Galnt6 | GALNT6 | 0.84 | 1.30E-03 | 2.51E-02 |
| Ggt6 | GGT6 | 0.85 | 5.85E-04 | 1.42E-02 |
| Gm11992 | C7orf57 | 0.85 | 2.26E-03 | 3.65E-02 |
| Orai2 | ORAI2 | 0.85 | 1.44E-03 | 2.73E-02 |
| Inpp5d | INPP5D | 0.86 | 6.78E-04 | 1.58E-02 |
| Il10ra | IL10RA | 0.86 | 2.35E-03 | 3.76E-02 |
| Slc2a1 | SLC2A1 | 0.86 | 2.16E-05 | 1.07E-03 |
| Klk11 | KLK11 | 0.86 | 5.13E-04 | 1.28E-02 |
| Nckap1l | NCKAP1L | 0.87 | 2.99E-03 | 4.39E-02 |
| Uchl3 | UCHL3 | 0.87 | 6.78E-04 | 1.58E-02 |
| Slfn2 | SLFN12 | 0.87 | 2.63E-04 | 7.65E-03 |
| Clec5a | CLEC5A | 0.88 | 2.65E-03 | 4.10E-02 |
| Mdfi | MDFI | 0.89 | 1.75E-03 | 3.04E-02 |
| Rasa4 | RASA4B | 0.89 | 3.43E-03 | 4.78E-02 |
| Vsig8 | VSIG8 | 0.89 | 2.02E-03 | 3.37E-02 |
| Plekhg1 | PLEKHG1 | 0.89 | 1.30E-03 | 2.51E-02 |
| 4632428N05Rik | C10orf54 | 0.90 | 9.37E-04 | 1.98E-02 |
| Bok | BOK | 0.90 | 1.00E-04 | 3.60E-03 |
| Mt1 | MT1B | 0.90 | 1.54E-03 | 2.82E-02 |
| Mt2 | MT1H | 0.91 | 2.67E-04 | 7.72E-03 |
| Bmp2k | BMP2K | 0.91 | 1.82E-05 | 9.38E-04 |
| Cd36 | CD36 | 0.91 | 2.49E-04 | 7.36E-03 |
| Spsb1 | SPSB1 | 0.91 | 2.01E-03 | 3.36E-02 |
| Sh3bp2 | SH3BP2 | 0.92 | 1.47E-04 | 4.83E-03 |
| Gpx2 | GPX2 | 0.92 | 6.25E-05 | 2.53E-03 |
| Gla | GLA | 0.92 | 4.53E-04 | 1.17E-02 |
| Cdh3 | CDH3 | 0.92 | 9.13E-05 | 3.32E-03 |
| St6galnac4 | ST6GALNAC4 | 0.92 | 2.98E-03 | 4.38E-02 |
| Mybl2 | MYBL2 | 0.92 | 1.36E-03 | 2.60E-02 |
| Sh3kbp1 | SH3KBP1 | 0.93 | 1.21E-03 | 2.41E-02 |
| Hck | HCK | 0.93 | 2.64E-03 | 4.10E-02 |
| Hcls1 | HCLS1 | 0.94 | 1.55E-04 | 5.03E-03 |
| Cd68 | CD68 | 0.94 | 6.98E-04 | 1.61E-02 |
| Spag1 | SPAG1 | 0.94 | 2.97E-04 | 8.46E-03 |
| Atp6v1c2 | ATP6V1C2 | 0.94 | 7.54E-04 | 1.69E-02 |
| Kcnab2 | KCNAB2 | 0.94 | 1.27E-03 | 2.48E-02 |
| Them5 | THEM5 | 0.94 | 2.30E-03 | 3.70E-02 |
| Plk3 | PLK3 | 0.94 | 8.96E-04 | 1.91E-02 |
| Pik3ap1 | PIK3AP1 | 0.94 | 1.55E-03 | 2.84E-02 |
| Spire1 | SPIRE1 | 0.95 | 1.26E-03 | 2.48E-02 |
| Stk17b | STK17B | 0.95 | 2.49E-05 | 1.20E-03 |
| Cd300a | CD300C | 0.95 | 2.41E-03 | 3.82E-02 |
| Klrg2 | KLRG2 | 0.95 | 1.73E-03 | 3.02E-02 |
| Hcar2 | HCAR3 | 0.96 | 6.47E-05 | 2.59E-03 |
| Ctse | CTSE | 0.97 | 3.24E-03 | 4.63E-02 |
| Aldh1a3 | ALDH1A3 | 0.97 | 2.70E-03 | 4.15E-02 |
| Gja1 | GJA1 | 0.97 | 4.70E-05 | 2.04E-03 |
| Adam19 | ADAM19 | 0.97 | 2.94E-03 | 4.36E-02 |
| Gsta2 | GSTA2 | 0.98 | 2.31E-03 | 3.71E-02 |
| Tmem45b | TMEM45B | 0.98 | 1.48E-03 | 2.75E-02 |
| Ninj1 | NINJ1 | 0.98 | 2.66E-03 | 4.11E-02 |
| Ttll7 | TTLL7 | 0.98 | 8.35E-04 | 1.82E-02 |
| Dmxl2 | DMXL2 | 0.99 | 2.79E-03 | 4.21E-02 |
| Ubash3b | UBASH3B | 0.99 | 2.17E-03 | 3.54E-02 |
| Krt20 | KRT20 | 0.99 | 1.90E-03 | 3.22E-02 |
| Bglap3 | BGLAP | 1.00 | 8.79E-05 | 3.25E-03 |
| Lrp8 | LRP8 | 1.00 | 6.94E-04 | 1.61E-02 |
| Pilra | PILRB | 1.00 | 2.62E-03 | 4.07E-02 |
| Sp6 | SP6 | 1.00 | 4.77E-04 | 1.22E-02 |
| Wipf1 | WIPF1 | 1.01 | 1.16E-05 | 6.72E-04 |
| Msn | MSN | 1.01 | 6.55E-07 | 7.33E-05 |
| Tmem158 | TMEM158 | 1.02 | 2.32E-03 | 3.71E-02 |
| Fut4 | FUT4 | 1.02 | 8.41E-04 | 1.83E-02 |
| Gpsm3 | GPSM3 | 1.02 | 7.01E-04 | 1.62E-02 |
| Flrt3 | FLRT3 | 1.02 | 1.68E-03 | 2.96E-02 |
| Ctgf | CTGF | 1.03 | 1.12E-03 | 2.28E-02 |
| Myo1f | MYO1F | 1.03 | 7.46E-04 | 1.69E-02 |
| Rac2 | RAC2 | 1.04 | 2.68E-04 | 7.76E-03 |
| Plau | PLAU | 1.04 | 3.48E-04 | 9.56E-03 |
| Pfkfb3 | PFKFB3 | 1.04 | 3.18E-06 | 2.47E-04 |
| Itgb2 | ITGB2 | 1.04 | 2.67E-03 | 4.12E-02 |
| Tm4sf1 | TM4SF1 | 1.04 | 2.24E-03 | 3.64E-02 |
| Hsd17b7 | HSD17B7 | 1.05 | 4.71E-04 | 1.20E-02 |
| Cyth4 | CYTH4 | 1.05 | 1.03E-04 | 3.65E-03 |
| Sncg | SNCG | 1.05 | 2.00E-04 | 6.17E-03 |
| Ereg | EREG | 1.06 | 8.52E-05 | 3.19E-03 |
| Sh2d3c | SH2D3C | 1.06 | 3.61E-03 | 4.96E-02 |
| Neurl3 | NEURL3 | 1.07 | 1.91E-03 | 3.23E-02 |
| Cpt1c | CPT1C | 1.08 | 2.56E-04 | 7.51E-03 |
| Has3 | HAS3 | 1.08 | 1.84E-04 | 5.77E-03 |
| Itga2 | ITGA2 | 1.08 | 7.63E-04 | 1.70E-02 |
| Serpine1 | SERPINE1 | 1.09 | 1.03E-03 | 2.13E-02 |
| Sult2b1 | SULT2B1 | 1.09 | 1.82E-03 | 3.13E-02 |
| Nqo1 | NQO1 | 1.09 | 2.66E-06 | 2.12E-04 |
| Il1rn | IL1RN | 1.09 | 5.33E-05 | 2.27E-03 |
| Psrc1 | PSRC1 | 1.09 | 2.95E-04 | 8.44E-03 |
| Il4ra | IL4R | 1.09 | 1.06E-07 | 1.72E-05 |
| Vav1 | VAV1 | 1.09 | 6.08E-05 | 2.50E-03 |
| Ccl9 | CCL23 | 1.10 | 8.86E-04 | 1.90E-02 |
| Cdkn1a | CDKN1A | 1.10 | 8.87E-07 | 9.26E-05 |
| Arhgap30 | ARHGAP30 | 1.11 | 7.49E-04 | 1.69E-02 |
| Cyfip2 | CYFIP2 | 1.11 | 2.62E-05 | 1.26E-03 |
| Ncf1 | NCF1 | 1.11 | 3.48E-04 | 9.56E-03 |
| Gjb6 | GJB6 | 1.12 | 1.62E-03 | 2.90E-02 |
| Ctsl | CTSV | 1.13 | 1.02E-03 | 2.12E-02 |
| Hmox1 | HMOX1 | 1.13 | 4.40E-04 | 1.14E-02 |
| Serpine2 | SERPINE2 | 1.14 | 5.05E-08 | 9.35E-06 |
| Fst | FST | 1.14 | 2.04E-05 | 1.02E-03 |
| Rps6ka2 | RPS6KA2 | 1.14 | 5.77E-06 | 4.04E-04 |
| Fas | FAS | 1.14 | 8.72E-04 | 1.88E-02 |
| Wdr62 | WDR62 | 1.14 | 1.28E-03 | 2.49E-02 |
| Cst6 | CST6 | 1.14 | 2.01E-03 | 3.36E-02 |
| Ugcg | UGCG | 1.14 | 7.75E-05 | 2.99E-03 |
| Nfam1 | NFAM1 | 1.14 | 6.95E-04 | 1.61E-02 |
| Cd300lb | CD300LB | 1.15 | 1.51E-03 | 2.78E-02 |
| Pi16 | PI16 | 1.15 | 1.13E-03 | 2.30E-02 |
| Pinlyp | PINLYP | 1.15 | 3.91E-04 | 1.04E-02 |
| Coro1a | CORO1A | 1.16 | 1.46E-04 | 4.82E-03 |
| Pygl | PYGL | 1.16 | 3.49E-04 | 9.56E-03 |
| Fn1 | FN1 | 1.16 | 2.96E-03 | 4.37E-02 |
| Procr | PROCR | 1.16 | 4.36E-06 | 3.16E-04 |
| Cpne2 | CPNE2 | 1.17 | 2.78E-05 | 1.32E-03 |
| Csf2rb | CSF2RB | 1.17 | 3.85E-04 | 1.03E-02 |
| Syk | SYK | 1.17 | 2.37E-06 | 1.94E-04 |
| Gpr115 | NA | 1.18 | 1.06E-03 | 2.18E-02 |
| Glb1l2 | GLB1L2 | 1.18 | 8.93E-05 | 3.28E-03 |
| Tnip3 | TNIP3 | 1.18 | 4.25E-07 | 5.05E-05 |
| Gtse1 | GTSE1 | 1.19 | 5.84E-05 | 2.43E-03 |
| Tnfrsf1b | TNFRSF1B | 1.19 | 1.44E-06 | 1.37E-04 |
| Fbxl2 | FBXL2 | 1.20 | 3.04E-03 | 4.44E-02 |
| Lgals3bp | LGALS3BP | 1.20 | 2.97E-03 | 4.37E-02 |
| Rgcc | RGCC | 1.21 | 8.71E-05 | 3.24E-03 |
| Gpr35 | GPR35 | 1.21 | 1.46E-04 | 4.82E-03 |
| Cfp | CFP | 1.22 | 1.01E-04 | 3.62E-03 |
| Dok2 | DOK2 | 1.22 | 2.36E-03 | 3.76E-02 |
| Egr3 | EGR3 | 1.22 | 1.89E-03 | 3.21E-02 |
| Selplg | SELPLG | 1.22 | 3.13E-06 | 2.45E-04 |
| Themis2 | THEMIS2 | 1.22 | 1.23E-04 | 4.25E-03 |
| Il34 | IL34 | 1.22 | 4.79E-05 | 2.07E-03 |
| Chit1 | CHIT1 | 1.22 | 3.86E-04 | 1.03E-02 |
| Nipal4 | NIPAL4 | 1.23 | 1.24E-03 | 2.45E-02 |
| Gjb2 | GJB2 | 1.23 | 3.43E-04 | 9.51E-03 |
| Tnfrsf9 | TNFRSF9 | 1.23 | 2.88E-03 | 4.31E-02 |
| Fam83a | FAM83A | 1.23 | 1.58E-03 | 2.86E-02 |
| Ptprc | PTPRC | 1.23 | 3.45E-06 | 2.63E-04 |
| Gcnt3 | GCNT3 | 1.24 | 2.14E-03 | 3.50E-02 |
| Rdh12 | RDH12 | 1.24 | 1.32E-05 | 7.45E-04 |
| Clec2f | NA | 1.24 | 1.65E-03 | 2.94E-02 |
| Napsa | NAPSA | 1.24 | 2.04E-04 | 6.23E-03 |
| Ms4a6d | MS4A6A | 1.24 | 5.16E-04 | 1.28E-02 |
| Arhgap9 | ARHGAP9 | 1.24 | 7.24E-05 | 2.85E-03 |
| Spi1 | SPI1 | 1.25 | 6.24E-05 | 2.53E-03 |
| Otop3 | OTOP3 | 1.25 | 5.28E-04 | 1.30E-02 |
| Plcd4 | PLCD4 | 1.25 | 1.34E-03 | 2.58E-02 |
| Rbp2 | RBP2 | 1.25 | 6.67E-04 | 1.57E-02 |
| Tyrobp | TYROBP | 1.25 | 7.40E-06 | 4.83E-04 |
| Cass4 | CASS4 | 1.26 | 3.67E-04 | 9.94E-03 |
| Fhad1 | FHAD1 | 1.26 | 2.01E-03 | 3.36E-02 |
| Ltb4r1 | LTB4R | 1.27 | 1.77E-05 | 9.24E-04 |
| Ccl6 | CCL15 | 1.27 | 8.19E-06 | 5.20E-04 |
| Elovl4 | ELOVL4 | 1.28 | 7.89E-04 | 1.74E-02 |
| Mcam | MCAM | 1.28 | 9.15E-07 | 9.49E-05 |
| Ttc34 | TTC34 | 1.28 | 1.34E-03 | 2.58E-02 |
| Slc28a2 | SLC28A2 | 1.28 | 3.60E-03 | 4.96E-02 |
| Naip5 | NAIP | 1.28 | 7.66E-04 | 1.70E-02 |
| Flt3l | FLT3LG | 1.28 | 1.88E-03 | 3.20E-02 |
| Lipg | LIPG | 1.28 | 1.13E-03 | 2.29E-02 |
| Wnt11 | WNT11 | 1.28 | 1.60E-05 | 8.57E-04 |
| Rgs14 | RGS14 | 1.29 | 4.08E-04 | 1.08E-02 |
| Plaur | PLAUR | 1.29 | 8.44E-05 | 3.18E-03 |
| Slc16a6 | SLC16A6 | 1.30 | 1.26E-04 | 4.31E-03 |
| Fkbp5 | FKBP5 | 1.30 | 5.79E-07 | 6.68E-05 |
| Card9 | CARD9 | 1.30 | 1.09E-03 | 2.24E-02 |
| Snx20 | SNX20 | 1.30 | 1.16E-04 | 4.02E-03 |
| Cyp26b1 | CYP26B1 | 1.30 | 2.15E-04 | 6.49E-03 |
| Arid3a | ARID3A | 1.30 | 6.15E-05 | 2.52E-03 |
| Ncf2 | NCF2 | 1.31 | 3.24E-07 | 4.05E-05 |
| F10 | F10 | 1.31 | 1.13E-03 | 2.30E-02 |
| Klhl6 | KLHL6 | 1.32 | 1.69E-03 | 2.97E-02 |
| 2010109I03Rik | NA | 1.32 | 3.63E-03 | 4.98E-02 |
| Bpifc | BPIFC | 1.32 | 2.57E-03 | 4.01E-02 |
| Chst11 | CHST11 | 1.32 | 1.51E-07 | 2.22E-05 |
| Rasgrp4 | RASGRP4 | 1.33 | 2.36E-03 | 3.76E-02 |
| Fmnl1 | FMNL1 | 1.33 | 4.06E-06 | 2.99E-04 |
| Ccdc147 | NA | 1.35 | 2.82E-03 | 4.24E-02 |
| Ankrd37 | ANKRD37 | 1.35 | 1.57E-04 | 5.05E-03 |
| Ccrl2 | CCRL2 | 1.36 | 8.01E-05 | 3.08E-03 |
| Ttc21a | TTC21A | 1.37 | 2.94E-03 | 4.36E-02 |
| Trim15 | TRIM15 | 1.37 | 7.74E-08 | 1.32E-05 |
| Gm19557 | NA | 1.38 | 1.39E-04 | 4.66E-03 |
| Mafb | MAFB | 1.40 | 1.12E-07 | 1.79E-05 |
| Elmod1 | ELMOD1 | 1.40 | 1.16E-05 | 6.72E-04 |
| Fam65b | FAM65B | 1.40 | 3.49E-04 | 9.56E-03 |
| Ncf4 | NCF4 | 1.42 | 4.19E-07 | 5.01E-05 |
| Ccdc11 | NA | 1.42 | 2.90E-03 | 4.33E-02 |
| Gdf15 | GDF15 | 1.42 | 2.99E-04 | 8.51E-03 |
| Col27a1 | COL27A1 | 1.42 | 2.07E-03 | 3.43E-02 |
| Fxyd5 | FXYD5 | 1.43 | 1.28E-08 | 2.96E-06 |
| Lrrc25 | LRRC25 | 1.43 | 3.41E-03 | 4.77E-02 |
| Lcp2 | LCP2 | 1.44 | 1.96E-06 | 1.67E-04 |
| AB124611 | C19orf38 | 1.44 | 1.88E-05 | 9.52E-04 |
| Cd84 | CD84 | 1.44 | 5.70E-05 | 2.39E-03 |
| Ugt1a7c | NA | 1.44 | 3.89E-06 | 2.90E-04 |
| Slc7a11 | SLC7A11 | 1.44 | 3.30E-09 | 1.03E-06 |
| Basp1 | BASP1 | 1.45 | 1.52E-05 | 8.30E-04 |
| Hpgds | HPGDS | 1.45 | 1.27E-05 | 7.22E-04 |
| Cyp1b1 | CYP1B1 | 1.45 | 9.00E-04 | 1.92E-02 |
| 2610528A11Rik | C10orf99 | 1.46 | 4.73E-08 | 8.87E-06 |
| Plek | PLEK | 1.46 | 4.39E-07 | 5.18E-05 |
| Ly6c1 | NA | 1.46 | 2.02E-05 | 1.01E-03 |
| Dok3 | DOK3 | 1.47 | 4.70E-05 | 2.04E-03 |
| Glipr1 | GLIPR1 | 1.47 | 7.47E-07 | 8.07E-05 |
| Lrrc36 | LRRC36 | 1.48 | 1.53E-03 | 2.81E-02 |
| Clec4a2 | CLEC4A | 1.48 | 1.48E-04 | 4.86E-03 |
| Fabp5 | FABP5 | 1.49 | 7.57E-05 | 2.94E-03 |
| Hal | HAL | 1.49 | 1.87E-05 | 9.52E-04 |
| Relt | RELT | 1.49 | 2.26E-03 | 3.66E-02 |
| Slc4a11 | SLC4A11 | 1.49 | 1.56E-06 | 1.44E-04 |
| Nr1h5 | NA | 1.49 | 2.78E-03 | 4.21E-02 |
| Pirb | LILRA1 | 1.50 | 1.60E-05 | 8.57E-04 |
| Ldlrad1 | LDLRAD1 | 1.50 | 3.17E-03 | 4.56E-02 |
| Sh2b2 | SH2B2 | 1.51 | 3.59E-05 | 1.63E-03 |
| 4930539E08Rik | C6orf222 | 1.51 | 5.39E-05 | 2.28E-03 |
| Rnf183 | RNF183 | 1.51 | 3.28E-03 | 4.66E-02 |
| Rhof | RHOF | 1.52 | 2.67E-04 | 7.72E-03 |
| Slc15a3 | SLC15A3 | 1.52 | 3.31E-07 | 4.09E-05 |
| Efcab5 | EFCAB5 | 1.52 | 1.28E-03 | 2.49E-02 |
| Ces2e | CES2 | 1.53 | 6.26E-11 | 3.61E-08 |
| Lrrc10b | LRRC10B | 1.53 | 1.67E-03 | 2.95E-02 |
| Fam167a | FAM167A | 1.53 | 2.85E-08 | 5.68E-06 |
| Slfn4 | SLFN12 | 1.54 | 5.54E-04 | 1.35E-02 |
| Gas2l2 | GAS2L2 | 1.54 | 1.47E-03 | 2.74E-02 |
| Dpep2 | DPEP2 | 1.55 | 1.74E-06 | 1.56E-04 |
| Sgpp2 | SGPP2 | 1.56 | 5.22E-04 | 1.29E-02 |
| Klk12 | KLK12 | 1.56 | 2.78E-03 | 4.21E-02 |
| Nek5 | NEK5 | 1.56 | 2.21E-03 | 3.60E-02 |
| Slc7a8 | SLC7A8 | 1.57 | 1.87E-05 | 9.52E-04 |
| Cd101 | CD101 | 1.58 | 1.60E-04 | 5.11E-03 |
| Klk13 | KLK13 | 1.58 | 8.35E-04 | 1.82E-02 |
| Cyp2c65 | CYP2C9 | 1.58 | 8.73E-05 | 3.24E-03 |
| Siglece | SIGLEC9 | 1.59 | 9.89E-06 | 6.03E-04 |
| Fcer1g | FCER1G | 1.59 | 2.21E-07 | 2.96E-05 |
| Cidea | CIDEA | 1.59 | 4.96E-11 | 2.97E-08 |
| Nfkbid | NFKBID | 1.59 | 3.90E-05 | 1.75E-03 |
| D130043K22Rik | KIAA0319 | 1.60 | 2.48E-03 | 3.91E-02 |
| Tmprss11d | TMPRSS11D | 1.61 | 3.21E-05 | 1.48E-03 |
| Speer4e | NA | 1.61 | 9.05E-04 | 1.92E-02 |
| Ccdc37 | CCDC37 | 1.61 | 8.21E-04 | 1.80E-02 |
| Snai1 | SNAI1 | 1.61 | 1.13E-03 | 2.30E-02 |
| Pram1 | PRAM1 | 1.62 | 6.33E-04 | 1.51E-02 |
| Gm10639 | GSTA2 | 1.62 | 7.47E-04 | 1.69E-02 |
| Fgr | FGR | 1.63 | 5.65E-08 | 1.03E-05 |
| Fcgr1 | FCGR1B | 1.63 | 1.38E-03 | 2.63E-02 |
| 4632434I11Rik | NA | 1.64 | 2.08E-06 | 1.76E-04 |
| Pf4 | PF4V1 | 1.64 | 1.91E-04 | 5.95E-03 |
| Rnd1 | RND1 | 1.64 | 1.01E-03 | 2.10E-02 |
| Cd33 | CD33 | 1.64 | 6.19E-07 | 7.08E-05 |
| Cd53 | CD53 | 1.65 | 7.28E-10 | 2.98E-07 |
| Lilrb4 | LILRB3 | 1.65 | 4.07E-07 | 4.93E-05 |
| Mcemp1 | MCEMP1 | 1.65 | 6.34E-05 | 2.56E-03 |
| B430212C06Rik | NA | 1.65 | 1.16E-03 | 2.34E-02 |
| Gm5547 | NA | 1.65 | 1.18E-03 | 2.37E-02 |
| Gpr84 | GPR84 | 1.65 | 2.78E-03 | 4.21E-02 |
| Ptafr | PTAFR | 1.66 | 9.69E-09 | 2.39E-06 |
| Gm17019 | NA | 1.66 | 3.25E-03 | 4.64E-02 |
| Hcst | HCST | 1.67 | 2.56E-03 | 4.00E-02 |
| Hk3 | HK3 | 1.70 | 4.39E-05 | 1.94E-03 |
| Ipcef1 | IPCEF1 | 1.70 | 8.15E-06 | 5.20E-04 |
| Ctla4 | CTLA4 | 1.71 | 1.89E-03 | 3.21E-02 |
| Gtsf1l | GTSF1L | 1.72 | 2.44E-03 | 3.86E-02 |
| Gm5150 | SIRPB2 | 1.72 | 6.69E-06 | 4.48E-04 |
| Fcgr3 | FCGR2B | 1.73 | 4.11E-09 | 1.21E-06 |
| Clec4a1 | CLEC4A | 1.73 | 3.28E-03 | 4.66E-02 |
| Gpnmb | GPNMB | 1.74 | 1.20E-03 | 2.39E-02 |
| Cd300ld | CD300LF | 1.74 | 1.02E-09 | 4.07E-07 |
| Slfn1 | SLFN12L | 1.75 | 4.61E-08 | 8.84E-06 |
| Sirpb1b | SIRPA | 1.75 | 1.47E-03 | 2.74E-02 |
| Ush1g | USH1G | 1.76 | 1.94E-04 | 6.02E-03 |
| Fcgr2b | FCGR2B | 1.77 | 8.70E-08 | 1.44E-05 |
| Sema6b | SEMA6B | 1.77 | 3.20E-06 | 2.47E-04 |
| Msr1 | MSR1 | 1.77 | 4.13E-05 | 1.84E-03 |
| Tmem212 | TMEM212 | 1.77 | 1.44E-04 | 4.80E-03 |
| Aqp9 | AQP9 | 1.78 | 1.10E-05 | 6.47E-04 |
| Mmp9 | MMP9 | 1.79 | 8.17E-06 | 5.20E-04 |
| Npl | NPL | 1.79 | 3.08E-05 | 1.43E-03 |
| Gsta1 | GSTA1 | 1.79 | 1.33E-05 | 7.49E-04 |
| Il1f6 | IL36A | 1.79 | 3.22E-05 | 1.48E-03 |
| Klra2 | NA | 1.79 | 1.08E-05 | 6.39E-04 |
| Trem2 | TREM2 | 1.79 | 1.56E-05 | 8.47E-04 |
| Il1f9 | IL36G | 1.79 | 8.32E-06 | 5.22E-04 |
| Slc11a1 | SLC11A1 | 1.80 | 2.12E-08 | 4.40E-06 |
| Pla2g7 | PLA2G7 | 1.80 | 1.56E-10 | 8.10E-08 |
| Stx11 | STX11 | 1.81 | 3.18E-09 | 1.01E-06 |
| Il23a | IL23A | 1.81 | 1.88E-03 | 3.20E-02 |
| Ccr1 | CCR1 | 1.82 | 3.69E-13 | 5.75E-10 |
| Endou | ENDOU | 1.82 | 2.40E-06 | 1.94E-04 |
| Mab21l3 | MAB21L3 | 1.83 | 1.75E-06 | 1.56E-04 |
| Sirpb1a | SIRPG | 1.83 | 5.37E-05 | 2.27E-03 |
| Cdhr3 | CDHR3 | 1.84 | 6.43E-05 | 2.59E-03 |
| Nat8l | NAT8L | 1.84 | 6.15E-04 | 1.47E-02 |
| Emilin2 | EMILIN2 | 1.84 | 2.05E-09 | 7.23E-07 |
| AU040972 | NA | 1.86 | 5.76E-05 | 2.41E-03 |
| Ccl4 | CCL4 | 1.86 | 3.65E-05 | 1.65E-03 |
| Arg2 | ARG2 | 1.86 | 4.48E-04 | 1.16E-02 |
| Tdh | NA | 1.86 | 7.58E-04 | 1.70E-02 |
| Alox5ap | ALOX5AP | 1.87 | 3.26E-07 | 4.05E-05 |
| Mctp1 | MCTP1 | 1.88 | 1.69E-05 | 9.02E-04 |
| Thbs2 | THBS2 | 1.88 | 2.01E-04 | 6.19E-03 |
| Il18rap | IL18RAP | 1.88 | 3.77E-10 | 1.64E-07 |
| Cd300lf | CD300LF | 1.88 | 1.16E-09 | 4.50E-07 |
| Gsdmc | GSDMC | 1.89 | 3.05E-05 | 1.42E-03 |
| Serpinb3b | SERPINB3 | 1.90 | 6.17E-04 | 1.47E-02 |
| Gp49a | LILRB3 | 1.90 | 1.36E-07 | 2.06E-05 |
| Chia1 | CHIA | 1.90 | 3.77E-04 | 1.01E-02 |
| Socs3 | SOCS3 | 1.91 | 1.63E-09 | 6.00E-07 |
| Mansc4 | MANSC4 | 1.91 | 1.04E-04 | 3.70E-03 |
| Amer2 | AMER2 | 1.92 | 3.15E-03 | 4.54E-02 |
| Slc16a3 | SLC16A3 | 1.92 | 1.79E-07 | 2.52E-05 |
| Prss12 | PRSS12 | 1.93 | 1.87E-08 | 3.95E-06 |
| C3ar1 | C3AR1 | 1.93 | 6.13E-06 | 4.18E-04 |
| 2210407C18Rik | NA | 1.93 | 1.05E-06 | 1.06E-04 |
| Mefv | MEFV | 1.93 | 1.48E-05 | 8.16E-04 |
| Igsf6 | IGSF6 | 1.94 | 1.37E-08 | 3.13E-06 |
| Pira2 | LILRB2 | 1.95 | 2.68E-03 | 4.12E-02 |
| Fndc7 | FNDC7 | 1.95 | 2.18E-06 | 1.80E-04 |
| Erich3 | ERICH3 | 1.96 | 3.17E-05 | 1.47E-03 |
| Bst1 | BST1 | 1.96 | 6.07E-06 | 4.17E-04 |
| Fcrlb | FCRLB | 1.97 | 1.50E-03 | 2.78E-02 |
| Trim58 | TRIM58 | 1.98 | 1.76E-03 | 3.04E-02 |
| Mmp13 | MMP13 | 1.98 | 5.55E-05 | 2.33E-03 |
| Rab44 | RAB44 | 1.99 | 2.41E-06 | 1.95E-04 |
| Lilra6 | LILRB2 | 2.01 | 1.31E-07 | 1.99E-05 |
| Atp1a3 | ATP1A3 | 2.02 | 2.40E-07 | 3.13E-05 |
| Slc2a3 | SLC2A14 | 2.04 | 2.11E-06 | 1.77E-04 |
| Mgam | MGAM | 2.04 | 1.43E-06 | 1.37E-04 |
| Srgn | SRGN | 2.06 | 1.66E-09 | 6.00E-07 |
| Irg1 | IRG1 | 2.07 | 2.77E-11 | 1.90E-08 |
| Tnfsf9 | TNFSF9 | 2.08 | 7.54E-05 | 2.94E-03 |
| Kng2 | KNG1 | 2.09 | 6.00E-04 | 1.44E-02 |
| Cxcr2 | CXCR2 | 2.10 | 8.51E-17 | 6.62E-13 |
| Degs2 | DEGS2 | 2.10 | 2.42E-04 | 7.20E-03 |
| Tnfrsf26 | NA | 2.11 | 1.81E-07 | 2.53E-05 |
| Gm3776 | NA | 2.11 | 1.94E-07 | 2.70E-05 |
| Gcnt4 | GCNT4 | 2.11 | 3.41E-03 | 4.77E-02 |
| Slc38a11 | SLC38A11 | 2.11 | 1.07E-03 | 2.21E-02 |
| Clec4e | CLEC4E | 2.12 | 1.28E-07 | 1.98E-05 |
| Tnfsf14 | TNFSF14 | 2.12 | 1.36E-05 | 7.60E-04 |
| Timp1 | TIMP1 | 2.13 | 1.47E-08 | 3.19E-06 |
| Nkx2-9 | NKX2-8 | 2.15 | 1.05E-05 | 6.33E-04 |
| Gpr97 | NA | 2.15 | 9.78E-07 | 1.00E-04 |
| Ccl3 | CCL3L3 | 2.16 | 9.86E-05 | 3.55E-03 |
| Sell | SELL | 2.16 | 4.72E-10 | 1.98E-07 |
| Chil4 | CHIA | 2.20 | 1.87E-06 | 1.61E-04 |
| Celsr3 | CELSR3 | 2.21 | 3.69E-04 | 9.97E-03 |
| Cdh26 | CDH26 | 2.22 | 2.38E-06 | 1.94E-04 |
| B430306N03Rik | NA | 2.22 | 5.63E-06 | 3.96E-04 |
| Il1b | IL1B | 2.23 | 6.15E-08 | 1.10E-05 |
| Bend4 | BEND4 | 2.23 | 4.09E-07 | 4.93E-05 |
| Csf3r | CSF3R | 2.24 | 3.65E-10 | 1.64E-07 |
| Samsn1 | SAMSN1 | 2.24 | 3.18E-09 | 1.01E-06 |
| Krt6b | KRT6A | 2.25 | 9.02E-04 | 1.92E-02 |
| Tarm1 | TARM1 | 2.25 | 1.14E-07 | 1.81E-05 |
| Tlr13 | NA | 2.28 | 1.74E-16 | 7.04E-13 |
| Adam8 | ADAM8 | 2.28 | 3.02E-15 | 9.39E-12 |
| Otop2 | OTOP2 | 2.28 | 8.35E-05 | 3.16E-03 |
| Gm10872 | NA | 2.28 | 4.72E-05 | 2.05E-03 |
| C5ar2 | C5AR2 | 2.30 | 3.85E-06 | 2.90E-04 |
| Gm19705 | NA | 2.30 | 1.19E-03 | 2.37E-02 |
| Crxos | NA | 2.32 | 3.55E-03 | 4.90E-02 |
| C5ar1 | C5AR1 | 2.35 | 1.81E-16 | 7.04E-13 |
| Gm4832 | NA | 2.35 | 1.10E-03 | 2.25E-02 |
| Gm1123 | CXADR | 2.36 | 1.46E-03 | 2.74E-02 |
| Lrg1 | LRG1 | 2.36 | 6.43E-08 | 1.14E-05 |
| U90926 | NA | 2.38 | 1.24E-03 | 2.45E-02 |
| Mmp8 | MMP8 | 2.40 | 3.72E-04 | 1.00E-02 |
| Cdhr1 | CDHR1 | 2.42 | 1.25E-06 | 1.23E-04 |
| 1100001G20Rik | NA | 2.42 | 3.86E-06 | 2.90E-04 |
| Stfa3 | CSTA | 2.44 | 1.96E-05 | 9.84E-04 |
| Ifltd1 | NA | 2.45 | 2.67E-03 | 4.11E-02 |
| Apoc2 | APOC4-APOC2 | 2.46 | 2.69E-06 | 2.14E-04 |
| Gal3st2 | GAL3ST2 | 2.46 | 2.90E-03 | 4.33E-02 |
| Slc6a12 | SLC6A12 | 2.46 | 1.44E-06 | 1.37E-04 |
| Trem3 | NA | 2.46 | 1.29E-03 | 2.50E-02 |
| Selp | SELP | 2.49 | 3.08E-06 | 2.42E-04 |
| Nlrp12 | NLRP12 | 2.50 | 3.04E-05 | 1.42E-03 |
| Mrgpra2b | MRGPRX2 | 2.52 | 1.23E-03 | 2.43E-02 |
| Tubb3 | TUBB3 | 2.53 | 1.46E-07 | 2.17E-05 |
| Cxcl5 | CXCL6 | 2.53 | 9.01E-08 | 1.48E-05 |
| Sprr1b | NA | 2.53 | 2.77E-03 | 4.21E-02 |
| Fpr2 | FPR2 | 2.54 | 1.47E-11 | 1.27E-08 |
| 9830107B12Rik | NA | 2.54 | 5.24E-04 | 1.30E-02 |
| Psors1c2 | PSORS1C2 | 2.58 | 1.59E-05 | 8.57E-04 |
| Chst5 | CHST6 | 2.58 | 7.42E-06 | 4.83E-04 |
| Sprr2j-ps | NA | 2.59 | 2.02E-07 | 2.78E-05 |
| A530064D06Rik | NA | 2.63 | 1.79E-06 | 1.57E-04 |
| Nlrp3 | NLRP3 | 2.68 | 3.83E-09 | 1.17E-06 |
| Clec4d | CLEC4D | 2.68 | 2.60E-11 | 1.90E-08 |
| 4933432I03Rik | NA | 2.68 | 6.85E-04 | 1.59E-02 |
| Cyp4f18 | CYP4F3 | 2.73 | 2.50E-12 | 2.59E-09 |
| Serpinb3c | SERPINB3 | 2.75 | 6.25E-05 | 2.53E-03 |
| Trem1 | TREM1 | 2.77 | 4.07E-09 | 1.21E-06 |
| Ccl2 | CCL2 | 2.77 | 1.39E-12 | 1.96E-09 |
| Wfdc17 | NA | 2.81 | 4.48E-09 | 1.27E-06 |
| Bpifa5 | BPIFA1 | 2.84 | 7.98E-07 | 8.50E-05 |
| Csf3 | CSF3 | 2.86 | 3.17E-10 | 1.54E-07 |
| Cxcl2 | CXCL2 | 2.87 | 1.56E-06 | 1.44E-04 |
| Krt84 | KRT84 | 2.88 | 1.74E-03 | 3.03E-02 |
| Chil1 | CHI3L1 | 2.89 | 3.86E-11 | 2.50E-08 |
| Krt16 | KRT16 | 2.90 | 6.22E-05 | 2.53E-03 |
| Prok2 | PROK2 | 2.92 | 1.96E-05 | 9.85E-04 |
| Tmem252 | TMEM252 | 2.92 | 5.15E-05 | 2.19E-03 |
| Sprr2k | NA | 2.92 | 4.30E-04 | 1.12E-02 |
| Npy | NPY | 2.94 | 1.81E-05 | 9.35E-04 |
| Cxcl3 | CXCL2 | 2.94 | 3.47E-12 | 3.37E-09 |
| F630028O10Rik | NA | 2.95 | 5.84E-08 | 1.06E-05 |
| Nfe2 | NFE2 | 3.01 | 1.56E-10 | 8.10E-08 |
| BC100530 | CSTA | 3.03 | 8.29E-07 | 8.77E-05 |
| Lipn | LIPN | 3.04 | 2.39E-05 | 1.16E-03 |
| Msx3 | MSX2 | 3.05 | 1.08E-03 | 2.21E-02 |
| Cd177 | CD177 | 3.06 | 2.04E-07 | 2.78E-05 |
| Creb3l3 | CREB3L3 | 3.09 | 2.70E-03 | 4.15E-02 |
| Sprr2g | NA | 3.09 | 3.88E-06 | 2.90E-04 |
| Lin28a | LIN28A | 3.10 | 1.59E-03 | 2.88E-02 |
| S100a8 | S100A8 | 3.15 | 2.30E-09 | 7.77E-07 |
| Trim30b | NA | 3.15 | 1.03E-08 | 2.50E-06 |
| BC049730 | CD177 | 3.22 | 2.22E-14 | 4.94E-11 |
| Ppbp | PPBP | 3.24 | 1.55E-07 | 2.26E-05 |
| Prap1 | PRAP1 | 3.25 | 2.45E-03 | 3.87E-02 |
| Sh2d5 | SH2D5 | 3.26 | 6.66E-06 | 4.48E-04 |
| Rnase2b | RNASE2 | 3.28 | 4.83E-09 | 1.34E-06 |
| Il19 | IL19 | 3.30 | 7.48E-04 | 1.69E-02 |
| Ccl7 | CCL7 | 3.31 | 2.27E-12 | 2.52E-09 |
| Retnlg | RETNLB | 3.32 | 1.81E-06 | 1.58E-04 |
| Mmp10 | MMP10 | 3.35 | 1.65E-04 | 5.25E-03 |
| Sprr2f | NA | 3.39 | 4.37E-06 | 3.16E-04 |
| Cd5l | CD5L | 3.46 | 2.15E-04 | 6.49E-03 |
| Klk6 | KLK6 | 3.53 | 3.71E-04 | 1.00E-02 |
| Ifitm6 | IFITM3 | 3.58 | 5.70E-09 | 1.54E-06 |
| Sprr2h | NA | 3.59 | 3.64E-05 | 1.64E-03 |
| S100a9 | S100A9 | 3.73 | 4.42E-11 | 2.75E-08 |
| Ambp | AMBP | 3.76 | 1.16E-05 | 6.72E-04 |
| Igll1 | IGLL5 | 3.80 | 6.55E-05 | 2.60E-03 |
| Gm5483 | CSTA | 3.94 | 2.24E-12 | 2.52E-09 |
| Sprr2i | NA | 4.12 | 1.24E-06 | 1.22E-04 |
| Htr2b | HTR2B | 4.31 | 2.07E-07 | 2.80E-05 |
| Ngp | NA | 4.34 | 9.23E-05 | 3.34E-03 |
| Gm5416 | CSTA | 4.36 | 8.95E-09 | 2.27E-06 |
| Saa3 | NA | 4.48 | 5.05E-07 | 5.90E-05 |
| Akr1d1 | AKR1D1 | 4.60 | 8.82E-06 | 5.46E-04 |
| Stfa2l1 | CSTA | 4.64 | 6.69E-14 | 1.30E-10 |
| Stfa2 | CSTA | 4.77 | 3.80E-10 | 1.64E-07 |
| Defb3 | NA | 5.04 | 1.53E-13 | 2.65E-10 |
| Sprr2b | NA | 5.74 | 6.00E-12 | 5.49E-09 |
| Sprr2e | NA | 6.04 | 1.73E-21 | 2.69E-17 |
| Sprr2d | NA | 6.36 | 1.88E-08 | 3.95E-06 |
