# Supplementary material for: Bronchial airway gene expression signatures in mouse lung squamous cell carcinoma and their modulation by cancer chemopreventive agents
Source: Oncotarget. 2016 Dec 7;8(12):18885–900. doi: 10.18632/oncotarget.13806 (PMC5386655; doi:10.18632/oncotarget.13806)
Supplement: Supplementary file 3 [file oncotarget-08-18885-s003.docx]

Table S3. Detailed information of the 3,018 differential expressed genes caused by pioglitazone treatment, which consisted of 1,491 significantly down-regulated genes and 1,527 significantly up-regulated genes after pioglitazone treatment. logFC (i.e., log2 Fold change in RNA-seq data comparing pioglitazone treated samples vs non-treated samples), P value and FDR (false discovery rate) were given for each gene. All the genes had FDR < 0.05 to be considered significant to adjust for multiple testing issue. MGIsymbol and HGNCsymbol mean the mouse gene name and corresponding human gene name. The mouse genes without corresponding human homolog genes were labeled ‘NA’ in the “HGNCsymbol” column.

| MGIsymbol | HGNCsymbol | logFC | P value | FDR |
| --- | --- | --- | --- | --- |
| Cdc20b | CDC20B | -8.53 | 1.19E-23 | 9.19E-20 |
| Myh1 | MYH1 | -6.87 | 5.66E-07 | 2.10E-05 |
| 4930444F02Rik | NA | -6.53 | 4.25E-07 | 1.68E-05 |
| 1190003K10Rik | NA | -6.48 | 1.49E-04 | 1.91E-03 |
| Plin1 | PLIN1 | -6.44 | 4.02E-07 | 1.61E-05 |
| Slc5a7 | SLC5A7 | -6.41 | 5.24E-06 | 1.24E-04 |
| Scgb1b3 | NA | -6.26 | 3.04E-06 | 8.11E-05 |
| Mcidas | MCIDAS | -6.19 | 1.66E-07 | 7.77E-06 |
| Foxn4 | FOXN4 | -6.13 | 3.34E-13 | 1.36E-10 |
| Zpbp | ZPBP | -5.96 | 5.01E-04 | 5.01E-03 |
| Ckmt2 | CKMT2 | -5.87 | 4.00E-08 | 2.40E-06 |
| Myh4 | MYH4 | -5.86 | 3.83E-08 | 2.34E-06 |
| Myl1 | MYL1 | -5.79 | 1.38E-06 | 4.31E-05 |
| Sln | SLN | -5.76 | 5.13E-08 | 2.91E-06 |
| Xirp2 | XIRP2 | -5.70 | 1.85E-07 | 8.44E-06 |
| Epha3 | EPHA3 | -5.64 | 1.24E-04 | 1.64E-03 |
| Myh13 | MYH13 | -5.56 | 1.50E-05 | 2.92E-04 |
| Tnnc2 | TNNC2 | -5.54 | 5.59E-06 | 1.30E-04 |
| 4930455B14Rik | NA | -5.53 | 1.06E-03 | 9.08E-03 |
| Car3 | CA3 | -5.53 | 2.21E-09 | 2.06E-07 |
| Trdn | TRDN | -5.53 | 3.20E-07 | 1.34E-05 |
| Fam196b | FAM196B | -5.53 | 1.14E-03 | 9.65E-03 |
| Kank4 | KANK4 | -5.51 | 4.98E-06 | 1.19E-04 |
| Neb | NEB | -5.48 | 5.46E-08 | 3.07E-06 |
| Chp2 | CHP2 | -5.48 | 1.27E-04 | 1.67E-03 |
| Fbxo43 | FBXO43 | -5.46 | 8.21E-04 | 7.45E-03 |
| Tmem132c | TMEM132C | -5.43 | 1.57E-06 | 4.78E-05 |
| Tusc5 | TUSC5 | -5.40 | 1.19E-05 | 2.40E-04 |
| Zfp454 | ZNF454 | -5.38 | 1.24E-04 | 1.64E-03 |
| Adipoq | ADIPOQ | -5.38 | 5.91E-07 | 2.16E-05 |
| Rgs13 | RGS13 | -5.33 | 1.31E-06 | 4.17E-05 |
| Tas2r108 | TAS2R4 | -5.30 | 2.24E-04 | 2.62E-03 |
| Gpihbp1 | GPIHBP1 | -5.27 | 3.76E-04 | 4.00E-03 |
| Eef1a2 | EEF1A2 | -5.26 | 2.11E-06 | 6.07E-05 |
| Ttn | TTN | -5.24 | 3.62E-11 | 6.68E-09 |
| Dcaf12l1 | DCAF12L2 | -5.21 | 1.17E-03 | 9.83E-03 |
| Dusp26 | DUSP26 | -5.19 | 1.87E-04 | 2.27E-03 |
| Prr32 | PRR32 | -5.17 | 3.47E-05 | 5.78E-04 |
| 6330403K07Rik | NA | -5.14 | 4.98E-06 | 1.19E-04 |
| Ankrd63 | ANKRD63 | -5.14 | 5.47E-04 | 5.35E-03 |
| Pvalb | PVALB | -5.09 | 3.54E-06 | 9.07E-05 |
| Tlr11 | NA | -5.09 | 8.70E-04 | 7.76E-03 |
| Myoz3 | MYOZ3 | -5.08 | 2.33E-11 | 4.61E-09 |
| Mybpc1 | MYBPC1 | -5.06 | 1.30E-06 | 4.15E-05 |
| AV039307 | NA | -5.02 | 3.31E-03 | 2.21E-02 |
| E130102H24Rik | NA | -4.99 | 2.11E-03 | 1.54E-02 |
| Cyp4f40 | CYP4F8 | -4.97 | 3.40E-04 | 3.68E-03 |
| Pde6c | PDE6C | -4.97 | 5.27E-03 | 3.14E-02 |
| Acta1 | ACTA1 | -4.95 | 2.34E-05 | 4.25E-04 |
| Scgb2b20 | NA | -4.95 | 3.69E-03 | 2.39E-02 |
| Cntd1 | CNTD1 | -4.94 | 2.34E-04 | 2.71E-03 |
| Asb14 | ASB14 | -4.93 | 1.62E-03 | 1.25E-02 |
| Zcchc18 | ZCCHC18 | -4.92 | 3.32E-03 | 2.21E-02 |
| Inmt | INMT | -4.90 | 5.47E-15 | 3.52E-12 |
| Dhrs7c | DHRS7C | -4.89 | 2.21E-04 | 2.59E-03 |
| Csrp3 | CSRP3 | -4.87 | 8.90E-07 | 3.04E-05 |
| Agtr2 | AGTR2 | -4.85 | 2.16E-05 | 3.97E-04 |
| Retn | RETN | -4.84 | 6.01E-05 | 9.18E-04 |
| Smpx | SMPX | -4.84 | 9.61E-07 | 3.22E-05 |
| Dcpp3 | NA | -4.79 | 1.66E-20 | 5.11E-17 |
| Lrrc39 | LRRC39 | -4.79 | 1.99E-06 | 5.77E-05 |
| Ube2u | UBE2U | -4.77 | 4.08E-03 | 2.59E-02 |
| Cyp2g1 | NA | -4.77 | 9.23E-03 | 4.81E-02 |
| 2310002L09Rik | NA | -4.76 | 7.45E-06 | 1.65E-04 |
| Pck1 | PCK1 | -4.75 | 1.34E-04 | 1.75E-03 |
| Retnla | RETNLB | -4.73 | 2.45E-11 | 4.74E-09 |
| Tdo2 | TDO2 | -4.72 | 2.32E-04 | 2.69E-03 |
| Ccno | CCNO | -4.72 | 1.20E-21 | 6.15E-18 |
| C1qtnf7 | C1QTNF7 | -4.69 | 7.17E-07 | 2.53E-05 |
| Phex | PHEX | -4.69 | 3.19E-08 | 2.02E-06 |
| Agbl1 | AGBL1 | -4.67 | 7.61E-04 | 7.00E-03 |
| A430093F15Rik | NA | -4.67 | 4.17E-03 | 2.63E-02 |
| Sh2d7 | NA | -4.65 | 3.53E-05 | 5.84E-04 |
| Abca6 | ABCA6 | -4.63 | 5.07E-03 | 3.05E-02 |
| Dner | DNER | -4.63 | 5.76E-08 | 3.19E-06 |
| Negr1 | NEGR1 | -4.62 | 1.68E-03 | 1.29E-02 |
| Abra | ABRA | -4.62 | 1.18E-03 | 9.87E-03 |
| Sbk3 | SBK3 | -4.61 | 1.43E-05 | 2.82E-04 |
| Cmya5 | CMYA5 | -4.60 | 7.51E-07 | 2.62E-05 |
| Mettl21e | NA | -4.58 | 8.87E-07 | 3.04E-05 |
| Crhr2 | CRHR2 | -4.58 | 2.56E-04 | 2.92E-03 |
| Opcml | OPCML | -4.57 | 1.14E-05 | 2.32E-04 |
| Cox8b | NA | -4.55 | 4.32E-06 | 1.07E-04 |
| Gprasp2 | GPRASP2 | -4.53 | 1.27E-03 | 1.04E-02 |
| Sorcs1 | SORCS1 | -4.51 | 1.80E-06 | 5.32E-05 |
| 2310057J18Rik | C6orf58 | -4.51 | 1.87E-12 | 5.26E-10 |
| Hhatl | HHATL | -4.50 | 1.22E-04 | 1.63E-03 |
| Lipf | LIPF | -4.49 | 1.70E-05 | 3.23E-04 |
| Klhl13 | KLHL13 | -4.49 | 6.11E-07 | 2.20E-05 |
| Chrne | CHRNE | -4.47 | 3.90E-03 | 2.49E-02 |
| Alpk3 | ALPK3 | -4.46 | 3.64E-07 | 1.49E-05 |
| Tceal7 | TCEAL7 | -4.45 | 4.90E-03 | 2.97E-02 |
| 3425401B19Rik | C10orf71 | -4.45 | 9.27E-06 | 1.97E-04 |
| Slco5a1 | SLCO5A1 | -4.42 | 1.05E-06 | 3.50E-05 |
| Adcyap1r1 | ADCYAP1R1 | -4.39 | 5.59E-07 | 2.09E-05 |
| Rptoros | NA | -4.38 | 1.38E-10 | 1.97E-08 |
| Hrasls | HRASLS | -4.38 | 2.12E-04 | 2.51E-03 |
| Lrrc3b | LRRC3B | -4.38 | 2.02E-03 | 1.49E-02 |
| Scgb2b7 | NA | -4.37 | 4.16E-03 | 2.62E-02 |
| Epyc | EPYC | -4.36 | 2.66E-05 | 4.67E-04 |
| Gabra4 | GABRA4 | -4.35 | 4.20E-04 | 4.36E-03 |
| Cidec | CIDEC | -4.30 | 5.84E-07 | 2.14E-05 |
| Ano5 | ANO5 | -4.30 | 2.44E-05 | 4.36E-04 |
| Jsrp1 | JSRP1 | -4.29 | 3.16E-03 | 2.13E-02 |
| Pgam2 | PGAM2 | -4.28 | 3.34E-04 | 3.63E-03 |
| Epha5 | EPHA5 | -4.27 | 2.35E-03 | 1.68E-02 |
| Vat1l | VAT1L | -4.27 | 1.21E-03 | 1.01E-02 |
| Cfd | CFD | -4.27 | 7.77E-07 | 2.70E-05 |
| 1810046K07Rik | C11orf53 | -4.26 | 1.52E-05 | 2.95E-04 |
| Zdbf2 | ZDBF2 | -4.26 | 7.31E-03 | 4.00E-02 |
| Cyp2e1 | CYP2E1 | -4.26 | 9.81E-11 | 1.52E-08 |
| Iqch | IQCH | -4.25 | 2.76E-03 | 1.91E-02 |
| Myh2 | NA | -4.23 | 1.31E-04 | 1.72E-03 |
| Tcap | TCAP | -4.22 | 3.24E-06 | 8.47E-05 |
| Fbxo40 | FBXO40 | -4.22 | 3.17E-03 | 2.14E-02 |
| Nrap | NRAP | -4.22 | 8.26E-08 | 4.37E-06 |
| Hecw1 | HECW1 | -4.22 | 9.68E-03 | 4.97E-02 |
| Klhl31 | KLHL31 | -4.20 | 3.88E-04 | 4.10E-03 |
| Tnnt3 | TNNT3 | -4.17 | 2.91E-06 | 7.81E-05 |
| Gabra3 | GABRA3 | -4.17 | 4.41E-03 | 2.74E-02 |
| Larp6 | LARP6 | -4.16 | 4.50E-03 | 2.78E-02 |
| Atp2a1 | ATP2A1 | -4.15 | 5.08E-07 | 1.93E-05 |
| Cadm2 | CADM2 | -4.14 | 3.57E-06 | 9.12E-05 |
| Lmod3 | LMOD3 | -4.14 | 5.72E-04 | 5.54E-03 |
| Ccdc85a | CCDC85A | -4.14 | 6.13E-03 | 3.51E-02 |
| Tbx4 | TBX4 | -4.13 | 2.80E-05 | 4.86E-04 |
| Wfdc13 | WFDC13 | -4.08 | 4.07E-08 | 2.42E-06 |
| Tnnt1 | TNNT1 | -4.08 | 1.40E-11 | 3.04E-09 |
| Got1l1 | GOT1L1 | -4.07 | 7.74E-03 | 4.19E-02 |
| Stx1b | STX1B | -4.07 | 2.49E-03 | 1.76E-02 |
| Dcpp1 | NA | -4.05 | 5.18E-14 | 2.66E-11 |
| Dcpp2 | NA | -3.99 | 3.08E-20 | 7.92E-17 |
| Hfe2 | HFE2 | -3.99 | 9.10E-05 | 1.29E-03 |
| Tril | TRIL | -3.97 | 2.76E-04 | 3.09E-03 |
| Trpc3 | TRPC3 | -3.97 | 7.37E-04 | 6.80E-03 |
| Prr33 | NA | -3.96 | 5.82E-04 | 5.62E-03 |
| Kcnb1 | KCNB1 | -3.93 | 1.58E-04 | 1.99E-03 |
| Eddm3b | EDDM3B | -3.93 | 7.03E-05 | 1.04E-03 |
| Myo18b | MYO18B | -3.93 | 6.44E-04 | 6.11E-03 |
| Slc17a8 | SLC17A8 | -3.92 | 2.40E-03 | 1.70E-02 |
| Myot | MYOT | -3.87 | 5.52E-05 | 8.53E-04 |
| Crispld1 | CRISPLD1 | -3.86 | 3.26E-03 | 2.18E-02 |
| A530099J19Rik | NA | -3.86 | 6.59E-03 | 3.71E-02 |
| Atp1a2 | ATP1A2 | -3.85 | 1.97E-08 | 1.35E-06 |
| Hpse2 | HPSE2 | -3.85 | 6.97E-03 | 3.86E-02 |
| Myoz1 | MYOZ1 | -3.84 | 1.13E-05 | 2.32E-04 |
| Igj | NA | -3.84 | 3.90E-07 | 1.57E-05 |
| Gmnc | GMNC | -3.83 | 2.62E-12 | 6.85E-10 |
| Hspa12b | HSPA12B | -3.82 | 2.59E-03 | 1.81E-02 |
| Slc38a5 | SLC38A5 | -3.82 | 1.22E-03 | 1.02E-02 |
| Agtr1a | AGTR1 | -3.81 | 5.04E-03 | 3.04E-02 |
| Optc | OPTC | -3.81 | 1.69E-04 | 2.09E-03 |
| Myom2 | MYOM2 | -3.79 | 9.99E-10 | 1.07E-07 |
| Asb10 | ASB10 | -3.78 | 2.75E-03 | 1.91E-02 |
| Pygm | PYGM | -3.76 | 2.56E-06 | 7.08E-05 |
| E030013I19Rik | NA | -3.75 | 1.42E-03 | 1.13E-02 |
| Crym | CRYM | -3.74 | 1.67E-03 | 1.29E-02 |
| Art1 | ART1 | -3.73 | 3.64E-05 | 6.01E-04 |
| Avpr1a | AVPR1A | -3.73 | 1.27E-04 | 1.67E-03 |
| Naalad2 | NAALAD2 | -3.73 | 6.78E-03 | 3.78E-02 |
| Ngb | NGB | -3.73 | 1.17E-03 | 9.83E-03 |
| Ckm | CKM | -3.72 | 3.57E-04 | 3.82E-03 |
| Mstn | MSTN | -3.72 | 1.64E-03 | 1.27E-02 |
| Art2a-ps | NA | -3.72 | 4.99E-03 | 3.02E-02 |
| Hhip | HHIP | -3.67 | 2.21E-07 | 9.89E-06 |
| Tmod1 | TMOD1 | -3.67 | 3.17E-08 | 2.02E-06 |
| Kcnd3 | KCND3 | -3.66 | 3.47E-05 | 5.79E-04 |
| Lrrn1 | LRRN1 | -3.65 | 5.87E-03 | 3.41E-02 |
| Treml1 | TREML1 | -3.61 | 2.16E-03 | 1.57E-02 |
| Rgs7bp | RGS7BP | -3.60 | 1.43E-03 | 1.14E-02 |
| Usp13 | USP13 | -3.60 | 3.10E-06 | 8.20E-05 |
| Fam184b | FAM184B | -3.60 | 8.80E-05 | 1.25E-03 |
| Ralyl | RALYL | -3.60 | 1.38E-03 | 1.11E-02 |
| Hrc | HRC | -3.60 | 8.10E-05 | 1.17E-03 |
| Ppp1r3a | PPP1R3A | -3.59 | 8.17E-06 | 1.77E-04 |
| Klhl41 | KLHL41 | -3.59 | 1.69E-08 | 1.22E-06 |
| Ablim2 | ABLIM2 | -3.59 | 3.86E-06 | 9.75E-05 |
| 2310065F04Rik | NA | -3.59 | 2.24E-03 | 1.62E-02 |
| Dusp27 | DUSP27 | -3.59 | 2.46E-04 | 2.82E-03 |
| Myf6 | MYF6 | -3.59 | 5.15E-03 | 3.09E-02 |
| Susd5 | SUSD5 | -3.57 | 1.72E-03 | 1.32E-02 |
| Gpm6a | GPM6A | -3.57 | 3.70E-03 | 2.40E-02 |
| A430088P11Rik | NA | -3.54 | 1.66E-04 | 2.07E-03 |
| Chrm2 | CHRM2 | -3.53 | 4.13E-04 | 4.31E-03 |
| Fbxl16 | FBXL16 | -3.53 | 4.98E-03 | 3.01E-02 |
| Psg20 | CEACAM4 | -3.52 | 2.51E-03 | 1.77E-02 |
| Gm10790 | NA | -3.52 | 3.76E-04 | 4.01E-03 |
| Svopl | SVOPL | -3.50 | 6.33E-06 | 1.45E-04 |
| Sfrp4 | SFRP4 | -3.49 | 3.18E-05 | 5.43E-04 |
| Trim54 | TRIM54 | -3.48 | 4.83E-04 | 4.87E-03 |
| Wnt2b | WNT2B | -3.48 | 6.96E-07 | 2.47E-05 |
| Apol6 | APOL6 | -3.47 | 3.39E-04 | 3.67E-03 |
| Drd1a | NA | -3.46 | 3.14E-04 | 3.44E-03 |
| Tbx15 | TBX15 | -3.44 | 4.20E-05 | 6.73E-04 |
| Cav3 | CAV3 | -3.43 | 2.03E-03 | 1.50E-02 |
| Myom1 | MYOM1 | -3.43 | 1.62E-07 | 7.73E-06 |
| 4933439C10Rik | NA | -3.42 | 8.08E-03 | 4.34E-02 |
| Sypl2 | SYPL2 | -3.41 | 2.45E-05 | 4.37E-04 |
| Klhl40 | KLHL40 | -3.41 | 5.18E-04 | 5.13E-03 |
| Sec31b | SEC31B | -3.41 | 6.30E-03 | 3.59E-02 |
| Btn2a2 | BTN2A1 | -3.39 | 4.95E-03 | 3.00E-02 |
| 4831440E17Rik | NA | -3.39 | 6.91E-03 | 3.83E-02 |
| Ank1 | ANK1 | -3.38 | 3.37E-05 | 5.67E-04 |
| Gucy1a3 | GUCY1A3 | -3.37 | 5.25E-04 | 5.18E-03 |
| Gbp10 | GBP6 | -3.36 | 4.85E-04 | 4.88E-03 |
| Tg | TG | -3.35 | 1.65E-03 | 1.27E-02 |
| Srpx | SRPX | -3.35 | 8.66E-05 | 1.24E-03 |
| Scn2a1 | SCN2A | -3.34 | 3.03E-03 | 2.06E-02 |
| Ccna1 | CCNA1 | -3.34 | 2.42E-07 | 1.06E-05 |
| Sgcg | SGCG | -3.30 | 1.58E-06 | 4.80E-05 |
| Piezo2 | PIEZO2 | -3.30 | 6.49E-03 | 3.67E-02 |
| Tet1 | TET1 | -3.30 | 3.86E-05 | 6.27E-04 |
| Frzb | FRZB | -3.29 | 6.82E-06 | 1.53E-04 |
| Hspb7 | HSPB7 | -3.27 | 1.52E-06 | 4.65E-05 |
| Cox6a2 | COX6A2 | -3.26 | 5.91E-03 | 3.42E-02 |
| Aoc3 | AOC3 | -3.26 | 2.42E-06 | 6.75E-05 |
| Runx1t1 | RUNX1T1 | -3.25 | 1.74E-03 | 1.33E-02 |
| Tmem182 | TMEM182 | -3.25 | 6.34E-05 | 9.58E-04 |
| Amigo2 | AMIGO2 | -3.23 | 3.22E-07 | 1.35E-05 |
| Wscd2 | WSCD2 | -3.22 | 3.61E-03 | 2.35E-02 |
| Mylpf | MYLPF | -3.21 | 1.56E-04 | 1.97E-03 |
| Chrna1 | CHRNA1 | -3.21 | 7.52E-03 | 4.10E-02 |
| Mzb1 | MZB1 | -3.19 | 4.56E-04 | 4.65E-03 |
| Slc4a5 | SLC4A5 | -3.18 | 3.74E-10 | 4.77E-08 |
| Obscn | OBSCN | -3.18 | 2.48E-06 | 6.87E-05 |
| Coro6 | CORO6 | -3.17 | 1.76E-03 | 1.34E-02 |
| She | SHE | -3.17 | 2.52E-03 | 1.77E-02 |
| Bpifb1 | BPIFB1 | -3.16 | 4.01E-03 | 2.55E-02 |
| Fndc5 | FNDC5 | -3.16 | 1.46E-07 | 7.12E-06 |
| 3300002I08Rik | ZNF69 | -3.16 | 9.74E-03 | 4.99E-02 |
| Egflam | EGFLAM | -3.15 | 1.18E-03 | 9.87E-03 |
| Scn4a | SCN4A | -3.13 | 2.05E-04 | 2.45E-03 |
| Cd209f | NA | -3.13 | 6.54E-03 | 3.68E-02 |
| Cpz | CPZ | -3.12 | 9.33E-03 | 4.85E-02 |
| Cacna1c | CACNA1C | -3.11 | 7.24E-04 | 6.71E-03 |
| Thrsp | THRSP | -3.09 | 1.77E-05 | 3.35E-04 |
| Zcchc5 | ZCCHC5 | -3.09 | 4.48E-03 | 2.78E-02 |
| Mypn | MYPN | -3.08 | 1.23E-05 | 2.46E-04 |
| Egf | EGF | -3.06 | 1.91E-07 | 8.64E-06 |
| Fam47e | FAM47E | -3.06 | 7.13E-03 | 3.92E-02 |
| Chrm1 | CHRM1 | -3.06 | 6.53E-05 | 9.80E-04 |
| Tnni2 | TNNI2 | -3.06 | 1.65E-05 | 3.16E-04 |
| Scn4b | SCN4B | -3.04 | 1.42E-05 | 2.79E-04 |
| A330048O09Rik | NA | -3.02 | 3.51E-05 | 5.82E-04 |
| Rcan2 | RCAN2 | -3.02 | 1.10E-12 | 3.40E-10 |
| Ptgfr | PTGFR | -3.02 | 8.12E-05 | 1.17E-03 |
| Perm1 | PERM1 | -3.02 | 7.83E-06 | 1.72E-04 |
| Cdkl1 | CDKL1 | -3.01 | 8.07E-04 | 7.37E-03 |
| Yipf7 | YIPF7 | -3.01 | 3.08E-03 | 2.09E-02 |
| Slc26a7 | SLC26A7 | -3.01 | 8.50E-03 | 4.51E-02 |
| Cxcl12 | CXCL12 | -3.00 | 1.49E-09 | 1.47E-07 |
| 5930438M14Rik | NA | -3.00 | 4.23E-03 | 2.66E-02 |
| Zfp385c | ZNF385C | -2.99 | 6.02E-03 | 3.47E-02 |
| 1810041L15Rik | KIAA1644 | -2.98 | 1.67E-04 | 2.08E-03 |
| Actn2 | ACTN2 | -2.96 | 7.43E-06 | 1.65E-04 |
| Casq1 | CASQ1 | -2.95 | 6.30E-04 | 6.00E-03 |
| Filip1 | FILIP1 | -2.94 | 3.55E-04 | 3.81E-03 |
| Lman1l | LMAN1L | -2.93 | 2.76E-07 | 1.16E-05 |
| Phactr1 | PHACTR1 | -2.93 | 1.89E-03 | 1.41E-02 |
| C4a | NA | -2.93 | 8.07E-03 | 4.33E-02 |
| Aifm3 | AIFM3 | -2.92 | 9.34E-04 | 8.21E-03 |
| Ar | AR | -2.92 | 5.83E-04 | 5.63E-03 |
| Gldn | GLDN | -2.91 | 4.20E-04 | 4.36E-03 |
| Nsg2 | NA | -2.91 | 3.41E-03 | 2.25E-02 |
| 2810468N07Rik | NA | -2.90 | 5.90E-03 | 3.42E-02 |
| AI463170 | NA | -2.90 | 2.57E-03 | 1.80E-02 |
| Pknox2 | PKNOX2 | -2.90 | 1.69E-04 | 2.09E-03 |
| Prss2 | PRSS3 | -2.89 | 5.94E-03 | 3.44E-02 |
| Pln | PLN | -2.88 | 9.03E-04 | 7.97E-03 |
| Mrvi1 | MRVI1 | -2.86 | 5.79E-05 | 8.88E-04 |
| Txlnb | TXLNB | -2.86 | 5.93E-06 | 1.37E-04 |
| B4galnt2 | B4GALNT2 | -2.86 | 2.24E-05 | 4.09E-04 |
| Zfp641 | ZNF641 | -2.86 | 1.39E-03 | 1.12E-02 |
| Slc2a5 | SLC2A5 | -2.85 | 6.40E-03 | 3.63E-02 |
| Actc1 | ACTC1 | -2.85 | 1.22E-10 | 1.81E-08 |
| Npy1r | NPY1R | -2.84 | 2.23E-03 | 1.61E-02 |
| Cacna2d3 | CACNA2D3 | -2.84 | 1.04E-03 | 8.95E-03 |
| Fam171b | FAM171B | -2.84 | 9.37E-03 | 4.86E-02 |
| Aqp7 | AQP7 | -2.84 | 8.48E-04 | 7.64E-03 |
| Wbscr17 | WBSCR17 | -2.83 | 3.82E-03 | 2.46E-02 |
| 6330419J24Rik | NA | -2.80 | 8.78E-05 | 1.25E-03 |
| Vtn | VTN | -2.79 | 6.91E-03 | 3.83E-02 |
| Pax1 | PAX1 | -2.79 | 2.42E-05 | 4.34E-04 |
| Clstn3 | CLSTN3 | -2.79 | 3.75E-03 | 2.42E-02 |
| Slc6a17 | SLC6A17 | -2.78 | 8.56E-04 | 7.68E-03 |
| Asb5 | ASB5 | -2.76 | 6.48E-04 | 6.14E-03 |
| Fsd2 | FSD2 | -2.76 | 2.18E-05 | 4.00E-04 |
| Adam23 | ADAM23 | -2.76 | 1.93E-03 | 1.44E-02 |
| Spon1 | SPON1 | -2.75 | 1.41E-06 | 4.38E-05 |
| Tff2 | TFF2 | -2.74 | 3.69E-08 | 2.28E-06 |
| Smoc1 | SMOC1 | -2.73 | 2.21E-03 | 1.60E-02 |
| Speg | SPEG | -2.71 | 2.47E-03 | 1.75E-02 |
| Eno3 | ENO3 | -2.71 | 4.02E-09 | 3.59E-07 |
| Plin4 | PLIN4 | -2.69 | 5.01E-04 | 5.01E-03 |
| Zfp951 | NA | -2.69 | 2.67E-03 | 1.86E-02 |
| Dmbt1 | DMBT1 | -2.68 | 1.30E-06 | 4.14E-05 |
| 1700040L02Rik | C10orf107 | -2.68 | 3.69E-08 | 2.28E-06 |
| Kcnj13 | KCNJ13 | -2.67 | 8.11E-06 | 1.76E-04 |
| Scn7a | SCN7A | -2.67 | 1.76E-06 | 5.27E-05 |
| Slco4c1 | SLCO4C1 | -2.67 | 9.71E-11 | 1.52E-08 |
| Smgc | NA | -2.67 | 9.62E-06 | 2.03E-04 |
| 9430037G07Rik | NA | -2.66 | 3.91E-03 | 2.50E-02 |
| Sult1c2 | SULT1C2 | -2.66 | 3.12E-06 | 8.26E-05 |
| 9330159F19Rik | KIAA0408 | -2.65 | 7.19E-03 | 3.95E-02 |
| Slc25a34 | SLC25A34 | -2.63 | 6.83E-03 | 3.80E-02 |
| 1110032F04Rik | C3orf80 | -2.63 | 3.80E-04 | 4.04E-03 |
| Fam26e | FAM26E | -2.63 | 1.56E-03 | 1.22E-02 |
| Sgce | SGCE | -2.63 | 7.68E-05 | 1.12E-03 |
| Svep1 | SVEP1 | -2.63 | 1.71E-05 | 3.25E-04 |
| Unc45b | UNC45B | -2.61 | 1.20E-03 | 1.01E-02 |
| Rspo1 | RSPO1 | -2.60 | 7.30E-03 | 4.00E-02 |
| Muc5b | MUC5B | -2.60 | 9.51E-17 | 1.13E-13 |
| Lama2 | LAMA2 | -2.60 | 3.91E-06 | 9.85E-05 |
| Lhfpl4 | LHFPL4 | -2.59 | 7.52E-03 | 4.10E-02 |
| Itgb1bp2 | ITGB1BP2 | -2.59 | 2.68E-04 | 3.01E-03 |
| Sox10 | SOX10 | -2.58 | 3.92E-03 | 2.51E-02 |
| Bend5 | BEND5 | -2.58 | 1.64E-03 | 1.27E-02 |
| Wfdc18 | NA | -2.56 | 8.75E-05 | 1.25E-03 |
| Nkain3 | NKAIN3 | -2.56 | 8.82E-03 | 4.65E-02 |
| Cyp4a12a | CYP4A22 | -2.56 | 2.71E-14 | 1.44E-11 |
| Rtn2 | RTN2 | -2.55 | 2.69E-05 | 4.71E-04 |
| 8430408G22Rik | C10orf10 | -2.54 | 7.10E-11 | 1.18E-08 |
| Srl | SRL | -2.54 | 1.22E-07 | 6.16E-06 |
| Lrrn3 | LRRN3 | -2.54 | 2.92E-08 | 1.88E-06 |
| Sbspon | SBSPON | -2.53 | 2.70E-05 | 4.71E-04 |
| Slc6a15 | SLC6A15 | -2.53 | 1.42E-03 | 1.13E-02 |
| Pgm5 | PGM5 | -2.53 | 6.89E-13 | 2.36E-10 |
| Pxmp2 | PXMP2 | -2.52 | 4.78E-09 | 4.17E-07 |
| Itga8 | ITGA8 | -2.52 | 4.98E-05 | 7.78E-04 |
| Ppp1r3c | PPP1R3C | -2.52 | 1.98E-11 | 4.12E-09 |
| Ddah1 | DDAH1 | -2.51 | 5.59E-03 | 3.29E-02 |
| Fabp3 | FABP3 | -2.51 | 3.71E-03 | 2.41E-02 |
| Mb | MB | -2.50 | 1.48E-04 | 1.91E-03 |
| Fat4 | FAT4 | -2.49 | 1.28E-03 | 1.05E-02 |
| Cnrip1 | CNRIP1 | -2.49 | 3.31E-03 | 2.21E-02 |
| Olfml1 | OLFML1 | -2.49 | 5.39E-06 | 1.27E-04 |
| Stac3 | STAC3 | -2.49 | 4.23E-03 | 2.66E-02 |
| 9330179D12Rik | NA | -2.48 | 9.74E-03 | 4.99E-02 |
| Dpy19l2 | DPY19L2 | -2.48 | 6.34E-03 | 3.61E-02 |
| Cxcr3 | CXCR3 | -2.47 | 8.28E-03 | 4.42E-02 |
| Ccdc67 | DEUP1 | -2.47 | 5.35E-06 | 1.26E-04 |
| Jph2 | JPH2 | -2.47 | 2.46E-04 | 2.82E-03 |
| Fgf7 | FGF7 | -2.46 | 1.41E-05 | 2.79E-04 |
| Rgs5 | RGS5 | -2.44 | 1.53E-05 | 2.97E-04 |
| Flnc | FLNC | -2.44 | 1.70E-05 | 3.23E-04 |
| Tmod4 | TMOD4 | -2.44 | 7.76E-04 | 7.12E-03 |
| Scgb3a2 | SCGB3A2 | -2.44 | 7.06E-15 | 4.36E-12 |
| Fgl1 | FGL1 | -2.43 | 6.53E-03 | 3.68E-02 |
| Gm4841 | NA | -2.43 | 4.52E-03 | 2.79E-02 |
| Abca8a | NA | -2.43 | 9.92E-11 | 1.52E-08 |
| Zkscan4 | ZKSCAN4 | -2.42 | 1.05E-03 | 9.06E-03 |
| Nexn | NEXN | -2.42 | 5.19E-05 | 8.07E-04 |
| Gfra1 | GFRA1 | -2.42 | 1.46E-04 | 1.89E-03 |
| 1700020N18Rik | NA | -2.42 | 6.11E-04 | 5.86E-03 |
| Ccdc159 | CCDC159 | -2.41 | 6.03E-03 | 3.47E-02 |
| Rasd2 | RASD2 | -2.41 | 6.74E-05 | 1.01E-03 |
| Mlip | MLIP | -2.40 | 7.17E-03 | 3.94E-02 |
| Adam5 | NA | -2.40 | 1.44E-03 | 1.14E-02 |
| Zcwpw1 | ZCWPW1 | -2.39 | 5.88E-03 | 3.41E-02 |
| Sh2d1b1 | SH2D1B | -2.39 | 1.02E-04 | 1.41E-03 |
| Sbk2 | SBK2 | -2.37 | 1.22E-03 | 1.01E-02 |
| Sox8 | SOX8 | -2.37 | 1.86E-03 | 1.39E-02 |
| Ryr1 | RYR1 | -2.37 | 5.33E-04 | 5.24E-03 |
| Pnmal2 | PNMAL2 | -2.37 | 5.90E-03 | 3.42E-02 |
| Abca8b | ABCA8 | -2.36 | 6.07E-05 | 9.25E-04 |
| Actg2 | ACTG2 | -2.36 | 1.30E-08 | 9.70E-07 |
| Ldb3 | LDB3 | -2.35 | 5.20E-10 | 6.13E-08 |
| Armcx2 | ARMCX2 | -2.35 | 2.74E-03 | 1.90E-02 |
| Scube1 | SCUBE1 | -2.34 | 5.23E-04 | 5.18E-03 |
| Mfap4 | MFAP4 | -2.34 | 1.05E-12 | 3.30E-10 |
| Pdgfra | PDGFRA | -2.33 | 3.61E-06 | 9.20E-05 |
| Smtnl1 | SMTNL1 | -2.33 | 1.88E-08 | 1.30E-06 |
| Adcy5 | ADCY5 | -2.32 | 3.50E-03 | 2.30E-02 |
| Nkx3-1 | NKX3-1 | -2.31 | 2.93E-12 | 7.50E-10 |
| Mme | MME | -2.31 | 1.78E-12 | 5.10E-10 |
| Rnase6 | RNASE6 | -2.31 | 1.05E-05 | 2.19E-04 |
| Abat | ABAT | -2.30 | 1.41E-05 | 2.79E-04 |
| Sncaip | SNCAIP | -2.30 | 7.05E-04 | 6.56E-03 |
| Cpxm1 | CPXM1 | -2.29 | 7.65E-03 | 4.16E-02 |
| Bhlha15 | BHLHA15 | -2.29 | 1.84E-07 | 8.42E-06 |
| Dpt | DPT | -2.28 | 9.99E-16 | 7.71E-13 |
| BC005561 | THOC2 | -2.27 | 4.00E-03 | 2.55E-02 |
| Kcnc4 | KCNC4 | -2.26 | 1.38E-03 | 1.11E-02 |
| Fam109b | FAM109B | -2.26 | 4.28E-03 | 2.68E-02 |
| Adora1 | ADORA1 | -2.25 | 4.10E-04 | 4.29E-03 |
| Tagln | TAGLN | -2.24 | 2.30E-06 | 6.48E-05 |
| Fxyd1 | FXYD1 | -2.23 | 1.36E-05 | 2.70E-04 |
| Zfp521 | ZNF521 | -2.22 | 1.30E-03 | 1.06E-02 |
| Cnn1 | CNN1 | -2.21 | 1.36E-07 | 6.69E-06 |
| Xcr1 | XCR1 | -2.21 | 3.28E-03 | 2.20E-02 |
| Cxcl15 | NA | -2.21 | 4.92E-05 | 7.69E-04 |
| Galnt16 | GALNT16 | -2.21 | 1.69E-03 | 1.30E-02 |
| Zfp354c | ZNF354C | -2.21 | 4.31E-04 | 4.45E-03 |
| Rasl12 | RASL12 | -2.21 | 2.42E-03 | 1.72E-02 |
| Smad9 | SMAD9 | -2.21 | 1.16E-03 | 9.76E-03 |
| Gp2 | GP2 | -2.21 | 1.05E-13 | 5.04E-11 |
| Lyz1 | LYZ | -2.21 | 3.54E-08 | 2.21E-06 |
| Pcdh18 | PCDH18 | -2.20 | 3.78E-04 | 4.02E-03 |
| BC030307 | NA | -2.19 | 4.18E-03 | 2.63E-02 |
| Abi3bp | ABI3BP | -2.18 | 3.63E-15 | 2.43E-12 |
| Synpo2 | SYNPO2 | -2.18 | 6.72E-08 | 3.64E-06 |
| Adhfe1 | ADHFE1 | -2.18 | 1.65E-03 | 1.28E-02 |
| Sgca | SGCA | -2.18 | 6.51E-03 | 3.67E-02 |
| Myl9 | MYL9 | -2.17 | 6.62E-08 | 3.60E-06 |
| Slc2a4rg-ps | NA | -2.17 | 3.44E-03 | 2.26E-02 |
| Derl3 | DERL3 | -2.17 | 5.95E-03 | 3.44E-02 |
| Des | DES | -2.16 | 4.78E-05 | 7.52E-04 |
| 5330417C22Rik | KIAA1324 | -2.15 | 7.03E-11 | 1.18E-08 |
| Myh11 | NA | -2.15 | 9.92E-10 | 1.07E-07 |
| Lgi1 | LGI1 | -2.13 | 1.93E-03 | 1.43E-02 |
| Ampd1 | AMPD1 | -2.13 | 5.26E-04 | 5.19E-03 |
| Xirp1 | XIRP1 | -2.12 | 4.34E-03 | 2.71E-02 |
| Gnat3 | GNAT3 | -2.12 | 8.43E-06 | 1.82E-04 |
| Mef2c | MEF2C | -2.12 | 2.68E-08 | 1.76E-06 |
| Pclo | PCLO | -2.12 | 1.36E-03 | 1.10E-02 |
| Mylk | MYLK | -2.11 | 6.90E-11 | 1.17E-08 |
| Slc25a53 | SLC25A53 | -2.11 | 9.66E-03 | 4.97E-02 |
| Cacna1s | CACNA1S | -2.11 | 8.33E-04 | 7.53E-03 |
| Sfrp2 | SFRP2 | -2.10 | 6.03E-06 | 1.39E-04 |
| Sftpa1 | SFTPA1 | -2.10 | 6.62E-04 | 6.23E-03 |
| Kcnj11 | KCNJ11 | -2.09 | 5.89E-04 | 5.68E-03 |
| Prkcq | PRKCQ | -2.08 | 1.99E-03 | 1.47E-02 |
| Bche | BCHE | -2.05 | 1.91E-06 | 5.60E-05 |
| Tmem200b | TMEM200B | -2.05 | 5.49E-03 | 3.25E-02 |
| Igsf10 | IGSF10 | -2.05 | 2.47E-03 | 1.75E-02 |
| Gstt1 | NA | -2.05 | 2.37E-05 | 4.29E-04 |
| Sult1c1 | SULT1C3 | -2.04 | 7.47E-06 | 1.65E-04 |
| Dnah8 | DNAH8 | -2.04 | 8.43E-04 | 7.61E-03 |
| Abcc9 | ABCC9 | -2.04 | 3.02E-04 | 3.32E-03 |
| Ces1c | CES1 | -2.04 | 7.87E-04 | 7.20E-03 |
| Ky | KY | -2.04 | 5.11E-03 | 3.07E-02 |
| Myom3 | MYOM3 | -2.04 | 3.17E-05 | 5.42E-04 |
| Prkg1 | PRKG1 | -2.04 | 2.11E-04 | 2.50E-03 |
| Itih2 | ITIH2 | -2.04 | 3.82E-07 | 1.55E-05 |
| Rpl3l | RPL3L | -2.02 | 6.40E-04 | 6.08E-03 |
| Wfdc15b | NA | -2.02 | 4.56E-04 | 4.65E-03 |
| Sparcl1 | SPARCL1 | -2.02 | 9.80E-09 | 7.76E-07 |
| Fbln7 | FBLN7 | -2.02 | 2.05E-04 | 2.45E-03 |
| Clec14a | CLEC14A | -2.01 | 3.36E-03 | 2.23E-02 |
| Gm12185 | NA | -2.00 | 5.67E-04 | 5.51E-03 |
| Magix | MAGIX | -2.00 | 1.90E-06 | 5.58E-05 |
| Abca4 | ABCA4 | -2.00 | 2.44E-03 | 1.73E-02 |
| Hspb6 | HSPB6 | -1.99 | 7.35E-21 | 2.84E-17 |
| Pnck | PNCK | -1.99 | 2.34E-04 | 2.72E-03 |
| Hyls1 | HYLS1 | -1.98 | 5.49E-06 | 1.29E-04 |
| Pon1 | PON1 | -1.97 | 2.59E-07 | 1.11E-05 |
| Edar | EDAR | -1.97 | 4.47E-05 | 7.08E-04 |
| Kcnk3 | KCNK3 | -1.96 | 6.50E-03 | 3.67E-02 |
| Azgp1 | AZGP1 | -1.95 | 1.04E-03 | 8.95E-03 |
| Pcp4l1 | PCP4L1 | -1.95 | 7.68E-10 | 8.41E-08 |
| Scd1 | SCD | -1.94 | 7.96E-11 | 1.31E-08 |
| C1rb | C1R | -1.94 | 2.22E-03 | 1.61E-02 |
| Ptgdr | PTGDR | -1.94 | 1.11E-03 | 9.46E-03 |
| Kcnk2 | KCNK2 | -1.94 | 4.24E-05 | 6.77E-04 |
| Bves | BVES | -1.93 | 5.11E-03 | 3.07E-02 |
| Ank2 | ANK2 | -1.93 | 8.16E-03 | 4.36E-02 |
| Ednra | EDNRA | -1.93 | 1.55E-04 | 1.96E-03 |
| Fmo3 | FMO3 | -1.92 | 2.28E-13 | 1.01E-10 |
| Gnb3 | GNB3 | -1.92 | 5.33E-09 | 4.54E-07 |
| Crip3 | CRIP3 | -1.92 | 3.89E-04 | 4.11E-03 |
| Abcc8 | ABCC8 | -1.92 | 4.63E-04 | 4.71E-03 |
| Lrrc17 | LRRC17 | -1.90 | 1.63E-05 | 3.15E-04 |
| Frem2 | FREM2 | -1.90 | 5.44E-03 | 3.22E-02 |
| Cldn8 | CLDN8 | -1.89 | 3.22E-16 | 2.93E-13 |
| Olfml3 | OLFML3 | -1.88 | 2.12E-03 | 1.55E-02 |
| Maob | MAOB | -1.88 | 1.59E-04 | 2.00E-03 |
| Twist2 | TWIST2 | -1.88 | 4.69E-03 | 2.87E-02 |
| Hoxb5 | HOXB5 | -1.87 | 7.38E-04 | 6.81E-03 |
| Mustn1 | MUSTN1 | -1.86 | 1.50E-04 | 1.92E-03 |
| F8 | F8 | -1.86 | 4.71E-03 | 2.88E-02 |
| Myh10 | MYH10 | -1.86 | 1.60E-03 | 1.24E-02 |
| Slc15a2 | SLC15A2 | -1.86 | 3.88E-18 | 7.10E-15 |
| Adcy2 | ADCY2 | -1.86 | 1.33E-16 | 1.40E-13 |
| Fgd5 | FGD5 | -1.85 | 2.79E-03 | 1.92E-02 |
| Col14a1 | COL14A1 | -1.85 | 9.16E-07 | 3.11E-05 |
| Sdk1 | SDK1 | -1.84 | 5.42E-03 | 3.21E-02 |
| Iigp1 | NA | -1.84 | 2.14E-04 | 2.52E-03 |
| Ogn | OGN | -1.84 | 2.03E-12 | 5.59E-10 |
| Pde8b | PDE8B | -1.83 | 1.26E-03 | 1.04E-02 |
| Cox7a1 | COX7A1 | -1.83 | 1.85E-04 | 2.25E-03 |
| Ntf3 | NTF3 | -1.82 | 1.44E-05 | 2.83E-04 |
| Scgb1a1 | SCGB1A1 | -1.82 | 2.34E-05 | 4.25E-04 |
| Fxyd6 | FXYD6 | -1.82 | 1.01E-11 | 2.28E-09 |
| Zfp85 | NA | -1.82 | 2.60E-04 | 2.95E-03 |
| Sptb | SPTB | -1.82 | 5.99E-05 | 9.16E-04 |
| Ptgis | PTGIS | -1.81 | 8.84E-04 | 7.85E-03 |
| Pla2g2d | PLA2G2D | -1.81 | 1.40E-03 | 1.12E-02 |
| Myb | MYB | -1.80 | 6.75E-11 | 1.16E-08 |
| Aass | AASS | -1.80 | 4.63E-04 | 4.71E-03 |
| Nkg7 | NKG7 | -1.80 | 6.02E-03 | 3.46E-02 |
| Acta2 | ACTA2 | -1.80 | 1.13E-09 | 1.18E-07 |
| Ddr2 | DDR2 | -1.80 | 4.81E-07 | 1.85E-05 |
| Lrrc23 | LRRC23 | -1.78 | 1.39E-05 | 2.74E-04 |
| Limch1 | LIMCH1 | -1.78 | 1.27E-04 | 1.67E-03 |
| Mn1 | MN1 | -1.78 | 1.64E-03 | 1.27E-02 |
| Ecm2 | ECM2 | -1.78 | 2.76E-03 | 1.91E-02 |
| Ntrk3 | NTRK3 | -1.77 | 5.88E-03 | 3.41E-02 |
| Apobec2 | APOBEC2 | -1.77 | 1.36E-03 | 1.10E-02 |
| Aldh1a2 | ALDH1A2 | -1.77 | 2.98E-04 | 3.29E-03 |
| Zfp72 | NA | -1.76 | 1.02E-03 | 8.81E-03 |
| Park2 | PARK2 | -1.76 | 4.57E-04 | 4.65E-03 |
| Dnah7a | DNAH7 | -1.76 | 7.96E-03 | 4.28E-02 |
| Aox3 | NA | -1.76 | 4.20E-16 | 3.60E-13 |
| Zfp9 | ZNF25 | -1.76 | 1.06E-03 | 9.08E-03 |
| Enpep | ENPEP | -1.75 | 3.24E-03 | 2.18E-02 |
| Tmtc1 | TMTC1 | -1.74 | 6.36E-05 | 9.60E-04 |
| Pard3b | PARD3B | -1.74 | 4.62E-06 | 1.12E-04 |
| Muc19 | NA | -1.74 | 3.59E-04 | 3.84E-03 |
| Sardh | SARDH | -1.73 | 1.67E-06 | 5.03E-05 |
| Edaradd | EDARADD | -1.73 | 4.27E-05 | 6.82E-04 |
| Trim72 | TRIM72 | -1.73 | 1.13E-04 | 1.53E-03 |
| Nfatc4 | NFATC4 | -1.73 | 7.14E-03 | 3.92E-02 |
| G630090E17Rik | NA | -1.71 | 3.87E-03 | 2.48E-02 |
| Emcn | EMCN | -1.71 | 2.27E-03 | 1.63E-02 |
| Cyp2a4 | CYP2A13 | -1.71 | 1.71E-03 | 1.31E-02 |
| Cap2 | CAP2 | -1.71 | 2.25E-05 | 4.11E-04 |
| Pycr1 | PYCR1 | -1.70 | 1.47E-03 | 1.16E-02 |
| Lmod1 | LMOD1 | -1.70 | 5.17E-06 | 1.23E-04 |
| Crisp3 | NA | -1.70 | 1.22E-03 | 1.02E-02 |
| Abca9 | ABCA9 | -1.70 | 5.33E-04 | 5.24E-03 |
| Slc12a8 | SLC12A8 | -1.69 | 1.15E-04 | 1.55E-03 |
| Csrp2 | CSRP2 | -1.69 | 1.50E-07 | 7.27E-06 |
| Bco2 | BCO2 | -1.69 | 3.03E-03 | 2.06E-02 |
| Zfp551 | ZNF551 | -1.69 | 1.09E-04 | 1.49E-03 |
| Mamdc2 | MAMDC2 | -1.69 | 2.18E-06 | 6.21E-05 |
| Cyp2d22 | CYP2D7 | -1.69 | 1.54E-05 | 2.99E-04 |
| Cpa3 | CPA3 | -1.68 | 6.39E-04 | 6.08E-03 |
| Atp2a3 | ATP2A3 | -1.68 | 1.48E-13 | 6.93E-11 |
| Sult1d1 | NA | -1.68 | 2.10E-06 | 6.06E-05 |
| Mmrn1 | MMRN1 | -1.67 | 5.51E-04 | 5.38E-03 |
| Zfp273 | NA | -1.66 | 2.05E-03 | 1.50E-02 |
| Rd3 | RD3 | -1.66 | 9.48E-03 | 4.90E-02 |
| A330023F24Rik | NA | -1.66 | 5.84E-03 | 3.40E-02 |
| Hoxa2 | HOXA2 | -1.66 | 6.19E-08 | 3.38E-06 |
| Fam13a | FAM13A | -1.66 | 2.70E-04 | 3.03E-03 |
| Rftn2 | RFTN2 | -1.66 | 3.17E-03 | 2.14E-02 |
| Lpar1 | LPAR1 | -1.66 | 2.53E-08 | 1.67E-06 |
| Airn | NA | -1.65 | 5.87E-03 | 3.41E-02 |
| Dact3 | DACT3 | -1.65 | 2.84E-03 | 1.95E-02 |
| Tekt2 | TEKT2 | -1.65 | 5.09E-03 | 3.06E-02 |
| Cacna2d1 | CACNA2D1 | -1.65 | 2.49E-05 | 4.43E-04 |
| Itga7 | ITGA7 | -1.65 | 5.25E-04 | 5.18E-03 |
| D630039A03Rik | C9orf152 | -1.64 | 1.26E-05 | 2.54E-04 |
| Trpm5 | TRPM5 | -1.64 | 3.37E-04 | 3.65E-03 |
| Baiap2l2 | BAIAP2L2 | -1.64 | 1.17E-03 | 9.81E-03 |
| Slc25a23 | SLC25A23 | -1.64 | 6.08E-12 | 1.44E-09 |
| Tnfrsf19 | TNFRSF19 | -1.63 | 2.15E-13 | 9.78E-11 |
| Col4a6 | COL4A6 | -1.63 | 4.14E-18 | 7.10E-15 |
| Nynrin | NYNRIN | -1.63 | 4.19E-04 | 4.35E-03 |
| Pdgfrb | PDGFRB | -1.63 | 3.71E-03 | 2.40E-02 |
| Zfp30 | ZFP30 | -1.62 | 8.46E-03 | 4.49E-02 |
| Slc25a21 | SLC25A21 | -1.62 | 2.27E-04 | 2.65E-03 |
| Casq2 | CASQ2 | -1.61 | 4.48E-04 | 4.59E-03 |
| Hepacam | HEPACAM | -1.61 | 9.37E-03 | 4.86E-02 |
| Itln1 | ITLN1 | -1.61 | 1.05E-07 | 5.41E-06 |
| 6430531B16Rik | NA | -1.61 | 5.80E-03 | 3.39E-02 |
| Hectd2 | HECTD2 | -1.61 | 6.83E-04 | 6.38E-03 |
| Adck3 | NA | -1.61 | 1.39E-10 | 1.97E-08 |
| Il7 | IL7 | -1.60 | 8.62E-03 | 4.56E-02 |
| Serpinf1 | SERPINF1 | -1.60 | 5.84E-07 | 2.14E-05 |
| Sh3bgr | SH3BGR | -1.59 | 9.05E-03 | 4.73E-02 |
| Tmem8b | TMEM8B | -1.59 | 1.38E-03 | 1.11E-02 |
| Rpgrip1 | RPGRIP1 | -1.58 | 6.63E-03 | 3.73E-02 |
| Bpifa1 | BPIFA1 | -1.58 | 6.40E-06 | 1.45E-04 |
| Clip3 | CLIP3 | -1.58 | 2.33E-03 | 1.67E-02 |
| Isl1 | ISL1 | -1.58 | 8.21E-13 | 2.70E-10 |
| Lepr | LEPR | -1.57 | 3.79E-04 | 4.03E-03 |
| Acsm1 | ACSM1 | -1.57 | 4.21E-13 | 1.62E-10 |
| Klf15 | KLF15 | -1.57 | 4.72E-04 | 4.78E-03 |
| Shisa2 | SHISA2 | -1.57 | 6.78E-03 | 3.78E-02 |
| Col15a1 | COL15A1 | -1.56 | 3.29E-08 | 2.08E-06 |
| Ppp1r9a | PPP1R9A | -1.56 | 5.12E-03 | 3.08E-02 |
| Apod | APOD | -1.56 | 5.28E-07 | 1.99E-05 |
| Igfbp5 | IGFBP5 | -1.56 | 5.65E-13 | 2.07E-10 |
| Dcdc2a | DCDC2 | -1.56 | 4.56E-03 | 2.81E-02 |
| Dmd | DMD | -1.55 | 4.12E-06 | 1.03E-04 |
| Cntnap2 | CNTNAP2 | -1.55 | 5.64E-03 | 3.31E-02 |
| Nkd1 | NKD1 | -1.54 | 3.54E-03 | 2.32E-02 |
| Grb14 | GRB14 | -1.53 | 9.57E-06 | 2.02E-04 |
| Upk3a | UPK3A | -1.53 | 5.84E-03 | 3.40E-02 |
| Ugt2b34 | UGT2B17 | -1.52 | 6.28E-04 | 5.99E-03 |
| Ptprn2 | PTPRN2 | -1.52 | 1.05E-05 | 2.19E-04 |
| Fam229b | FAM229B | -1.52 | 2.48E-03 | 1.75E-02 |
| Faah | FAAH | -1.52 | 9.88E-11 | 1.52E-08 |
| Capn13 | CAPN13 | -1.52 | 1.00E-04 | 1.38E-03 |
| Map1a | MAP1A | -1.51 | 1.75E-04 | 2.15E-03 |
| Cx3cr1 | CX3CR1 | -1.51 | 2.63E-08 | 1.73E-06 |
| Ntrk2 | NTRK2 | -1.50 | 9.49E-05 | 1.33E-03 |
| Epas1 | EPAS1 | -1.50 | 5.64E-12 | 1.36E-09 |
| Pkdrej | PKDREJ | -1.50 | 9.00E-03 | 4.71E-02 |
| Fbln5 | FBLN5 | -1.49 | 1.21E-03 | 1.01E-02 |
| Smyd1 | SMYD1 | -1.49 | 1.64E-05 | 3.16E-04 |
| Zfp493 | NA | -1.49 | 7.01E-03 | 3.87E-02 |
| Padi2 | PADI2 | -1.49 | 3.71E-05 | 6.07E-04 |
| Adamtsl3 | ADAMTSL3 | -1.49 | 3.88E-05 | 6.30E-04 |
| Fermt2 | FERMT2 | -1.48 | 2.63E-07 | 1.12E-05 |
| Lhfp | LHFP | -1.47 | 3.71E-04 | 3.96E-03 |
| Sftpc | SFTPC | -1.47 | 1.27E-03 | 1.04E-02 |
| Atoh8 | ATOH8 | -1.47 | 4.00E-05 | 6.45E-04 |
| Thnsl2 | THNSL2 | -1.47 | 1.54E-03 | 1.21E-02 |
| Adamtsl1 | ADAMTSL1 | -1.47 | 1.32E-04 | 1.74E-03 |
| Tacr1 | TACR1 | -1.47 | 8.71E-04 | 7.76E-03 |
| Kcnma1 | KCNMA1 | -1.47 | 1.18E-03 | 9.92E-03 |
| Pdlim3 | PDLIM3 | -1.47 | 7.39E-05 | 1.08E-03 |
| Ppp1r12b | PPP1R12B | -1.47 | 5.03E-04 | 5.02E-03 |
| Morn5 | MORN5 | -1.47 | 2.65E-03 | 1.85E-02 |
| Ccdc129 | CCDC129 | -1.47 | 1.35E-08 | 1.00E-06 |
| Cldn10 | CLDN10 | -1.46 | 4.76E-05 | 7.50E-04 |
| Ppp2r2b | PPP2R2B | -1.45 | 8.26E-07 | 2.86E-05 |
| Acnat1 | NA | -1.45 | 6.06E-03 | 3.48E-02 |
| C130074G19Rik | C1orf115 | -1.45 | 2.23E-03 | 1.61E-02 |
| Rbp4 | RBP4 | -1.44 | 2.71E-03 | 1.88E-02 |
| Sema3d | SEMA3D | -1.44 | 2.65E-10 | 3.55E-08 |
| Bmf | BMF | -1.44 | 9.02E-09 | 7.22E-07 |
| Tc2n | TC2N | -1.44 | 2.55E-12 | 6.78E-10 |
| Chad | CHAD | -1.43 | 1.16E-04 | 1.57E-03 |
| Rasgrp3 | RASGRP3 | -1.43 | 9.57E-03 | 4.93E-02 |
| Plscr4 | PLSCR4 | -1.43 | 1.42E-12 | 4.14E-10 |
| Bmpr1b | BMPR1B | -1.43 | 1.41E-08 | 1.04E-06 |
| Itga1 | ITGA1 | -1.43 | 2.19E-05 | 4.01E-04 |
| Tmod2 | TMOD2 | -1.43 | 8.57E-03 | 4.54E-02 |
| Ppargc1a | PPARGC1A | -1.42 | 7.12E-05 | 1.05E-03 |
| Fhod3 | FHOD3 | -1.42 | 8.94E-04 | 7.90E-03 |
| Dclk1 | DCLK1 | -1.42 | 7.50E-05 | 1.10E-03 |
| Smarcd3 | SMARCD3 | -1.42 | 2.35E-03 | 1.68E-02 |
| Gbp6 | GBP6 | -1.40 | 8.13E-03 | 4.35E-02 |
| C4b | C4B | -1.40 | 2.49E-06 | 6.89E-05 |
| 2810459M11Rik | C2orf72 | -1.40 | 1.85E-03 | 1.39E-02 |
| Khdrbs3 | KHDRBS3 | -1.40 | 1.14E-03 | 9.61E-03 |
| Cyp4b1 | CYP4B1 | -1.40 | 1.58E-09 | 1.52E-07 |
| Fmo1 | FMO1 | -1.40 | 6.11E-05 | 9.29E-04 |
| Lyz2 | LYZ | -1.40 | 1.92E-06 | 5.61E-05 |
| Ism1 | ISM1 | -1.40 | 4.81E-04 | 4.85E-03 |
| 4933412E12Rik | NA | -1.39 | 2.49E-03 | 1.76E-02 |
| Tmem117 | TMEM117 | -1.39 | 5.71E-04 | 5.54E-03 |
| Chrm3 | CHRM3 | -1.39 | 7.01E-03 | 3.87E-02 |
| Rarres2 | RARRES2 | -1.38 | 4.38E-05 | 6.96E-04 |
| Galnt5 | GALNT5 | -1.38 | 1.24E-10 | 1.83E-08 |
| Dnah10 | DNAH10 | -1.38 | 1.28E-03 | 1.05E-02 |
| Fgf1 | FGF1 | -1.38 | 9.96E-08 | 5.21E-06 |
| Zfp455 | ZNF738 | -1.38 | 2.15E-03 | 1.57E-02 |
| Col20a1 | COL20A1 | -1.37 | 6.71E-03 | 3.76E-02 |
| Epdr1 | EPDR1 | -1.37 | 3.34E-05 | 5.63E-04 |
| Ano1 | ANO1 | -1.37 | 2.27E-16 | 2.19E-13 |
| Lifr | LIFR | -1.37 | 1.52E-09 | 1.49E-07 |
| Gpr124 | NA | -1.37 | 6.80E-03 | 3.79E-02 |
| Tmem47 | TMEM47 | -1.37 | 4.55E-04 | 4.65E-03 |
| Sspn | SSPN | -1.37 | 1.62E-04 | 2.03E-03 |
| Sidt1 | SIDT1 | -1.36 | 5.98E-10 | 6.89E-08 |
| Akr1e1 | AKR1E2 | -1.36 | 5.64E-05 | 8.70E-04 |
| Cpq | CPQ | -1.36 | 3.87E-06 | 9.76E-05 |
| Zeb1 | ZEB1 | -1.35 | 3.47E-04 | 3.74E-03 |
| Creb3l1 | CREB3L1 | -1.35 | 6.10E-08 | 3.35E-06 |
| Prrx1 | PRRX1 | -1.35 | 1.26E-03 | 1.04E-02 |
| Ptprd | PTPRD | -1.34 | 1.02E-04 | 1.41E-03 |
| Acacb | ACACB | -1.34 | 2.31E-05 | 4.21E-04 |
| Mpdz | MPDZ | -1.34 | 1.50E-04 | 1.92E-03 |
| Acsf2 | ACSF2 | -1.34 | 9.76E-16 | 7.71E-13 |
| Angpt1 | ANGPT1 | -1.34 | 3.58E-03 | 2.34E-02 |
| Eltd1 | NA | -1.33 | 4.96E-03 | 3.01E-02 |
| Zfp760 | NA | -1.33 | 2.33E-03 | 1.67E-02 |
| Camk2n1 | CAMK2N1 | -1.33 | 1.42E-06 | 4.40E-05 |
| Foxf1 | FOXF1 | -1.33 | 5.04E-03 | 3.04E-02 |
| Caprin2 | CAPRIN2 | -1.32 | 6.71E-03 | 3.76E-02 |
| Psg17 | CEACAM4 | -1.32 | 6.82E-03 | 3.80E-02 |
| Smim6 | SMIM6 | -1.32 | 1.58E-04 | 1.99E-03 |
| Lilra5 | LILRA2 | -1.32 | 2.14E-03 | 1.56E-02 |
| Tenm3 | TENM3 | -1.32 | 8.86E-05 | 1.26E-03 |
| Dhdh | DHDH | -1.32 | 2.24E-03 | 1.62E-02 |
| Aqp11 | AQP11 | -1.32 | 2.48E-03 | 1.75E-02 |
| Zfp712 | NA | -1.31 | 9.23E-03 | 4.81E-02 |
| Dnajc28 | DNAJC28 | -1.31 | 2.30E-03 | 1.65E-02 |
| H2-Q1 | HLA-E | -1.31 | 1.69E-04 | 2.09E-03 |
| Nrep | NREP | -1.30 | 2.47E-05 | 4.39E-04 |
| Usp11 | USP11 | -1.30 | 9.46E-05 | 1.33E-03 |
| Creb3l4 | CREB3L4 | -1.30 | 2.66E-05 | 4.67E-04 |
| Plekhd1 | PLEKHD1 | -1.30 | 4.40E-03 | 2.74E-02 |
| Zfp383 | ZNF383 | -1.30 | 3.18E-03 | 2.14E-02 |
| Tnfsf10 | TNFSF10 | -1.30 | 1.99E-06 | 5.77E-05 |
| Meig1 | MEIG1 | -1.30 | 7.29E-03 | 4.00E-02 |
| Dlc1 | DLC1 | -1.29 | 6.15E-03 | 3.52E-02 |
| Fcgbp | FCGBP | -1.29 | 1.18E-08 | 8.98E-07 |
| Zfp41 | ZFP41 | -1.29 | 2.45E-03 | 1.74E-02 |
| St3gal5 | ST3GAL5 | -1.29 | 1.87E-09 | 1.77E-07 |
| Stc2 | STC2 | -1.28 | 3.26E-05 | 5.53E-04 |
| Grb10 | GRB10 | -1.28 | 3.30E-03 | 2.20E-02 |
| Hepacam2 | HEPACAM2 | -1.28 | 5.10E-04 | 5.07E-03 |
| Cped1 | CPED1 | -1.28 | 7.31E-03 | 4.00E-02 |
| Reck | RECK | -1.28 | 5.84E-03 | 3.40E-02 |
| Lgr6 | LGR6 | -1.28 | 3.99E-03 | 2.55E-02 |
| Mcpt4 | NA | -1.28 | 9.48E-03 | 4.90E-02 |
| Zfp318 | ZNF318 | -1.27 | 1.86E-08 | 1.30E-06 |
| Fhl1 | FHL1 | -1.27 | 4.96E-06 | 1.19E-04 |
| Zfp763 | NA | -1.27 | 1.86E-05 | 3.48E-04 |
| Gng13 | GNG13 | -1.26 | 4.01E-04 | 4.21E-03 |
| Gata3 | GATA3 | -1.26 | 2.64E-03 | 1.84E-02 |
| Prr15 | PRR15 | -1.26 | 4.10E-03 | 2.60E-02 |
| Kctd12b | NA | -1.25 | 1.91E-05 | 3.55E-04 |
| Sobp | SOBP | -1.25 | 6.85E-03 | 3.81E-02 |
| Ahcyl2 | AHCYL2 | -1.24 | 9.86E-12 | 2.27E-09 |
| Cyp4f15 | CYP4F8 | -1.24 | 1.32E-03 | 1.07E-02 |
| Lzts3 | LZTS3 | -1.24 | 4.04E-09 | 3.59E-07 |
| Me3 | ME3 | -1.24 | 6.90E-05 | 1.03E-03 |
| Tsnaxip1 | TSNAXIP1 | -1.24 | 7.42E-03 | 4.05E-02 |
| Zfp108 | NA | -1.24 | 9.87E-04 | 8.59E-03 |
| Lemd1 | LEMD1 | -1.24 | 2.40E-03 | 1.71E-02 |
| Wnt5b | WNT5B | -1.24 | 4.94E-04 | 4.96E-03 |
| Olfml2b | OLFML2B | -1.24 | 3.31E-03 | 2.21E-02 |
| Aox1 | AOX1 | -1.24 | 2.88E-06 | 7.77E-05 |
| Hdac11 | HDAC11 | -1.24 | 7.23E-05 | 1.07E-03 |
| Serping1 | SERPING1 | -1.23 | 3.38E-06 | 8.73E-05 |
| Mturn | MTURN | -1.23 | 9.03E-03 | 4.72E-02 |
| Cldn2 | CLDN2 | -1.23 | 4.43E-04 | 4.55E-03 |
| Fras1 | FRAS1 | -1.23 | 4.35E-04 | 4.48E-03 |
| Kcne3 | KCNE3 | -1.22 | 1.11E-11 | 2.48E-09 |
| Slc27a2 | SLC27A2 | -1.22 | 4.42E-03 | 2.75E-02 |
| Glt8d2 | GLT8D2 | -1.22 | 1.48E-03 | 1.17E-02 |
| Ptpru | PTPRU | -1.21 | 2.89E-08 | 1.87E-06 |
| Stil | STIL | -1.20 | 6.88E-03 | 3.82E-02 |
| 4930412C18Rik | NA | -1.20 | 8.44E-03 | 4.48E-02 |
| Susd2 | SUSD2 | -1.20 | 7.23E-03 | 3.97E-02 |
| Rhoj | RHOJ | -1.20 | 1.08E-03 | 9.23E-03 |
| Igdcc4 | IGDCC4 | -1.20 | 2.01E-04 | 2.40E-03 |
| Ang | ANG | -1.18 | 2.10E-03 | 1.53E-02 |
| Ptprb | PTPRB | -1.18 | 3.09E-03 | 2.09E-02 |
| Kcnq1 | KCNQ1 | -1.18 | 1.45E-08 | 1.07E-06 |
| Dcn | DCN | -1.18 | 1.36E-05 | 2.70E-04 |
| Gm684 | COLCA2 | -1.17 | 4.40E-05 | 6.98E-04 |
| Olfml2a | OLFML2A | -1.17 | 1.56E-06 | 4.77E-05 |
| Copz2 | COPZ2 | -1.17 | 1.26E-06 | 4.06E-05 |
| Nr6a1 | NR6A1 | -1.17 | 4.43E-06 | 1.09E-04 |
| Stxbp6 | STXBP6 | -1.17 | 2.76E-08 | 1.80E-06 |
| Kcnj16 | KCNJ16 | -1.16 | 2.95E-04 | 3.27E-03 |
| Asap3 | ASAP3 | -1.16 | 4.91E-06 | 1.18E-04 |
| Fabp4 | FABP4 | -1.16 | 1.75E-03 | 1.33E-02 |
| Mtss1l | MTSS1L | -1.16 | 2.42E-05 | 4.34E-04 |
| Tns1 | TNS1 | -1.16 | 4.36E-08 | 2.54E-06 |
| Acss1 | ACSS1 | -1.16 | 2.68E-15 | 1.88E-12 |
| Scgb3a1 | SCGB3A1 | -1.15 | 2.47E-08 | 1.64E-06 |
| Cacnb1 | CACNB1 | -1.15 | 1.90E-03 | 1.41E-02 |
| Pygb | PYGB | -1.15 | 3.24E-12 | 7.94E-10 |
| Sall2 | SALL2 | -1.15 | 2.08E-03 | 1.52E-02 |
| Pcca | PCCA | -1.15 | 1.64E-05 | 3.15E-04 |
| Cplx2 | CPLX2 | -1.15 | 2.14E-04 | 2.53E-03 |
| Sema4c | SEMA4C | -1.15 | 3.68E-06 | 9.35E-05 |
| Rad51b | RAD51B | -1.14 | 5.61E-08 | 3.14E-06 |
| Cyp2j9 | CYP2J2 | -1.14 | 3.09E-05 | 5.31E-04 |
| Rbbp9 | RBBP9 | -1.14 | 1.30E-08 | 9.70E-07 |
| Medag | MEDAG | -1.14 | 5.02E-04 | 5.01E-03 |
| Arxes2 | NA | -1.14 | 7.66E-03 | 4.16E-02 |
| Igf2 | IGF2 | -1.13 | 8.47E-03 | 4.50E-02 |
| Lypd6 | LYPD6 | -1.13 | 3.79E-03 | 2.44E-02 |
| Tfcp2l1 | TFCP2L1 | -1.12 | 1.46E-04 | 1.88E-03 |
| 1810010H24Rik | C17orf58 | -1.12 | 7.76E-05 | 1.13E-03 |
| Ppfia4 | PPFIA4 | -1.12 | 3.36E-03 | 2.23E-02 |
| Wdr5b | WDR5B | -1.12 | 8.12E-03 | 4.35E-02 |
| Snhg18 | NA | -1.12 | 2.72E-04 | 3.04E-03 |
| Chst9 | CHST9 | -1.11 | 4.12E-03 | 2.61E-02 |
| Slc2a4 | SLC2A4 | -1.11 | 1.36E-05 | 2.70E-04 |
| Fam161a | FAM161A | -1.11 | 5.44E-03 | 3.22E-02 |
| Sox5 | SOX5 | -1.11 | 1.63E-04 | 2.03E-03 |
| Phka1 | PHKA1 | -1.11 | 1.42E-05 | 2.80E-04 |
| Dach1 | DACH1 | -1.10 | 8.02E-03 | 4.31E-02 |
| Cables1 | CABLES1 | -1.10 | 4.00E-03 | 2.55E-02 |
| Mrc2 | MRC2 | -1.10 | 4.13E-03 | 2.61E-02 |
| Jam2 | JAM2 | -1.10 | 1.12E-03 | 9.54E-03 |
| Smoc2 | SMOC2 | -1.10 | 4.32E-04 | 4.46E-03 |
| Akr1c13 | AKR1C3 | -1.09 | 1.95E-05 | 3.63E-04 |
| Klhl17 | KLHL17 | -1.09 | 2.71E-03 | 1.88E-02 |
| Slc9a7 | SLC9A7 | -1.09 | 1.43E-04 | 1.86E-03 |
| Six4 | SIX4 | -1.09 | 1.74E-06 | 5.23E-05 |
| Sod3 | SOD3 | -1.09 | 2.14E-04 | 2.52E-03 |
| Mettl7a1 | METTL7A | -1.09 | 1.42E-09 | 1.42E-07 |
| Osbpl6 | OSBPL6 | -1.08 | 5.26E-04 | 5.19E-03 |
| Prelp | PRELP | -1.08 | 8.23E-04 | 7.45E-03 |
| Mycl | MYCL | -1.08 | 2.22E-12 | 6.00E-10 |
| Slc4a4 | SLC4A4 | -1.08 | 8.15E-04 | 7.42E-03 |
| Tmem220 | TMEM220 | -1.07 | 5.20E-05 | 8.07E-04 |
| Dio2 | DIO2 | -1.07 | 2.63E-04 | 2.97E-03 |
| Itih5 | ITIH5 | -1.07 | 9.18E-04 | 8.09E-03 |
| Eda | EDA | -1.07 | 3.43E-03 | 2.26E-02 |
| Pik3ip1 | PIK3IP1 | -1.06 | 5.40E-04 | 5.28E-03 |
| Ern2 | ERN2 | -1.06 | 6.55E-04 | 6.20E-03 |
| Tcea3 | TCEA3 | -1.06 | 3.40E-03 | 2.25E-02 |
| Rbms3 | RBMS3 | -1.06 | 2.42E-05 | 4.34E-04 |
| Pfkm | PFKM | -1.06 | 1.50E-05 | 2.92E-04 |
| Sytl3 | SYTL3 | -1.06 | 7.22E-03 | 3.96E-02 |
| Myc | NA | -1.05 | 1.18E-08 | 8.98E-07 |
| Fcgrt | FCGRT | -1.05 | 7.26E-05 | 1.07E-03 |
| Sectm1b | SECTM1 | -1.05 | 1.10E-03 | 9.43E-03 |
| C1ra | C1R | -1.05 | 2.27E-04 | 2.65E-03 |
| Aldh6a1 | ALDH6A1 | -1.05 | 4.67E-08 | 2.69E-06 |
| Dtna | DTNA | -1.05 | 2.26E-03 | 1.63E-02 |
| Adamts15 | ADAMTS15 | -1.05 | 5.65E-03 | 3.32E-02 |
| Sult1b1 | SULT1B1 | -1.04 | 2.59E-03 | 1.82E-02 |
| Itpr1 | ITPR1 | -1.04 | 1.24E-07 | 6.22E-06 |
| Rgs4 | RGS4 | -1.04 | 2.29E-03 | 1.64E-02 |
| Abca13 | ABCA13 | -1.04 | 6.37E-07 | 2.29E-05 |
| E2f7 | E2F7 | -1.03 | 6.30E-03 | 3.59E-02 |
| Asb13 | ASB13 | -1.03 | 6.22E-10 | 7.06E-08 |
| 1700001O22Rik | C9orf50 | -1.03 | 9.29E-03 | 4.84E-02 |
| Ncald | NCALD | -1.03 | 4.01E-04 | 4.21E-03 |
| Slc16a7 | SLC16A7 | -1.03 | 1.61E-04 | 2.02E-03 |
| Akap6 | AKAP6 | -1.03 | 9.30E-03 | 4.84E-02 |
| Irs1 | IRS1 | -1.03 | 2.55E-04 | 2.91E-03 |
| Grem2 | GREM2 | -1.02 | 7.08E-03 | 3.90E-02 |
| Fam174b | FAM174B | -1.02 | 2.73E-10 | 3.63E-08 |
| Lgi2 | LGI2 | -1.02 | 1.27E-07 | 6.34E-06 |
| Gstk1 | GSTK1 | -1.02 | 5.86E-11 | 1.03E-08 |
| Slc34a2 | SLC34A2 | -1.02 | 7.61E-07 | 2.65E-05 |
| Gabrp | GABRP | -1.01 | 1.54E-07 | 7.41E-06 |
| Zfp3 | ZFP3 | -1.01 | 1.53E-03 | 1.21E-02 |
| Slc10a5 | SLC10A5 | -1.01 | 4.90E-05 | 7.69E-04 |
| Zfp39 | NA | -1.01 | 2.54E-04 | 2.90E-03 |
| Zfp395 | ZNF395 | -1.00 | 3.32E-07 | 1.38E-05 |
| Calcrl | CALCRL | -1.00 | 4.36E-03 | 2.72E-02 |
| Mdm1 | MDM1 | -1.00 | 2.57E-03 | 1.81E-02 |
| Syde1 | SYDE1 | -1.00 | 8.74E-04 | 7.78E-03 |
| Scnn1g | SCNN1G | -1.00 | 4.08E-06 | 1.02E-04 |
| Ces1e | NA | -1.00 | 5.35E-09 | 4.54E-07 |
| Chpt1 | CHPT1 | -1.00 | 6.15E-10 | 7.03E-08 |
| Firre | NA | -1.00 | 2.76E-03 | 1.91E-02 |
| Igfbp4 | IGFBP4 | -0.99 | 1.26E-08 | 9.51E-07 |
| Krt18 | KRT18 | -0.99 | 5.55E-10 | 6.45E-08 |
| Pck2 | PCK2 | -0.99 | 2.04E-05 | 3.78E-04 |
| Plce1 | PLCE1 | -0.99 | 1.22E-04 | 1.63E-03 |
| Decr1 | DECR1 | -0.99 | 7.41E-10 | 8.18E-08 |
| Scara3 | SCARA3 | -0.99 | 2.65E-03 | 1.85E-02 |
| Fmo5 | FMO5 | -0.98 | 1.03E-09 | 1.08E-07 |
| Nhlrc1 | NHLRC1 | -0.98 | 9.24E-04 | 8.14E-03 |
| Mgst1 | MGST1 | -0.98 | 1.74E-10 | 2.44E-08 |
| Abhd14b | ABHD14B | -0.98 | 1.34E-10 | 1.95E-08 |
| Slc44a3 | SLC44A3 | -0.98 | 7.27E-05 | 1.07E-03 |
| Ppp1r1b | PPP1R1B | -0.97 | 1.61E-05 | 3.11E-04 |
| Ccdc149 | CCDC149 | -0.97 | 5.66E-05 | 8.73E-04 |
| Cd34 | CD34 | -0.97 | 1.63E-03 | 1.26E-02 |
| Nid1 | NID1 | -0.97 | 1.61E-03 | 1.25E-02 |
| Nrbp2 | NRBP2 | -0.97 | 2.10E-09 | 1.96E-07 |
| Art3 | ART3 | -0.96 | 4.18E-04 | 4.35E-03 |
| Xist | NA | -0.96 | 6.04E-08 | 3.33E-06 |
| Pde3b | PDE3B | -0.96 | 4.57E-03 | 2.82E-02 |
| Cyp2f2 | CYP2F1 | -0.96 | 3.41E-05 | 5.71E-04 |
| Foxp2 | FOXP2 | -0.96 | 5.78E-09 | 4.82E-07 |
| Setbp1 | SETBP1 | -0.96 | 1.33E-07 | 6.59E-06 |
| Pam | PAM | -0.96 | 5.73E-09 | 4.82E-07 |
| Plcb4 | PLCB4 | -0.96 | 1.03E-06 | 3.41E-05 |
| Emb | EMB | -0.95 | 3.42E-08 | 2.16E-06 |
| Lypd2 | LYPD2 | -0.95 | 1.24E-08 | 9.42E-07 |
| Fam3c | FAM3C | -0.95 | 7.83E-10 | 8.51E-08 |
| Zfp157 | NA | -0.95 | 1.26E-03 | 1.04E-02 |
| Arhgap29 | ARHGAP29 | -0.95 | 5.75E-07 | 2.12E-05 |
| Cyp2a5 | CYP2A13 | -0.94 | 1.14E-05 | 2.33E-04 |
| Tspyl4 | TSPYL4 | -0.94 | 1.34E-03 | 1.09E-02 |
| Lipt2 | LIPT2 | -0.94 | 3.06E-03 | 2.08E-02 |
| Trnp1 | TRNP1 | -0.94 | 9.08E-03 | 4.74E-02 |
| Bgn | BGN | -0.94 | 2.08E-04 | 2.47E-03 |
| 1700029J07Rik | C4orf47 | -0.94 | 9.32E-03 | 4.85E-02 |
| Zc3hav1l | ZC3HAV1L | -0.94 | 5.51E-03 | 3.25E-02 |
| Oat | OAT | -0.94 | 5.31E-09 | 4.54E-07 |
| S100g | S100G | -0.94 | 8.82E-04 | 7.84E-03 |
| Rragd | RRAGD | -0.93 | 6.04E-03 | 3.47E-02 |
| Nin | NIN | -0.93 | 2.44E-07 | 1.07E-05 |
| Cbr2 | NA | -0.93 | 4.11E-05 | 6.59E-04 |
| Fzd8 | FZD8 | -0.93 | 9.81E-04 | 8.55E-03 |
| Ces1g | NA | -0.93 | 2.15E-03 | 1.56E-02 |
| Tmem9 | TMEM9 | -0.93 | 1.09E-06 | 3.60E-05 |
| Alox15 | ALOX15 | -0.93 | 6.40E-06 | 1.45E-04 |
| Map3k1 | MAP3K1 | -0.92 | 1.21E-11 | 2.67E-09 |
| Trim68 | TRIM68 | -0.92 | 1.47E-03 | 1.17E-02 |
| Zfp956 | NA | -0.92 | 1.69E-03 | 1.30E-02 |
| Cdk6 | CDK6 | -0.92 | 1.80E-03 | 1.36E-02 |
| Rab19 | RAB19 | -0.92 | 6.52E-04 | 6.17E-03 |
| Rttn | RTTN | -0.92 | 1.76E-03 | 1.33E-02 |
| Deptor | DEPTOR | -0.92 | 4.98E-08 | 2.84E-06 |
| Mapk1ip1 | MAPK1IP1L | -0.91 | 4.89E-03 | 2.97E-02 |
| Slc38a1 | SLC38A1 | -0.91 | 1.38E-03 | 1.11E-02 |
| Ccdc66 | CCDC66 | -0.91 | 8.37E-03 | 4.45E-02 |
| Pm20d2 | PM20D2 | -0.91 | 8.40E-04 | 7.59E-03 |
| Bcl2l14 | BCL2L14 | -0.90 | 1.19E-03 | 9.96E-03 |
| C3 | C3 | -0.90 | 1.82E-04 | 2.22E-03 |
| Akr1c18 | NA | -0.90 | 1.01E-08 | 7.99E-07 |
| Parva | PARVA | -0.90 | 2.31E-08 | 1.55E-06 |
| Slamf9 | SLAMF9 | -0.90 | 7.16E-03 | 3.94E-02 |
| Nsun4 | NSUN4 | -0.90 | 8.21E-05 | 1.18E-03 |
| Bmx | BMX | -0.89 | 2.86E-10 | 3.78E-08 |
| Pde4d | PDE4D | -0.89 | 6.11E-07 | 2.20E-05 |
| Lurap1 | LURAP1 | -0.89 | 6.49E-03 | 3.67E-02 |
| Synpo | SYNPO | -0.89 | 1.10E-05 | 2.26E-04 |
| Rab17 | RAB17 | -0.89 | 2.54E-05 | 4.50E-04 |
| Syde2 | SYDE2 | -0.89 | 1.87E-04 | 2.27E-03 |
| Ttc28 | TTC28 | -0.89 | 1.39E-03 | 1.12E-02 |
| Sytl4 | SYTL4 | -0.88 | 5.96E-05 | 9.12E-04 |
| 5730409E04Rik | C1orf216 | -0.88 | 6.07E-03 | 3.48E-02 |
| 1110051M20Rik | C11orf49 | -0.88 | 3.83E-05 | 6.25E-04 |
| Zfp59 | ZNF780A | -0.88 | 4.59E-03 | 2.82E-02 |
| Il6st | IL6ST | -0.88 | 1.47E-09 | 1.46E-07 |
| Clec2d | CLEC2A | -0.88 | 4.64E-06 | 1.13E-04 |
| Efemp1 | EFEMP1 | -0.88 | 3.64E-11 | 6.68E-09 |
| Tmem42 | TMEM42 | -0.88 | 3.36E-03 | 2.23E-02 |
| Casc4 | CASC4 | -0.88 | 3.64E-04 | 3.90E-03 |
| Sdc2 | SDC2 | -0.87 | 4.91E-05 | 7.69E-04 |
| Wfdc1 | WFDC1 | -0.87 | 1.54E-03 | 1.21E-02 |
| Il17rd | IL17RD | -0.87 | 4.44E-04 | 4.56E-03 |
| Gm14326 | NA | -0.87 | 8.69E-03 | 4.59E-02 |
| Cx3cl1 | CX3CL1 | -0.87 | 1.62E-06 | 4.90E-05 |
| Npnt | NPNT | -0.87 | 2.57E-04 | 2.92E-03 |
| Sftpd | SFTPD | -0.87 | 6.88E-05 | 1.03E-03 |
| Cyp4a12b | CYP4A22 | -0.86 | 2.64E-05 | 4.63E-04 |
| Tmem181b-ps | NA | -0.86 | 4.28E-03 | 2.68E-02 |
| Lyplal1 | LYPLAL1 | -0.85 | 4.55E-04 | 4.65E-03 |
| Ces1d | NA | -0.85 | 2.17E-05 | 3.99E-04 |
| Amigo1 | AMIGO1 | -0.85 | 1.64E-05 | 3.16E-04 |
| Gm10336 | NA | -0.85 | 8.77E-03 | 4.62E-02 |
| Plxna3 | PLXNA3 | -0.85 | 1.18E-06 | 3.85E-05 |
| C1rl | C1RL | -0.85 | 2.71E-03 | 1.88E-02 |
| Zfp937 | NA | -0.85 | 5.34E-04 | 5.24E-03 |
| Ppm1k | PPM1K | -0.85 | 4.37E-05 | 6.95E-04 |
| Sult1a1 | SULT1A2 | -0.85 | 3.63E-03 | 2.36E-02 |
| H2-DMb1 | HLA-DMB | -0.85 | 5.35E-04 | 5.25E-03 |
| Sntb1 | SNTB1 | -0.85 | 9.54E-04 | 8.36E-03 |
| Plscr2 | PLSCR1 | -0.84 | 1.36E-03 | 1.10E-02 |
| Hey1 | HEY1 | -0.84 | 5.12E-04 | 5.08E-03 |
| C1s1 | C1S | -0.84 | 9.49E-04 | 8.32E-03 |
| Csad | CSAD | -0.84 | 2.14E-06 | 6.12E-05 |
| Acsm3 | ACSM3 | -0.84 | 1.16E-04 | 1.56E-03 |
| Enpp5 | ENPP5 | -0.84 | 2.10E-08 | 1.44E-06 |
| Hpn | HPN | -0.83 | 3.40E-03 | 2.25E-02 |
| Id4 | ID4 | -0.83 | 9.60E-05 | 1.34E-03 |
| Ramp1 | RAMP1 | -0.83 | 4.35E-03 | 2.71E-02 |
| Pdk2 | PDK2 | -0.83 | 2.32E-05 | 4.23E-04 |
| Casp12 | CASP12 | -0.83 | 3.95E-06 | 9.92E-05 |
| E030019B13Rik | NA | -0.83 | 1.51E-04 | 1.92E-03 |
| Dqx1 | DQX1 | -0.83 | 4.61E-03 | 2.83E-02 |
| Ttbk2 | TTBK2 | -0.83 | 1.22E-04 | 1.63E-03 |
| Prss32 | NA | -0.83 | 1.76E-05 | 3.32E-04 |
| Fam120c | FAM120C | -0.83 | 6.79E-06 | 1.53E-04 |
| Mill2 | MICA | -0.82 | 4.63E-03 | 2.84E-02 |
| Il11ra1 | IL11RA | -0.82 | 5.61E-03 | 3.30E-02 |
| Lpcat1 | LPCAT1 | -0.82 | 1.13E-04 | 1.53E-03 |
| Txndc16 | TXNDC16 | -0.82 | 4.13E-04 | 4.31E-03 |
| Cadps2 | CADPS2 | -0.82 | 3.09E-06 | 8.19E-05 |
| Adcy3 | ADCY3 | -0.82 | 1.65E-07 | 7.77E-06 |
| Ccdc166 | CCDC166 | -0.82 | 5.31E-03 | 3.16E-02 |
| Ispd | ISPD | -0.82 | 5.61E-03 | 3.30E-02 |
| Bicd1 | BICD1 | -0.82 | 4.43E-03 | 2.75E-02 |
| Ces1f | NA | -0.82 | 4.32E-03 | 2.70E-02 |
| Ntn1 | NTN1 | -0.82 | 2.79E-03 | 1.92E-02 |
| Cyp39a1 | CYP39A1 | -0.81 | 1.05E-07 | 5.41E-06 |
| F5 | F5 | -0.81 | 8.09E-03 | 4.34E-02 |
| Zmym3 | ZMYM3 | -0.81 | 4.55E-05 | 7.20E-04 |
| Klhl23 | KLHL23 | -0.81 | 7.72E-03 | 4.19E-02 |
| Cand2 | CAND2 | -0.81 | 3.35E-04 | 3.63E-03 |
| Ngfr | NGFR | -0.81 | 3.50E-04 | 3.77E-03 |
| Col4a5 | COL4A5 | -0.81 | 3.55E-05 | 5.87E-04 |
| Tshz2 | TSHZ2 | -0.81 | 3.73E-08 | 2.29E-06 |
| Spry2 | SPRY2 | -0.81 | 7.77E-05 | 1.13E-03 |
| Tmprss2 | TMPRSS2 | -0.80 | 5.25E-08 | 2.97E-06 |
| Pkp2 | PKP2 | -0.80 | 9.49E-07 | 3.20E-05 |
| Rif1 | RIF1 | -0.80 | 3.86E-05 | 6.27E-04 |
| Cftr | CFTR | -0.80 | 8.97E-03 | 4.70E-02 |
| Ffar4 | FFAR4 | -0.80 | 7.56E-06 | 1.67E-04 |
| Tmem107 | TMEM107 | -0.80 | 5.38E-03 | 3.20E-02 |
| Tox3 | TOX3 | -0.80 | 1.54E-03 | 1.21E-02 |
| Gstm7 | GSTM2 | -0.80 | 8.26E-03 | 4.41E-02 |
| Macrod1 | MACROD1 | -0.80 | 5.16E-03 | 3.10E-02 |
| Ccr2 | CCR2 | -0.79 | 2.38E-04 | 2.75E-03 |
| Cyp2b10 | CYP2B6 | -0.79 | 7.12E-07 | 2.52E-05 |
| Kank2 | KANK2 | -0.79 | 6.81E-05 | 1.02E-03 |
| Cep128 | CEP128 | -0.79 | 7.35E-03 | 4.02E-02 |
| Sertad4 | SERTAD4 | -0.79 | 1.89E-04 | 2.28E-03 |
| Fgfr3 | FGFR3 | -0.79 | 3.93E-08 | 2.37E-06 |
| Zfp36l1 | ZFP36L1 | -0.79 | 1.71E-07 | 7.95E-06 |
| Tmem176b | TMEM176B | -0.79 | 5.18E-05 | 8.07E-04 |
| Dixdc1 | DIXDC1 | -0.79 | 1.24E-04 | 1.64E-03 |
| Ifi44 | IFI44 | -0.79 | 7.14E-04 | 6.64E-03 |
| Chd3 | CHD3 | -0.79 | 3.92E-08 | 2.37E-06 |
| Aqp1 | AQP1 | -0.79 | 3.17E-03 | 2.14E-02 |
| Gpm6b | GPM6B | -0.79 | 8.59E-03 | 4.55E-02 |
| Hint2 | HINT2 | -0.78 | 1.31E-05 | 2.61E-04 |
| Tst | TST | -0.78 | 4.69E-07 | 1.83E-05 |
| Tmtc4 | TMTC4 | -0.78 | 1.16E-06 | 3.78E-05 |
| Muc1 | MUC1 | -0.78 | 9.26E-06 | 1.97E-04 |
| Patz1 | PATZ1 | -0.78 | 1.57E-03 | 1.23E-02 |
| Nnt | NNT | -0.78 | 1.51E-07 | 7.30E-06 |
| Steap2 | STEAP2 | -0.78 | 1.40E-03 | 1.12E-02 |
| Tmem186 | TMEM186 | -0.78 | 8.22E-04 | 7.45E-03 |
| Usp28 | USP28 | -0.78 | 2.47E-03 | 1.75E-02 |
| Hoxa3 | HOXA3 | -0.78 | 5.18E-04 | 5.14E-03 |
| Pxylp1 | PXYLP1 | -0.77 | 1.58E-04 | 1.99E-03 |
| Eml1 | EML1 | -0.77 | 5.63E-03 | 3.31E-02 |
| Nadk2 | NADK2 | -0.77 | 6.84E-06 | 1.53E-04 |
| Morn2 | MORN2 | -0.77 | 5.93E-03 | 3.43E-02 |
| Agmo | AGMO | -0.77 | 1.87E-07 | 8.49E-06 |
| Aldh1a1 | ALDH1A1 | -0.77 | 9.76E-04 | 8.52E-03 |
| Scara5 | SCARA5 | -0.77 | 2.34E-03 | 1.67E-02 |
| Nr3c2 | NR3C2 | -0.77 | 3.09E-03 | 2.09E-02 |
| Gstz1 | GSTZ1 | -0.76 | 2.73E-06 | 7.52E-05 |
| Zfp113 | ZNF3 | -0.76 | 6.77E-04 | 6.35E-03 |
| Prkdc | PRKDC | -0.76 | 8.09E-05 | 1.17E-03 |
| Irgm2 | IRGM | -0.76 | 8.62E-04 | 7.71E-03 |
| Adcy9 | ADCY9 | -0.76 | 3.01E-04 | 3.31E-03 |
| Adora2b | ADORA2B | -0.76 | 1.77E-04 | 2.17E-03 |
| Hid1 | HID1 | -0.76 | 3.34E-04 | 3.63E-03 |
| Ankrd6 | ANKRD6 | -0.75 | 7.55E-04 | 6.95E-03 |
| Zfp472 | NA | -0.75 | 2.24E-03 | 1.62E-02 |
| Grip1 | GRIP1 | -0.75 | 6.49E-03 | 3.67E-02 |
| Peo1 | C10orf2 | -0.75 | 6.08E-03 | 3.49E-02 |
| Tgfbr3 | TGFBR3 | -0.75 | 6.59E-03 | 3.71E-02 |
| Armcx3 | ARMCX3 | -0.75 | 4.60E-03 | 2.83E-02 |
| Gpd1 | GPD1 | -0.75 | 9.47E-03 | 4.90E-02 |
| Ccpg1os | C15orf65 | -0.75 | 9.72E-03 | 4.98E-02 |
| Zfp799 | ZNF878 | -0.75 | 1.44E-03 | 1.14E-02 |
| Tle1 | TLE1 | -0.74 | 2.19E-04 | 2.57E-03 |
| Casd1 | CASD1 | -0.74 | 3.26E-05 | 5.52E-04 |
| Slc23a2 | SLC23A2 | -0.74 | 1.70E-03 | 1.31E-02 |
| Fam118a | FAM118A | -0.74 | 6.83E-03 | 3.80E-02 |
| Zbed3 | ZBED3 | -0.74 | 1.35E-03 | 1.09E-02 |
| Hoxb2 | HOXB2 | -0.74 | 1.54E-04 | 1.96E-03 |
| Tmem176a | TMEM176A | -0.74 | 1.11E-04 | 1.52E-03 |
| Fbxo10 | FBXO10 | -0.74 | 4.64E-03 | 2.85E-02 |
| Mapk12 | MAPK12 | -0.73 | 1.37E-06 | 4.31E-05 |
| Hoxa5 | HOXA5 | -0.73 | 6.88E-03 | 3.82E-02 |
| Zfp119b | NA | -0.73 | 8.84E-03 | 4.65E-02 |
| Kitl | KITLG | -0.73 | 1.00E-04 | 1.38E-03 |
| Mettl23 | METTL23 | -0.73 | 3.42E-03 | 2.26E-02 |
| Mob3b | MOB3B | -0.73 | 2.31E-04 | 2.68E-03 |
| Agbl5 | AGBL5 | -0.73 | 7.65E-03 | 4.16E-02 |
| Atm | ATM | -0.73 | 2.33E-05 | 4.23E-04 |
| Selenbp2 | SELENBP1 | -0.73 | 1.04E-04 | 1.43E-03 |
| Sox6 | SOX6 | -0.72 | 6.83E-05 | 1.02E-03 |
| Lmf1 | LMF1 | -0.72 | 3.19E-04 | 3.49E-03 |
| Calcoco1 | CALCOCO1 | -0.72 | 1.66E-07 | 7.77E-06 |
| Ralgps1 | RALGPS1 | -0.72 | 4.94E-03 | 3.00E-02 |
| Paics | PAICS | -0.72 | 6.40E-07 | 2.29E-05 |
| Cntln | CNTLN | -0.72 | 3.99E-03 | 2.55E-02 |
| Tbc1d8b | TBC1D8B | -0.72 | 4.60E-04 | 4.69E-03 |
| Selenbp1 | SELENBP1 | -0.72 | 1.39E-04 | 1.80E-03 |
| Porcn | PORCN | -0.71 | 2.16E-04 | 2.55E-03 |
| Ctf1 | CTF1 | -0.71 | 1.49E-03 | 1.18E-02 |
| Sox21 | SOX21 | -0.71 | 3.23E-06 | 8.47E-05 |
| Tmem56 | TMEM56-RWDD3 | -0.71 | 8.34E-06 | 1.80E-04 |
| Rcor3 | RCOR3 | -0.71 | 9.72E-05 | 1.36E-03 |
| Dcaf6 | DCAF6 | -0.71 | 4.79E-07 | 1.85E-05 |
| Nfia | NFIA | -0.71 | 1.00E-04 | 1.38E-03 |
| Coq10a | COQ10A | -0.71 | 1.31E-03 | 1.06E-02 |
| Cdon | CDON | -0.71 | 1.65E-03 | 1.27E-02 |
| Gstm1 | NA | -0.70 | 4.02E-05 | 6.48E-04 |
| Spice1 | SPICE1 | -0.70 | 2.17E-03 | 1.58E-02 |
| Zfp952 | NA | -0.70 | 1.68E-03 | 1.29E-02 |
| Tbc1d19 | TBC1D19 | -0.70 | 4.27E-03 | 2.68E-02 |
| Tln2 | TLN2 | -0.70 | 5.56E-04 | 5.42E-03 |
| Agr2 | AGR2 | -0.70 | 3.37E-05 | 5.67E-04 |
| Cbx7 | CBX7 | -0.70 | 1.13E-03 | 9.59E-03 |
| Kdelr3 | KDELR3 | -0.70 | 1.42E-03 | 1.13E-02 |
| Pcyt1b | PCYT1B | -0.70 | 6.14E-06 | 1.41E-04 |
| Dtx4 | DTX4 | -0.70 | 5.24E-03 | 3.13E-02 |
| Pcnt | PCNT | -0.70 | 1.39E-03 | 1.11E-02 |
| Fut11 | FUT11 | -0.69 | 2.13E-04 | 2.52E-03 |
| Zfp704 | ZNF704 | -0.69 | 1.69E-03 | 1.30E-02 |
| Fam114a1 | FAM114A1 | -0.69 | 7.01E-05 | 1.04E-03 |
| Gnai1 | GNAI1 | -0.69 | 5.18E-05 | 8.07E-04 |
| AI464131 | KIAA1161 | -0.69 | 8.38E-03 | 4.46E-02 |
| Creb3l2 | CREB3L2 | -0.69 | 3.52E-05 | 5.84E-04 |
| Zfp606 | ZNF606 | -0.68 | 7.40E-03 | 4.04E-02 |
| Zscan12 | ZSCAN12 | -0.68 | 7.57E-03 | 4.13E-02 |
| Tuba1b | TUBA1B | -0.68 | 1.48E-06 | 4.57E-05 |
| Slc1a5 | SLC1A5 | -0.68 | 6.06E-07 | 2.20E-05 |
| Ppap2b | NA | -0.68 | 5.71E-03 | 3.35E-02 |
| Txlng | TXLNG | -0.68 | 8.94E-03 | 4.70E-02 |
| Atp8a1 | ATP8A1 | -0.68 | 1.03E-04 | 1.41E-03 |
| St8sia6 | ST8SIA6 | -0.68 | 3.12E-03 | 2.11E-02 |
| 6-Sep | 6-Sep | -0.68 | 8.57E-03 | 4.54E-02 |
| Adrbk2 | GRK3 | -0.68 | 1.55E-04 | 1.97E-03 |
| Nt5dc3 | NT5DC3 | -0.68 | 3.22E-03 | 2.16E-02 |
| Ago4 | AGO4 | -0.68 | 1.68E-04 | 2.09E-03 |
| Pccb | PCCB | -0.68 | 6.76E-08 | 3.65E-06 |
| Zfp316 | ZNF316 | -0.68 | 6.35E-03 | 3.61E-02 |
| 4930452B06Rik | C3orf67 | -0.68 | 4.02E-03 | 2.55E-02 |
| Fam208a | FAM208A | -0.68 | 1.07E-05 | 2.20E-04 |
| Adprm | ADPRM | -0.67 | 1.94E-04 | 2.33E-03 |
| Cdk5rap2 | CDK5RAP2 | -0.67 | 1.67E-03 | 1.28E-02 |
| Eif4a2 | EIF4A2 | -0.67 | 2.40E-05 | 4.32E-04 |
| Wdr72 | WDR72 | -0.67 | 4.31E-06 | 1.07E-04 |
| Zmym4 | ZMYM4 | -0.67 | 4.58E-05 | 7.23E-04 |
| Vmac | VMAC | -0.67 | 1.14E-04 | 1.54E-03 |
| Trit1 | TRIT1 | -0.67 | 3.59E-03 | 2.34E-02 |
| Wfdc2 | WFDC2 | -0.67 | 1.17E-03 | 9.84E-03 |
| Kmt2a | KMT2A | -0.67 | 1.33E-04 | 1.75E-03 |
| Zfp608 | ZNF608 | -0.67 | 5.21E-04 | 5.15E-03 |
| Wbscr27 | WBSCR27 | -0.67 | 2.74E-03 | 1.90E-02 |
| Smo | SMO | -0.67 | 1.01E-06 | 3.38E-05 |
| Alg6 | ALG6 | -0.67 | 3.69E-03 | 2.40E-02 |
| Tgm2 | TGM2 | -0.67 | 9.03E-03 | 4.72E-02 |
| Mgat3 | MGAT3 | -0.67 | 1.78E-05 | 3.36E-04 |
| Gpx4 | GPX4 | -0.66 | 3.85E-06 | 9.74E-05 |
| Rbbp7 | RBBP7 | -0.66 | 7.05E-06 | 1.57E-04 |
| Nicn1 | NICN1 | -0.66 | 1.23E-03 | 1.02E-02 |
| Atp1b1 | ATP1B1 | -0.66 | 7.81E-04 | 7.16E-03 |
| Sod1 | SOD1 | -0.66 | 5.62E-06 | 1.31E-04 |
| Tmem223 | NA | -0.66 | 4.10E-04 | 4.29E-03 |
| Gstm2 | NA | -0.66 | 6.48E-05 | 9.75E-04 |
| BC030870 | LINC01207 | -0.66 | 1.72E-03 | 1.32E-02 |
| Map1lc3a | MAP1LC3A | -0.66 | 4.69E-06 | 1.14E-04 |
| Gpr125 | NA | -0.65 | 1.81E-03 | 1.36E-02 |
| Arsk | ARSK | -0.65 | 1.81E-03 | 1.36E-02 |
| Tmem64 | TMEM64 | -0.65 | 2.30E-04 | 2.67E-03 |
| Zfp317 | ZNF317 | -0.65 | 6.22E-04 | 5.95E-03 |
| Gpr116 | NA | -0.65 | 5.68E-03 | 3.33E-02 |
| H2-Ke6 | HSD17B8 | -0.65 | 7.19E-04 | 6.68E-03 |
| Mccc2 | MCCC2 | -0.65 | 3.31E-03 | 2.21E-02 |
| Eya1 | EYA1 | -0.65 | 8.47E-04 | 7.64E-03 |
| Dmrt2 | DMRT2 | -0.65 | 6.08E-05 | 9.25E-04 |
| Neo1 | NEO1 | -0.65 | 2.20E-06 | 6.26E-05 |
| Sms | SMS | -0.65 | 1.67E-04 | 2.08E-03 |
| Galm | GALM | -0.65 | 1.64E-04 | 2.05E-03 |
| Paqr4 | PAQR4 | -0.65 | 9.86E-05 | 1.37E-03 |
| Cxxc5 | CXXC5 | -0.64 | 3.39E-04 | 3.67E-03 |
| Mfsd4 | NA | -0.64 | 1.21E-03 | 1.01E-02 |
| Fzd3 | FZD3 | -0.64 | 1.10E-03 | 9.37E-03 |
| Bphl | BPHL | -0.64 | 4.92E-04 | 4.94E-03 |
| Acsl1 | ACSL1 | -0.64 | 1.74E-06 | 5.21E-05 |
| Zfp251 | ZNF251 | -0.64 | 7.21E-03 | 3.96E-02 |
| Gpr56 | NA | -0.64 | 8.36E-06 | 1.81E-04 |
| Adi1 | ADI1 | -0.64 | 3.55E-04 | 3.81E-03 |
| Coq9 | COQ9 | -0.64 | 8.74E-04 | 7.78E-03 |
| Dynll2 | DYNLL2 | -0.64 | 4.19E-06 | 1.04E-04 |
| Zbtb4 | ZBTB4 | -0.64 | 8.69E-05 | 1.25E-03 |
| Ifi27 | IFI27 | -0.64 | 1.03E-05 | 2.15E-04 |
| Gcdh | GCDH | -0.64 | 3.92E-04 | 4.13E-03 |
| Dhrs11 | DHRS11 | -0.63 | 4.80E-03 | 2.92E-02 |
| Ephb2 | EPHB2 | -0.63 | 3.30E-03 | 2.20E-02 |
| Sepp1 | SEPP1 | -0.63 | 3.02E-03 | 2.06E-02 |
| Tmem263 | TMEM263 | -0.63 | 2.52E-03 | 1.77E-02 |
| Nmi | NMI | -0.63 | 1.00E-03 | 8.70E-03 |
| B230118H07Rik | C11orf74 | -0.63 | 7.72E-03 | 4.19E-02 |
| Nqo2 | NQO2 | -0.63 | 8.62E-06 | 1.85E-04 |
| Rassf9 | RASSF9 | -0.63 | 1.47E-03 | 1.17E-02 |
| Lpar3 | LPAR3 | -0.63 | 4.66E-04 | 4.73E-03 |
| St6galnac2 | ST6GALNAC2 | -0.63 | 9.94E-05 | 1.38E-03 |
| Mccc1 | MCCC1 | -0.63 | 2.13E-03 | 1.55E-02 |
| Tspan12 | TSPAN12 | -0.63 | 4.35E-06 | 1.08E-04 |
| Cep83os | NA | -0.63 | 5.95E-03 | 3.44E-02 |
| Cdk14 | CDK14 | -0.63 | 1.98E-04 | 2.37E-03 |
| Sestd1 | SESTD1 | -0.63 | 8.13E-06 | 1.76E-04 |
| Fzd7 | FZD7 | -0.63 | 8.29E-04 | 7.50E-03 |
| Zfp292 | ZNF292 | -0.62 | 1.71E-04 | 2.11E-03 |
| Fat2 | FAT2 | -0.62 | 2.56E-05 | 4.51E-04 |
| Pkp4 | PKP4 | -0.62 | 2.77E-06 | 7.58E-05 |
| Ggcx | GGCX | -0.62 | 4.91E-04 | 4.93E-03 |
| Tcf7l1 | TCF7L1 | -0.62 | 3.62E-03 | 2.36E-02 |
| Gpam | GPAM | -0.62 | 1.79E-03 | 1.35E-02 |
| Tanc2 | TANC2 | -0.62 | 3.11E-03 | 2.10E-02 |
| Hnrnph3 | HNRNPH3 | -0.62 | 9.12E-04 | 8.04E-03 |
| Sfrp1 | SFRP1 | -0.62 | 1.07E-03 | 9.20E-03 |
| Dhrs3 | DHRS3 | -0.62 | 3.22E-05 | 5.48E-04 |
| Vill | VILL | -0.62 | 2.54E-04 | 2.90E-03 |
| Ptprg | PTPRG | -0.62 | 6.60E-04 | 6.22E-03 |
| Fto | FTO | -0.61 | 6.49E-05 | 9.75E-04 |
| Artn | ARTN | -0.61 | 6.67E-03 | 3.74E-02 |
| Vegfb | VEGFB | -0.61 | 3.14E-03 | 2.12E-02 |
| Tbc1d4 | TBC1D4 | -0.61 | 4.27E-03 | 2.68E-02 |
| Pdf | PDF | -0.61 | 5.91E-04 | 5.69E-03 |
| Nf1 | NF1 | -0.61 | 8.12E-04 | 7.40E-03 |
| H2afy2 | H2AFY2 | -0.61 | 6.25E-03 | 3.56E-02 |
| Msi2 | MSI2 | -0.61 | 2.03E-03 | 1.49E-02 |
| Unc119 | UNC119 | -0.61 | 9.34E-03 | 4.85E-02 |
| Fgfr2 | FGFR2 | -0.61 | 4.82E-06 | 1.16E-04 |
| Alox12e | NA | -0.61 | 7.32E-03 | 4.01E-02 |
| Dapk2 | DAPK2 | -0.60 | 1.11E-03 | 9.47E-03 |
| Habp4 | HABP4 | -0.60 | 2.78E-03 | 1.92E-02 |
| Bach2 | BACH2 | -0.60 | 6.21E-03 | 3.55E-02 |
| Ltbp1 | LTBP1 | -0.60 | 2.07E-03 | 1.52E-02 |
| Gtf2i | GTF2I | -0.60 | 6.78E-06 | 1.53E-04 |
| Fyco1 | FYCO1 | -0.60 | 4.39E-06 | 1.08E-04 |
| Arhgap42 | ARHGAP42 | -0.59 | 4.34E-03 | 2.71E-02 |
| Ctps2 | CTPS2 | -0.59 | 2.81E-04 | 3.13E-03 |
| Slc22a5 | SLC22A5 | -0.59 | 1.22E-04 | 1.63E-03 |
| Abcd1 | ABCD1 | -0.59 | 5.84E-03 | 3.40E-02 |
| Prob1 | PROB1 | -0.59 | 2.28E-03 | 1.64E-02 |
| Chd6 | CHD6 | -0.59 | 2.77E-04 | 3.09E-03 |
| Sphk1 | SPHK1 | -0.59 | 7.45E-03 | 4.07E-02 |
| Adcy6 | ADCY6 | -0.59 | 4.27E-04 | 4.42E-03 |
| Ifit1 | IFIT1B | -0.59 | 3.35E-04 | 3.64E-03 |
| Herc3 | HERC3 | -0.59 | 4.11E-03 | 2.60E-02 |
| Msra | MSRA | -0.59 | 2.83E-03 | 1.94E-02 |
| Slc12a7 | SLC12A7 | -0.58 | 3.55E-03 | 2.32E-02 |
| Hibch | HIBCH | -0.58 | 2.13E-04 | 2.52E-03 |
| F3 | F3 | -0.58 | 1.31E-03 | 1.07E-02 |
| Idh2 | IDH2 | -0.58 | 2.11E-05 | 3.89E-04 |
| H2-Aa | HLA-DQA2 | -0.58 | 5.63E-03 | 3.31E-02 |
| Agfg2 | AGFG2 | -0.58 | 3.73E-03 | 2.41E-02 |
| Lrig1 | LRIG1 | -0.58 | 3.12E-04 | 3.43E-03 |
| Thtpa | THTPA | -0.58 | 9.01E-03 | 4.71E-02 |
| Limd2 | LIMD2 | -0.58 | 2.36E-03 | 1.68E-02 |
| Vps13c | VPS13C | -0.58 | 4.06E-03 | 2.58E-02 |
| Cep63 | CEP63 | -0.58 | 1.45E-03 | 1.15E-02 |
| Cstf2t | CSTF2T | -0.58 | 6.50E-04 | 6.16E-03 |
| Zcchc14 | ZCCHC14 | -0.58 | 1.54E-04 | 1.95E-03 |
| Arl4a | ARL4A | -0.58 | 5.78E-04 | 5.60E-03 |
| 0610009O20Rik | KIAA0141 | -0.58 | 6.30E-04 | 6.00E-03 |
| Rdm1 | RDM1 | -0.57 | 5.63E-03 | 3.31E-02 |
| Cln6 | CLN6 | -0.57 | 4.00E-04 | 4.20E-03 |
| D3Ertd254e | NA | -0.57 | 5.07E-03 | 3.05E-02 |
| Cd74 | CD74 | -0.57 | 5.51E-03 | 3.25E-02 |
| Cblb | CBLB | -0.57 | 4.96E-04 | 4.97E-03 |
| Clybl | CLYBL | -0.57 | 6.80E-03 | 3.79E-02 |
| Atp10a | ATP10A | -0.57 | 1.30E-03 | 1.06E-02 |
| Pros1 | PROS1 | -0.57 | 5.76E-05 | 8.86E-04 |
| Foxa1 | FOXA1 | -0.57 | 1.67E-05 | 3.19E-04 |
| Rasef | RASEF | -0.57 | 6.65E-03 | 3.74E-02 |
| Dguok | DGUOK | -0.57 | 1.75E-03 | 1.33E-02 |
| Trim44 | TRIM44 | -0.57 | 2.38E-04 | 2.75E-03 |
| Yipf6 | YIPF6 | -0.57 | 3.16E-05 | 5.41E-04 |
| Pctp | PCTP | -0.57 | 4.66E-03 | 2.86E-02 |
| Atxn1 | ATXN1 | -0.57 | 3.49E-03 | 2.29E-02 |
| Amer1 | AMER1 | -0.57 | 5.55E-03 | 3.27E-02 |
| Il1r1 | IL1R1 | -0.57 | 9.42E-05 | 1.32E-03 |
| Maml2 | MAML2 | -0.56 | 3.89E-04 | 4.11E-03 |
| Abcc5 | ABCC5 | -0.56 | 5.73E-03 | 3.36E-02 |
| Irgm1 | IRGM | -0.56 | 3.17E-03 | 2.14E-02 |
| Dkk3 | DKK3 | -0.56 | 2.82E-03 | 1.94E-02 |
| Znrf3 | ZNRF3 | -0.56 | 3.23E-03 | 2.17E-02 |
| Nr2f2 | NR2F2 | -0.56 | 5.41E-05 | 8.37E-04 |
| Hsd17b11 | HSD17B11 | -0.56 | 3.93E-05 | 6.36E-04 |
| Osgep | OSGEP | -0.56 | 8.23E-04 | 7.45E-03 |
| Rhoq | RHOQ | -0.56 | 3.48E-04 | 3.75E-03 |
| Ccdc3 | CCDC3 | -0.56 | 4.28E-05 | 6.82E-04 |
| BC017158 | C16orf58 | -0.56 | 4.06E-03 | 2.58E-02 |
| Nedd4 | NEDD4 | -0.56 | 1.19E-04 | 1.60E-03 |
| Meis2 | MEIS2 | -0.56 | 3.47E-03 | 2.28E-02 |
| Marveld1 | MARVELD1 | -0.56 | 7.32E-04 | 6.78E-03 |
| Rev3l | REV3L | -0.56 | 1.69E-03 | 1.30E-02 |
| Vtcn1 | VTCN1 | -0.56 | 3.99E-04 | 4.20E-03 |
| Tspan13 | TSPAN13 | -0.56 | 3.89E-03 | 2.49E-02 |
| Cluap1 | CLUAP1 | -0.56 | 5.51E-03 | 3.25E-02 |
| Slc25a16 | SLC25A16 | -0.56 | 1.37E-04 | 1.79E-03 |
| Ptprk | PTPRK | -0.56 | 1.04E-05 | 2.17E-04 |
| Wbp5 | TCEAL9 | -0.55 | 2.19E-04 | 2.57E-03 |
| Btbd6 | BTBD6 | -0.55 | 6.66E-03 | 3.74E-02 |
| Apoa1bp | NAXE | -0.55 | 4.23E-05 | 6.77E-04 |
| Maged1 | MAGED1 | -0.55 | 1.86E-04 | 2.26E-03 |
| P2rx4 | P2RX4 | -0.55 | 8.60E-04 | 7.69E-03 |
| Gbas | GBAS | -0.55 | 6.98E-05 | 1.04E-03 |
| Gstt3 | NA | -0.55 | 9.78E-05 | 1.36E-03 |
| Clstn1 | CLSTN1 | -0.55 | 3.26E-04 | 3.56E-03 |
| Irx5 | IRX5 | -0.55 | 1.81E-03 | 1.36E-02 |
| Pald1 | PALD1 | -0.55 | 9.57E-03 | 4.93E-02 |
| Ubl4 | NA | -0.55 | 8.79E-04 | 7.82E-03 |
| Cd59a | CD59 | -0.55 | 4.72E-03 | 2.88E-02 |
| Afap1 | AFAP1 | -0.54 | 1.22E-04 | 1.63E-03 |
| Nipsnap1 | NIPSNAP1 | -0.54 | 1.61E-03 | 1.25E-02 |
| Smad5 | SMAD5 | -0.54 | 7.70E-05 | 1.12E-03 |
| Mllt6 | MLLT6 | -0.54 | 2.12E-03 | 1.55E-02 |
| Prps1l3 | PRPS1 | -0.54 | 5.34E-05 | 8.27E-04 |
| Dapk1 | DAPK1 | -0.54 | 2.14E-03 | 1.56E-02 |
| Dock8 | DOCK8 | -0.54 | 1.68E-04 | 2.09E-03 |
| Eci1 | ECI1 | -0.54 | 2.43E-04 | 2.80E-03 |
| Ahr | AHR | -0.54 | 4.49E-05 | 7.10E-04 |
| Gm14322 | NA | -0.54 | 3.44E-03 | 2.27E-02 |
| Six1 | SIX1 | -0.54 | 4.61E-04 | 4.69E-03 |
| Smim19 | SMIM19 | -0.54 | 1.16E-03 | 9.79E-03 |
| Cnnm3 | CNNM3 | -0.54 | 2.94E-03 | 2.01E-02 |
| Pde4dip | PDE4DIP | -0.54 | 3.93E-04 | 4.14E-03 |
| Atraid | ATRAID | -0.54 | 2.81E-05 | 4.87E-04 |
| Bbx | BBX | -0.54 | 4.48E-03 | 2.77E-02 |
| Zfp467 | ZNF467 | -0.54 | 7.84E-03 | 4.24E-02 |
| Ino80 | INO80 | -0.54 | 1.30E-03 | 1.06E-02 |
| Zfp422 | ZNF22 | -0.54 | 9.58E-04 | 8.39E-03 |
| Cspp1 | CSPP1 | -0.54 | 7.33E-03 | 4.01E-02 |
| Asrgl1 | ASRGL1 | -0.54 | 7.63E-03 | 4.15E-02 |
| Rab2b | RAB2B | -0.53 | 3.96E-03 | 2.53E-02 |
| Ccnt2 | CCNT2 | -0.53 | 6.83E-04 | 6.38E-03 |
| Zfp275 | ZNF275 | -0.53 | 1.23E-03 | 1.02E-02 |
| Ddx60 | DDX60 | -0.53 | 1.41E-03 | 1.13E-02 |
| Cav1 | CAV1 | -0.53 | 4.80E-03 | 2.92E-02 |
| Nbea | NBEA | -0.53 | 1.12E-03 | 9.51E-03 |
| Pgrmc2 | PGRMC2 | -0.53 | 1.49E-04 | 1.91E-03 |
| Bckdhb | BCKDHB | -0.53 | 3.19E-03 | 2.15E-02 |
| Chchd10 | CHCHD10 | -0.53 | 3.71E-03 | 2.41E-02 |
| Ctso | CTSO | -0.53 | 3.74E-03 | 2.42E-02 |
| S100a1 | S100A1 | -0.53 | 9.65E-04 | 8.44E-03 |
| Farp1 | FARP1 | -0.53 | 5.13E-03 | 3.08E-02 |
| Syne2 | SYNE2 | -0.52 | 3.17E-03 | 2.14E-02 |
| E2f6 | E2F6 | -0.52 | 5.08E-03 | 3.06E-02 |
| C2cd2 | C2CD2 | -0.52 | 6.19E-05 | 9.37E-04 |
| Prkaa2 | PRKAA2 | -0.52 | 1.73E-03 | 1.32E-02 |
| Tns3 | TNS3 | -0.52 | 6.16E-04 | 5.90E-03 |
| P4ha2 | P4HA2 | -0.52 | 2.85E-03 | 1.95E-02 |
| Asph | ASPH | -0.52 | 1.40E-03 | 1.12E-02 |
| Boc | BOC | -0.51 | 2.56E-03 | 1.80E-02 |
| Aldh3a2 | ALDH3A2 | -0.51 | 2.98E-05 | 5.14E-04 |
| Mtus1 | MTUS1 | -0.51 | 4.15E-04 | 4.32E-03 |
| Zbtb14 | ZBTB14 | -0.51 | 3.99E-03 | 2.55E-02 |
| Mpv17 | MPV17 | -0.51 | 1.48E-03 | 1.17E-02 |
| Ssbp2 | SSBP2 | -0.51 | 1.37E-04 | 1.79E-03 |
| Nr2c2 | NR2C2 | -0.51 | 4.15E-04 | 4.32E-03 |
| Dip2c | DIP2C | -0.51 | 7.93E-03 | 4.28E-02 |
| Vipr1 | VIPR1 | -0.51 | 7.95E-04 | 7.27E-03 |
| Ltbp4 | LTBP4 | -0.51 | 3.43E-03 | 2.26E-02 |
| Slc35g1 | SLC35G1 | -0.50 | 7.74E-03 | 4.19E-02 |
| Zfp26 | NA | -0.50 | 1.47E-03 | 1.16E-02 |
| 1810030O07Rik | CXorf38 | -0.50 | 2.74E-04 | 3.06E-03 |
| Tmem106c | TMEM106C | -0.50 | 8.09E-04 | 7.38E-03 |
| 1110008L16Rik | KIAA0391 | -0.50 | 7.01E-03 | 3.87E-02 |
| Rabep1 | RABEP1 | -0.50 | 2.99E-03 | 2.04E-02 |
| R3hdm1 | R3HDM1 | -0.50 | 1.58E-03 | 1.23E-02 |
| Lap3 | LAP3 | -0.50 | 1.96E-04 | 2.35E-03 |
| Pgrmc1 | PGRMC1 | -0.50 | 2.04E-03 | 1.50E-02 |
| St5 | ST5 | -0.50 | 3.90E-04 | 4.11E-03 |
| Add3 | ADD3 | -0.50 | 1.05E-04 | 1.45E-03 |
| Sel1l3 | SEL1L3 | -0.50 | 1.36E-03 | 1.10E-02 |
| Rab3ip | NA | -0.50 | 4.38E-04 | 4.51E-03 |
| Fbxl17 | FBXL17 | -0.49 | 1.73E-03 | 1.32E-02 |
| Ivd | IVD | -0.49 | 1.48E-03 | 1.17E-02 |
| Birc2 | BIRC2 | -0.49 | 1.15E-03 | 9.74E-03 |
| Pbx1 | PBX1 | -0.49 | 1.50E-04 | 1.92E-03 |
| Urod | UROD | -0.49 | 1.48E-03 | 1.17E-02 |
| Ptp4a1 | PTP4A1 | -0.49 | 6.29E-04 | 5.99E-03 |
| Mrpl24 | MRPL24 | -0.49 | 5.27E-04 | 5.19E-03 |
| Tbc1d5 | TBC1D5 | -0.49 | 1.65E-03 | 1.28E-02 |
| Ptprs | PTPRS | -0.49 | 9.52E-03 | 4.91E-02 |
| Cox20 | COX20 | -0.49 | 3.51E-03 | 2.30E-02 |
| Sap18 | SAP18 | -0.49 | 1.76E-03 | 1.33E-02 |
| 2900097C17Rik | NA | -0.49 | 2.38E-04 | 2.75E-03 |
| Ston1 | STON1 | -0.49 | 6.52E-03 | 3.68E-02 |
| D230025D16Rik | C16orf70 | -0.49 | 6.58E-04 | 6.21E-03 |
| Pde5a | PDE5A | -0.49 | 8.88E-04 | 7.88E-03 |
| Rgmb | RGMB | -0.49 | 4.87E-03 | 2.96E-02 |
| Slc39a8 | SLC39A8 | -0.49 | 8.51E-04 | 7.65E-03 |
| Vps13b | VPS13B | -0.49 | 1.74E-03 | 1.33E-02 |
| Hibadh | HIBADH | -0.48 | 6.93E-04 | 6.47E-03 |
| Rac3 | RAC3 | -0.48 | 1.79E-03 | 1.35E-02 |
| Kiz | KIZ | -0.48 | 2.38E-03 | 1.70E-02 |
| Herc1 | HERC1 | -0.48 | 2.33E-03 | 1.67E-02 |
| Gstm4 | GSTM4 | -0.48 | 6.24E-03 | 3.56E-02 |
| Cyp4v3 | CYP4V2 | -0.48 | 3.45E-03 | 2.27E-02 |
| Phyh | PHYH | -0.48 | 1.45E-03 | 1.15E-02 |
| Rras | RRAS | -0.48 | 3.55E-03 | 2.32E-02 |
| Zfp397 | ZNF397 | -0.48 | 3.35E-03 | 2.23E-02 |
| Abcc3 | ABCC3 | -0.48 | 1.81E-03 | 1.36E-02 |
| Nr2c1 | NR2C1 | -0.48 | 8.47E-04 | 7.64E-03 |
| Sox2 | SOX2 | -0.48 | 4.91E-04 | 4.93E-03 |
| Lpp | LPP | -0.48 | 2.76E-04 | 3.09E-03 |
| Ppa2 | PPA2 | -0.48 | 1.37E-03 | 1.11E-02 |
| Tnrc6a | TNRC6A | -0.48 | 3.56E-03 | 2.32E-02 |
| Insr | INSR | -0.48 | 8.62E-04 | 7.71E-03 |
| Six2 | SIX2 | -0.48 | 6.22E-03 | 3.56E-02 |
| Pla2g16 | PLA2G16 | -0.48 | 7.21E-04 | 6.69E-03 |
| Zfp146 | ZNF146 | -0.48 | 9.15E-03 | 4.78E-02 |
| Zfp629 | ZNF629 | -0.47 | 9.71E-03 | 4.98E-02 |
| Jmjd8 | JMJD8 | -0.47 | 1.35E-03 | 1.09E-02 |
| Ankmy2 | ANKMY2 | -0.47 | 8.51E-04 | 7.65E-03 |
| Cdc23 | CDC23 | -0.47 | 8.51E-03 | 4.51E-02 |
| Rnf13 | RNF13 | -0.47 | 1.25E-03 | 1.03E-02 |
| Amz2 | AMZ2 | -0.47 | 2.04E-03 | 1.50E-02 |
| Mgat4b | MGAT4B | -0.47 | 1.72E-03 | 1.32E-02 |
| Pde7a | PDE7A | -0.47 | 2.74E-03 | 1.90E-02 |
| Sh3bgrl | SH3BGRL | -0.47 | 1.60E-03 | 1.24E-02 |
| Il13ra1 | IL13RA1 | -0.47 | 2.87E-03 | 1.97E-02 |
| Nisch | NISCH | -0.47 | 2.31E-03 | 1.66E-02 |
| Mfge8 | MFGE8 | -0.46 | 1.84E-03 | 1.38E-02 |
| Gsta3 | GSTA3 | -0.46 | 1.10E-03 | 9.37E-03 |
| Birc6 | BIRC6 | -0.46 | 2.04E-03 | 1.50E-02 |
| Adh1 | ADH1C | -0.46 | 2.54E-03 | 1.79E-02 |
| Anxa6 | ANXA6 | -0.46 | 2.88E-04 | 3.20E-03 |
| Abcc1 | NA | -0.46 | 8.93E-04 | 7.90E-03 |
| Dpyd | DPYD | -0.46 | 4.17E-03 | 2.63E-02 |
| Tgoln1 | TGOLN2 | -0.46 | 1.89E-03 | 1.41E-02 |
| Acsl3 | ACSL3 | -0.46 | 4.74E-03 | 2.89E-02 |
| Tmem260 | TMEM260 | -0.46 | 8.64E-03 | 4.57E-02 |
| Uqcc1 | UQCC1 | -0.46 | 4.56E-03 | 2.81E-02 |
| Cnpy2 | CNPY2 | -0.46 | 8.16E-04 | 7.42E-03 |
| Echs1 | ECHS1 | -0.46 | 2.63E-04 | 2.97E-03 |
| Gm14403 | NA | -0.46 | 2.20E-03 | 1.60E-02 |
| Stard3nl | STARD3NL | -0.46 | 2.96E-03 | 2.02E-02 |
| Oxsm | OXSM | -0.45 | 7.61E-03 | 4.14E-02 |
| Enox2 | ENOX2 | -0.45 | 8.34E-03 | 4.44E-02 |
| Tm7sf3 | TM7SF3 | -0.45 | 2.66E-03 | 1.85E-02 |
| Lrpprc | LRPPRC | -0.45 | 4.66E-03 | 2.86E-02 |
| Vav3 | VAV3 | -0.45 | 1.94E-03 | 1.44E-02 |
| Carkd | NAXD | -0.45 | 1.28E-03 | 1.05E-02 |
| Zfp503 | ZNF503 | -0.45 | 6.17E-03 | 3.53E-02 |
| St7 | ST7 | -0.45 | 5.49E-03 | 3.25E-02 |
| Trim37 | TRIM37 | -0.45 | 7.39E-03 | 4.04E-02 |
| Kmt2c | KMT2C | -0.45 | 4.84E-03 | 2.95E-02 |
| Cdo1 | CDO1 | -0.45 | 8.89E-03 | 4.67E-02 |
| Fkbp9 | FKBP9 | -0.45 | 3.69E-04 | 3.94E-03 |
| Ormdl3 | ORMDL3 | -0.45 | 2.97E-03 | 2.03E-02 |
| Zfp106 | ZNF106 | -0.45 | 2.67E-03 | 1.86E-02 |
| Ndufaf7 | NDUFAF7 | -0.45 | 5.01E-03 | 3.03E-02 |
| Eif3f | NA | -0.45 | 1.43E-03 | 1.14E-02 |
| Pacs1 | PACS1 | -0.44 | 6.44E-03 | 3.65E-02 |
| Nfix | NFIX | -0.44 | 3.72E-03 | 2.41E-02 |
| Cd164 | CD164 | -0.44 | 5.81E-03 | 3.39E-02 |
| Lgr4 | LGR4 | -0.44 | 1.69E-03 | 1.30E-02 |
| Casp2 | CASP2 | -0.44 | 2.34E-03 | 1.67E-02 |
| Aaed1 | AAED1 | -0.44 | 5.55E-03 | 3.27E-02 |
| Ubr1 | UBR1 | -0.44 | 4.04E-03 | 2.56E-02 |
| Plekha5 | PLEKHA5 | -0.44 | 5.24E-03 | 3.13E-02 |
| Lclat1 | LCLAT1 | -0.44 | 6.00E-03 | 3.46E-02 |
| Cyb5 | NA | -0.44 | 3.55E-03 | 2.32E-02 |
| Heatr5a | HEATR5A | -0.44 | 1.72E-03 | 1.32E-02 |
| Smarcal1 | SMARCAL1 | -0.44 | 9.47E-03 | 4.90E-02 |
| Tm9sf3 | TM9SF3 | -0.44 | 5.28E-03 | 3.15E-02 |
| Rdx | RDX | -0.44 | 9.57E-04 | 8.38E-03 |
| Zscan26 | ZSCAN26 | -0.43 | 4.46E-03 | 2.76E-02 |
| Misp | MISP | -0.43 | 7.68E-03 | 4.17E-02 |
| Snx19 | SNX19 | -0.43 | 5.13E-03 | 3.08E-02 |
| Sema3c | SEMA3C | -0.43 | 8.16E-03 | 4.36E-02 |
| Ddx17 | DDX17 | -0.43 | 2.80E-03 | 1.93E-02 |
| Cers5 | CERS5 | -0.43 | 6.72E-03 | 3.76E-02 |
| Arsb | ARSB | -0.43 | 5.04E-03 | 3.04E-02 |
| Paip1 | PAIP1 | -0.43 | 1.14E-03 | 9.61E-03 |
| Cep350 | CEP350 | -0.43 | 5.16E-03 | 3.09E-02 |
| Sfxn3 | SFXN3 | -0.43 | 3.52E-03 | 2.31E-02 |
| Vwa8 | VWA8 | -0.42 | 3.25E-03 | 2.18E-02 |
| Hes1 | HES1 | -0.42 | 1.77E-03 | 1.34E-02 |
| Phc3 | PHC3 | -0.42 | 3.36E-03 | 2.23E-02 |
| Entpd4 | ENTPD4 | -0.42 | 4.32E-03 | 2.70E-02 |
| Utrn | UTRN | -0.42 | 2.07E-03 | 1.52E-02 |
| Ppic | PPIC | -0.42 | 4.49E-03 | 2.78E-02 |
| Rae1 | RAE1 | -0.42 | 5.34E-03 | 3.17E-02 |
| Oxa1l | OXA1L | -0.42 | 7.46E-03 | 4.07E-02 |
| Rcbtb2 | RCBTB2 | -0.41 | 6.03E-03 | 3.47E-02 |
| Pon3 | PON3 | -0.41 | 2.05E-03 | 1.50E-02 |
| Nupr1 | NUPR1 | -0.41 | 8.10E-03 | 4.34E-02 |
| Rnf130 | RNF130 | -0.41 | 4.23E-03 | 2.66E-02 |
| Irf2bpl | IRF2BPL | -0.41 | 4.38E-03 | 2.73E-02 |
| Irf9 | IRF9 | -0.41 | 1.98E-03 | 1.46E-02 |
| Psme1 | PSME1 | -0.41 | 9.84E-04 | 8.57E-03 |
| Pcbp2 | PCBP2 | -0.41 | 1.97E-03 | 1.46E-02 |
| Prkacb | PRKACB | -0.41 | 6.06E-03 | 3.48E-02 |
| Ndrg3 | NDRG3 | -0.41 | 2.38E-03 | 1.70E-02 |
| Brd8 | BRD8 | -0.41 | 7.73E-03 | 4.19E-02 |
| Mtfr1l | MTFR1L | -0.41 | 3.80E-03 | 2.44E-02 |
| Zfp871 | ZNF878 | -0.41 | 4.77E-03 | 2.91E-02 |
| Mkl2 | MKL2 | -0.41 | 6.91E-03 | 3.83E-02 |
| Zfp7 | ZNF7 | -0.41 | 3.47E-03 | 2.28E-02 |
| Vhl | VHLL | -0.41 | 5.88E-03 | 3.41E-02 |
| Ivns1abp | IVNS1ABP | -0.41 | 3.50E-03 | 2.30E-02 |
| Erap1 | ERAP1 | -0.41 | 2.22E-03 | 1.60E-02 |
| Ikbkg | IKBKG | -0.40 | 2.25E-03 | 1.62E-02 |
| H2afv | H2AFV | -0.40 | 8.88E-03 | 4.67E-02 |
| Tmem38b | TMEM38B | -0.40 | 6.71E-03 | 3.76E-02 |
| Mrpl39 | MRPL39 | -0.40 | 4.42E-03 | 2.75E-02 |
| Lmo4 | LMO4 | -0.40 | 6.16E-03 | 3.53E-02 |
| Smarca2 | SMARCA2 | -0.40 | 6.84E-03 | 3.80E-02 |
| Pmpcb | PMPCB | -0.40 | 9.68E-03 | 4.97E-02 |
| Josd1 | JOSD1 | -0.40 | 3.10E-03 | 2.10E-02 |
| Sav1 | SAV1 | -0.40 | 8.87E-03 | 4.67E-02 |
| Ago1 | AGO1 | -0.40 | 2.00E-03 | 1.48E-02 |
| Zc3h7b | ZC3H7B | -0.40 | 4.84E-03 | 2.95E-02 |
| Zfp869 | ZNF101 | -0.40 | 6.90E-03 | 3.83E-02 |
| Cab39l | CAB39L | -0.40 | 1.25E-03 | 1.03E-02 |
| Hsd17b4 | HSD17B4 | -0.39 | 2.04E-03 | 1.50E-02 |
| Lancl1 | LANCL1 | -0.39 | 9.16E-03 | 4.78E-02 |
| Sin3a | SIN3A | -0.39 | 3.88E-03 | 2.49E-02 |
| 1810026J23Rik | C19orf52 | -0.39 | 3.50E-03 | 2.30E-02 |
| Met | MET | -0.39 | 6.00E-03 | 3.46E-02 |
| Ep400 | EP400 | -0.39 | 5.99E-03 | 3.45E-02 |
| P2rx7 | P2RX7 | -0.39 | 8.61E-03 | 4.55E-02 |
| Mylip | MYLIP | -0.39 | 9.58E-03 | 4.93E-02 |
| Dnajc10 | DNAJC10 | -0.39 | 4.51E-03 | 2.79E-02 |
| 6820431F20Rik | NA | -0.39 | 5.50E-03 | 3.25E-02 |
| Zfp609 | ZNF609 | -0.38 | 8.69E-03 | 4.59E-02 |
| Aplp2 | APLP2 | -0.38 | 7.03E-03 | 3.88E-02 |
| Coa3 | COA3 | -0.38 | 8.96E-03 | 4.70E-02 |
| Zbtb44 | ZBTB44 | -0.38 | 6.44E-03 | 3.65E-02 |
| Pir | PIR | -0.37 | 6.83E-03 | 3.80E-02 |
| Zkscan1 | ZKSCAN1 | -0.37 | 8.92E-03 | 4.69E-02 |
| Ubr4 | UBR4 | -0.37 | 6.40E-03 | 3.63E-02 |
| Snrnp200 | SNRNP200 | -0.37 | 3.25E-03 | 2.18E-02 |
| Erbb2 | ERBB2 | -0.37 | 5.20E-03 | 3.11E-02 |
| Phkb | PHKB | -0.37 | 5.56E-03 | 3.28E-02 |
| Sdha | SDHA | -0.37 | 2.52E-03 | 1.77E-02 |
| Acadsb | ACADSB | -0.37 | 8.40E-03 | 4.46E-02 |
| Man1b1 | MAN1B1 | -0.36 | 4.11E-03 | 2.60E-02 |
| Pdxdc1 | PDXDC1 | -0.36 | 6.17E-03 | 3.53E-02 |
| Lrp6 | LRP6 | -0.36 | 4.46E-03 | 2.77E-02 |
| Ilk | ILK | -0.36 | 5.79E-03 | 3.38E-02 |
| Mapk9 | MAPK9 | -0.36 | 9.68E-03 | 4.97E-02 |
| Idh3g | IDH3G | -0.36 | 8.59E-03 | 4.55E-02 |
| Sel1l | SEL1L | -0.35 | 7.85E-03 | 4.24E-02 |
| Bmpr1a | BMPR1A | -0.35 | 6.65E-03 | 3.74E-02 |
| Eif2a | EIF2A | -0.35 | 6.86E-03 | 3.81E-02 |
| Btf3l4 | BTF3L4 | -0.35 | 8.27E-03 | 4.41E-02 |
| Cdk19 | CDK19 | -0.34 | 8.70E-03 | 4.59E-02 |
| Dag1 | DAG1 | -0.34 | 9.52E-03 | 4.91E-02 |
| Arfgap3 | ARFGAP3 | -0.34 | 8.13E-03 | 4.35E-02 |
| Khdrbs1 | KHDRBS1 | -0.33 | 8.18E-03 | 4.37E-02 |
| Vdac3 | VDAC3 | -0.33 | 9.48E-03 | 4.90E-02 |
| Dcaf11 | DCAF11 | -0.32 | 6.30E-03 | 3.59E-02 |
| Tgfa | TGFA | 0.32 | 9.61E-03 | 4.94E-02 |
| Ppp3ca | PPP3CA | 0.33 | 6.72E-03 | 3.76E-02 |
| Dhx8 | DHX8 | 0.33 | 9.00E-03 | 4.71E-02 |
| Sh3d19 | SH3D19 | 0.33 | 9.54E-03 | 4.92E-02 |
| Map3k9 | MAP3K9 | 0.33 | 9.76E-03 | 4.99E-02 |
| Golga2 | GOLGA6L4 | 0.34 | 7.44E-03 | 4.07E-02 |
| Arpc4 | ARPC4 | 0.34 | 5.44E-03 | 3.22E-02 |
| Fbxo28 | FBXO28 | 0.34 | 5.73E-03 | 3.36E-02 |
| Zbtb43 | ZBTB43 | 0.35 | 9.42E-03 | 4.88E-02 |
| Stam2 | STAM2 | 0.35 | 6.24E-03 | 3.56E-02 |
| Nans | NANS | 0.35 | 6.44E-03 | 3.65E-02 |
| Itsn2 | ITSN2 | 0.35 | 7.63E-03 | 4.15E-02 |
| Atp6v1c1 | ATP6V1C1 | 0.35 | 4.13E-03 | 2.61E-02 |
| Gsk3a | GSK3A | 0.35 | 5.05E-03 | 3.04E-02 |
| Vps36 | VPS36 | 0.35 | 8.50E-03 | 4.51E-02 |
| Rab35 | RAB35 | 0.35 | 9.07E-03 | 4.74E-02 |
| Samd4b | SAMD4B | 0.35 | 9.31E-03 | 4.84E-02 |
| Ostf1 | OSTF1 | 0.36 | 8.15E-03 | 4.36E-02 |
| Zdhhc20 | ZDHHC20 | 0.36 | 9.70E-03 | 4.98E-02 |
| Cttn | CTTN | 0.36 | 8.67E-03 | 4.58E-02 |
| Ckmt1 | CKMT1A | 0.36 | 8.14E-03 | 4.35E-02 |
| Slk | SLK | 0.36 | 6.93E-03 | 3.84E-02 |
| Phactr4 | PHACTR4 | 0.36 | 5.28E-03 | 3.15E-02 |
| Ube2h | UBE2H | 0.36 | 7.96E-03 | 4.28E-02 |
| Ptgfrn | PTGFRN | 0.36 | 6.73E-03 | 3.76E-02 |
| Strn | STRN | 0.37 | 6.24E-03 | 3.56E-02 |
| Sh3bp4 | SH3BP4 | 0.37 | 7.90E-03 | 4.26E-02 |
| Smurf1 | SMURF1 | 0.37 | 7.23E-03 | 3.97E-02 |
| Abhd17c | ABHD17C | 0.37 | 7.95E-03 | 4.28E-02 |
| Mboat1 | MBOAT1 | 0.37 | 9.76E-03 | 4.99E-02 |
| Edem1 | EDEM1 | 0.37 | 4.31E-03 | 2.70E-02 |
| Map2k4 | MAP2K4 | 0.37 | 5.98E-03 | 3.45E-02 |
| Ube2v1 | UBE2V1 | 0.37 | 4.51E-03 | 2.78E-02 |
| Setd8 | NA | 0.37 | 2.81E-03 | 1.93E-02 |
| Rap2c | RAP2C | 0.37 | 9.47E-03 | 4.90E-02 |
| Cfl1 | CFL1 | 0.37 | 6.80E-03 | 3.79E-02 |
| Ppp1r15b | PPP1R15B | 0.38 | 4.36E-03 | 2.72E-02 |
| Tmod3 | TMOD3 | 0.38 | 9.33E-03 | 4.85E-02 |
| Phf23 | PHF23 | 0.38 | 4.51E-03 | 2.79E-02 |
| Camk1d | CAMK1D | 0.38 | 2.92E-03 | 2.00E-02 |
| Atp6v0b | ATP6V0B | 0.38 | 8.18E-03 | 4.37E-02 |
| Slc30a4 | SLC30A4 | 0.38 | 5.65E-03 | 3.32E-02 |
| Ranbp1 | RANBP1 | 0.38 | 3.76E-03 | 2.42E-02 |
| Map2k1 | MAP2K1 | 0.38 | 9.34E-03 | 4.85E-02 |
| Twf1 | TWF1 | 0.38 | 5.25E-03 | 3.13E-02 |
| Prnp | PRNP | 0.38 | 4.72E-03 | 2.88E-02 |
| 1810013L24Rik | C16orf72 | 0.38 | 5.92E-03 | 3.43E-02 |
| Acap2 | ACAP2 | 0.39 | 1.82E-03 | 1.36E-02 |
| Txndc17 | TXNDC17 | 0.39 | 6.36E-03 | 3.61E-02 |
| Fbxo38 | FBXO38 | 0.39 | 2.07E-03 | 1.52E-02 |
| Nhsl1 | NHSL1 | 0.39 | 5.48E-03 | 3.24E-02 |
| Dennd2c | DENND2C | 0.39 | 7.71E-03 | 4.19E-02 |
| Ptbp3 | PTBP3 | 0.39 | 6.35E-03 | 3.61E-02 |
| Nup50 | NUP50 | 0.39 | 8.57E-04 | 7.68E-03 |
| Atp11b | ATP11B | 0.39 | 8.77E-03 | 4.62E-02 |
| Zdhhc5 | ZDHHC5 | 0.39 | 6.49E-03 | 3.67E-02 |
| Jund | JUND | 0.40 | 4.93E-03 | 2.99E-02 |
| Marcks | MARCKS | 0.40 | 4.01E-03 | 2.55E-02 |
| Usp25 | USP25 | 0.40 | 5.78E-03 | 3.38E-02 |
| Irgq | IRGQ | 0.40 | 4.30E-03 | 2.69E-02 |
| Mprip | MPRIP | 0.40 | 4.57E-03 | 2.82E-02 |
| Prdx5 | PRDX5 | 0.40 | 5.68E-03 | 3.33E-02 |
| Ap1s1 | AP1S1 | 0.40 | 5.44E-03 | 3.22E-02 |
| Mark2 | MARK2 | 0.40 | 2.20E-03 | 1.59E-02 |
| Casp8 | CASP8 | 0.40 | 2.36E-03 | 1.69E-02 |
| Casp3 | CASP3 | 0.40 | 6.14E-03 | 3.52E-02 |
| Hsd17b12 | HSD17B12 | 0.40 | 6.15E-03 | 3.52E-02 |
| Gars | GARS | 0.40 | 3.13E-03 | 2.12E-02 |
| Kctd1 | KCTD1 | 0.40 | 4.68E-03 | 2.87E-02 |
| Ifitm3 | IFITM1 | 0.40 | 6.65E-03 | 3.74E-02 |
| Ube2r2 | UBE2R2 | 0.40 | 5.17E-03 | 3.10E-02 |
| Dgka | DGKA | 0.40 | 5.75E-03 | 3.37E-02 |
| Rac1 | RAC1 | 0.40 | 6.70E-03 | 3.76E-02 |
| Dnajb1 | DNAJB1 | 0.41 | 4.12E-03 | 2.61E-02 |
| Appbp2 | APPBP2 | 0.41 | 3.10E-03 | 2.10E-02 |
| Eif5a | EIF5A | 0.41 | 7.86E-03 | 4.25E-02 |
| Ehd4 | EHD4 | 0.41 | 3.29E-03 | 2.20E-02 |
| Usp6nl | USP6NL | 0.41 | 1.15E-03 | 9.74E-03 |
| Myl12a | MYL12A | 0.41 | 2.76E-03 | 1.91E-02 |
| Rangap1 | RANGAP1 | 0.41 | 9.75E-03 | 4.99E-02 |
| Vps53 | VPS53 | 0.41 | 2.56E-03 | 1.80E-02 |
| Cc2d1b | CC2D1B | 0.41 | 9.47E-03 | 4.90E-02 |
| Bcl10 | BCL10 | 0.41 | 3.56E-03 | 2.32E-02 |
| Nsf | NSF | 0.41 | 1.74E-03 | 1.33E-02 |
| Atp1b3 | ATP1B3 | 0.41 | 7.23E-03 | 3.97E-02 |
| Atp6v0e | ATP6V0E1 | 0.42 | 4.65E-03 | 2.85E-02 |
| Nebl | NA | 0.42 | 9.59E-03 | 4.93E-02 |
| Osbpl11 | OSBPL11 | 0.42 | 4.39E-03 | 2.73E-02 |
| Pa2g4 | PA2G4 | 0.42 | 3.86E-03 | 2.48E-02 |
| Rabgef1 | RABGEF1 | 0.42 | 1.40E-03 | 1.12E-02 |
| Nfkb2 | NFKB2 | 0.42 | 8.47E-03 | 4.50E-02 |
| Plin2 | PLIN2 | 0.42 | 4.15E-03 | 2.62E-02 |
| Myo1e | MYO1E | 0.42 | 1.52E-03 | 1.20E-02 |
| Ndufa11 | NDUFA11 | 0.42 | 8.26E-03 | 4.41E-02 |
| Pcgf3 | PCGF3 | 0.42 | 6.86E-04 | 6.41E-03 |
| Arpc3 | ARPC3 | 0.42 | 1.87E-03 | 1.40E-02 |
| Capn2 | CAPN2 | 0.42 | 3.37E-03 | 2.24E-02 |
| Itga6 | ITGA6 | 0.42 | 6.01E-03 | 3.46E-02 |
| Hn1 | HN1 | 0.42 | 5.80E-03 | 3.39E-02 |
| Tmem41a | TMEM41A | 0.42 | 8.15E-03 | 4.36E-02 |
| Tbcb | TBCB | 0.42 | 3.38E-03 | 2.24E-02 |
| Tfdp1 | TFDP1 | 0.43 | 1.02E-03 | 8.85E-03 |
| Ppp1r12a | PPP1R12A | 0.43 | 4.76E-03 | 2.90E-02 |
| Pgk1 | PGK1 | 0.43 | 3.21E-03 | 2.16E-02 |
| Rps29 | RPS29 | 0.43 | 8.83E-03 | 4.65E-02 |
| Kank1 | KANK1 | 0.43 | 3.92E-03 | 2.51E-02 |
| Ralb | RALB | 0.43 | 1.40E-03 | 1.12E-02 |
| Rgl2 | RGL2 | 0.43 | 9.56E-03 | 4.92E-02 |
| Rnf169 | RNF169 | 0.43 | 6.35E-03 | 3.61E-02 |
| Ddhd1 | DDHD1 | 0.43 | 5.82E-03 | 3.40E-02 |
| Trim25 | TRIM25 | 0.43 | 3.61E-03 | 2.35E-02 |
| Daam1 | DAAM1 | 0.43 | 4.20E-03 | 2.64E-02 |
| Zbtb7b | ZBTB7B | 0.43 | 1.52E-03 | 1.19E-02 |
| P2ry2 | P2RY2 | 0.43 | 7.90E-03 | 4.26E-02 |
| Zmiz2 | ZMIZ2 | 0.43 | 6.36E-03 | 3.61E-02 |
| Sh3pxd2a | SH3PXD2A | 0.43 | 1.76E-03 | 1.34E-02 |
| Ptpn1 | PTPN1 | 0.44 | 8.54E-04 | 7.67E-03 |
| Triobp | TRIOBP | 0.44 | 5.35E-03 | 3.18E-02 |
| Ankrd13a | ANKRD13A | 0.44 | 8.51E-04 | 7.65E-03 |
| Myo18a | MYO18A | 0.44 | 2.19E-03 | 1.59E-02 |
| Zfp46 | ZNF436 | 0.44 | 3.73E-03 | 2.41E-02 |
| Tom1 | TOM1 | 0.44 | 8.73E-03 | 4.61E-02 |
| Mfsd5 | MFSD5 | 0.44 | 6.45E-03 | 3.65E-02 |
| Urah | NA | 0.44 | 4.14E-03 | 2.61E-02 |
| Tmprss4 | TMPRSS4 | 0.44 | 9.73E-03 | 4.99E-02 |
| Tnfrsf1a | TNFRSF1A | 0.44 | 1.23E-03 | 1.02E-02 |
| Malt1 | MALT1 | 0.44 | 9.00E-03 | 4.71E-02 |
| Nudcd2 | NUDCD2 | 0.44 | 6.30E-03 | 3.59E-02 |
| Psd4 | PSD4 | 0.44 | 6.08E-03 | 3.49E-02 |
| Ube2m | UBE2M | 0.44 | 5.24E-04 | 5.18E-03 |
| Arid5b | ARID5B | 0.44 | 3.17E-03 | 2.14E-02 |
| Slc31a2 | SLC31A2 | 0.44 | 5.75E-03 | 3.37E-02 |
| Cdk8 | CDK8 | 0.44 | 1.24E-03 | 1.03E-02 |
| Cdc34 | CDC34 | 0.44 | 3.54E-03 | 2.32E-02 |
| Srf | SRF | 0.44 | 4.01E-03 | 2.55E-02 |
| Arhgap17 | ARHGAP17 | 0.45 | 5.41E-03 | 3.21E-02 |
| Sp110 | SP110 | 0.45 | 2.40E-03 | 1.70E-02 |
| Zfp36l2 | ZFP36L2 | 0.45 | 1.33E-03 | 1.08E-02 |
| Hprt | HPRT1 | 0.45 | 3.37E-03 | 2.23E-02 |
| Ablim1 | ABLIM1 | 0.45 | 3.80E-03 | 2.44E-02 |
| Uaca | UACA | 0.45 | 3.63E-03 | 2.36E-02 |
| Sec23a | SEC23A | 0.45 | 1.57E-03 | 1.23E-02 |
| Scap | SCAP | 0.45 | 5.98E-03 | 3.45E-02 |
| Tjp2 | TJP2 | 0.45 | 1.79E-03 | 1.35E-02 |
| Actr3 | ACTR3 | 0.45 | 2.00E-03 | 1.48E-02 |
| Btbd3 | BTBD3 | 0.45 | 6.56E-03 | 3.69E-02 |
| Ubap1 | UBAP1 | 0.45 | 1.31E-03 | 1.06E-02 |
| Hars | HARS | 0.45 | 1.24E-03 | 1.02E-02 |
| Zdhhc13 | ZDHHC13 | 0.45 | 5.35E-03 | 3.18E-02 |
| Liph | LIPH | 0.45 | 1.75E-03 | 1.33E-02 |
| Ankrd50 | NA | 0.45 | 4.60E-03 | 2.83E-02 |
| Fxr2 | FXR2 | 0.45 | 1.59E-03 | 1.24E-02 |
| Gsn | GSN | 0.46 | 9.10E-03 | 4.75E-02 |
| Tmem189 | TMEM189 | 0.46 | 6.61E-03 | 3.72E-02 |
| Fbxo34 | FBXO34 | 0.46 | 3.08E-03 | 2.09E-02 |
| Pfdn1 | PFDN1 | 0.46 | 1.45E-03 | 1.15E-02 |
| Mlf2 | MLF2 | 0.46 | 5.33E-04 | 5.24E-03 |
| Crot | CROT | 0.46 | 4.59E-03 | 2.83E-02 |
| Chic2 | CHIC2 | 0.46 | 7.10E-03 | 3.91E-02 |
| Abl2 | ABL2 | 0.46 | 1.84E-03 | 1.38E-02 |
| Ccdc12 | CCDC12 | 0.46 | 6.68E-03 | 3.75E-02 |
| Slc27a4 | SLC27A4 | 0.46 | 7.91E-03 | 4.27E-02 |
| Mrpl20 | MRPL20 | 0.46 | 9.45E-03 | 4.89E-02 |
| Ppp2r2a | PPP2R2A | 0.46 | 2.02E-03 | 1.49E-02 |
| Tuft1 | TUFT1 | 0.46 | 5.41E-03 | 3.21E-02 |
| Fgfbp1 | FGFBP1 | 0.46 | 6.30E-03 | 3.59E-02 |
| Ier5 | IER5 | 0.46 | 1.72E-03 | 1.32E-02 |
| N4bp1 | N4BP1 | 0.46 | 5.78E-04 | 5.60E-03 |
| Atp6v0a1 | ATP6V0A1 | 0.46 | 6.54E-04 | 6.19E-03 |
| Fgd6 | FGD6 | 0.47 | 1.58E-03 | 1.23E-02 |
| B4galt5 | B4GALT5 | 0.47 | 3.75E-03 | 2.42E-02 |
| Tuba1c | TUBA1C | 0.47 | 5.03E-03 | 3.04E-02 |
| Eif2s2 | EIF2S2 | 0.47 | 2.36E-03 | 1.69E-02 |
| Pvrl4 | NA | 0.47 | 5.24E-03 | 3.13E-02 |
| Hras | HRAS | 0.47 | 1.50E-03 | 1.18E-02 |
| Dhcr7 | DHCR7 | 0.47 | 3.40E-03 | 2.25E-02 |
| Pard6g | PARD6G | 0.47 | 8.50E-04 | 7.65E-03 |
| Hace1 | HACE1 | 0.47 | 2.02E-03 | 1.49E-02 |
| Sptbn2 | SPTBN2 | 0.47 | 2.02E-03 | 1.49E-02 |
| Capn1 | CAPN1 | 0.47 | 2.59E-03 | 1.81E-02 |
| Osbpl2 | OSBPL2 | 0.47 | 2.29E-03 | 1.64E-02 |
| Taok3 | TAOK3 | 0.47 | 7.34E-04 | 6.78E-03 |
| Lphn2 | NA | 0.47 | 9.26E-04 | 8.14E-03 |
| Cyth1 | CYTH1 | 0.47 | 1.78E-03 | 1.35E-02 |
| Notch1 | NOTCH1 | 0.47 | 7.08E-03 | 3.90E-02 |
| Car13 | CA13 | 0.47 | 1.75E-03 | 1.33E-02 |
| Tomm40 | TOMM40 | 0.48 | 7.77E-03 | 4.21E-02 |
| Wdr26 | WDR26 | 0.48 | 3.03E-03 | 2.06E-02 |
| Tmtc3 | TMTC3 | 0.48 | 3.72E-04 | 3.96E-03 |
| Plekhg6 | PLEKHG6 | 0.48 | 2.03E-03 | 1.49E-02 |
| Usp38 | USP38 | 0.48 | 3.94E-03 | 2.52E-02 |
| Riok3 | RIOK3 | 0.48 | 2.64E-04 | 2.98E-03 |
| Ell | ELL | 0.48 | 4.75E-03 | 2.90E-02 |
| Sun2 | SUN2 | 0.48 | 1.11E-03 | 9.48E-03 |
| Lrrfip1 | LRRFIP1 | 0.48 | 2.99E-04 | 3.29E-03 |
| Zfp185 | ZNF185 | 0.48 | 2.85E-03 | 1.95E-02 |
| Usp53 | USP53 | 0.48 | 9.50E-04 | 8.32E-03 |
| Lysmd3 | LYSMD3 | 0.48 | 5.30E-03 | 3.16E-02 |
| Rnf144b | RNF144B | 0.48 | 4.02E-04 | 4.22E-03 |
| Atg2a | ATG2A | 0.48 | 5.94E-03 | 3.43E-02 |
| 3110002H16Rik | C18orf8 | 0.48 | 1.90E-03 | 1.41E-02 |
| Tom1l2 | TOM1L2 | 0.48 | 1.34E-03 | 1.09E-02 |
| Nt5e | NT5E | 0.48 | 1.39E-03 | 1.12E-02 |
| Acp5 | ACP5 | 0.48 | 5.41E-03 | 3.21E-02 |
| Ranbp10 | RANBP10 | 0.49 | 2.12E-03 | 1.55E-02 |
| Wnt4 | WNT4 | 0.49 | 8.19E-04 | 7.44E-03 |
| Tmem184c | TMEM184C | 0.49 | 6.94E-03 | 3.85E-02 |
| AA986860 | C1orf116 | 0.49 | 4.29E-03 | 2.68E-02 |
| Dgkz | DGKZ | 0.49 | 4.06E-03 | 2.58E-02 |
| Fam220a | FAM220A | 0.49 | 5.04E-03 | 3.04E-02 |
| Txnrd1 | TXNRD1 | 0.49 | 2.27E-03 | 1.63E-02 |
| Ctps | CTPS1 | 0.49 | 9.58E-03 | 4.93E-02 |
| Ywhag | YWHAG | 0.49 | 8.95E-05 | 1.27E-03 |
| Rab11fip5 | RAB11FIP5 | 0.49 | 4.21E-03 | 2.65E-02 |
| Usp54 | USP54 | 0.49 | 1.87E-03 | 1.40E-02 |
| Dync1li1 | DYNC1LI1 | 0.49 | 2.19E-04 | 2.57E-03 |
| Efhd2 | EFHD2 | 0.49 | 4.33E-03 | 2.71E-02 |
| Cadm4 | CADM4 | 0.49 | 3.30E-03 | 2.20E-02 |
| Mef2d | MEF2D | 0.49 | 2.15E-03 | 1.57E-02 |
| Tinagl1 | TINAGL1 | 0.49 | 9.13E-03 | 4.77E-02 |
| Lxn | LXN | 0.49 | 2.01E-03 | 1.48E-02 |
| Hip1r | HIP1R | 0.49 | 5.82E-03 | 3.40E-02 |
| Pgs1 | PGS1 | 0.50 | 1.28E-03 | 1.05E-02 |
| Bak1 | BAK1 | 0.50 | 1.78E-03 | 1.35E-02 |
| Garem | GAREM1 | 0.50 | 2.28E-03 | 1.64E-02 |
| Rassf5 | RASSF5 | 0.50 | 5.27E-03 | 3.14E-02 |
| Nab2 | NAB2 | 0.50 | 2.57E-03 | 1.80E-02 |
| Arf2 | NA | 0.50 | 7.32E-04 | 6.78E-03 |
| Padi1 | PADI1 | 0.50 | 4.58E-03 | 2.82E-02 |
| Bspry | BSPRY | 0.50 | 1.34E-03 | 1.09E-02 |
| Ptpre | PTPRE | 0.50 | 9.58E-03 | 4.93E-02 |
| Skil | SKIL | 0.50 | 2.22E-03 | 1.60E-02 |
| Csf1r | CSF1R | 0.50 | 4.84E-03 | 2.95E-02 |
| Clu | CLU | 0.50 | 6.97E-03 | 3.86E-02 |
| Slc6a8 | SLC6A8 | 0.50 | 9.28E-05 | 1.31E-03 |
| Srxn1 | SRXN1 | 0.50 | 4.31E-04 | 4.46E-03 |
| Atp6v1a | ATP6V1A | 0.50 | 2.43E-04 | 2.79E-03 |
| Oser1 | OSER1 | 0.50 | 2.41E-03 | 1.71E-02 |
| Dlgap4 | DLGAP4 | 0.50 | 1.48E-04 | 1.91E-03 |
| Pdzk1ip1 | PDZK1IP1 | 0.50 | 8.34E-03 | 4.44E-02 |
| Rab8b | RAB8B | 0.50 | 6.99E-03 | 3.86E-02 |
| Rars | RARS | 0.50 | 1.06E-03 | 9.14E-03 |
| Wnt7b | WNT7B | 0.50 | 9.25E-04 | 8.14E-03 |
| Gpr87 | GPR87 | 0.51 | 2.64E-03 | 1.85E-02 |
| Tspan5 | TSPAN5 | 0.51 | 4.44E-04 | 4.56E-03 |
| Plxdc2 | PLXDC2 | 0.51 | 8.65E-04 | 7.73E-03 |
| Dda1 | DDA1 | 0.51 | 7.86E-04 | 7.20E-03 |
| Ckap4 | CKAP4 | 0.51 | 3.73E-04 | 3.98E-03 |
| Ctsz | CTSZ | 0.51 | 7.10E-03 | 3.91E-02 |
| Cry1 | CRY1 | 0.51 | 6.52E-03 | 3.68E-02 |
| Serpinb6b | SERPINB6 | 0.51 | 7.47E-05 | 1.09E-03 |
| Bscl2 | BSCL2 | 0.51 | 1.14E-03 | 9.67E-03 |
| Ankrd44 | ANKRD44 | 0.51 | 4.13E-03 | 2.61E-02 |
| Abhd6 | ABHD6 | 0.51 | 7.34E-04 | 6.78E-03 |
| Eps8l2 | EPS8L2 | 0.51 | 3.82E-03 | 2.46E-02 |
| Tbc1d10b | TBC1D10B | 0.51 | 2.52E-03 | 1.77E-02 |
| Slmo2 | PRELID3B | 0.51 | 1.47E-03 | 1.16E-02 |
| Zmiz1 | ZMIZ1 | 0.51 | 2.80E-04 | 3.13E-03 |
| Tmcc3 | TMCC3 | 0.51 | 2.64E-04 | 2.98E-03 |
| Nudt5 | NUDT5 | 0.51 | 2.65E-03 | 1.85E-02 |
| Pls3 | PLS3 | 0.51 | 3.54E-03 | 2.32E-02 |
| Mcc | MCC | 0.52 | 9.98E-04 | 8.67E-03 |
| Rpl29 | RPL29 | 0.52 | 8.71E-04 | 7.76E-03 |
| Ccdc71l | CCDC71L | 0.52 | 2.67E-03 | 1.86E-02 |
| Rnd3 | RND3 | 0.52 | 1.75E-03 | 1.33E-02 |
| Jmjd6 | JMJD6 | 0.52 | 5.24E-03 | 3.13E-02 |
| Cpeb4 | CPEB4 | 0.52 | 2.08E-04 | 2.48E-03 |
| Pik3r3 | PIK3R3 | 0.52 | 2.18E-04 | 2.56E-03 |
| Gltp | GLTP | 0.52 | 1.31E-03 | 1.06E-02 |
| Tmem120a | TMEM120A | 0.52 | 3.25E-03 | 2.18E-02 |
| Ces2f | CES2 | 0.52 | 1.20E-03 | 1.01E-02 |
| Plbd1 | PLBD1 | 0.52 | 1.87E-03 | 1.40E-02 |
| Adrbk1 | GRK2 | 0.52 | 2.24E-04 | 2.62E-03 |
| Ccnd3 | CCND3 | 0.52 | 2.11E-04 | 2.50E-03 |
| Chmp1b | CHMP1B | 0.53 | 4.77E-04 | 4.82E-03 |
| Mcm3ap | MCM3AP | 0.53 | 3.75E-03 | 2.42E-02 |
| Pde12 | PDE12 | 0.53 | 1.56E-03 | 1.22E-02 |
| Max | MAX | 0.53 | 1.86E-04 | 2.26E-03 |
| Leprel1 | NA | 0.53 | 3.43E-03 | 2.26E-02 |
| Degs1 | DEGS1 | 0.53 | 8.55E-04 | 7.67E-03 |
| Map2k2 | MAP2K2 | 0.53 | 1.42E-03 | 1.13E-02 |
| Sh3rf2 | SH3RF2 | 0.53 | 6.73E-04 | 6.32E-03 |
| Kazn | KAZN | 0.53 | 5.63E-04 | 5.48E-03 |
| Rps6kb2 | RPS6KB2 | 0.53 | 6.42E-03 | 3.64E-02 |
| Coq10b | COQ10B | 0.53 | 2.88E-03 | 1.97E-02 |
| Golga7 | GOLGA7 | 0.53 | 2.00E-04 | 2.40E-03 |
| Rcn1 | RCN1 | 0.53 | 1.26E-03 | 1.04E-02 |
| Dpp3 | DPP3 | 0.53 | 5.10E-04 | 5.07E-03 |
| Atp6v1e1 | ATP6V1E1 | 0.53 | 6.73E-04 | 6.32E-03 |
| Baz1a | BAZ1A | 0.53 | 5.24E-04 | 5.18E-03 |
| Prom2 | PROM2 | 0.53 | 4.23E-04 | 4.38E-03 |
| Actn4 | ACTN4 | 0.53 | 7.56E-04 | 6.96E-03 |
| Slc39a6 | SLC39A6 | 0.53 | 1.56E-04 | 1.97E-03 |
| Smap2 | SMAP2 | 0.53 | 1.25E-03 | 1.03E-02 |
| Sidt2 | SIDT2 | 0.53 | 1.77E-03 | 1.34E-02 |
| Cdc42ep3 | CDC42EP3 | 0.54 | 6.79E-04 | 6.37E-03 |
| Actb | ACTB | 0.54 | 4.19E-03 | 2.63E-02 |
| 5031439G07Rik | KIAA0930 | 0.54 | 1.41E-03 | 1.13E-02 |
| Frrs1 | FRRS1 | 0.54 | 1.57E-03 | 1.23E-02 |
| Map3k11 | MAP3K11 | 0.54 | 7.78E-03 | 4.21E-02 |
| Csnk2b | CSNK2B | 0.54 | 2.90E-04 | 3.21E-03 |
| Prelid1 | PRELID1 | 0.54 | 2.74E-04 | 3.07E-03 |
| Psat1 | PSAT1 | 0.54 | 8.26E-05 | 1.19E-03 |
| Gm16515 | NA | 0.54 | 1.94E-03 | 1.44E-02 |
| Def8 | DEF8 | 0.54 | 8.16E-04 | 7.42E-03 |
| Rab7 | RAB7A | 0.54 | 4.54E-04 | 4.65E-03 |
| Msrb1 | MSRB1 | 0.54 | 2.65E-03 | 1.85E-02 |
| Smpd1 | SMPD1 | 0.54 | 6.98E-03 | 3.86E-02 |
| Tmem79 | TMEM79 | 0.54 | 2.53E-04 | 2.89E-03 |
| Mtmr10 | MTMR10 | 0.54 | 1.18E-04 | 1.59E-03 |
| AI846148 | C11orf84 | 0.54 | 6.67E-03 | 3.75E-02 |
| Zdhhc21 | ZDHHC21 | 0.54 | 1.83E-03 | 1.37E-02 |
| Gyk | NA | 0.54 | 9.22E-03 | 4.81E-02 |
| Osbpl7 | OSBPL7 | 0.54 | 6.75E-03 | 3.77E-02 |
| Mcm4 | MCM4 | 0.55 | 8.20E-04 | 7.44E-03 |
| Gapdh | NA | 0.55 | 6.45E-05 | 9.71E-04 |
| Gipc1 | GIPC1 | 0.55 | 2.23E-03 | 1.61E-02 |
| Top2a | TOP2A | 0.55 | 6.99E-03 | 3.86E-02 |
| Pcsk6 | PCSK6 | 0.55 | 8.11E-04 | 7.39E-03 |
| Aacs | AACS | 0.55 | 9.38E-03 | 4.86E-02 |
| Cltb | CLTB | 0.55 | 2.36E-04 | 2.74E-03 |
| Rhob | RHOB | 0.55 | 1.87E-03 | 1.39E-02 |
| Pgam1 | PGAM1 | 0.55 | 3.20E-05 | 5.45E-04 |
| Adamtsl4 | ADAMTSL4 | 0.55 | 1.18E-03 | 9.87E-03 |
| Lmo7 | LMO7 | 0.55 | 1.11E-03 | 9.48E-03 |
| Atp2c2 | ATP2C2 | 0.56 | 3.82E-03 | 2.46E-02 |
| Klc1 | KLC1 | 0.56 | 1.13E-03 | 9.56E-03 |
| Rab11a | RAB11A | 0.56 | 1.22E-03 | 1.02E-02 |
| Rnf19b | RNF19B | 0.56 | 3.18E-04 | 3.48E-03 |
| Ccng1 | CCNG1 | 0.56 | 8.60E-04 | 7.69E-03 |
| S100a16 | S100A16 | 0.56 | 2.64E-03 | 1.85E-02 |
| Fnip2 | FNIP2 | 0.56 | 2.62E-03 | 1.83E-02 |
| Erf | ERF | 0.56 | 4.39E-03 | 2.73E-02 |
| Arl5b | ARL5B | 0.56 | 1.20E-03 | 1.01E-02 |
| Cbr3 | CBR3 | 0.56 | 1.53E-03 | 1.20E-02 |
| Cerk | CERK | 0.56 | 4.01E-04 | 4.21E-03 |
| Cpped1 | CPPED1 | 0.56 | 2.58E-04 | 2.93E-03 |
| Gpcpd1 | GPCPD1 | 0.56 | 3.94E-04 | 4.14E-03 |
| Smg8 | SMG8 | 0.56 | 3.25E-04 | 3.55E-03 |
| Sowahb | SOWAHB | 0.56 | 1.37E-03 | 1.11E-02 |
| Glul | GLUL | 0.57 | 3.78E-03 | 2.44E-02 |
| Pdlim2 | PDLIM2 | 0.57 | 1.81E-03 | 1.36E-02 |
| Piezo1 | PIEZO1 | 0.57 | 1.87E-04 | 2.27E-03 |
| Cblc | CBLC | 0.57 | 1.25E-03 | 1.03E-02 |
| Rplp1 | RPLP1 | 0.57 | 3.13E-03 | 2.12E-02 |
| Amotl2 | AMOTL2 | 0.57 | 6.10E-04 | 5.85E-03 |
| Sdr42e1 | SDR42E1 | 0.57 | 3.70E-03 | 2.40E-02 |
| Rnasek | RNASEK | 0.57 | 1.21E-04 | 1.62E-03 |
| Sdcbp2 | SDCBP2 | 0.57 | 4.46E-03 | 2.77E-02 |
| Tmsb10 | TMSB10 | 0.57 | 2.18E-04 | 2.56E-03 |
| Plekhm2 | PLEKHM2 | 0.57 | 7.13E-04 | 6.63E-03 |
| Lgmn | LGMN | 0.57 | 4.49E-03 | 2.78E-02 |
| Elk3 | ELK3 | 0.57 | 1.10E-03 | 9.41E-03 |
| Ppp1r14b | PPP1R14B | 0.57 | 2.30E-04 | 2.67E-03 |
| Mcm6 | MCM6 | 0.57 | 1.12E-03 | 9.52E-03 |
| Shroom3 | SHROOM3 | 0.57 | 1.93E-04 | 2.32E-03 |
| Slc26a9 | SLC26A9 | 0.57 | 4.14E-03 | 2.61E-02 |
| Mpp1 | MPP1 | 0.57 | 1.55E-04 | 1.96E-03 |
| Blnk | BLNK | 0.57 | 1.37E-04 | 1.79E-03 |
| Rps6ka4 | RPS6KA4 | 0.57 | 1.00E-04 | 1.38E-03 |
| Mcl1 | MCL1 | 0.57 | 1.23E-03 | 1.02E-02 |
| Bcl2l1 | BCL2L1 | 0.57 | 4.43E-04 | 4.55E-03 |
| Rbms1 | RBMS1 | 0.57 | 9.64E-06 | 2.03E-04 |
| S100a14 | S100A14 | 0.57 | 1.92E-03 | 1.43E-02 |
| Cdca4 | CDCA4 | 0.58 | 7.97E-05 | 1.16E-03 |
| Arhgap27 | ARHGAP27 | 0.58 | 1.91E-04 | 2.30E-03 |
| Eps8 | EPS8 | 0.58 | 1.17E-05 | 2.37E-04 |
| Pycard | PYCARD | 0.58 | 6.97E-04 | 6.50E-03 |
| Irs2 | IRS2 | 0.58 | 9.56E-03 | 4.93E-02 |
| Mgmt | MGMT | 0.58 | 4.79E-03 | 2.92E-02 |
| Zbtb7a | ZBTB7A | 0.58 | 3.49E-06 | 8.95E-05 |
| Fen1 | FEN1 | 0.58 | 5.79E-03 | 3.38E-02 |
| Plac8 | PLAC8 | 0.58 | 4.43E-03 | 2.75E-02 |
| Slc2a1 | SLC2A1 | 0.58 | 1.81E-03 | 1.36E-02 |
| Eif4ebp1 | EIF4EBP1 | 0.58 | 7.04E-03 | 3.88E-02 |
| S100a10 | S100A10 | 0.58 | 5.21E-03 | 3.12E-02 |
| Pip5k1c | PIP5K1C | 0.58 | 7.79E-04 | 7.15E-03 |
| Ppif | PPIF | 0.58 | 1.70E-03 | 1.31E-02 |
| Reep4 | REEP4 | 0.58 | 1.20E-03 | 1.00E-02 |
| Bicd2 | BICD2 | 0.58 | 7.25E-05 | 1.07E-03 |
| Apaf1 | APAF1 | 0.58 | 7.74E-04 | 7.11E-03 |
| Spats2 | SPATS2 | 0.59 | 2.48E-03 | 1.75E-02 |
| Vps37b | VPS37B | 0.59 | 5.05E-04 | 5.04E-03 |
| Grhl1 | GRHL1 | 0.59 | 1.33E-04 | 1.75E-03 |
| Nlrx1 | NLRX1 | 0.59 | 1.59E-03 | 1.24E-02 |
| Abhd5 | ABHD5 | 0.59 | 2.56E-05 | 4.52E-04 |
| Dsp | DSP | 0.59 | 1.25E-03 | 1.03E-02 |
| Specc1 | SPECC1 | 0.59 | 1.16E-03 | 9.79E-03 |
| Tmem43 | TMEM43 | 0.59 | 1.78E-04 | 2.18E-03 |
| N4bp2l1 | N4BP2L1 | 0.59 | 1.81E-03 | 1.36E-02 |
| Ly6g6e | LY6G6E | 0.59 | 6.97E-03 | 3.86E-02 |
| Lrrc8a | LRRC8A | 0.59 | 2.17E-05 | 3.99E-04 |
| Zfp772 | NA | 0.59 | 5.16E-03 | 3.10E-02 |
| Ralgds | RALGDS | 0.59 | 6.97E-06 | 1.56E-04 |
| Stk35 | STK35 | 0.59 | 8.69E-04 | 7.76E-03 |
| Ttpal | TTPAL | 0.59 | 2.58E-04 | 2.93E-03 |
| Rnf126 | RNF126 | 0.59 | 7.72E-03 | 4.19E-02 |
| Dusp6 | DUSP6 | 0.59 | 2.52E-05 | 4.47E-04 |
| Rap2a | RAP2A | 0.59 | 3.17E-05 | 5.42E-04 |
| Zfp36 | ZFP36 | 0.59 | 2.38E-03 | 1.70E-02 |
| Sox13 | SOX13 | 0.59 | 3.05E-03 | 2.07E-02 |
| Tmem97 | TMEM97 | 0.59 | 8.69E-03 | 4.59E-02 |
| 5430435G22Rik | NA | 0.59 | 6.95E-04 | 6.48E-03 |
| Gtf2f1 | GTF2F1 | 0.59 | 9.71E-05 | 1.36E-03 |
| Ampd3 | AMPD3 | 0.59 | 7.58E-03 | 4.13E-02 |
| Sc5d | SC5D | 0.59 | 4.83E-05 | 7.60E-04 |
| Wnt9a | WNT9A | 0.60 | 6.06E-03 | 3.48E-02 |
| Aprt | APRT | 0.60 | 3.86E-03 | 2.48E-02 |
| Aim1l | AIM1L | 0.60 | 6.23E-04 | 5.95E-03 |
| Akirin1 | AKIRIN1 | 0.60 | 2.51E-05 | 4.46E-04 |
| Ell2 | ELL2 | 0.60 | 3.36E-04 | 3.65E-03 |
| Racgap1 | RACGAP1 | 0.60 | 4.78E-03 | 2.92E-02 |
| Gm2a | GM2A | 0.60 | 1.39E-03 | 1.11E-02 |
| Acot7 | ACOT7 | 0.60 | 7.92E-04 | 7.24E-03 |
| Apobec1 | APOBEC1 | 0.60 | 1.70E-04 | 2.10E-03 |
| Prr13 | NA | 0.60 | 1.55E-04 | 1.96E-03 |
| Fam129b | FAM129B | 0.60 | 2.24E-03 | 1.62E-02 |
| Tceb2 | TCEB2 | 0.60 | 1.02E-03 | 8.80E-03 |
| 9030617O03Rik | C14orf159 | 0.60 | 6.30E-03 | 3.59E-02 |
| Brwd3 | BRWD3 | 0.60 | 2.62E-04 | 2.96E-03 |
| Eif6 | EIF6 | 0.60 | 2.64E-04 | 2.98E-03 |
| Tmem104 | TMEM104 | 0.60 | 8.08E-03 | 4.34E-02 |
| Trim29 | TRIM29 | 0.61 | 1.42E-03 | 1.13E-02 |
| Syt8 | SYT8 | 0.61 | 2.58E-03 | 1.81E-02 |
| Dclre1b | DCLRE1B | 0.61 | 3.28E-04 | 3.57E-03 |
| Plau | PLAU | 0.61 | 2.26E-03 | 1.63E-02 |
| Fmn1 | FMN1 | 0.61 | 4.95E-04 | 4.96E-03 |
| Grina | GRINA | 0.61 | 5.95E-03 | 3.44E-02 |
| Ctsb | CTSB | 0.61 | 5.31E-03 | 3.16E-02 |
| Polr2f | POLR2F | 0.61 | 5.96E-03 | 3.44E-02 |
| Kifc3 | KIFC3 | 0.61 | 8.77E-04 | 7.80E-03 |
| Scd2 | SCD | 0.61 | 9.87E-05 | 1.37E-03 |
| Pvrl1 | NA | 0.61 | 1.80E-04 | 2.20E-03 |
| Samd8 | SAMD8 | 0.61 | 2.93E-05 | 5.07E-04 |
| Fam160a1 | FAM160A1 | 0.61 | 4.55E-06 | 1.11E-04 |
| Ier2 | IER2 | 0.61 | 5.67E-03 | 3.33E-02 |
| Mt1 | MT1A | 0.61 | 9.46E-03 | 4.90E-02 |
| Fosl2 | FOSL2 | 0.61 | 2.66E-04 | 2.99E-03 |
| Cndp2 | CNDP2 | 0.61 | 1.38E-05 | 2.73E-04 |
| Cyp3a13 | CYP3A4 | 0.61 | 1.58E-03 | 1.23E-02 |
| Spink5 | SPINK5 | 0.62 | 2.02E-03 | 1.49E-02 |
| Zfp710 | ZNF710 | 0.62 | 1.72E-04 | 2.12E-03 |
| Slfn2 | SLFN12L | 0.62 | 5.05E-03 | 3.04E-02 |
| 2310007B03Rik | C2orf54 | 0.62 | 6.60E-04 | 6.22E-03 |
| Phyhip | PHYHIP | 0.62 | 1.41E-03 | 1.13E-02 |
| Map4k4 | MAP4K4 | 0.62 | 9.24E-07 | 3.14E-05 |
| Ggta1 | NA | 0.62 | 2.03E-05 | 3.77E-04 |
| Slc43a2 | SLC43A2 | 0.62 | 8.89E-04 | 7.88E-03 |
| Ctsk | CTSK | 0.62 | 5.92E-03 | 3.43E-02 |
| Slc7a5 | SLC7A5 | 0.62 | 3.31E-03 | 2.21E-02 |
| Chmp4c | CHMP4C | 0.62 | 7.45E-05 | 1.09E-03 |
| Fbxw9 | FBXW9 | 0.62 | 5.42E-03 | 3.21E-02 |
| Defb1 | DEFB1 | 0.62 | 6.16E-03 | 3.53E-02 |
| Tmem106a | TMEM106A | 0.62 | 3.20E-03 | 2.15E-02 |
| Hif1a | HIF1A | 0.62 | 4.67E-04 | 4.74E-03 |
| Tubg1 | TUBG1 | 0.62 | 1.31E-03 | 1.07E-02 |
| Tmem71 | TMEM71 | 0.62 | 1.59E-03 | 1.24E-02 |
| Hebp2 | HEBP2 | 0.62 | 4.12E-04 | 4.30E-03 |
| Mon1a | MON1A | 0.62 | 2.78E-03 | 1.92E-02 |
| Prdm1 | PRDM1 | 0.62 | 5.01E-04 | 5.01E-03 |
| Zmat3 | ZMAT3 | 0.62 | 6.96E-05 | 1.04E-03 |
| Cyr61 | CYR61 | 0.62 | 7.88E-03 | 4.26E-02 |
| Lsp1 | LSP1 | 0.63 | 7.28E-03 | 3.99E-02 |
| Rhbdl2 | RHBDL2 | 0.63 | 1.09E-03 | 9.32E-03 |
| Kctd5 | KCTD5 | 0.63 | 4.70E-05 | 7.41E-04 |
| Aim1 | AIM1 | 0.63 | 5.91E-04 | 5.69E-03 |
| Bmp2k | BMP2K | 0.63 | 4.19E-05 | 6.71E-04 |
| Def6 | DEF6 | 0.63 | 5.85E-03 | 3.40E-02 |
| Cyth3 | CYTH3 | 0.63 | 9.78E-05 | 1.36E-03 |
| Fth1 | FTH1 | 0.63 | 4.90E-03 | 2.98E-02 |
| Cryab | CRYAB | 0.63 | 2.75E-03 | 1.91E-02 |
| Hr | HR | 0.63 | 2.39E-04 | 2.76E-03 |
| Hs3st1 | HS3ST1 | 0.63 | 7.99E-04 | 7.30E-03 |
| Pgd | PGD | 0.63 | 3.21E-05 | 5.46E-04 |
| Cstb | CSTB | 0.63 | 2.78E-06 | 7.58E-05 |
| Itprip | NA | 0.63 | 1.27E-04 | 1.67E-03 |
| 5-Sep | 5-Sep | 0.63 | 3.53E-05 | 5.84E-04 |
| Pik3cb | PIK3CB | 0.63 | 2.45E-05 | 4.37E-04 |
| Lama3 | LAMA3 | 0.63 | 2.61E-03 | 1.83E-02 |
| S100a4 | S100A4 | 0.63 | 5.76E-03 | 3.37E-02 |
| Klf10 | KLF10 | 0.63 | 1.49E-04 | 1.91E-03 |
| Sdc3 | SDC3 | 0.63 | 5.65E-04 | 5.49E-03 |
| Tob1 | TOB1 | 0.63 | 1.59E-04 | 2.00E-03 |
| 6430548M08Rik | KIAA0513 | 0.63 | 1.19E-04 | 1.59E-03 |
| Fam46b | FAM46B | 0.64 | 5.80E-05 | 8.90E-04 |
| Cttnbp2nl | CTTNBP2NL | 0.64 | 1.30E-05 | 2.60E-04 |
| Gpr160 | GPR160 | 0.64 | 1.01E-03 | 8.72E-03 |
| Ablim3 | ABLIM3 | 0.64 | 8.68E-05 | 1.25E-03 |
| Zfand2a | ZFAND2A | 0.64 | 7.94E-05 | 1.15E-03 |
| Pkm | PKM | 0.64 | 3.28E-04 | 3.57E-03 |
| Spsb3 | SPSB3 | 0.64 | 3.36E-03 | 2.23E-02 |
| Fzr1 | FZR1 | 0.64 | 3.55E-04 | 3.81E-03 |
| Serpina9 | SERPINA9 | 0.64 | 9.14E-03 | 4.77E-02 |
| Mdm2 | MDM2 | 0.64 | 3.24E-06 | 8.47E-05 |
| Stk17b | STK17B | 0.64 | 5.37E-03 | 3.19E-02 |
| Lmna | LMNA | 0.64 | 1.26E-04 | 1.67E-03 |
| Sesn2 | SESN2 | 0.64 | 6.67E-04 | 6.27E-03 |
| Dusp4 | DUSP4 | 0.64 | 1.47E-03 | 1.16E-02 |
| Dck | DCK | 0.64 | 6.68E-04 | 6.28E-03 |
| Esrra | ESRRA | 0.64 | 5.71E-03 | 3.35E-02 |
| Polk | POLK | 0.64 | 1.43E-04 | 1.85E-03 |
| Cdh3 | CDH3 | 0.64 | 4.17E-03 | 2.63E-02 |
| Sowahc | SOWAHC | 0.65 | 3.32E-04 | 3.62E-03 |
| Rsrp1 | RSRP1 | 0.65 | 8.79E-05 | 1.25E-03 |
| Kcnk6 | KCNK6 | 0.65 | 1.51E-03 | 1.19E-02 |
| Rel | REL | 0.65 | 4.01E-03 | 2.55E-02 |
| Clic3 | CLIC3 | 0.65 | 2.28E-04 | 2.66E-03 |
| Snx7 | SNX7 | 0.65 | 1.63E-03 | 1.26E-02 |
| Vat1 | VAT1 | 0.65 | 2.38E-04 | 2.75E-03 |
| Sh3gl1 | SH3GL1 | 0.65 | 3.09E-04 | 3.39E-03 |
| Arhgef1 | ARHGEF1 | 0.65 | 1.77E-03 | 1.34E-02 |
| Anxa1 | ANXA1 | 0.65 | 3.69E-03 | 2.39E-02 |
| Slc35e4 | SLC35E4 | 0.65 | 5.23E-03 | 3.13E-02 |
| Nucb2 | NUCB2 | 0.65 | 8.45E-04 | 7.62E-03 |
| Pacsin3 | PACSIN3 | 0.65 | 1.13E-03 | 9.58E-03 |
| Elovl6 | ELOVL6 | 0.65 | 3.49E-04 | 3.76E-03 |
| Tuba8 | TUBA8 | 0.65 | 4.62E-03 | 2.84E-02 |
| Rhog | RHOG | 0.65 | 1.53E-04 | 1.95E-03 |
| Mark4 | MARK4 | 0.65 | 2.16E-03 | 1.57E-02 |
| Anapc15 | ANAPC15 | 0.66 | 1.72E-03 | 1.32E-02 |
| Midn | MIDN | 0.66 | 1.20E-05 | 2.41E-04 |
| Serpinb5 | SERPINB5 | 0.66 | 7.08E-05 | 1.05E-03 |
| Tmem154 | TMEM154 | 0.66 | 1.38E-06 | 4.31E-05 |
| H2afj | H2AFJ | 0.66 | 9.31E-03 | 4.84E-02 |
| Dab2 | DAB2 | 0.66 | 3.68E-03 | 2.39E-02 |
| Eepd1 | EEPD1 | 0.66 | 8.92E-03 | 4.69E-02 |
| Lipe | LIPE | 0.66 | 7.71E-03 | 4.19E-02 |
| Vim | VIM | 0.66 | 1.25E-03 | 1.03E-02 |
| Nme1 | NME1 | 0.66 | 1.13E-05 | 2.30E-04 |
| Trib1 | TRIB1 | 0.66 | 3.43E-04 | 3.70E-03 |
| Hmgcr | HMGCR | 0.66 | 7.93E-06 | 1.73E-04 |
| Rcc1 | RCC1 | 0.67 | 2.73E-03 | 1.90E-02 |
| Elovl1 | ELOVL1 | 0.67 | 3.16E-05 | 5.41E-04 |
| Sh3bp5l | SH3BP5L | 0.67 | 1.09E-04 | 1.50E-03 |
| Krt7 | KRT7 | 0.67 | 2.75E-03 | 1.90E-02 |
| Lrp12 | LRP12 | 0.67 | 9.85E-06 | 2.07E-04 |
| Capg | CAPG | 0.67 | 2.21E-04 | 2.59E-03 |
| Tuba4a | TUBA4A | 0.67 | 1.83E-04 | 2.24E-03 |
| 2310014L17Rik | NA | 0.67 | 3.72E-03 | 2.41E-02 |
| Entpd3 | ENTPD3 | 0.67 | 1.68E-04 | 2.09E-03 |
| Camk2d | CAMK2D | 0.67 | 1.49E-07 | 7.26E-06 |
| Gja1 | GJA1 | 0.67 | 7.80E-04 | 7.16E-03 |
| Pbx3 | PBX3 | 0.67 | 1.24E-04 | 1.64E-03 |
| Bax | BAX | 0.68 | 8.53E-05 | 1.23E-03 |
| Trim16 | TRIM16L | 0.68 | 2.75E-06 | 7.56E-05 |
| Rgs12 | RGS12 | 0.68 | 4.81E-04 | 4.85E-03 |
| Suox | SUOX | 0.68 | 2.86E-04 | 3.18E-03 |
| Tnfrsf10b | TNFRSF10B | 0.68 | 1.36E-03 | 1.10E-02 |
| Ccdc122 | CCDC122 | 0.68 | 5.25E-03 | 3.13E-02 |
| Tsc22d2 | TSC22D2 | 0.68 | 9.24E-05 | 1.30E-03 |
| Fam111a | FAM111A | 0.68 | 5.19E-05 | 8.07E-04 |
| Pip5k1a | PIP5K1A | 0.68 | 8.57E-06 | 1.84E-04 |
| Wipi1 | WIPI1 | 0.68 | 3.39E-06 | 8.75E-05 |
| Lcp1 | LCP1 | 0.68 | 6.78E-03 | 3.78E-02 |
| Mid2 | MID2 | 0.68 | 6.91E-06 | 1.55E-04 |
| Gpx2 | GPX2 | 0.68 | 7.28E-05 | 1.07E-03 |
| Smim3 | SMIM3 | 0.68 | 2.38E-05 | 4.30E-04 |
| Sh3bp1 | SH3BP1 | 0.68 | 9.40E-05 | 1.32E-03 |
| Slc9a3r1 | SLC9A3R1 | 0.68 | 3.33E-06 | 8.62E-05 |
| Ppp1r15a | PPP1R15A | 0.68 | 1.04E-03 | 8.95E-03 |
| Ddit3 | DDIT3 | 0.68 | 4.50E-03 | 2.78E-02 |
| 1810037I17Rik | C4orf3 | 0.68 | 2.36E-05 | 4.27E-04 |
| Rap2b | RAP2B | 0.69 | 2.54E-07 | 1.10E-05 |
| Sbf1 | SBF1 | 0.69 | 3.40E-05 | 5.70E-04 |
| Phlda3 | PHLDA3 | 0.69 | 1.19E-04 | 1.59E-03 |
| Slc52a3 | SLC52A3 | 0.69 | 2.85E-04 | 3.17E-03 |
| Ddit4 | DDIT4 | 0.69 | 6.10E-04 | 5.86E-03 |
| Gm9573 | MUC21 | 0.69 | 3.82E-03 | 2.46E-02 |
| Eps8l1 | EPS8L1 | 0.69 | 2.50E-04 | 2.87E-03 |
| Glipr2 | GLIPR2 | 0.69 | 2.58E-05 | 4.53E-04 |
| Arap2 | ARAP2 | 0.69 | 3.08E-07 | 1.30E-05 |
| Cdc42bpg | CDC42BPG | 0.70 | 2.40E-05 | 4.32E-04 |
| Kcnq5 | KCNQ5 | 0.70 | 8.36E-03 | 4.45E-02 |
| Dhrs9 | DHRS9 | 0.70 | 2.75E-05 | 4.80E-04 |
| Errfi1 | ERRFI1 | 0.70 | 1.07E-04 | 1.46E-03 |
| Lrrc20 | LRRC20 | 0.70 | 1.17E-03 | 9.83E-03 |
| Ccnb2 | CCNB2 | 0.70 | 5.97E-03 | 3.45E-02 |
| Sdc1 | SDC1 | 0.70 | 1.44E-05 | 2.83E-04 |
| Prkch | PRKCH | 0.70 | 7.20E-05 | 1.07E-03 |
| Eno1b | ENO1 | 0.70 | 2.51E-04 | 2.88E-03 |
| Ly6d | LY6D | 0.70 | 3.80E-04 | 4.04E-03 |
| AI661453 | C6orf132 | 0.70 | 1.33E-04 | 1.75E-03 |
| Macc1 | MACC1 | 0.71 | 5.99E-06 | 1.38E-04 |
| Ccna2 | CCNA2 | 0.71 | 1.27E-03 | 1.04E-02 |
| Clk1 | CLK1 | 0.71 | 8.98E-05 | 1.27E-03 |
| Phgdh | PHGDH | 0.71 | 1.71E-04 | 2.11E-03 |
| Sertad1 | SERTAD1 | 0.71 | 1.61E-04 | 2.02E-03 |
| Scel | SCEL | 0.71 | 1.99E-04 | 2.38E-03 |
| Pof1b | POF1B | 0.71 | 8.77E-04 | 7.80E-03 |
| Blzf1 | BLZF1 | 0.71 | 1.34E-04 | 1.75E-03 |
| Rnasel | RNASEL | 0.71 | 1.01E-04 | 1.39E-03 |
| 1700017B05Rik | C15orf39 | 0.71 | 5.47E-06 | 1.28E-04 |
| Dsc3 | DSC3 | 0.71 | 2.06E-06 | 5.94E-05 |
| Jup | JUP | 0.71 | 1.74E-04 | 2.13E-03 |
| Mal | MAL | 0.71 | 1.01E-03 | 8.73E-03 |
| Nsdhl | NSDHL | 0.71 | 9.71E-05 | 1.36E-03 |
| Ptgr1 | PTGR1 | 0.71 | 1.41E-04 | 1.83E-03 |
| Gale | GALE | 0.71 | 3.33E-05 | 5.63E-04 |
| Tgfbi | TGFBI | 0.71 | 3.06E-04 | 3.36E-03 |
| 1110008P14Rik | C9orf16 | 0.72 | 1.77E-03 | 1.34E-02 |
| Nrp2 | NRP2 | 0.72 | 2.40E-06 | 6.73E-05 |
| Bcl3 | BCL3 | 0.72 | 8.91E-05 | 1.27E-03 |
| Helb | HELB | 0.72 | 3.24E-03 | 2.17E-02 |
| Cks1b | CKS1B | 0.72 | 2.42E-03 | 1.72E-02 |
| Nt5c3 | NT5C3A | 0.72 | 5.30E-03 | 3.16E-02 |
| A430105I19Rik | C15orf52 | 0.72 | 2.07E-04 | 2.46E-03 |
| Rhod | RHOD | 0.72 | 1.53E-04 | 1.95E-03 |
| Gla | GLA | 0.72 | 3.23E-03 | 2.17E-02 |
| Ubtd1 | UBTD1 | 0.72 | 7.44E-04 | 6.85E-03 |
| Gramd1c | GRAMD1C | 0.72 | 1.26E-03 | 1.04E-02 |
| Pld3 | PLD3 | 0.72 | 1.61E-04 | 2.02E-03 |
| Tnfrsf12a | TNFRSF12A | 0.72 | 2.72E-03 | 1.89E-02 |
| Scamp5 | SCAMP5 | 0.73 | 7.72E-03 | 4.19E-02 |
| Jun | JUN | 0.73 | 2.97E-04 | 3.28E-03 |
| Hspb1 | HSPB1 | 0.73 | 9.26E-05 | 1.30E-03 |
| Arhgef10l | ARHGEF10L | 0.73 | 2.86E-04 | 3.17E-03 |
| Clcn2 | CLCN2 | 0.73 | 3.02E-03 | 2.06E-02 |
| Nrg1 | NRG1 | 0.73 | 9.68E-03 | 4.97E-02 |
| Fdft1 | FDFT1 | 0.73 | 1.85E-05 | 3.47E-04 |
| Sema4d | SEMA4D | 0.73 | 1.07E-03 | 9.17E-03 |
| Maff | MAFF | 0.73 | 8.17E-08 | 4.35E-06 |
| Pard6b | PARD6B | 0.73 | 9.15E-05 | 1.29E-03 |
| Nfkbia | NFKBIA | 0.74 | 7.73E-06 | 1.70E-04 |
| Blmh | BLMH | 0.74 | 8.58E-04 | 7.69E-03 |
| Rilpl2 | RILPL2 | 0.74 | 6.60E-04 | 6.22E-03 |
| Ripk3 | RIPK3 | 0.74 | 1.54E-03 | 1.21E-02 |
| Nbeal2 | NBEAL2 | 0.74 | 3.68E-05 | 6.04E-04 |
| Ldlr | LDLR | 0.74 | 2.53E-05 | 4.48E-04 |
| 3-Mar | 3-Mar | 0.74 | 2.51E-03 | 1.77E-02 |
| Ptk2b | PTK2B | 0.74 | 3.17E-04 | 3.47E-03 |
| Msh6 | MSH6 | 0.74 | 4.23E-08 | 2.49E-06 |
| Ptp4a3 | PTP4A3 | 0.74 | 7.33E-05 | 1.08E-03 |
| Stx19 | STX19 | 0.74 | 4.68E-04 | 4.74E-03 |
| Lrrc28 | LRRC28 | 0.74 | 3.45E-05 | 5.77E-04 |
| Akr1b8 | AKR1B10 | 0.74 | 3.44E-06 | 8.84E-05 |
| Myo5a | MYO5A | 0.74 | 1.80E-07 | 8.29E-06 |
| Ptrh1 | PTRH1 | 0.75 | 8.74E-03 | 4.61E-02 |
| Abcg1 | ABCG1 | 0.75 | 4.01E-05 | 6.47E-04 |
| Fmnl2 | FMNL2 | 0.75 | 4.37E-04 | 4.50E-03 |
| Perp | PERP | 0.75 | 2.57E-04 | 2.93E-03 |
| Il4ra | IL4R | 0.75 | 7.34E-05 | 1.08E-03 |
| Ppp1r13l | PPP1R13L | 0.75 | 6.26E-06 | 1.43E-04 |
| Btg2 | BTG2 | 0.75 | 1.79E-03 | 1.35E-02 |
| Plekhs1 | PLEKHS1 | 0.75 | 8.04E-03 | 4.32E-02 |
| Irak3 | IRAK3 | 0.75 | 7.82E-03 | 4.23E-02 |
| 8-Sep | 8-Sep | 0.75 | 1.99E-07 | 8.97E-06 |
| Galnt6 | GALNT6 | 0.75 | 1.85E-04 | 2.25E-03 |
| Slc6a14 | SLC6A14 | 0.75 | 1.63E-03 | 1.26E-02 |
| Uchl3 | UCHL3 | 0.75 | 5.49E-04 | 5.36E-03 |
| Dcun1d3 | DCUN1D3 | 0.75 | 3.05E-06 | 8.11E-05 |
| Tspan6 | TSPAN6 | 0.75 | 2.58E-06 | 7.11E-05 |
| Dusp14 | DUSP14 | 0.75 | 4.50E-04 | 4.61E-03 |
| Ppl | PPL | 0.75 | 3.76E-05 | 6.14E-04 |
| Plekha7 | PLEKHA7 | 0.75 | 1.37E-06 | 4.31E-05 |
| Nlrp10 | NLRP10 | 0.76 | 5.17E-04 | 5.13E-03 |
| Plcd1 | PLCD1 | 0.76 | 1.13E-05 | 2.32E-04 |
| Phldb3 | PHLDB3 | 0.76 | 9.68E-05 | 1.35E-03 |
| Llgl2 | LLGL2 | 0.76 | 7.25E-05 | 1.07E-03 |
| Ass1 | ASS1 | 0.76 | 5.55E-06 | 1.30E-04 |
| Mocos | MOCOS | 0.76 | 3.67E-05 | 6.03E-04 |
| Trp53inp1 | TP53INP1 | 0.76 | 5.57E-06 | 1.30E-04 |
| Gm14137 | C15orf62 | 0.76 | 2.07E-05 | 3.83E-04 |
| Serpinb1a | SERPINB1 | 0.76 | 2.82E-05 | 4.90E-04 |
| Defb14 | DEFB103B | 0.76 | 3.29E-03 | 2.20E-02 |
| Foxm1 | FOXM1 | 0.76 | 2.12E-03 | 1.55E-02 |
| Ier3 | IER3 | 0.76 | 4.09E-05 | 6.57E-04 |
| AA467197 | C15orf48 | 0.76 | 3.85E-03 | 2.47E-02 |
| Esd | ESD | 0.76 | 3.42E-07 | 1.41E-05 |
| Tlr2 | TLR2 | 0.76 | 9.65E-04 | 8.44E-03 |
| Alpl | ALPL | 0.77 | 7.45E-03 | 4.07E-02 |
| Myzap | MYZAP | 0.77 | 9.03E-05 | 1.28E-03 |
| Adrb2 | ADRB2 | 0.77 | 1.19E-05 | 2.41E-04 |
| Sqle | SQLE | 0.77 | 5.35E-05 | 8.28E-04 |
| Aldh3b2 | ALDH3B2 | 0.77 | 4.76E-04 | 4.82E-03 |
| Hcls1 | HCLS1 | 0.77 | 2.07E-03 | 1.52E-02 |
| Rgl1 | RGL1 | 0.77 | 4.65E-03 | 2.85E-02 |
| Cast | CAST | 0.77 | 1.66E-07 | 7.77E-06 |
| Ctss | CTSS | 0.77 | 4.59E-03 | 2.82E-02 |
| Lhfpl2 | LHFPL2 | 0.77 | 4.19E-03 | 2.63E-02 |
| Wsb1 | WSB1 | 0.77 | 5.60E-04 | 5.46E-03 |
| Sytl1 | SYTL1 | 0.77 | 3.90E-05 | 6.33E-04 |
| Pigf | PIGF | 0.77 | 2.76E-06 | 7.58E-05 |
| Dusp10 | DUSP10 | 0.77 | 3.05E-04 | 3.36E-03 |
| Arhgap40 | ARHGAP40 | 0.78 | 8.12E-05 | 1.17E-03 |
| Egln3 | EGLN3 | 0.78 | 2.31E-03 | 1.65E-02 |
| Rab31 | RAB31 | 0.78 | 1.77E-06 | 5.29E-05 |
| Slc16a1 | SLC16A1 | 0.78 | 9.71E-04 | 8.48E-03 |
| Cldn1 | CLDN1 | 0.78 | 3.78E-07 | 1.54E-05 |
| Map3k8 | MAP3K8 | 0.78 | 9.88E-04 | 8.59E-03 |
| Gas7 | GAS7 | 0.78 | 3.96E-05 | 6.39E-04 |
| Itgam | ITGAM | 0.78 | 1.25E-03 | 1.03E-02 |
| Rab24 | RAB24 | 0.78 | 8.43E-05 | 1.21E-03 |
| Serpinb2 | SERPINB2 | 0.78 | 4.90E-05 | 7.69E-04 |
| Omp | OMP | 0.78 | 5.19E-03 | 3.11E-02 |
| Pim1 | PIM1 | 0.78 | 8.92E-04 | 7.89E-03 |
| Spag1 | SPAG1 | 0.79 | 1.15E-03 | 9.74E-03 |
| Cenpa | CENPA | 0.79 | 6.73E-03 | 3.76E-02 |
| Pkp1 | PKP1 | 0.79 | 2.88E-06 | 7.77E-05 |
| Pfkfb3 | PFKFB3 | 0.79 | 1.84E-05 | 3.45E-04 |
| Gas2l3 | GAS2L3 | 0.79 | 1.60E-03 | 1.24E-02 |
| Ppp1r10 | PPP1R10 | 0.79 | 2.20E-06 | 6.27E-05 |
| Pfkfb4 | PFKFB4 | 0.79 | 9.29E-05 | 1.31E-03 |
| Ms4a6c | MS4A6A | 0.79 | 5.04E-03 | 3.04E-02 |
| Asap1 | ASAP1 | 0.79 | 4.79E-08 | 2.74E-06 |
| Skp2 | SKP2 | 0.79 | 9.28E-03 | 4.83E-02 |
| Marcksl1 | MARCKSL1 | 0.79 | 1.65E-03 | 1.28E-02 |
| Cars | CARS | 0.79 | 4.63E-07 | 1.81E-05 |
| Pcdh1 | PCDH1 | 0.80 | 1.46E-05 | 2.86E-04 |
| Palm | PALM | 0.80 | 1.19E-03 | 9.99E-03 |
| Il18 | IL18 | 0.80 | 4.37E-04 | 4.50E-03 |
| Dsc2 | DSC2 | 0.80 | 8.89E-05 | 1.27E-03 |
| Mapk6 | MAPK6 | 0.80 | 1.58E-07 | 7.59E-06 |
| Dhcr24 | DHCR24 | 0.80 | 9.15E-07 | 3.11E-05 |
| Nckap1l | NCKAP1L | 0.80 | 2.12E-03 | 1.55E-02 |
| Ndel1 | NDEL1 | 0.80 | 2.36E-07 | 1.04E-05 |
| Hvcn1 | HVCN1 | 0.80 | 2.82E-03 | 1.94E-02 |
| Aknad1 | AKNAD1 | 0.80 | 4.17E-04 | 4.34E-03 |
| Mt2 | MT1X | 0.80 | 2.47E-05 | 4.39E-04 |
| Sbno2 | SBNO2 | 0.81 | 1.53E-04 | 1.95E-03 |
| Cystm1 | CYSTM1 | 0.81 | 5.92E-04 | 5.70E-03 |
| Diap3 | NA | 0.81 | 6.36E-04 | 6.06E-03 |
| Slc16a9 | SLC16A9 | 0.81 | 8.29E-03 | 4.42E-02 |
| Dusp22 | DUSP22 | 0.81 | 1.16E-05 | 2.35E-04 |
| Ptprc | PTPRC | 0.81 | 9.40E-03 | 4.87E-02 |
| Zfp52 | NA | 0.81 | 2.14E-05 | 3.94E-04 |
| Klhl18 | KLHL18 | 0.81 | 7.79E-06 | 1.71E-04 |
| Proser2 | PROSER2 | 0.81 | 5.91E-06 | 1.37E-04 |
| Pglyrp4 | PGLYRP4 | 0.81 | 1.77E-03 | 1.34E-02 |
| Lacc1 | LACC1 | 0.82 | 5.07E-04 | 5.05E-03 |
| Pnpla3 | PNPLA3 | 0.82 | 3.76E-03 | 2.43E-02 |
| Sh3kbp1 | SH3KBP1 | 0.82 | 4.40E-06 | 1.08E-04 |
| Inpp5d | INPP5D | 0.82 | 2.87E-03 | 1.97E-02 |
| Sh3bgrl3 | SH3BGRL3 | 0.82 | 2.56E-07 | 1.10E-05 |
| Klf6 | KLF6 | 0.82 | 1.81E-06 | 5.35E-05 |
| Klrg2 | KLRG2 | 0.82 | 4.92E-03 | 2.99E-02 |
| Fam83g | FAM83G | 0.82 | 6.58E-06 | 1.49E-04 |
| Ifi203 | NA | 0.82 | 1.23E-04 | 1.64E-03 |
| Inf2 | INF2 | 0.82 | 1.12E-03 | 9.55E-03 |
| Pla2g4f | PLA2G4F | 0.82 | 2.91E-04 | 3.22E-03 |
| Btk | BTK | 0.82 | 8.76E-03 | 4.62E-02 |
| Ppm1d | PPM1D | 0.82 | 1.73E-07 | 8.04E-06 |
| Wnt10a | WNT10A | 0.82 | 5.93E-04 | 5.70E-03 |
| Kalrn | KALRN | 0.82 | 3.33E-07 | 1.38E-05 |
| Gna15 | GNA15 | 0.82 | 1.09E-05 | 2.24E-04 |
| Grhl3 | GRHL3 | 0.82 | 3.55E-06 | 9.07E-05 |
| Yod1 | YOD1 | 0.83 | 6.02E-05 | 9.18E-04 |
| Fam135a | FAM135A | 0.83 | 1.63E-05 | 3.14E-04 |
| E2f8 | E2F8 | 0.83 | 1.38E-03 | 1.11E-02 |
| Msmo1 | MSMO1 | 0.83 | 1.27E-06 | 4.07E-05 |
| Ncapg2 | NCAPG2 | 0.83 | 1.24E-03 | 1.03E-02 |
| Nfam1 | NFAM1 | 0.83 | 6.86E-03 | 3.81E-02 |
| Ctsd | CTSD | 0.83 | 8.07E-05 | 1.17E-03 |
| Osr1 | OSR1 | 0.83 | 4.45E-05 | 7.06E-04 |
| Ppfia3 | PPFIA3 | 0.83 | 2.82E-04 | 3.14E-03 |
| Slfn5 | SLFN5 | 0.84 | 2.05E-04 | 2.45E-03 |
| Tnf | TNF | 0.84 | 6.45E-03 | 3.65E-02 |
| Rnf39 | RNF39 | 0.84 | 1.79E-05 | 3.38E-04 |
| Acat2 | ACAT2 | 0.84 | 5.29E-05 | 8.21E-04 |
| Tmprss11e | TMPRSS11E | 0.84 | 1.81E-04 | 2.21E-03 |
| Spc25 | SPC25 | 0.84 | 2.80E-03 | 1.93E-02 |
| Emp3 | EMP3 | 0.84 | 1.57E-03 | 1.23E-02 |
| Scrn2 | SCRN2 | 0.84 | 1.28E-03 | 1.05E-02 |
| Rusc2 | RUSC2 | 0.85 | 5.83E-03 | 3.40E-02 |
| Clca5 | NA | 0.85 | 5.15E-07 | 1.95E-05 |
| Prrg4 | PRRG4 | 0.85 | 1.39E-06 | 4.32E-05 |
| Metrnl | METRNL | 0.85 | 1.28E-05 | 2.56E-04 |
| Mid1ip1 | MID1IP1 | 0.85 | 2.31E-07 | 1.03E-05 |
| Klf2 | KLF2 | 0.85 | 1.89E-03 | 1.41E-02 |
| Nrarp | NRARP | 0.85 | 1.67E-07 | 7.77E-06 |
| Zswim4 | ZSWIM4 | 0.85 | 6.41E-06 | 1.45E-04 |
| Fermt3 | FERMT3 | 0.86 | 3.65E-04 | 3.91E-03 |
| Hells | HELLS | 0.86 | 3.64E-03 | 2.37E-02 |
| Atp13a4 | ATP13A4 | 0.86 | 1.83E-03 | 1.37E-02 |
| Evpl | EVPL | 0.86 | 8.52E-06 | 1.83E-04 |
| Gba | GBA | 0.86 | 1.33E-06 | 4.22E-05 |
| Gjb4 | GJB4 | 0.86 | 3.17E-06 | 8.36E-05 |
| Ace2 | ACE2 | 0.86 | 5.46E-04 | 5.34E-03 |
| Snrk | SNRK | 0.86 | 4.84E-07 | 1.86E-05 |
| Duox1 | DUOX1 | 0.87 | 5.08E-04 | 5.06E-03 |
| Zeb2 | ZEB2 | 0.87 | 5.39E-04 | 5.28E-03 |
| Msn | MSN | 0.87 | 4.75E-06 | 1.15E-04 |
| Cldn4 | CLDN4 | 0.87 | 3.26E-06 | 8.50E-05 |
| Plk2 | PLK2 | 0.87 | 2.54E-07 | 1.10E-05 |
| Ak2 | AK2 | 0.87 | 6.37E-07 | 2.29E-05 |
| Gm8801 | NA | 0.87 | 4.24E-03 | 2.66E-02 |
| Nqo1 | NQO1 | 0.87 | 1.50E-06 | 4.59E-05 |
| Spns2 | SPNS2 | 0.87 | 7.95E-06 | 1.73E-04 |
| Epn3 | EPN3 | 0.87 | 9.98E-06 | 2.09E-04 |
| Hopx | HOPX | 0.87 | 3.33E-06 | 8.62E-05 |
| Trpv2 | TRPV2 | 0.88 | 3.91E-03 | 2.50E-02 |
| Phlda1 | PHLDA1 | 0.88 | 5.26E-06 | 1.24E-04 |
| Iqgap3 | IQGAP3 | 0.88 | 4.00E-03 | 2.55E-02 |
| Pla2g4e | PLA2G4E | 0.88 | 1.73E-04 | 2.13E-03 |
| Selplg | SELPLG | 0.88 | 7.57E-03 | 4.12E-02 |
| Susd1 | SUSD1 | 0.88 | 3.06E-03 | 2.08E-02 |
| Gpsm3 | GPSM3 | 0.88 | 8.33E-03 | 4.44E-02 |
| Dhrs1 | DHRS1 | 0.88 | 2.78E-06 | 7.58E-05 |
| Syt16 | SYT16 | 0.88 | 1.84E-05 | 3.45E-04 |
| Vasn | VASN | 0.88 | 3.03E-05 | 5.20E-04 |
| Lss | LSS | 0.88 | 1.44E-04 | 1.86E-03 |
| Pkp3 | PKP3 | 0.88 | 3.71E-08 | 2.28E-06 |
| Hck | HCK | 0.89 | 7.34E-04 | 6.78E-03 |
| Fam212b | FAM212B | 0.89 | 2.77E-05 | 4.82E-04 |
| Hyal1 | HYAL1 | 0.89 | 4.15E-04 | 4.32E-03 |
| Tmem40 | TMEM40 | 0.89 | 1.09E-05 | 2.24E-04 |
| Cda | CDA | 0.89 | 4.06E-03 | 2.58E-02 |
| Rpl36 | RPL36 | 0.89 | 1.93E-04 | 2.33E-03 |
| Ttc22 | TTC22 | 0.89 | 6.57E-06 | 1.49E-04 |
| Mthfd1l | MTHFD1L | 0.89 | 1.57E-03 | 1.23E-02 |
| Abtb2 | ABTB2 | 0.89 | 1.01E-05 | 2.12E-04 |
| Ccrn4l | NA | 0.89 | 4.81E-06 | 1.16E-04 |
| Bnc1 | BNC1 | 0.89 | 1.90E-04 | 2.29E-03 |
| Slc15a1 | SLC15A1 | 0.89 | 1.34E-05 | 2.66E-04 |
| Cd84 | CD84 | 0.89 | 1.13E-03 | 9.55E-03 |
| Cln8 | CLN8 | 0.89 | 3.40E-07 | 1.41E-05 |
| Stom | STOM | 0.89 | 4.35E-06 | 1.08E-04 |
| Ecm1 | ECM1 | 0.90 | 1.85E-04 | 2.25E-03 |
| Crisp1 | NA | 0.90 | 8.95E-03 | 4.70E-02 |
| Fam188a | FAM188A | 0.90 | 1.36E-10 | 1.97E-08 |
| Ovol1 | OVOL1 | 0.90 | 2.22E-06 | 6.30E-05 |
| Dedd2 | DEDD2 | 0.90 | 2.49E-07 | 1.08E-05 |
| Tmem45b | TMEM45B | 0.90 | 3.09E-04 | 3.39E-03 |
| Ada | ADA | 0.90 | 1.67E-03 | 1.29E-02 |
| Rbp1 | RBP1 | 0.90 | 1.08E-04 | 1.47E-03 |
| Ide | IDE | 0.90 | 1.53E-04 | 1.95E-03 |
| Apobr | APOBR | 0.90 | 2.06E-04 | 2.46E-03 |
| Gba2 | GBA2 | 0.90 | 8.96E-04 | 7.91E-03 |
| Oasl1 | OASL | 0.91 | 1.78E-04 | 2.18E-03 |
| P2ry4 | P2RY4 | 0.91 | 6.98E-03 | 3.86E-02 |
| Ninj1 | NINJ1 | 0.91 | 5.53E-04 | 5.39E-03 |
| Slc25a25 | SLC25A25 | 0.91 | 1.84E-06 | 5.44E-05 |
| Birc5 | BIRC5 | 0.91 | 4.42E-03 | 2.75E-02 |
| Adh6a | ADH6 | 0.91 | 2.69E-05 | 4.71E-04 |
| Abhd11os | NA | 0.91 | 2.56E-03 | 1.80E-02 |
| Mpzl3 | MPZL3 | 0.91 | 1.82E-07 | 8.38E-06 |
| Mvd | MVD | 0.91 | 6.59E-04 | 6.22E-03 |
| Fam213b | FAM213B | 0.91 | 2.65E-04 | 2.98E-03 |
| Dgat2 | DGAT2 | 0.91 | 3.27E-06 | 8.50E-05 |
| Cd93 | CD93 | 0.92 | 4.33E-03 | 2.71E-02 |
| Cd80 | CD80 | 0.92 | 7.26E-04 | 6.73E-03 |
| Smpdl3b | SMPDL3B | 0.92 | 2.24E-04 | 2.62E-03 |
| Psca | PSCA | 0.92 | 3.58E-03 | 2.34E-02 |
| Tyrobp | TYROBP | 0.92 | 2.69E-04 | 3.02E-03 |
| Lpin3 | LPIN3 | 0.92 | 7.65E-06 | 1.68E-04 |
| Aaas | AAAS | 0.92 | 1.30E-06 | 4.14E-05 |
| Anxa9 | ANXA9 | 0.92 | 1.14E-04 | 1.54E-03 |
| Plk1 | PLK1 | 0.92 | 4.41E-03 | 2.74E-02 |
| Il34 | IL34 | 0.92 | 7.15E-04 | 6.65E-03 |
| Elovl7 | ELOVL7 | 0.92 | 3.48E-05 | 5.80E-04 |
| Tiparp | TIPARP | 0.92 | 2.96E-09 | 2.72E-07 |
| St6gal1 | ST6GAL1 | 0.92 | 7.07E-03 | 3.90E-02 |
| Ccdc64b | BICDL2 | 0.92 | 1.67E-07 | 7.77E-06 |
| Mfi2 | MELTF | 0.92 | 1.12E-03 | 9.54E-03 |
| Tnfaip3 | TNFAIP3 | 0.93 | 3.38E-05 | 5.68E-04 |
| Gda | GDA | 0.93 | 1.96E-06 | 5.69E-05 |
| Dbi | DBI | 0.93 | 3.43E-06 | 8.83E-05 |
| Tmem54 | TMEM54 | 0.93 | 1.85E-06 | 5.44E-05 |
| Egfl6 | EGFL6 | 0.93 | 6.42E-03 | 3.64E-02 |
| Dsg1a | DSG1 | 0.93 | 2.17E-04 | 2.55E-03 |
| Ppard | PPARD | 0.93 | 8.89E-08 | 4.67E-06 |
| Cyp1b1 | CYP1B1 | 0.93 | 6.13E-05 | 9.31E-04 |
| Ctnnbip1 | CTNNBIP1 | 0.93 | 2.61E-10 | 3.53E-08 |
| Tnfrsf23 | NA | 0.93 | 1.57E-08 | 1.13E-06 |
| Trim30a | TRIM5 | 0.94 | 5.71E-04 | 5.54E-03 |
| Ifi204 | IFI16 | 0.94 | 3.83E-04 | 4.06E-03 |
| Pstpip1 | PSTPIP1 | 0.94 | 1.01E-04 | 1.39E-03 |
| Hrnr | HRNR | 0.94 | 6.15E-04 | 5.89E-03 |
| Cd86 | CD86 | 0.94 | 1.03E-03 | 8.87E-03 |
| Afap1l2 | AFAP1L2 | 0.94 | 8.14E-05 | 1.18E-03 |
| Cyp51 | CYP51A1 | 0.94 | 5.08E-07 | 1.93E-05 |
| Krt5 | KRT5 | 0.95 | 4.09E-05 | 6.57E-04 |
| Idi1 | IDI1 | 0.95 | 3.16E-06 | 8.35E-05 |
| Esyt3 | ESYT3 | 0.95 | 7.39E-06 | 1.64E-04 |
| Mif | MIF | 0.95 | 4.07E-06 | 1.02E-04 |
| Apbb1ip | APBB1IP | 0.95 | 3.78E-03 | 2.44E-02 |
| Clec7a | CLEC7A | 0.95 | 2.84E-03 | 1.95E-02 |
| Dmxl2 | DMXL2 | 0.96 | 4.37E-03 | 2.72E-02 |
| Ttk | TTK | 0.96 | 8.03E-03 | 4.31E-02 |
| Eda2r | EDA2R | 0.96 | 1.67E-05 | 3.20E-04 |
| Blm | BLM | 0.96 | 3.44E-03 | 2.26E-02 |
| Trerf1 | NA | 0.96 | 4.87E-03 | 2.96E-02 |
| Cers3 | CERS3 | 0.96 | 1.16E-08 | 8.91E-07 |
| Lipk | LIPK | 0.96 | 1.30E-03 | 1.06E-02 |
| Cd109 | CD109 | 0.96 | 2.22E-11 | 4.46E-09 |
| Ankrd9 | ANKRD9 | 0.96 | 2.81E-03 | 1.93E-02 |
| Ugt1a7c | UGT1A6 | 0.96 | 1.87E-04 | 2.27E-03 |
| Plekho2 | PLEKHO2 | 0.96 | 1.09E-03 | 9.30E-03 |
| Kif15 | KIF15 | 0.96 | 7.95E-03 | 4.28E-02 |
| Hsd17b2 | HSD17B2 | 0.96 | 3.46E-08 | 2.17E-06 |
| Ccne2 | CCNE2 | 0.96 | 2.45E-03 | 1.74E-02 |
| Ehd1 | EHD1 | 0.96 | 2.23E-08 | 1.50E-06 |
| Dpep2 | DPEP2 | 0.96 | 1.14E-03 | 9.61E-03 |
| Kif18b | KIF18B | 0.96 | 7.92E-03 | 4.27E-02 |
| Plcxd2 | PLCXD2 | 0.97 | 1.81E-05 | 3.40E-04 |
| Cpne2 | CPNE2 | 0.97 | 1.81E-04 | 2.21E-03 |
| Ceacam19 | CEACAM19 | 0.97 | 2.02E-04 | 2.42E-03 |
| Fdps | FDPS | 0.97 | 2.89E-06 | 7.77E-05 |
| Gpld1 | GPLD1 | 0.97 | 1.21E-04 | 1.62E-03 |
| Itgb2 | ITGB2 | 0.97 | 9.07E-04 | 8.00E-03 |
| 2210407C18Rik | NA | 0.97 | 7.27E-05 | 1.07E-03 |
| Nrros | NRROS | 0.97 | 8.97E-03 | 4.70E-02 |
| Snrnp25 | SNRNP25 | 0.98 | 2.18E-05 | 4.00E-04 |
| Basp1 | BASP1 | 0.98 | 6.28E-03 | 3.58E-02 |
| Smox | SMOX | 0.98 | 6.38E-07 | 2.29E-05 |
| Ankk1 | ANKK1 | 0.98 | 1.08E-05 | 2.23E-04 |
| Pik3cd | PIK3CD | 0.98 | 4.83E-03 | 2.94E-02 |
| Cd68 | CD68 | 0.98 | 1.78E-06 | 5.29E-05 |
| Rnf208 | RNF208 | 0.98 | 9.17E-05 | 1.30E-03 |
| Gm94 | C5orf46 | 0.98 | 6.27E-05 | 9.49E-04 |
| Tbc1d24 | NTN3 | 0.98 | 5.93E-04 | 5.70E-03 |
| Polr3g | POLR3G | 0.98 | 1.47E-05 | 2.86E-04 |
| Fam214b | FAM214B | 0.98 | 1.24E-09 | 1.27E-07 |
| Fam110a | FAM110A | 0.99 | 2.47E-06 | 6.86E-05 |
| Slc46a2 | SLC46A2 | 0.99 | 9.50E-03 | 4.90E-02 |
| 5430427O19Rik | CXorf21 | 0.99 | 8.93E-03 | 4.69E-02 |
| Slc19a2 | SLC19A2 | 0.99 | 1.42E-06 | 4.40E-05 |
| Sh3tc2 | SH3TC2 | 0.99 | 3.89E-04 | 4.11E-03 |
| Syk | SYK | 0.99 | 9.22E-05 | 1.30E-03 |
| Tubb6 | TUBB6 | 0.99 | 3.13E-05 | 5.38E-04 |
| Tm7sf2 | TM7SF2 | 0.99 | 3.66E-04 | 3.91E-03 |
| Ube2c | UBE2C | 0.99 | 1.50E-04 | 1.92E-03 |
| Ccbl1 | NA | 0.99 | 3.73E-05 | 6.09E-04 |
| Esco2 | ESCO2 | 0.99 | 8.96E-03 | 4.70E-02 |
| Ptprh | PTPRH | 1.00 | 1.31E-03 | 1.06E-02 |
| Pik3ap1 | PIK3AP1 | 1.00 | 9.39E-03 | 4.87E-02 |
| Upp1 | UPP1 | 1.00 | 3.85E-05 | 6.27E-04 |
| Pmaip1 | PMAIP1 | 1.00 | 4.07E-07 | 1.62E-05 |
| Homer3 | HOMER3 | 1.00 | 1.12E-04 | 1.52E-03 |
| Sipa1l2 | SIPA1L2 | 1.00 | 3.37E-05 | 5.67E-04 |
| Clec4a2 | CLEC4A | 1.01 | 6.68E-03 | 3.75E-02 |
| Egr1 | EGR1 | 1.01 | 8.91E-04 | 7.89E-03 |
| Fxyd5 | FXYD5 | 1.01 | 6.81E-04 | 6.38E-03 |
| Mndal | IFI16 | 1.01 | 2.66E-03 | 1.85E-02 |
| Ccr5 | CCR2 | 1.02 | 3.86E-03 | 2.48E-02 |
| Aldh3a1 | ALDH3A1 | 1.02 | 3.30E-06 | 8.57E-05 |
| Mfsd2a | MFSD2A | 1.02 | 5.87E-04 | 5.67E-03 |
| Gmfg | GMFG | 1.02 | 6.17E-05 | 9.36E-04 |
| Gpr35 | GPR35 | 1.02 | 1.66E-03 | 1.28E-02 |
| Myo1f | MYO1F | 1.02 | 5.07E-04 | 5.05E-03 |
| Mreg | MREG | 1.02 | 1.06E-07 | 5.46E-06 |
| Emp1 | EMP1 | 1.02 | 8.07E-06 | 1.76E-04 |
| Cdca8 | CDCA8 | 1.02 | 4.66E-04 | 4.73E-03 |
| Kctd11 | KCTD11 | 1.02 | 2.94E-08 | 1.88E-06 |
| Rac2 | RAC2 | 1.02 | 9.31E-04 | 8.18E-03 |
| Lcp2 | LCP2 | 1.02 | 3.31E-03 | 2.21E-02 |
| Ugcg | UGCG | 1.03 | 1.14E-06 | 3.74E-05 |
| 2310002J15Rik | NA | 1.03 | 4.24E-05 | 6.77E-04 |
| Vsig10l | VSIG10L | 1.03 | 5.98E-07 | 2.18E-05 |
| Mvk | MVK | 1.03 | 1.65E-04 | 2.05E-03 |
| Brca1 | BRCA1 | 1.03 | 5.53E-04 | 5.39E-03 |
| Ccl17 | CCL17 | 1.03 | 8.09E-03 | 4.34E-02 |
| Aox4 | NA | 1.03 | 1.02E-03 | 8.80E-03 |
| Cdc45 | CDC45 | 1.03 | 2.66E-03 | 1.86E-02 |
| Rassf1 | RASSF1 | 1.03 | 1.20E-07 | 6.08E-06 |
| Rnf223 | RNF223 | 1.04 | 4.84E-04 | 4.88E-03 |
| 2510049J12Rik | NA | 1.04 | 8.30E-04 | 7.50E-03 |
| Cxcl14 | CXCL14 | 1.04 | 1.95E-03 | 1.45E-02 |
| Cebpd | CEBPD | 1.04 | 4.92E-06 | 1.18E-04 |
| Ceacam1 | CEACAM8 | 1.04 | 1.54E-08 | 1.12E-06 |
| Rgcc | RGCC | 1.04 | 2.74E-04 | 3.06E-03 |
| Zfp385a | ZNF385A | 1.04 | 2.23E-06 | 6.33E-05 |
| Tprg | TPRG1 | 1.05 | 9.55E-07 | 3.21E-05 |
| Pmvk | PMVK | 1.05 | 5.04E-06 | 1.20E-04 |
| Gpr183 | GPR183 | 1.05 | 1.41E-03 | 1.13E-02 |
| Plcxd1 | PLCXD1 | 1.05 | 5.25E-03 | 3.13E-02 |
| Fos | FOS | 1.05 | 2.47E-04 | 2.83E-03 |
| Casp14 | CASP14 | 1.05 | 5.80E-04 | 5.61E-03 |
| Il2rg | IL2RG | 1.05 | 2.61E-04 | 2.95E-03 |
| Frmpd1 | FRMPD1 | 1.05 | 8.82E-07 | 3.03E-05 |
| A530032D15Rik | SP140 | 1.05 | 8.65E-03 | 4.57E-02 |
| Hsd17b7 | HSD17B7 | 1.05 | 4.07E-08 | 2.42E-06 |
| Tnfrsf1b | TNFRSF1B | 1.05 | 9.50E-05 | 1.33E-03 |
| Agpat4 | AGPAT4 | 1.06 | 4.80E-04 | 4.85E-03 |
| Dusp13 | DUSP13 | 1.06 | 3.48E-03 | 2.29E-02 |
| 2310005G13Rik | NA | 1.06 | 9.83E-04 | 8.56E-03 |
| Klk11 | KLK11 | 1.06 | 5.74E-09 | 4.82E-07 |
| Crct1 | NA | 1.06 | 1.27E-06 | 4.07E-05 |
| Gsta2 | NA | 1.06 | 4.94E-05 | 7.71E-04 |
| Arntl2 | ARNTL2 | 1.06 | 5.27E-10 | 6.17E-08 |
| Tigit | TIGIT | 1.06 | 2.57E-05 | 4.53E-04 |
| 4632434I11Rik | NA | 1.06 | 8.77E-05 | 1.25E-03 |
| Elf5 | ELF5 | 1.07 | 4.13E-07 | 1.64E-05 |
| Lipm | LIPM | 1.07 | 1.14E-06 | 3.75E-05 |
| Ptgs2 | PTGS2 | 1.07 | 2.35E-05 | 4.27E-04 |
| Coro1a | CORO1A | 1.07 | 1.14E-04 | 1.54E-03 |
| Otub2 | OTUB2 | 1.07 | 2.90E-05 | 5.02E-04 |
| Hilpda | HILPDA | 1.08 | 1.13E-04 | 1.53E-03 |
| Slc15a3 | SLC15A3 | 1.08 | 3.32E-03 | 2.21E-02 |
| Plekhn1 | PLEKHN1 | 1.08 | 6.01E-07 | 2.18E-05 |
| Csf2rb | CSF2RB | 1.08 | 9.72E-04 | 8.49E-03 |
| Lyg1 | LYG1 | 1.08 | 1.46E-03 | 1.16E-02 |
| Zc3h12a | ZC3H12A | 1.08 | 2.27E-06 | 6.39E-05 |
| Mlkl | MLKL | 1.08 | 5.51E-05 | 8.51E-04 |
| Spi1 | SPI1 | 1.08 | 1.01E-03 | 8.77E-03 |
| Dynap | NA | 1.08 | 4.54E-06 | 1.11E-04 |
| Krt80 | KRT80 | 1.09 | 3.71E-08 | 2.28E-06 |
| Ctsl | CTSV | 1.09 | 5.81E-05 | 8.90E-04 |
| Il1rl1 | IL1RL1 | 1.09 | 8.99E-03 | 4.71E-02 |
| Lilra6 | LILRA1 | 1.09 | 1.40E-04 | 1.82E-03 |
| E2f1 | E2F1 | 1.09 | 1.23E-03 | 1.02E-02 |
| Pira6 | NA | 1.10 | 3.42E-03 | 2.26E-02 |
| Slc16a3 | SLC16A3 | 1.10 | 6.97E-04 | 6.50E-03 |
| Dhfr | DHFR | 1.10 | 1.63E-04 | 2.04E-03 |
| Clec2g | CLEC2D | 1.10 | 1.15E-06 | 3.75E-05 |
| Arhgef37 | ARHGEF37 | 1.10 | 6.83E-06 | 1.53E-04 |
| Cyp4f39 | CYP4F22 | 1.10 | 3.71E-07 | 1.52E-05 |
| Stx11 | STX11 | 1.10 | 2.10E-04 | 2.49E-03 |
| Chst11 | CHST11 | 1.11 | 1.06E-04 | 1.46E-03 |
| Nfkbid | NFKBID | 1.11 | 2.96E-03 | 2.02E-02 |
| B3galt5 | B3GALT5 | 1.11 | 3.18E-06 | 8.37E-05 |
| Cyp2c65 | CYP2C8 | 1.11 | 5.99E-04 | 5.75E-03 |
| Tgm3 | TGM3 | 1.11 | 4.84E-06 | 1.16E-04 |
| Ccne1 | CCNE1 | 1.11 | 1.90E-04 | 2.29E-03 |
| Fcer1g | FCER1G | 1.12 | 2.27E-04 | 2.65E-03 |
| Mt4 | MT4 | 1.12 | 3.33E-05 | 5.63E-04 |
| Ifi202b | NA | 1.12 | 1.03E-09 | 1.08E-07 |
| Rdh12 | RDH12 | 1.12 | 7.75E-05 | 1.13E-03 |
| Iffo2 | IFFO2 | 1.12 | 3.21E-10 | 4.18E-08 |
| Mfsd12 | MFSD12 | 1.12 | 1.41E-03 | 1.13E-02 |
| Cited4 | CITED4 | 1.12 | 2.55E-04 | 2.90E-03 |
| Kcnab2 | KCNAB2 | 1.12 | 6.23E-04 | 5.95E-03 |
| Krt4 | KRT4 | 1.12 | 3.68E-05 | 6.04E-04 |
| Rnf224 | RNF224 | 1.12 | 1.04E-03 | 8.95E-03 |
| Lrrc25 | LRRC25 | 1.13 | 4.91E-03 | 2.98E-02 |
| Pirb | LILRA2 | 1.13 | 3.85E-05 | 6.27E-04 |
| Cd300lb | CD300LB | 1.13 | 2.77E-03 | 1.91E-02 |
| Ccr1 | CCR1 | 1.13 | 9.91E-04 | 8.61E-03 |
| C130026I21Rik | SP140 | 1.13 | 3.73E-07 | 1.52E-05 |
| Ndrg1 | NDRG1 | 1.13 | 3.90E-07 | 1.57E-05 |
| Thop1 | THOP1 | 1.13 | 2.56E-07 | 1.10E-05 |
| Tnip3 | TNIP3 | 1.13 | 6.28E-04 | 5.99E-03 |
| Ncf2 | NCF2 | 1.14 | 2.89E-04 | 3.20E-03 |
| Il1rn | IL1RN | 1.14 | 1.44E-04 | 1.86E-03 |
| Procr | PROCR | 1.14 | 2.96E-12 | 7.50E-10 |
| Gp1bb | GP1BB | 1.14 | 9.53E-03 | 4.92E-02 |
| Slc16a6 | SLC16A6 | 1.14 | 2.10E-07 | 9.43E-06 |
| Bcar3 | BCAR3 | 1.14 | 2.17E-11 | 4.40E-09 |
| Ndufa4l2 | NDUFA4L2 | 1.14 | 1.84E-04 | 2.24E-03 |
| Ly6g6c | LY6G6C | 1.14 | 1.63E-06 | 4.91E-05 |
| Uhrf1 | UHRF1 | 1.14 | 3.69E-05 | 6.05E-04 |
| 9530053A07Rik | FCGBP | 1.14 | 1.08E-03 | 9.27E-03 |
| Slc25a48 | SLC25A48 | 1.15 | 1.57E-04 | 1.98E-03 |
| Etnk2 | ETNK2 | 1.15 | 1.02E-03 | 8.80E-03 |
| Mybl1 | MYBL1 | 1.15 | 1.03E-05 | 2.16E-04 |
| Col18a1 | COL18A1 | 1.15 | 1.89E-03 | 1.41E-02 |
| Fut4 | FUT4 | 1.15 | 6.97E-07 | 2.47E-05 |
| Fas | FAS | 1.16 | 4.61E-06 | 1.12E-04 |
| Unc93a | UNC93A | 1.16 | 3.06E-03 | 2.08E-02 |
| Ppp1r18 | PPP1R18 | 1.16 | 4.11E-08 | 2.43E-06 |
| Fam20c | FAM20C | 1.16 | 9.46E-06 | 2.00E-04 |
| 2610203C22Rik | NA | 1.16 | 1.25E-04 | 1.66E-03 |
| Ccdc88b | CCDC88B | 1.16 | 1.83E-03 | 1.37E-02 |
| Cxcr4 | CXCR4 | 1.16 | 9.77E-04 | 8.52E-03 |
| AB124611 | C19orf38 | 1.16 | 2.69E-04 | 3.02E-03 |
| Fam20a | FAM20A | 1.16 | 2.81E-06 | 7.64E-05 |
| Calm4 | CALML5 | 1.16 | 3.50E-05 | 5.82E-04 |
| Gjb2 | GJB2 | 1.16 | 1.33E-07 | 6.59E-06 |
| Tgm5 | TGM5 | 1.16 | 5.16E-09 | 4.45E-07 |
| Lce3f | NA | 1.17 | 9.21E-06 | 1.96E-04 |
| Fam57a | FAM57A | 1.17 | 2.35E-06 | 6.59E-05 |
| Klf4 | KLF4 | 1.17 | 8.29E-11 | 1.33E-08 |
| Plekhg1 | PLEKHG1 | 1.17 | 2.03E-11 | 4.18E-09 |
| Cfp | CFP | 1.17 | 1.07E-04 | 1.46E-03 |
| Psapl1 | PSAPL1 | 1.17 | 2.72E-07 | 1.15E-05 |
| Ccl6 | CCL23 | 1.17 | 1.12E-06 | 3.70E-05 |
| Csf2rb2 | CSF2RB | 1.18 | 1.65E-04 | 2.05E-03 |
| Cldn17 | CLDN17 | 1.18 | 3.82E-04 | 4.05E-03 |
| Sim2 | SIM2 | 1.18 | 1.11E-04 | 1.51E-03 |
| Mcm5 | MCM5 | 1.18 | 1.70E-05 | 3.24E-04 |
| Ubash3b | UBASH3B | 1.18 | 1.72E-04 | 2.12E-03 |
| Ltb4r1 | LTB4R | 1.18 | 1.49E-04 | 1.91E-03 |
| Acer1 | ACER1 | 1.18 | 3.28E-04 | 3.57E-03 |
| Atp6v0a4 | ATP6V0A4 | 1.18 | 2.36E-05 | 4.27E-04 |
| Dsg3 | DSG3 | 1.18 | 1.14E-10 | 1.70E-08 |
| Trim40 | TRIM40 | 1.19 | 1.38E-03 | 1.11E-02 |
| Glipr1 | GLIPR1 | 1.19 | 1.04E-07 | 5.41E-06 |
| Gpr132 | GPR132 | 1.19 | 2.68E-03 | 1.87E-02 |
| Duoxa1 | DUOXA1 | 1.19 | 1.08E-08 | 8.41E-07 |
| Fscn1 | FSCN1 | 1.19 | 2.98E-06 | 7.98E-05 |
| Pxdc1 | PXDC1 | 1.19 | 3.49E-07 | 1.44E-05 |
| Lrp8 | LRP8 | 1.19 | 6.13E-06 | 1.41E-04 |
| Trem2 | TREM2 | 1.19 | 6.74E-03 | 3.77E-02 |
| Shf | SHF | 1.19 | 1.94E-04 | 2.33E-03 |
| Glrx | GLRX | 1.19 | 4.27E-07 | 1.68E-05 |
| Dusp7 | DUSP7 | 1.19 | 7.58E-12 | 1.77E-09 |
| Epha2 | EPHA2 | 1.20 | 4.70E-09 | 4.12E-07 |
| Slc28a2 | SLC28A2 | 1.20 | 4.96E-03 | 3.00E-02 |
| Tmprss11a | TMPRSS11A | 1.20 | 1.27E-06 | 4.07E-05 |
| Ankrd35 | ANKRD35 | 1.20 | 4.48E-06 | 1.10E-04 |
| Mxd1 | MXD1 | 1.20 | 3.01E-06 | 8.03E-05 |
| Bglap3 | BGLAP | 1.20 | 1.55E-05 | 3.01E-04 |
| Myo7a | MYO7A | 1.20 | 1.23E-03 | 1.02E-02 |
| Serpinb3a | SERPINB3 | 1.21 | 2.38E-04 | 2.75E-03 |
| Dsc1 | DSC1 | 1.21 | 1.36E-04 | 1.78E-03 |
| Tmprss11g | NA | 1.21 | 4.36E-08 | 2.54E-06 |
| Arid3a | ARID3A | 1.21 | 1.07E-03 | 9.20E-03 |
| 2810417H13Rik | KIAA0101 | 1.21 | 4.34E-04 | 4.48E-03 |
| Lgalsl | LGALSL | 1.21 | 8.27E-09 | 6.65E-07 |
| Sdr16c5 | SDR16C5 | 1.21 | 6.76E-04 | 6.35E-03 |
| Tk1 | TK1 | 1.21 | 1.69E-05 | 3.22E-04 |
| Zfp365 | ZNF365 | 1.21 | 4.04E-06 | 1.01E-04 |
| Nipal1 | NIPAL1 | 1.21 | 3.22E-10 | 4.18E-08 |
| Ms4a6d | MS4A6A | 1.22 | 2.11E-04 | 2.50E-03 |
| Nr1h5 | NA | 1.22 | 4.08E-03 | 2.59E-02 |
| Slc37a2 | SLC37A2 | 1.22 | 7.48E-08 | 4.01E-06 |
| Xkrx | XKRX | 1.22 | 1.49E-04 | 1.91E-03 |
| S100a7a | NA | 1.23 | 2.20E-08 | 1.49E-06 |
| Pla2g7 | PLA2G7 | 1.23 | 9.47E-06 | 2.00E-04 |
| Dmkn | DMKN | 1.23 | 2.45E-06 | 6.84E-05 |
| Fst | FST | 1.23 | 1.20E-05 | 2.42E-04 |
| Gpr115 | NA | 1.23 | 1.87E-05 | 3.50E-04 |
| Cd37 | CD37 | 1.23 | 6.80E-03 | 3.79E-02 |
| Il1f5 | IL36RN | 1.23 | 1.22E-04 | 1.63E-03 |
| Acsbg1 | ACSBG1 | 1.23 | 6.20E-08 | 3.38E-06 |
| Dusp5 | DUSP5 | 1.23 | 3.78E-06 | 9.58E-05 |
| Cd52 | NA | 1.23 | 3.31E-05 | 5.59E-04 |
| Klk7 | KLK7 | 1.23 | 1.69E-04 | 2.09E-03 |
| Tmprss11bnl | NA | 1.23 | 4.54E-11 | 8.25E-09 |
| Tmprss11f | TMPRSS11F | 1.23 | 1.76E-08 | 1.25E-06 |
| Bub1 | BUB1 | 1.24 | 1.97E-03 | 1.46E-02 |
| Ccrl2 | CCRL2 | 1.24 | 4.26E-04 | 4.41E-03 |
| Aloxe3 | ALOXE3 | 1.24 | 5.15E-07 | 1.95E-05 |
| Pla2g3 | PLA2G3 | 1.24 | 2.27E-06 | 6.39E-05 |
| B430306N03Rik | NA | 1.24 | 4.69E-03 | 2.87E-02 |
| Lce1a2 | NA | 1.24 | 2.74E-05 | 4.78E-04 |
| Ptpn22 | PTPN22 | 1.24 | 3.83E-04 | 4.05E-03 |
| Cidea | CIDEA | 1.24 | 3.82E-07 | 1.55E-05 |
| Lce3a | NA | 1.25 | 4.41E-06 | 1.08E-04 |
| Sp6 | SP6 | 1.25 | 1.02E-09 | 1.08E-07 |
| Sgpp2 | SGPP2 | 1.25 | 4.94E-04 | 4.96E-03 |
| Alox5ap | ALOX5AP | 1.26 | 1.26E-06 | 4.06E-05 |
| Csrnp1 | CSRNP1 | 1.26 | 2.46E-05 | 4.38E-04 |
| Wfdc5 | WFDC5 | 1.26 | 2.19E-07 | 9.84E-06 |
| Fmnl1 | FMNL1 | 1.26 | 5.82E-04 | 5.62E-03 |
| Slc35f2 | SLC35F2 | 1.26 | 7.40E-04 | 6.82E-03 |
| Sema7a | SEMA7A | 1.27 | 3.26E-04 | 3.56E-03 |
| Clec5a | CLEC5A | 1.27 | 1.06E-05 | 2.20E-04 |
| Fcgr3 | FCGR2A | 1.27 | 2.47E-06 | 6.86E-05 |
| Slc2a3 | SLC2A14 | 1.27 | 2.81E-03 | 1.93E-02 |
| Il1f8 | IL36B | 1.27 | 4.88E-05 | 7.66E-04 |
| Igfbp2 | IGFBP2 | 1.27 | 1.81E-08 | 1.28E-06 |
| Papl | NA | 1.27 | 4.80E-04 | 4.85E-03 |
| Prss22 | PRSS22 | 1.27 | 1.13E-06 | 3.71E-05 |
| Mdfi | MDFI | 1.27 | 7.85E-06 | 1.72E-04 |
| Vcan | VCAN | 1.28 | 2.13E-06 | 6.12E-05 |
| Flrt3 | FLRT3 | 1.28 | 8.61E-06 | 1.85E-04 |
| Capn12 | CAPN12 | 1.28 | 5.09E-04 | 5.07E-03 |
| Clmp | CLMP | 1.28 | 5.29E-04 | 5.20E-03 |
| Crabp2 | CRABP2 | 1.28 | 6.47E-07 | 2.31E-05 |
| Bcl2a1b | BCL2A1 | 1.28 | 3.93E-05 | 6.36E-04 |
| Emilin2 | EMILIN2 | 1.28 | 6.22E-04 | 5.95E-03 |
| Fam83a | FAM83A | 1.29 | 2.92E-06 | 7.85E-05 |
| Lce1a1 | NA | 1.29 | 3.94E-05 | 6.36E-04 |
| Gpsm1 | GPSM1 | 1.29 | 6.63E-07 | 2.36E-05 |
| Slc10a6 | SLC10A6 | 1.29 | 2.66E-04 | 3.00E-03 |
| Gpnmb | GPNMB | 1.29 | 1.35E-04 | 1.77E-03 |
| Abca12 | ABCA12 | 1.29 | 5.78E-06 | 1.34E-04 |
| Rrm2 | RRM2 | 1.29 | 3.23E-05 | 5.48E-04 |
| Sdr16c6 | NA | 1.30 | 4.07E-04 | 4.27E-03 |
| Serpinb12 | SERPINB12 | 1.30 | 1.64E-05 | 3.15E-04 |
| Sult2b1 | SULT2B1 | 1.30 | 5.10E-07 | 1.93E-05 |
| Csta1 | CSTA | 1.30 | 5.75E-08 | 3.19E-06 |
| Hmox1 | HMOX1 | 1.30 | 1.90E-10 | 2.64E-08 |
| Fam65c | FAM65C | 1.31 | 7.02E-05 | 1.04E-03 |
| Vsig8 | VSIG8 | 1.31 | 5.50E-07 | 2.06E-05 |
| Fam83d | FAM83D | 1.31 | 1.92E-06 | 5.61E-05 |
| Lat2 | LAT2 | 1.32 | 2.95E-05 | 5.09E-04 |
| Cyp26b1 | CYP26B1 | 1.32 | 5.35E-06 | 1.26E-04 |
| Ltb4r2 | LTB4R2 | 1.32 | 1.91E-03 | 1.42E-02 |
| Naip5 | NAIP | 1.33 | 9.50E-03 | 4.90E-02 |
| Cd33 | SIGLEC9 | 1.33 | 1.55E-04 | 1.96E-03 |
| Dnase1l2 | DNASE1L2 | 1.33 | 8.75E-05 | 1.25E-03 |
| Defb4 | NA | 1.33 | 1.06E-05 | 2.20E-04 |
| Cpt1c | CPT1C | 1.33 | 9.90E-06 | 2.08E-04 |
| Pyhin1 | NA | 1.34 | 1.38E-06 | 4.31E-05 |
| Kprp | KPRP | 1.34 | 1.80E-05 | 3.39E-04 |
| Ggct | GGCT | 1.34 | 9.17E-06 | 1.96E-04 |
| Map6 | MAP6 | 1.34 | 2.83E-06 | 7.68E-05 |
| Gm19557 | NA | 1.34 | 7.84E-04 | 7.18E-03 |
| Cd177 | CD177 | 1.34 | 4.98E-04 | 4.99E-03 |
| Map3k6 | MAP3K6 | 1.34 | 4.63E-07 | 1.81E-05 |
| Capns2 | CAPNS2 | 1.35 | 2.69E-07 | 1.14E-05 |
| Asprv1 | ASPRV1 | 1.35 | 6.33E-06 | 1.45E-04 |
| Cdkn1a | CDKN1A | 1.35 | 8.18E-15 | 4.86E-12 |
| Plat | PLAT | 1.35 | 1.66E-11 | 3.55E-09 |
| Abcb1b | ABCB1 | 1.36 | 1.10E-07 | 5.63E-06 |
| Rptn | RPTN | 1.36 | 3.66E-05 | 6.03E-04 |
| Fkbp5 | FKBP5 | 1.36 | 1.59E-09 | 1.52E-07 |
| Aif1l | AIF1L | 1.36 | 4.69E-10 | 5.65E-08 |
| Socs3 | SOCS3 | 1.36 | 3.03E-12 | 7.55E-10 |
| Nccrp1 | NCCRP1 | 1.37 | 5.82E-07 | 2.14E-05 |
| Slc7a11 | SLC7A11 | 1.37 | 1.23E-07 | 6.18E-06 |
| Krt13 | KRT13 | 1.37 | 2.24E-06 | 6.34E-05 |
| B4galnt4 | B4GALNT4 | 1.37 | 3.37E-03 | 2.23E-02 |
| Atg9b | ATG9B | 1.37 | 7.17E-10 | 7.96E-08 |
| Sirpb1a | SIRPG | 1.37 | 5.83E-03 | 3.40E-02 |
| Sh2d3c | SH2D3C | 1.37 | 2.29E-03 | 1.65E-02 |
| Pglyrp3 | PGLYRP3 | 1.37 | 4.31E-05 | 6.85E-04 |
| Ephx3 | EPHX3 | 1.37 | 6.20E-09 | 5.09E-07 |
| Gpa33 | GPA33 | 1.37 | 3.51E-04 | 3.78E-03 |
| Slpi | SLPI | 1.38 | 2.33E-06 | 6.54E-05 |
| Mcam | MCAM | 1.38 | 4.79E-07 | 1.85E-05 |
| Lce1k | NA | 1.38 | 2.37E-05 | 4.29E-04 |
| Aadacl2 | AADACL2 | 1.38 | 4.98E-06 | 1.19E-04 |
| Cxcr2 | CXCR2 | 1.38 | 2.92E-03 | 2.00E-02 |
| Pigz | PIGZ | 1.39 | 1.75E-04 | 2.15E-03 |
| 2610528A11Rik | C10orf99 | 1.39 | 3.94E-10 | 4.99E-08 |
| Gdpd3 | GDPD3 | 1.39 | 2.44E-07 | 1.07E-05 |
| Krtdap | KRTDAP | 1.39 | 1.91E-06 | 5.59E-05 |
| Snx20 | SNX20 | 1.39 | 8.19E-04 | 7.44E-03 |
| Bcl2l15 | BCL2L15 | 1.39 | 6.80E-05 | 1.02E-03 |
| Sdr9c7 | SDR9C7 | 1.40 | 1.44E-07 | 7.05E-06 |
| Marco | MARCO | 1.40 | 5.13E-03 | 3.08E-02 |
| Lipg | LIPG | 1.40 | 7.95E-03 | 4.28E-02 |
| Krt78 | KRT78 | 1.40 | 3.08E-06 | 8.18E-05 |
| Ush1g | USH1G | 1.40 | 1.57E-03 | 1.23E-02 |
| Nipal4 | NIPAL4 | 1.40 | 8.75E-07 | 3.01E-05 |
| Dusp1 | DUSP1 | 1.41 | 1.05E-07 | 5.43E-06 |
| Plek | PLEK | 1.41 | 7.58E-05 | 1.11E-03 |
| Plaur | PLAUR | 1.41 | 3.14E-11 | 5.91E-09 |
| Mab21l3 | MAB21L3 | 1.41 | 2.59E-04 | 2.94E-03 |
| Tnfrsf18 | TNFRSF18 | 1.41 | 4.08E-04 | 4.27E-03 |
| Il7r | IL7R | 1.41 | 3.66E-05 | 6.03E-04 |
| Lor | NA | 1.41 | 8.11E-08 | 4.33E-06 |
| Adam8 | ADAM8 | 1.42 | 4.71E-07 | 1.83E-05 |
| Srms | SRMS | 1.42 | 3.29E-03 | 2.20E-02 |
| Gpr111 | NA | 1.43 | 3.81E-04 | 4.04E-03 |
| Dsg1b | DSG1 | 1.43 | 9.45E-07 | 3.19E-05 |
| Il1b | IL1B | 1.43 | 1.00E-03 | 8.71E-03 |
| Klk8 | KLK8 | 1.43 | 5.47E-06 | 1.28E-04 |
| Atp1a3 | ATP1A3 | 1.44 | 8.19E-03 | 4.37E-02 |
| Lce3e | NA | 1.44 | 1.03E-07 | 5.36E-06 |
| 4930539E08Rik | C6orf222 | 1.44 | 2.62E-04 | 2.96E-03 |
| Psrc1 | PSRC1 | 1.44 | 5.94E-07 | 2.17E-05 |
| Serpine2 | SERPINE2 | 1.44 | 5.78E-13 | 2.07E-10 |
| Rbp2 | RBP2 | 1.44 | 2.59E-07 | 1.11E-05 |
| St6galnac4 | ST6GALNAC4 | 1.45 | 1.27E-05 | 2.54E-04 |
| Lce1b | NA | 1.45 | 1.50E-06 | 4.59E-05 |
| Cdc6 | CDC6 | 1.45 | 6.38E-06 | 1.45E-04 |
| Krt6a | NA | 1.45 | 2.62E-07 | 1.12E-05 |
| Ccl22 | CCL22 | 1.46 | 1.10E-04 | 1.50E-03 |
| Pnpla1 | PNPLA1 | 1.46 | 6.44E-06 | 1.46E-04 |
| Bpifc | BPIFC | 1.46 | 4.12E-06 | 1.03E-04 |
| Shcbp1 | SHCBP1 | 1.46 | 1.12E-04 | 1.53E-03 |
| Otop3 | OTOP3 | 1.46 | 2.93E-08 | 1.88E-06 |
| Them5 | THEM5 | 1.46 | 3.91E-08 | 2.37E-06 |
| Serpinb3d | SERPINB4 | 1.46 | 7.33E-04 | 6.78E-03 |
| Dlx3 | DLX3 | 1.46 | 5.74E-05 | 8.83E-04 |
| Milr1 | MILR1 | 1.47 | 5.84E-03 | 3.40E-02 |
| Klk10 | KLK10 | 1.47 | 4.62E-08 | 2.67E-06 |
| Lcn2 | LCN2 | 1.47 | 5.25E-04 | 5.18E-03 |
| Cd53 | CD53 | 1.47 | 8.59E-06 | 1.84E-04 |
| Krt14 | KRT14 | 1.47 | 3.40E-05 | 5.69E-04 |
| Tnfrsf9 | TNFRSF9 | 1.47 | 4.24E-04 | 4.39E-03 |
| Rgs1 | RGS1 | 1.47 | 1.77E-04 | 2.17E-03 |
| Ccl9 | CCL15 | 1.47 | 1.35E-06 | 4.26E-05 |
| Card14 | CARD14 | 1.48 | 1.36E-06 | 4.30E-05 |
| Sbsn | SBSN | 1.48 | 6.29E-09 | 5.14E-07 |
| Slc6a19 | SLC6A19 | 1.48 | 9.31E-06 | 1.98E-04 |
| Sfn | SFN | 1.48 | 2.46E-11 | 4.74E-09 |
| Mafb | MAFB | 1.48 | 1.02E-12 | 3.28E-10 |
| Rsad2 | RSAD2 | 1.49 | 2.12E-06 | 6.10E-05 |
| Gtse1 | GTSE1 | 1.49 | 4.37E-08 | 2.54E-06 |
| Ano3 | ANO3 | 1.49 | 9.52E-06 | 2.01E-04 |
| Fam131c | FAM131C | 1.49 | 1.25E-04 | 1.66E-03 |
| Tsc22d3 | TSC22D3 | 1.49 | 6.76E-13 | 2.36E-10 |
| Timeless | TIMELESS | 1.50 | 2.29E-04 | 2.67E-03 |
| Plcd4 | PLCD4 | 1.50 | 6.82E-03 | 3.80E-02 |
| Alox12b | ALOX12B | 1.50 | 4.73E-08 | 2.71E-06 |
| Ncf1 | NCF1 | 1.50 | 2.41E-07 | 1.06E-05 |
| Arg1 | ARG1 | 1.50 | 1.15E-07 | 5.86E-06 |
| Dnm3 | DNM3 | 1.50 | 1.78E-03 | 1.35E-02 |
| Ptk6 | PTK6 | 1.50 | 1.18E-05 | 2.39E-04 |
| Anxa10 | ANXA10 | 1.51 | 2.97E-05 | 5.13E-04 |
| Fam25c | FAM25A | 1.51 | 5.34E-11 | 9.47E-09 |
| Gm19510 | NA | 1.51 | 3.47E-03 | 2.28E-02 |
| Tgm1 | TGM1 | 1.51 | 2.79E-13 | 1.16E-10 |
| Trem1 | TREM1 | 1.51 | 2.04E-03 | 1.50E-02 |
| Efna3 | EFNA3 | 1.51 | 1.25E-09 | 1.27E-07 |
| Kctd4 | KCTD4 | 1.51 | 2.06E-05 | 3.81E-04 |
| C3ar1 | C3AR1 | 1.51 | 1.21E-04 | 1.62E-03 |
| Junb | JUNB | 1.51 | 1.13E-08 | 8.69E-07 |
| Ptafr | PTAFR | 1.51 | 6.32E-05 | 9.55E-04 |
| Prss12 | PRSS12 | 1.51 | 8.67E-08 | 4.57E-06 |
| Serpinb3b | SERPINB4 | 1.52 | 1.37E-04 | 1.78E-03 |
| Trex2 | TREX2 | 1.52 | 6.97E-08 | 3.75E-06 |
| Gsdma | GSDMA | 1.52 | 4.90E-07 | 1.88E-05 |
| Lce3d | NA | 1.52 | 1.11E-04 | 1.51E-03 |
| Elovl4 | ELOVL4 | 1.52 | 6.04E-09 | 4.98E-07 |
| Spp1 | SPP1 | 1.53 | 3.19E-04 | 3.49E-03 |
| Nkpd1 | NKPD1 | 1.53 | 6.76E-09 | 5.49E-07 |
| Cpa4 | CPA4 | 1.53 | 5.52E-06 | 1.29E-04 |
| Cst6 | CST6 | 1.53 | 3.20E-06 | 8.40E-05 |
| Smpd3 | SMPD3 | 1.54 | 4.17E-07 | 1.65E-05 |
| Lce1e | NA | 1.54 | 1.03E-06 | 3.41E-05 |
| 2310042E22Rik | NA | 1.54 | 5.09E-07 | 1.93E-05 |
| Arc | ARC | 1.54 | 6.58E-10 | 7.42E-08 |
| Gsta1 | NA | 1.55 | 7.08E-06 | 1.58E-04 |
| Lce1h | NA | 1.55 | 2.85E-05 | 4.94E-04 |
| Lypd3 | LYPD3 | 1.55 | 4.89E-10 | 5.85E-08 |
| Cmklr1 | CMKLR1 | 1.55 | 5.40E-08 | 3.04E-06 |
| Nlrp12 | NLRP12 | 1.55 | 6.37E-03 | 3.62E-02 |
| Il18rap | IL18RAP | 1.55 | 1.79E-06 | 5.30E-05 |
| Hbegf | HBEGF | 1.55 | 1.71E-14 | 9.77E-12 |
| Pitx1 | PITX1 | 1.55 | 5.25E-11 | 9.42E-09 |
| Sprr3 | SPRR3 | 1.56 | 2.25E-07 | 1.00E-05 |
| Rnf222 | RNF222 | 1.56 | 1.70E-06 | 5.12E-05 |
| Gjb6 | GJB6 | 1.56 | 1.15E-07 | 5.86E-06 |
| Arg2 | ARG2 | 1.56 | 1.16E-05 | 2.35E-04 |
| Fn1 | FN1 | 1.56 | 2.88E-06 | 7.77E-05 |
| Fgr | FGR | 1.56 | 1.05E-05 | 2.18E-04 |
| Lce3c | NA | 1.56 | 4.06E-08 | 2.42E-06 |
| Cpn1 | CPN1 | 1.57 | 1.01E-03 | 8.72E-03 |
| Fndc9 | FNDC9 | 1.57 | 8.85E-07 | 3.04E-05 |
| Ctgf | CTGF | 1.57 | 1.84E-14 | 1.02E-11 |
| Prss27 | PRSS27 | 1.58 | 4.33E-10 | 5.39E-08 |
| Fabp5 | FABP5 | 1.58 | 1.56E-08 | 1.13E-06 |
| Pinlyp | PINLYP | 1.58 | 6.09E-11 | 1.06E-08 |
| Clec2f | NA | 1.58 | 9.45E-04 | 8.29E-03 |
| Pla2g4d | PLA2G4D | 1.58 | 1.46E-05 | 2.86E-04 |
| Aldh1a3 | ALDH1A3 | 1.58 | 8.92E-11 | 1.42E-08 |
| Pla2r1 | PLA2R1 | 1.58 | 1.07E-05 | 2.20E-04 |
| Myl7 | MYL7 | 1.58 | 1.63E-07 | 7.75E-06 |
| Krt17 | KRT17 | 1.58 | 4.37E-07 | 1.72E-05 |
| Tmprss11d | TMPRSS11D | 1.58 | 2.40E-13 | 1.03E-10 |
| 2300002M23Rik | C6orf15 | 1.59 | 7.25E-09 | 5.86E-07 |
| Lce3b | NA | 1.59 | 3.65E-09 | 3.29E-07 |
| Bst1 | BST1 | 1.59 | 2.28E-08 | 1.53E-06 |
| Plk3 | PLK3 | 1.59 | 5.57E-13 | 2.07E-10 |
| Thsd1 | THSD1 | 1.60 | 3.22E-05 | 5.47E-04 |
| Hal | HAL | 1.60 | 6.10E-06 | 1.40E-04 |
| Egr3 | EGR3 | 1.61 | 1.49E-04 | 1.91E-03 |
| Per1 | PER1 | 1.61 | 1.34E-09 | 1.35E-07 |
| Cd200r1 | CD200R1 | 1.61 | 6.41E-04 | 6.09E-03 |
| Tlr13 | NA | 1.62 | 7.13E-06 | 1.58E-04 |
| Atf3 | ATF3 | 1.62 | 3.61E-09 | 3.28E-07 |
| Mcm10 | MCM10 | 1.63 | 3.58E-03 | 2.34E-02 |
| Ces2e | CES2 | 1.63 | 5.10E-10 | 6.06E-08 |
| Lce1d | NA | 1.63 | 5.62E-07 | 2.09E-05 |
| Igsf6 | IGSF6 | 1.64 | 2.97E-06 | 7.96E-05 |
| Snai1 | SNAI1 | 1.65 | 2.81E-03 | 1.93E-02 |
| Ramp3 | RAMP3 | 1.65 | 3.61E-03 | 2.35E-02 |
| Vstm5 | VSTM5 | 1.65 | 1.17E-05 | 2.37E-04 |
| Ankdd1b | ANKDD1B | 1.66 | 1.17E-06 | 3.80E-05 |
| Nr4a1 | NR4A1 | 1.66 | 3.33E-10 | 4.28E-08 |
| Mmp19 | MMP19 | 1.66 | 2.01E-06 | 5.81E-05 |
| Samsn1 | SAMSN1 | 1.67 | 1.00E-04 | 1.38E-03 |
| Mmp25 | MMP25 | 1.67 | 3.64E-03 | 2.37E-02 |
| Fpr1 | FPR1 | 1.67 | 5.60E-03 | 3.30E-02 |
| Celf5 | CELF5 | 1.67 | 2.34E-03 | 1.67E-02 |
| Lgals7 | LGALS7B | 1.67 | 1.97E-09 | 1.85E-07 |
| Mmp9 | MMP9 | 1.67 | 4.86E-04 | 4.89E-03 |
| Thbs2 | THBS2 | 1.67 | 3.94E-03 | 2.51E-02 |
| Slfn4 | SLFN12L | 1.68 | 5.77E-04 | 5.60E-03 |
| Sncg | SNCG | 1.68 | 2.45E-07 | 1.07E-05 |
| Ncf4 | NCF4 | 1.68 | 4.55E-08 | 2.64E-06 |
| Cdsn | CDSN | 1.68 | 3.20E-09 | 2.93E-07 |
| Cdc7 | CDC7 | 1.69 | 2.93E-04 | 3.24E-03 |
| Tnfrsf26 | NA | 1.70 | 6.40E-05 | 9.65E-04 |
| Slc7a8 | SLC7A8 | 1.70 | 1.32E-09 | 1.34E-07 |
| Il1f6 | IL36A | 1.70 | 3.01E-05 | 5.18E-04 |
| Rnf152 | RNF152 | 1.71 | 4.70E-07 | 1.83E-05 |
| Cnfn | CNFN | 1.71 | 1.20E-12 | 3.62E-10 |
| Klk12 | KLK12 | 1.71 | 8.26E-08 | 4.37E-06 |
| Gp49a | NA | 1.72 | 7.22E-07 | 2.54E-05 |
| Csf3r | CSF3R | 1.73 | 2.36E-04 | 2.74E-03 |
| Klk14 | KLK14 | 1.73 | 1.05E-08 | 8.27E-07 |
| Edn1 | EDN1 | 1.73 | 7.25E-14 | 3.61E-11 |
| Il1f9 | IL36G | 1.74 | 1.59E-09 | 1.52E-07 |
| Endou | ENDOU | 1.74 | 4.50E-10 | 5.47E-08 |
| Nkain1 | NKAIN1 | 1.74 | 6.58E-04 | 6.21E-03 |
| Srgn | SRGN | 1.74 | 1.51E-05 | 2.94E-04 |
| Nlrp3 | NLRP3 | 1.75 | 2.88E-04 | 3.20E-03 |
| 2010109I03Rik | NA | 1.75 | 1.04E-10 | 1.58E-08 |
| Lilrb4 | NA | 1.76 | 9.27E-09 | 7.38E-07 |
| Lce1j | NA | 1.77 | 2.41E-07 | 1.06E-05 |
| Grid2ip | GRID2IP | 1.77 | 9.93E-04 | 8.63E-03 |
| Trim15 | TRIM15 | 1.77 | 4.17E-13 | 1.62E-10 |
| Klk9 | KLK9 | 1.78 | 3.78E-09 | 3.39E-07 |
| Cd300lf | CD300LF | 1.78 | 6.56E-06 | 1.49E-04 |
| Sprr1a | SPRR1A | 1.78 | 2.74E-11 | 5.22E-09 |
| Ska1 | SKA1 | 1.80 | 7.96E-03 | 4.28E-02 |
| Mefv | MEFV | 1.80 | 1.16E-04 | 1.57E-03 |
| Tcf23 | TCF23 | 1.81 | 6.39E-04 | 6.08E-03 |
| Msr1 | MSR1 | 1.82 | 7.15E-10 | 7.96E-08 |
| Il33 | IL33 | 1.82 | 6.02E-18 | 8.44E-15 |
| Slc26a4 | SLC26A4 | 1.83 | 1.75E-07 | 8.10E-06 |
| Fcgr1 | FCGR1A | 1.83 | 1.47E-04 | 1.89E-03 |
| Gcnt3 | GCNT3 | 1.84 | 2.56E-09 | 2.36E-07 |
| 3300005D01Rik | NA | 1.84 | 1.55E-03 | 1.21E-02 |
| Dok3 | DOK3 | 1.85 | 3.40E-04 | 3.68E-03 |
| Fcgr2b | FCGR2B | 1.85 | 1.36E-16 | 1.40E-13 |
| Csf3 | CSF3 | 1.86 | 1.23E-03 | 1.02E-02 |
| Tpsab1 | TPSAB1 | 1.86 | 1.89E-03 | 1.41E-02 |
| Ccl4 | CCL4 | 1.87 | 2.03E-05 | 3.77E-04 |
| Slc11a1 | SLC11A1 | 1.87 | 3.74E-07 | 1.52E-05 |
| Gm10639 | NA | 1.87 | 7.96E-03 | 4.28E-02 |
| Lypd5 | LYPD5 | 1.88 | 1.87E-08 | 1.30E-06 |
| Chit1 | CHIT1 | 1.88 | 7.14E-13 | 2.39E-10 |
| Rhof | RHOF | 1.89 | 1.63E-07 | 7.75E-06 |
| Lce1g | NA | 1.89 | 4.52E-06 | 1.11E-04 |
| 1700001C19Rik | NA | 1.91 | 1.17E-03 | 9.81E-03 |
| Tnfsf9 | TNFSF9 | 1.91 | 1.72E-05 | 3.26E-04 |
| Lce1c | NA | 1.91 | 1.08E-08 | 8.41E-07 |
| Ccr7 | CCR7 | 1.93 | 6.82E-04 | 6.38E-03 |
| Otop2 | OTOP2 | 1.94 | 1.53E-07 | 7.36E-06 |
| Hk3 | HK3 | 1.94 | 1.25E-06 | 4.06E-05 |
| Cyp4f18 | NA | 1.95 | 2.45E-04 | 2.82E-03 |
| Klk13 | KLK13 | 1.95 | 8.06E-11 | 1.31E-08 |
| Atp8b4 | ATP8B4 | 1.95 | 6.60E-05 | 9.90E-04 |
| Ereg | EREG | 1.95 | 5.74E-18 | 8.44E-15 |
| S100a8 | S100A8 | 1.96 | 2.14E-06 | 6.12E-05 |
| Lrg1 | LRG1 | 1.96 | 5.63E-07 | 2.09E-05 |
| Timp1 | TIMP1 | 1.97 | 2.17E-08 | 1.47E-06 |
| Lce1l | NA | 1.97 | 9.32E-07 | 3.15E-05 |
| Sell | SELL | 1.97 | 1.79E-03 | 1.35E-02 |
| Gsdmc | GSDMC | 1.98 | 1.83E-07 | 8.38E-06 |
| Lce1m | NA | 1.98 | 1.59E-03 | 1.24E-02 |
| Slc4a11 | SLC4A11 | 1.98 | 4.46E-10 | 5.47E-08 |
| Slfn1 | SLFN12 | 1.99 | 1.97E-04 | 2.37E-03 |
| 2310050C09Rik | C1orf68 | 1.99 | 1.30E-03 | 1.06E-02 |
| Etv4 | ETV4 | 1.99 | 3.96E-06 | 9.93E-05 |
| Gm3776 | NA | 1.99 | 7.10E-06 | 1.58E-04 |
| Cxcl3 | CXCL1 | 2.02 | 1.41E-09 | 1.41E-07 |
| Cxcl2 | CXCL2 | 2.02 | 5.02E-06 | 1.19E-04 |
| Lce1i | NA | 2.03 | 1.44E-06 | 4.46E-05 |
| B4galnt3 | B4GALNT3 | 2.04 | 3.56E-05 | 5.87E-04 |
| Serpinb9b | SERPINB9 | 2.04 | 2.68E-08 | 1.76E-06 |
| Clec4d | CLEC4D | 2.05 | 4.08E-07 | 1.62E-05 |
| Stfa3 | CSTA | 2.05 | 1.23E-04 | 1.64E-03 |
| Ifitm6 | NA | 2.05 | 1.49E-03 | 1.17E-02 |
| Selp | SELP | 2.07 | 2.15E-03 | 1.57E-02 |
| Bpifa5 | BPIFA1 | 2.07 | 2.62E-05 | 4.61E-04 |
| C5ar1 | C5AR1 | 2.07 | 1.23E-05 | 2.46E-04 |
| Sprr2f | NA | 2.08 | 2.55E-04 | 2.90E-03 |
| Ccl3 | CCL3L3 | 2.09 | 1.60E-06 | 4.85E-05 |
| Cxcl5 | CXCL5 | 2.09 | 1.36E-07 | 6.69E-06 |
| Sprr2j-ps | NA | 2.11 | 5.89E-03 | 3.41E-02 |
| Mmp12 | MMP12 | 2.12 | 3.40E-06 | 8.75E-05 |
| Vwa2 | VWA2 | 2.12 | 2.44E-05 | 4.36E-04 |
| Trim30b | NA | 2.12 | 6.11E-03 | 3.50E-02 |
| U90926 | NA | 2.14 | 9.27E-04 | 8.15E-03 |
| Mgam | MGAM | 2.14 | 4.56E-04 | 4.65E-03 |
| Cemip | CEMIP | 2.14 | 8.92E-06 | 1.91E-04 |
| Sprr2e | NA | 2.16 | 2.69E-03 | 1.87E-02 |
| Tmem213 | TMEM213 | 2.17 | 6.51E-04 | 6.17E-03 |
| Krt6b | NA | 2.19 | 1.92E-08 | 1.33E-06 |
| Sprr1b | SPRR1B | 2.19 | 5.38E-04 | 5.27E-03 |
| Irg1 | NA | 2.24 | 1.73E-05 | 3.28E-04 |
| Tarm1 | TARM1 | 2.26 | 2.44E-04 | 2.81E-03 |
| Chil1 | CHI3L1 | 2.26 | 1.16E-09 | 1.20E-07 |
| Lce1f | NA | 2.27 | 5.59E-07 | 2.09E-05 |
| Wfdc17 | NA | 2.28 | 4.11E-10 | 5.16E-08 |
| S100a9 | S100A9 | 2.30 | 7.34E-07 | 2.57E-05 |
| F10 | F10 | 2.30 | 5.94E-09 | 4.93E-07 |
| Slc2a6 | SLC2A6 | 2.31 | 7.39E-07 | 2.59E-05 |
| Phgr1 | NA | 2.31 | 1.25E-03 | 1.03E-02 |
| Clec4e | CLEC4E | 2.32 | 2.37E-10 | 3.24E-08 |
| Krt16 | KRT16 | 2.32 | 4.99E-09 | 4.32E-07 |
| 4732456N10Rik | NA | 2.35 | 1.52E-06 | 4.65E-05 |
| A530064D06Rik | NA | 2.38 | 4.46E-04 | 4.57E-03 |
| Areg | AREG | 2.39 | 1.28E-18 | 2.83E-15 |
| Gpr84 | GPR84 | 2.40 | 3.27E-07 | 1.36E-05 |
| Krt84 | KRT84 | 2.43 | 3.38E-03 | 2.24E-02 |
| Krt20 | KRT20 | 2.44 | 1.56E-15 | 1.15E-12 |
| Il23a | IL23A | 2.46 | 7.79E-06 | 1.71E-04 |
| Mmp8 | MMP8 | 2.48 | 4.42E-04 | 4.55E-03 |
| Gm6377 | NA | 2.49 | 3.68E-06 | 9.35E-05 |
| Tmem252 | TMEM252 | 2.54 | 1.22E-04 | 1.63E-03 |
| Sprr2k | NA | 2.54 | 8.27E-06 | 1.79E-04 |
| Serpinb3c | SERPINB4 | 2.55 | 1.84E-08 | 1.29E-06 |
| Psors1c2 | PSORS1C2 | 2.57 | 1.37E-12 | 4.06E-10 |
| Ppbp | PPBP | 2.60 | 5.50E-07 | 2.06E-05 |
| Sprr2g | NA | 2.61 | 9.93E-07 | 3.33E-05 |
| Tubb3 | TUBB3 | 2.63 | 5.71E-06 | 1.33E-04 |
| Lipn | LIPN | 2.64 | 3.55E-04 | 3.81E-03 |
| Epgn | EPGN | 2.67 | 2.88E-06 | 7.77E-05 |
| Dnmt3aos | NA | 2.70 | 7.81E-03 | 4.22E-02 |
| Ccl7 | CCL7 | 2.72 | 6.96E-04 | 6.49E-03 |
| Gdf15 | GDF15 | 2.73 | 9.04E-04 | 7.98E-03 |
| Sprr2h | NA | 2.74 | 1.33E-07 | 6.59E-06 |
| Syn1 | SYN1 | 2.74 | 7.13E-04 | 6.63E-03 |
| Apoc2 | APOC4-APOC2 | 2.76 | 3.97E-07 | 1.59E-05 |
| Slc6a12 | SLC6A12 | 2.77 | 8.56E-03 | 4.54E-02 |
| Ctla4 | CTLA4 | 2.81 | 1.08E-05 | 2.23E-04 |
| Rnase2b | RNASE3 | 2.82 | 5.78E-05 | 8.88E-04 |
| Mmp7 | MMP7 | 2.83 | 6.34E-03 | 3.61E-02 |
| Ccl2 | NA | 2.83 | 4.47E-10 | 5.47E-08 |
| Tgm7 | TGM7 | 2.85 | 2.21E-03 | 1.60E-02 |
| Tm4sf19 | TM4SF19-TCTEX1D2 | 2.88 | 4.65E-06 | 1.13E-04 |
| Gm4832 | NA | 2.90 | 2.88E-03 | 1.97E-02 |
| BC100530 | CSTA | 2.92 | 3.49E-05 | 5.80E-04 |
| Fosb | FOSB | 3.00 | 1.95E-11 | 4.11E-09 |
| Retnlg | RETNLB | 3.07 | 1.23E-06 | 3.98E-05 |
| Tdh | NA | 3.08 | 9.44E-03 | 4.89E-02 |
| Grm4 | GRM4 | 3.09 | 6.08E-05 | 9.25E-04 |
| Cnga3 | CNGA3 | 3.13 | 1.68E-05 | 3.22E-04 |
| Htr2b | HTR2B | 3.18 | 7.98E-03 | 4.29E-02 |
| Il24 | IL24 | 3.24 | 8.85E-04 | 7.85E-03 |
| Col6a5 | COL6A5 | 3.27 | 1.72E-08 | 1.23E-06 |
| Klk6 | KLK6 | 3.33 | 2.01E-05 | 3.73E-04 |
| Fosl1 | FOSL1 | 3.35 | 3.81E-17 | 4.90E-14 |
| Krt42 | NA | 3.36 | 3.34E-03 | 2.22E-02 |
| Slit1 | SLIT1 | 3.46 | 1.70E-09 | 1.62E-07 |
| Sprr2d | NA | 3.49 | 2.07E-10 | 2.85E-08 |
| Gm5483 | CSTA | 3.53 | 1.76E-08 | 1.25E-06 |
| Glycam1 | NA | 3.55 | 1.29E-03 | 1.06E-02 |
| Gm5416 | CSTA | 3.64 | 1.89E-04 | 2.29E-03 |
| Prok2 | PROK2 | 3.68 | 1.90E-05 | 3.55E-04 |
| Dpcr1 | DPCR1 | 3.70 | 8.39E-07 | 2.90E-05 |
| Stfa2l1 | CSTA | 3.72 | 5.66E-08 | 3.15E-06 |
| Sprr2b | NA | 3.73 | 1.38E-06 | 4.31E-05 |
| Galnt9 | GALNT9 | 3.77 | 4.97E-03 | 3.01E-02 |
| Defb3 | NA | 3.83 | 9.10E-06 | 1.94E-04 |
| Serpinb9f | SERPINB9 | 3.91 | 8.08E-04 | 7.37E-03 |
| Spink12 | SPINK9 | 3.94 | 1.14E-06 | 3.75E-05 |
| Ngp | NA | 4.01 | 6.98E-03 | 3.86E-02 |
| Sprr2i | NA | 4.38 | 1.49E-08 | 1.09E-06 |
| Sele | SELE | 4.67 | 3.80E-04 | 4.04E-03 |
| Npy | NPY | 4.69 | 1.27E-04 | 1.67E-03 |
| Sh2d5 | SH2D5 | 5.00 | 1.77E-06 | 5.29E-05 |
| Robo3 | ROBO3 | 5.47 | 3.06E-03 | 2.08E-02 |
| Gm15056 | NA | 5.49 | 5.68E-04 | 5.52E-03 |
| Stfa2 | CSTA | 5.76 | 7.51E-07 | 2.62E-05 |
| Saa3 | NA | 5.80 | 1.47E-40 | 2.26E-36 |
| Csn1s1 | NA | 6.54 | 4.29E-09 | 3.79E-07 |
