# Supplementary material for: Bronchial airway gene expression signatures in mouse lung squamous cell carcinoma and their modulation by cancer chemopreventive agents
Source: Oncotarget. 2016 Dec 7;8(12):18885–900. doi: 10.18632/oncotarget.13806 (PMC5386655; doi:10.18632/oncotarget.13806)
Supplement: Supplementary file 4 [file oncotarget-08-18885-s004.docx]

Table S4. Detailed information of the 2,335 miRNA-mRNA pairs that were significantly negative correlated in expression. The paired members were predicted to target each other by at least one miRNA-mRNA database (microCosm/TargetScan or both).

| mmuMiR | mmuGene | cor | pval | logratio.miRNA | logratio.mRNA |
| --- | --- | --- | --- | --- | --- |
| mmu-let-7i-5p | Pgrmc1 | -0.99 | 1.14E-08 | 1.79 | -2.17 |
| mmu-miR-433-3p | Notch1 | -0.98 | 2.08E-08 | -4.72 | 1.81 |
| mmu-miR-203-3p | Map3k1 | -0.98 | 3.09E-08 | 4.73 | -1.61 |
| mmu-miR-20a-5p | Pam | -0.98 | 3.22E-08 | 3.70 | -1.83 |
| mmu-let-7i-5p | Calm2 | -0.98 | 4.13E-08 | 1.79 | -1.70 |
| mmu-let-7i-5p | Reg3g | -0.98 | 6.77E-08 | 1.79 | -2.74 |
| mmu-miR-328-3p | Sbno2 | -0.98 | 9.23E-08 | -5.59 | 2.00 |
| mmu-let-7f-5p | Reg3g | -0.98 | 1.16E-07 | 3.20 | -2.74 |
| mmu-let-7g-5p | Reg3g | -0.98 | 1.57E-07 | 2.05 | -2.74 |
| mmu-miR-221-3p | Cyp4a12b | -0.98 | 1.66E-07 | 2.18 | -2.32 |
| mmu-miR-221-3p | Cyp4a12b | -0.98 | 1.66E-07 | 2.18 | -2.32 |
| mmu-miR-23b-3p | Map3k1 | -0.97 | 1.92E-07 | 2.62 | -1.61 |
| mmu-miR-149-3p | Zfp335 | -0.97 | 3.27E-07 | -4.06 | 1.77 |
| mmu-miR-760-3p | Hist1h1c | -0.97 | 3.46E-07 | -7.15 | 1.76 |
| mmu-miR-504-5p | Rassf1 | -0.97 | 3.51E-07 | -4.12 | 1.62 |
| mmu-miR-181a-5p | Phlda1 | -0.97 | 4.49E-07 | -3.01 | 2.46 |
| mmu-miR-760-3p | Sbno2 | -0.97 | 4.55E-07 | -7.15 | 2.00 |
| mmu-let-7d-3p | Bcat1 | -0.97 | 4.79E-07 | -6.28 | 5.31 |
| mmu-miR-328-3p | Rgl2 | -0.97 | 5.67E-07 | -5.59 | 1.80 |
| mmu-miR-20a-5p | 9230110C19Rik | -0.97 | 5.96E-07 | 3.70 | -2.63 |
| mmu-miR-200b-3p | Map3k1 | -0.97 | 7.27E-07 | 2.19 | -1.61 |
| mmu-miR-877-3p | Scrib | -0.96 | 8.03E-07 | -4.18 | 1.61 |
| mmu-miR-31-5p | Aldoc | -0.96 | 8.79E-07 | 4.65 | -2.23 |
| mmu-miR-221-3p | Fcgrt | -0.96 | 1.03E-06 | 2.18 | -1.94 |
| mmu-miR-23b-3p | Slc15a2 | -0.96 | 1.34E-06 | 2.62 | -3.27 |
| mmu-miR-200b-3p | Tspan13 | -0.96 | 1.52E-06 | 2.19 | -2.22 |
| mmu-miR-181c-5p | Calm2 | -0.96 | 1.57E-06 | 3.05 | -1.70 |
| mmu-miR-200b-3p | Slc15a2 | -0.96 | 1.57E-06 | 2.19 | -3.27 |
| mmu-miR-760-3p | Krt6a | -0.96 | 1.59E-06 | -7.15 | 3.57 |
| mmu-miR-328-3p | Junb | -0.96 | 1.66E-06 | -5.59 | 2.02 |
| mmu-miR-25-3p | Prkar2b | -0.96 | 1.82E-06 | 2.56 | -2.31 |
| mmu-miR-23b-3p | Slc38a1 | -0.96 | 1.88E-06 | 2.62 | -3.08 |
| mmu-miR-744-5p | Krt14 | -0.96 | 1.93E-06 | -3.34 | 3.74 |
| mmu-let-7d-3p | Mst1r | -0.96 | 2.08E-06 | -6.28 | 2.00 |
| mmu-miR-106b-5p | Pam | -0.96 | 2.14E-06 | 4.02 | -1.83 |
| mmu-miR-93-5p | Arl4a | -0.96 | 2.23E-06 | 2.30 | -1.95 |
| mmu-miR-92a-3p | Notch1 | -0.96 | 2.32E-06 | -2.41 | 1.81 |
| mmu-let-7b-5p | Cdkn1a | -0.96 | 2.38E-06 | -3.08 | 1.79 |
| mmu-let-7i-5p | Map3k1 | -0.95 | 2.42E-06 | 1.79 | -1.61 |
| mmu-miR-146a-5p | Slc38a1 | -0.95 | 2.64E-06 | 4.19 | -3.08 |
| mmu-miR-744-5p | Sbno2 | -0.95 | 2.66E-06 | -3.34 | 2.00 |
| mmu-miR-27b-3p | Cntnap2 | -0.95 | 2.74E-06 | 2.45 | -2.56 |
| mmu-miR-98-5p | Reg3g | -0.95 | 2.75E-06 | 3.72 | -2.74 |
| mmu-let-7d-3p | Tbc1d2 | -0.95 | 3.07E-06 | -6.28 | 2.59 |
| mmu-miR-92b-5p | Notch1 | -0.95 | 3.17E-06 | -6.76 | 1.81 |
| mmu-miR-20a-5p | Pfn2 | -0.95 | 3.20E-06 | 3.70 | -1.75 |
| mmu-miR-93-5p | 9230110C19Rik | -0.95 | 3.56E-06 | 2.30 | -2.63 |
| mmu-miR-20a-5p | Arl4a | -0.95 | 3.64E-06 | 3.70 | -1.95 |
| mmu-miR-23a-3p | Map3k1 | -0.95 | 3.67E-06 | 3.01 | -1.61 |
| mmu-miR-20a-5p | Enpp5 | -0.95 | 3.68E-06 | 3.70 | -1.89 |
| mmu-miR-20a-5p | Slc25a23 | -0.95 | 3.79E-06 | 3.70 | -2.24 |
| mmu-miR-20a-5p | Map3k1 | -0.95 | 3.82E-06 | 3.70 | -1.61 |
| mmu-miR-181c-5p | Pam | -0.95 | 3.93E-06 | 3.05 | -1.83 |
| mmu-miR-760-3p | Ecm1 | -0.95 | 4.02E-06 | -7.15 | 2.00 |
| mmu-miR-221-3p | Scgb3a2 | -0.95 | 4.07E-06 | 2.18 | -3.30 |
| mmu-miR-26a-5p | Map3k1 | -0.95 | 4.07E-06 | 2.26 | -1.61 |
| mmu-miR-31-5p | Map3k1 | -0.95 | 4.12E-06 | 4.65 | -1.61 |
| mmu-miR-141-3p | Pgrmc1 | -0.95 | 4.16E-06 | 4.66 | -2.17 |
| mmu-miR-25-3p | Abi3bp | -0.95 | 4.36E-06 | 2.56 | -3.56 |
| mmu-miR-433-3p | Ccnl2 | -0.95 | 4.50E-06 | -4.72 | 2.72 |
| mmu-miR-504-5p | Rbp1 | -0.95 | 4.53E-06 | -4.12 | 2.21 |
| mmu-miR-429-3p | Pam | -0.95 | 4.58E-06 | 3.03 | -1.83 |
| mmu-let-7d-3p | Bax | -0.95 | 4.60E-06 | -6.28 | 1.86 |
| mmu-let-7d-3p | Mxd1 | -0.95 | 4.63E-06 | -6.28 | 1.98 |
| mmu-miR-30a-5p | Map3k1 | -0.95 | 4.77E-06 | 2.87 | -1.61 |
| mmu-miR-3960 | Junb | -0.95 | 4.93E-06 | -6.13 | 2.02 |
| mmu-miR-26b-5p | Il13ra1 | -0.95 | 4.95E-06 | 3.80 | -1.76 |
| mmu-miR-23b-3p | Sms | -0.95 | 5.22E-06 | 2.62 | -2.09 |
| mmu-let-7d-3p | Mesdc1 | -0.95 | 5.34E-06 | -6.28 | 1.83 |
| mmu-let-7f-5p | Aldoc | -0.95 | 5.53E-06 | 3.20 | -2.23 |
| mmu-miR-221-3p | Msh2 | -0.95 | 5.72E-06 | 2.18 | -1.86 |
| mmu-miR-328-3p | Tnip2 | -0.94 | 5.82E-06 | -5.59 | 1.67 |
| mmu-miR-200b-3p | Sdc2 | -0.94 | 5.84E-06 | 2.19 | -2.29 |
| mmu-miR-20a-5p | Fam13a | -0.94 | 5.96E-06 | 3.70 | -3.21 |
| mmu-let-7d-3p | Ccnd2 | -0.94 | 6.03E-06 | -6.28 | 2.09 |
| mmu-miR-24-3p | Slc27a2 | -0.94 | 6.14E-06 | 1.73 | -3.23 |
| mmu-miR-203-3p | Acsl3 | -0.94 | 6.35E-06 | 4.73 | -1.65 |
| mmu-miR-182-5p | Pam | -0.94 | 6.37E-06 | 3.32 | -1.83 |
| mmu-miR-433-3p | Nlrc5 | -0.94 | 6.55E-06 | -4.72 | 4.26 |
| mmu-miR-433-3p | Tmsb10 | -0.94 | 6.71E-06 | -4.72 | 2.16 |
| mmu-miR-92a-3p | Myo5a | -0.94 | 6.82E-06 | -2.41 | 1.84 |
| mmu-miR-328-3p | Ttyh2 | -0.94 | 6.82E-06 | -5.59 | 1.76 |
| mmu-let-7b-5p | Phlda3 | -0.94 | 6.85E-06 | -3.08 | 2.39 |
| mmu-miR-200b-3p | Sar1b | -0.94 | 7.23E-06 | 2.19 | -1.65 |
| mmu-miR-23b-3p | Arhgap5 | -0.94 | 7.34E-06 | 2.62 | -1.85 |
| mmu-miR-141-3p | Slc15a2 | -0.94 | 7.41E-06 | 4.66 | -3.27 |
| mmu-miR-22-3p | Prdx1 | -0.94 | 7.46E-06 | 2.95 | -1.59 |
| mmu-let-7i-5p | Dnajb13 | -0.94 | 7.61E-06 | 1.79 | -1.82 |
| mmu-miR-26b-5p | Map3k1 | -0.94 | 7.71E-06 | 3.80 | -1.61 |
| mmu-let-7i-5p | Cdh26 | -0.94 | 7.71E-06 | 1.79 | -2.39 |
| mmu-miR-375-3p | Phlda1 | -0.94 | 8.17E-06 | -2.94 | 2.46 |
| mmu-let-7i-5p | Chpt1 | -0.94 | 8.44E-06 | 1.79 | -2.38 |
| mmu-miR-328-3p | Nbeal2 | -0.94 | 8.72E-06 | -5.59 | 2.56 |
| mmu-miR-744-5p | Junb | -0.94 | 8.76E-06 | -3.34 | 2.02 |
| mmu-miR-26a-5p | Sar1b | -0.94 | 8.79E-06 | 2.26 | -1.65 |
| mmu-let-7f-5p | Pgrmc1 | -0.94 | 8.84E-06 | 3.20 | -2.17 |
| mmu-miR-328-3p | Mesdc1 | -0.94 | 8.96E-06 | -5.59 | 1.83 |
| mmu-miR-760-3p | Iffo2 | -0.94 | 9.28E-06 | -7.15 | 1.74 |
| mmu-miR-23b-3p | Cyp4a12a | -0.94 | 9.31E-06 | 2.62 | -3.14 |
| mmu-miR-181c-5p | Itln1 | -0.94 | 9.39E-06 | 3.05 | -2.75 |
| mmu-miR-17-5p | 9230110C19Rik | -0.94 | 9.57E-06 | 3.83 | -2.63 |
| mmu-miR-429-3p | Map3k1 | -0.94 | 9.70E-06 | 3.03 | -1.61 |
| mmu-miR-25-3p | Casd1 | -0.94 | 9.79E-06 | 2.56 | -2.06 |
| mmu-miR-22-3p | Ogn | -0.94 | 1.02E-05 | 2.95 | -3.66 |
| mmu-miR-760-3p | Ankrd11 | -0.94 | 1.03E-05 | -7.15 | 1.76 |
| mmu-miR-181c-5p | Rcbtb2 | -0.94 | 1.04E-05 | 3.05 | -1.59 |
| mmu-miR-221-3p | Cdkn1c | -0.94 | 1.04E-05 | 2.18 | -2.19 |
| mmu-miR-744-5p | Syt8 | -0.94 | 1.04E-05 | -3.34 | 2.09 |
| mmu-miR-200b-3p | Pam | -0.94 | 1.04E-05 | 2.19 | -1.83 |
| mmu-let-7g-5p | Pgrmc1 | -0.94 | 1.06E-05 | 2.05 | -2.17 |
| mmu-miR-203-3p | Rhoq | -0.94 | 1.07E-05 | 4.73 | -1.59 |
| mmu-miR-22-3p | Cyp4a12b | -0.94 | 1.08E-05 | 2.95 | -2.32 |
| mmu-miR-22-3p | Cyp4a12b | -0.94 | 1.08E-05 | 2.95 | -2.32 |
| mmu-miR-106b-5p | Map3k1 | -0.94 | 1.08E-05 | 4.02 | -1.61 |
| mmu-miR-17-5p | Pam | -0.94 | 1.10E-05 | 3.83 | -1.83 |
| mmu-miR-328-3p | Ecm1 | -0.94 | 1.10E-05 | -5.59 | 2.00 |
| mmu-miR-98-5p | Calm2 | -0.94 | 1.12E-05 | 3.72 | -1.70 |
| mmu-miR-20a-5p | Ero1lb | -0.94 | 1.12E-05 | 3.70 | -1.73 |
| mmu-miR-149-3p | Gm8909 | -0.94 | 1.16E-05 | -4.06 | 3.30 |
| mmu-miR-149-3p | Ankrd11 | -0.94 | 1.19E-05 | -4.06 | 1.76 |
| mmu-miR-421-3p | Acsl3 | -0.94 | 1.20E-05 | 3.10 | -1.65 |
| mmu-miR-30b-5p | Map3k1 | -0.94 | 1.21E-05 | 2.59 | -1.61 |
| mmu-miR-17-5p | Rora | -0.93 | 1.22E-05 | 3.83 | -2.00 |
| mmu-miR-200b-3p | Prkar2b | -0.93 | 1.23E-05 | 2.19 | -2.31 |
| mmu-miR-93-5p | Rora | -0.93 | 1.24E-05 | 2.30 | -2.00 |
| mmu-miR-203-3p | Slc25a23 | -0.93 | 1.26E-05 | 4.73 | -2.24 |
| mmu-miR-181c-5p | Ric3 | -0.93 | 1.26E-05 | 3.05 | -2.30 |
| mmu-miR-17-5p | Elmod1 | -0.93 | 1.28E-05 | 3.83 | -1.66 |
| mmu-miR-149-3p | Mcam | -0.93 | 1.28E-05 | -4.06 | 3.37 |
| mmu-miR-23a-3p | Cyp4a12b | -0.93 | 1.29E-05 | 3.01 | -2.32 |
| mmu-miR-181a-5p | Rassf1 | -0.93 | 1.30E-05 | -3.01 | 1.62 |
| mmu-miR-340-5p | Map3k1 | -0.93 | 1.31E-05 | 9.01 | -1.61 |
| mmu-miR-17-5p | Fam13a | -0.93 | 1.34E-05 | 3.83 | -3.21 |
| mmu-miR-149-3p | Mst1r | -0.93 | 1.37E-05 | -4.06 | 2.00 |
| mmu-miR-203-3p | Igfbp5 | -0.93 | 1.39E-05 | 4.73 | -1.94 |
| mmu-miR-200b-3p | Fstl1 | -0.93 | 1.41E-05 | 2.19 | -2.02 |
| mmu-miR-146a-5p | Cyp2a5 | -0.93 | 1.41E-05 | 4.19 | -2.10 |
| mmu-miR-31-5p | Il13ra1 | -0.93 | 1.42E-05 | 4.65 | -1.76 |
| mmu-miR-744-5p | Pfkfb3 | -0.93 | 1.43E-05 | -3.34 | 1.73 |
| mmu-miR-27a-3p | Cntnap2 | -0.93 | 1.43E-05 | 3.17 | -2.56 |
| mmu-let-7d-3p | Prss22 | -0.93 | 1.48E-05 | -6.28 | 2.11 |
| mmu-miR-328-3p | Gadd45g | -0.93 | 1.53E-05 | -5.59 | 2.78 |
| mmu-miR-27a-3p | Rora | -0.93 | 1.56E-05 | 3.17 | -2.00 |
| mmu-miR-23a-3p | Slc15a2 | -0.93 | 1.58E-05 | 3.01 | -3.27 |
| mmu-let-7d-3p | Fbxl19 | -0.93 | 1.61E-05 | -6.28 | 1.74 |
| mmu-let-7g-5p | Aldoc | -0.93 | 1.63E-05 | 2.05 | -2.23 |
| mmu-miR-744-5p | Prss22 | -0.93 | 1.67E-05 | -3.34 | 2.11 |
| mmu-miR-210-5p | Ccnl2 | -0.93 | 1.69E-05 | -3.25 | 2.72 |
| mmu-miR-93-5p | Sar1b | -0.93 | 1.70E-05 | 2.30 | -1.65 |
| mmu-miR-98-5p | Pgrmc1 | -0.93 | 1.75E-05 | 3.72 | -2.17 |
| mmu-miR-23b-3p | Abi3bp | -0.93 | 1.79E-05 | 2.62 | -3.56 |
| mmu-miR-744-5p | Nbeal2 | -0.93 | 1.79E-05 | -3.34 | 2.56 |
| mmu-miR-20a-5p | Galm | -0.93 | 1.80E-05 | 3.70 | -2.36 |
| mmu-miR-17-5p | Arl4a | -0.93 | 1.80E-05 | 3.83 | -1.95 |
| mmu-miR-25-3p | Sdc2 | -0.93 | 1.83E-05 | 2.56 | -2.29 |
| mmu-miR-149-3p | Mxd1 | -0.93 | 1.83E-05 | -4.06 | 1.98 |
| mmu-let-7b-5p | Mxd1 | -0.93 | 1.89E-05 | -3.08 | 1.98 |
| mmu-miR-155-5p | Msh2 | -0.93 | 1.90E-05 | 1.93 | -1.86 |
| mmu-miR-210-5p | Mx1 | -0.93 | 1.90E-05 | -3.25 | 2.71 |
| mmu-miR-20a-5p | 4931406C07Rik | -0.93 | 1.94E-05 | 3.70 | -2.42 |
| mmu-miR-425-5p | Calm2 | -0.93 | 1.95E-05 | 1.93 | -1.70 |
| mmu-miR-23b-3p | Casd1 | -0.93 | 1.95E-05 | 2.62 | -2.06 |
| mmu-miR-25-3p | Sar1b | -0.93 | 2.04E-05 | 2.56 | -1.65 |
| mmu-miR-17-5p | Enpp5 | -0.93 | 2.06E-05 | 3.83 | -1.89 |
| mmu-miR-182-5p | Emb | -0.93 | 2.09E-05 | 3.32 | -2.73 |
| mmu-miR-181c-5p | Cntnap2 | -0.93 | 2.12E-05 | 3.05 | -2.56 |
| mmu-miR-20a-5p | Rora | -0.93 | 2.13E-05 | 3.70 | -2.00 |
| mmu-miR-23b-3p | Col15a1 | -0.93 | 2.14E-05 | 2.62 | -2.31 |
| mmu-miR-27a-3p | Pxmp2 | -0.93 | 2.17E-05 | 3.17 | -3.43 |
| mmu-miR-23a-3p | Abi3bp | -0.93 | 2.19E-05 | 3.01 | -3.56 |
| mmu-miR-29a-3p | Ppic | -0.93 | 2.22E-05 | 2.71 | -2.39 |
| mmu-miR-340-5p | Pam | -0.93 | 2.26E-05 | 9.01 | -1.83 |
| mmu-miR-149-3p | Myo9b | -0.92 | 2.28E-05 | -4.06 | 1.74 |
| mmu-miR-34a-5p | Fcgrt | -0.92 | 2.31E-05 | 3.60 | -1.94 |
| mmu-let-7f-5p | Map3k1 | -0.92 | 2.31E-05 | 3.20 | -1.61 |
| mmu-let-7i-5p | Cd36 | -0.92 | 2.32E-05 | 1.79 | -1.99 |
| mmu-miR-92b-5p | Myo5a | -0.92 | 2.34E-05 | -6.76 | 1.84 |
| mmu-miR-23b-3p | Ccdc103 | -0.92 | 2.35E-05 | 2.62 | -2.01 |
| mmu-miR-760-3p | Myo5a | -0.92 | 2.44E-05 | -7.15 | 1.84 |
| mmu-miR-210-5p | Muc4 | -0.92 | 2.47E-05 | -3.25 | 1.94 |
| mmu-miR-15a-5p | Pam | -0.92 | 2.55E-05 | 2.29 | -1.83 |
| mmu-miR-203-3p | Fam13a | -0.92 | 2.58E-05 | 4.73 | -3.21 |
| mmu-miR-26a-5p | Acsl3 | -0.92 | 2.60E-05 | 2.26 | -1.65 |
| mmu-miR-20a-5p | Stk33 | -0.92 | 2.60E-05 | 3.70 | -2.78 |
| mmu-let-7f-5p | Calm2 | -0.92 | 2.64E-05 | 3.20 | -1.70 |
| mmu-miR-143-3p | Car8 | -0.92 | 2.70E-05 | 2.79 | -2.18 |
| mmu-miR-26b-5p | Sar1b | -0.92 | 2.70E-05 | 3.80 | -1.65 |
| mmu-miR-200a-3p | Pgrmc1 | -0.92 | 2.71E-05 | 2.61 | -2.17 |
| mmu-miR-30a-5p | Abi3bp | -0.92 | 2.71E-05 | 2.87 | -3.56 |
| mmu-let-7b-5p | Plaur | -0.92 | 2.76E-05 | -3.08 | 2.60 |
| mmu-miR-17-5p | 1110032A03Rik | -0.92 | 2.86E-05 | 3.83 | -1.79 |
| mmu-miR-29a-3p | Sms | -0.92 | 2.87E-05 | 2.71 | -2.09 |
| mmu-miR-25-3p | Cdkn1c | -0.92 | 2.87E-05 | 2.56 | -2.19 |
| mmu-miR-185-3p | Ccnl2 | -0.92 | 2.88E-05 | -3.01 | 2.72 |
| mmu-miR-26a-5p | Il13ra1 | -0.92 | 2.91E-05 | 2.26 | -1.76 |
| mmu-miR-221-3p | Dcun1d1 | -0.92 | 2.93E-05 | 2.18 | -1.64 |
| mmu-miR-141-3p | Wnt5a | -0.92 | 2.94E-05 | 4.66 | -2.52 |
| mmu-miR-340-5p | Prkar2b | -0.92 | 2.97E-05 | 9.01 | -2.31 |
| mmu-miR-34a-5p | Ramp2 | -0.92 | 3.03E-05 | 3.60 | -2.72 |
| mmu-miR-340-5p | Calm2 | -0.92 | 3.07E-05 | 9.01 | -1.70 |
| mmu-miR-26a-5p | Prkaa2 | -0.92 | 3.11E-05 | 2.26 | -2.21 |
| mmu-miR-17-5p | 4931406C07Rik | -0.92 | 3.15E-05 | 3.83 | -2.42 |
| mmu-miR-504-5p | Bax | -0.92 | 3.16E-05 | -4.12 | 1.86 |
| mmu-miR-23a-3p | Arhgap5 | -0.92 | 3.20E-05 | 3.01 | -1.85 |
| mmu-miR-26a-5p | Gmnn | -0.92 | 3.22E-05 | 2.26 | -2.26 |
| mmu-miR-340-5p | Reg3g | -0.92 | 3.22E-05 | 9.01 | -2.74 |
| mmu-miR-17-5p | Map3k1 | -0.92 | 3.25E-05 | 3.83 | -1.61 |
| mmu-miR-92b-5p | Slc4a11 | -0.92 | 3.26E-05 | -6.76 | 3.42 |
| mmu-miR-92b-5p | Arntl2 | -0.92 | 3.26E-05 | -6.76 | 3.18 |
| mmu-miR-877-3p | Slc4a11 | -0.92 | 3.28E-05 | -4.18 | 3.42 |
| mmu-miR-141-3p | Sdc2 | -0.92 | 3.37E-05 | 4.66 | -2.29 |
| mmu-miR-429-3p | Msh2 | -0.92 | 3.39E-05 | 3.03 | -1.86 |
| mmu-miR-24-3p | Tbc1d30 | -0.92 | 3.41E-05 | 1.73 | -2.52 |
| mmu-miR-26a-5p | Slc38a1 | -0.92 | 3.44E-05 | 2.26 | -3.08 |
| mmu-miR-22-3p | Pxmp2 | -0.92 | 3.48E-05 | 2.95 | -3.43 |
| mmu-miR-29a-3p | Sparc | -0.92 | 3.58E-05 | 2.71 | -1.95 |
| mmu-miR-429-3p | Prkar2b | -0.92 | 3.58E-05 | 3.03 | -2.31 |
| mmu-let-7b-5p | Fbxl19 | -0.92 | 3.65E-05 | -3.08 | 1.74 |
| mmu-miR-340-5p | Sdc2 | -0.92 | 3.69E-05 | 9.01 | -2.29 |
| mmu-let-7g-5p | Calm2 | -0.92 | 3.74E-05 | 2.05 | -1.70 |
| mmu-miR-146a-5p | Cetn2 | -0.92 | 3.76E-05 | 4.19 | -1.85 |
| mmu-miR-17-5p | Pfn2 | -0.92 | 3.77E-05 | 3.83 | -1.75 |
| mmu-miR-24-3p | Tnfrsf19 | -0.92 | 3.86E-05 | 1.73 | -3.09 |
| mmu-miR-203-3p | Padi2 | -0.92 | 3.87E-05 | 4.73 | -2.11 |
| mmu-miR-22-3p | Il13ra1 | -0.91 | 3.95E-05 | 2.95 | -1.76 |
| mmu-let-7b-5p | Lilrb4 | -0.91 | 4.06E-05 | -3.08 | 3.54 |
| mmu-miR-30a-5p | Msh2 | -0.91 | 4.08E-05 | 2.87 | -1.86 |
| mmu-miR-92a-3p | Osr1 | -0.91 | 4.13E-05 | -2.41 | 1.93 |
| mmu-miR-23b-3p | Aldh1a1 | -0.91 | 4.14E-05 | 2.62 | -1.70 |
| mmu-miR-93-5p | 4931406C07Rik | -0.91 | 4.21E-05 | 2.30 | -2.42 |
| mmu-miR-181c-5p | Abi3bp | -0.91 | 4.22E-05 | 3.05 | -3.56 |
| mmu-miR-27b-3p | Rora | -0.91 | 4.23E-05 | 2.45 | -2.00 |
| mmu-miR-23a-3p | Sms | -0.91 | 4.24E-05 | 3.01 | -2.09 |
| mmu-miR-200b-3p | Casd1 | -0.91 | 4.30E-05 | 2.19 | -2.06 |
| mmu-miR-223-3p | Copz2 | -0.91 | 4.35E-05 | 9.15 | -2.99 |
| mmu-miR-23b-3p | Msrb2 | -0.91 | 4.36E-05 | 2.62 | -2.49 |
| mmu-let-7b-5p | Hbegf | -0.91 | 4.38E-05 | -3.08 | 1.73 |
| mmu-miR-93-5p | Gprasp1 | -0.91 | 4.42E-05 | 2.30 | -2.36 |
| mmu-miR-27b-3p | Pxmp2 | -0.91 | 4.43E-05 | 2.45 | -3.43 |
| mmu-miR-23a-3p | Tspan12 | -0.91 | 4.47E-05 | 3.01 | -1.95 |
| mmu-miR-328-3p | Zfp335 | -0.91 | 4.54E-05 | -5.59 | 1.77 |
| mmu-miR-146a-5p | Mettl7a1 | -0.91 | 4.55E-05 | 4.19 | -1.71 |
| mmu-miR-24-3p | Cyp2a5 | -0.91 | 4.56E-05 | 1.73 | -2.10 |
| mmu-miR-429-3p | Sar1b | -0.91 | 4.62E-05 | 3.03 | -1.65 |
| mmu-miR-98-5p | Rabgap1l | -0.91 | 4.76E-05 | 3.72 | -2.04 |
| mmu-miR-34a-5p | Tnfrsf19 | -0.91 | 4.82E-05 | 3.60 | -3.09 |
| mmu-let-7i-5p | Prkaa2 | -0.91 | 4.88E-05 | 1.79 | -2.21 |
| mmu-miR-24-3p | Fcgrt | -0.91 | 4.95E-05 | 1.73 | -1.94 |
| mmu-miR-429-3p | Sdc2 | -0.91 | 4.98E-05 | 3.03 | -2.29 |
| mmu-miR-27b-3p | Fam13a | -0.91 | 4.98E-05 | 2.45 | -3.21 |
| mmu-miR-34a-5p | Creb3l1 | -0.91 | 5.02E-05 | 3.60 | -2.44 |
| mmu-miR-149-3p | Rgl2 | -0.91 | 5.04E-05 | -4.06 | 1.80 |
| mmu-miR-328-3p | Isg15 | -0.91 | 5.04E-05 | -5.59 | 1.65 |
| mmu-miR-182-5p | Creb3l1 | -0.91 | 5.04E-05 | 3.32 | -2.44 |
| mmu-miR-182-5p | Sar1b | -0.91 | 5.11E-05 | 3.32 | -1.65 |
| mmu-miR-223-3p | Cldn8 | -0.91 | 5.19E-05 | 9.15 | -2.86 |
| mmu-miR-92a-3p | Arntl2 | -0.91 | 5.24E-05 | -2.41 | 3.18 |
| mmu-miR-25-3p | Ppic | -0.91 | 5.24E-05 | 2.56 | -2.39 |
| mmu-miR-181c-5p | Mlf1 | -0.91 | 5.28E-05 | 3.05 | -2.32 |
| mmu-miR-27b-3p | Fgf1 | -0.91 | 5.30E-05 | 2.45 | -1.67 |
| mmu-miR-26b-5p | Acsl3 | -0.91 | 5.36E-05 | 3.80 | -1.65 |
| mmu-miR-375-3p | Cdkn2b | -0.91 | 5.39E-05 | -2.94 | 1.78 |
| mmu-miR-29a-3p | Msh2 | -0.91 | 5.42E-05 | 2.71 | -1.86 |
| mmu-miR-23b-3p | Fgf1 | -0.91 | 5.43E-05 | 2.62 | -1.67 |
| mmu-let-7d-3p | C130026I21Rik | -0.91 | 5.44E-05 | -6.28 | 4.65 |
| mmu-let-7d-3p | Plaur | -0.91 | 5.47E-05 | -6.28 | 2.60 |
| mmu-miR-25-3p | Rora | -0.91 | 5.49E-05 | 2.56 | -2.00 |
| mmu-miR-29a-3p | Arl4a | -0.91 | 5.55E-05 | 2.71 | -1.95 |
| mmu-miR-92a-3p | Slc4a11 | -0.91 | 5.58E-05 | -2.41 | 3.42 |
| mmu-let-7g-5p | Map3k1 | -0.91 | 5.58E-05 | 2.05 | -1.61 |
| mmu-miR-27b-3p | Prkaa2 | -0.91 | 5.75E-05 | 2.45 | -2.21 |
| mmu-miR-92b-5p | Dusp5 | -0.91 | 5.86E-05 | -6.76 | 1.63 |
| mmu-miR-181c-5p | Lclat1 | -0.91 | 5.87E-05 | 3.05 | -1.84 |
| mmu-miR-155-5p | Cldn8 | -0.91 | 5.89E-05 | 1.93 | -2.86 |
| mmu-miR-23b-3p | Foxp2 | -0.91 | 6.01E-05 | 2.62 | -2.72 |
| mmu-miR-200a-3p | Slc15a2 | -0.91 | 6.02E-05 | 2.61 | -3.27 |
| mmu-miR-200b-3p | Rabgap1l | -0.91 | 6.08E-05 | 2.19 | -2.04 |
| mmu-let-7d-3p | Aen | -0.91 | 6.08E-05 | -6.28 | 1.77 |
| mmu-miR-31-5p | Fmo5 | -0.91 | 6.09E-05 | 4.65 | -1.61 |
| mmu-miR-433-3p | H2-Q4 | -0.91 | 6.15E-05 | -4.72 | 1.78 |
| mmu-miR-182-5p | Sdc2 | -0.91 | 6.21E-05 | 3.32 | -2.29 |
| mmu-miR-218-5p | Gmnn | -0.91 | 6.28E-05 | 3.13 | -2.26 |
| mmu-miR-328-3p | Nlrc5 | -0.91 | 6.28E-05 | -5.59 | 4.26 |
| mmu-miR-223-3p | Arhgap5 | -0.91 | 6.29E-05 | 9.15 | -1.85 |
| mmu-miR-26b-5p | Prkaa2 | -0.91 | 6.30E-05 | 3.80 | -2.21 |
| mmu-miR-149-3p | Iffo2 | -0.91 | 6.32E-05 | -4.06 | 1.74 |
| mmu-let-7d-3p | Ccl3 | -0.91 | 6.34E-05 | -6.28 | 3.93 |
| mmu-let-7d-3p | Ccl3 | -0.91 | 6.34E-05 | -6.28 | 3.93 |
| mmu-miR-203-3p | Tekt1 | -0.91 | 6.37E-05 | 4.73 | -2.28 |
| mmu-miR-93-5p | Enpp5 | -0.90 | 6.40E-05 | 2.30 | -1.89 |
| mmu-miR-340-5p | Arhgap5 | -0.90 | 6.45E-05 | 9.01 | -1.85 |
| mmu-miR-146a-5p | Crls1 | -0.90 | 6.46E-05 | 4.19 | -1.62 |
| mmu-miR-98-5p | Map3k1 | -0.90 | 6.48E-05 | 3.72 | -1.61 |
| mmu-miR-223-3p | Tnfrsf19 | -0.90 | 6.55E-05 | 9.15 | -3.09 |
| mmu-let-7d-3p | Muc4 | -0.90 | 6.57E-05 | -6.28 | 1.94 |
| mmu-miR-181c-5p | Cetn2 | -0.90 | 6.68E-05 | 3.05 | -1.85 |
| mmu-miR-200b-3p | Ccdc103 | -0.90 | 6.74E-05 | 2.19 | -2.01 |
| mmu-miR-27a-3p | Prkaa2 | -0.90 | 6.82E-05 | 3.17 | -2.21 |
| mmu-miR-23a-5p | Ccnl2 | -0.90 | 6.84E-05 | -2.43 | 2.72 |
| mmu-miR-29a-3p | Mlf1 | -0.90 | 6.92E-05 | 2.71 | -2.32 |
| mmu-miR-29a-3p | Ttc30a1 | -0.90 | 7.06E-05 | 2.71 | -2.40 |
| mmu-miR-30e-5p | Map3k1 | -0.90 | 7.06E-05 | 3.60 | -1.61 |
| mmu-miR-17-5p | Gprasp1 | -0.90 | 7.07E-05 | 3.83 | -2.36 |
| mmu-miR-181a-5p | Myo9b | -0.90 | 7.08E-05 | -3.01 | 1.74 |
| mmu-miR-433-3p | Krt6a | -0.90 | 7.10E-05 | -4.72 | 3.57 |
| mmu-miR-93-5p | Map3k1 | -0.90 | 7.15E-05 | 2.30 | -1.61 |
| mmu-miR-106b-5p | Slc25a23 | -0.90 | 7.19E-05 | 4.02 | -2.24 |
| mmu-miR-221-3p | Lgi2 | -0.90 | 7.23E-05 | 2.18 | -2.32 |
| mmu-miR-141-3p | Atp6v1c2 | -0.90 | 7.25E-05 | 4.66 | -2.75 |
| mmu-miR-22-3p | Rabgap1l | -0.90 | 7.26E-05 | 2.95 | -2.04 |
| mmu-miR-17-5p | Slc25a23 | -0.90 | 7.30E-05 | 3.83 | -2.24 |
| mmu-miR-143-3p | Elmod1 | -0.90 | 7.30E-05 | 2.79 | -1.66 |
| mmu-miR-760-3p | Itga5 | -0.90 | 7.31E-05 | -7.15 | 2.47 |
| mmu-miR-30b-5p | Msh2 | -0.90 | 7.44E-05 | 2.59 | -1.86 |
| mmu-miR-328-3p | Pim1 | -0.90 | 7.57E-05 | -5.59 | 1.62 |
| mmu-miR-328-3p | Rassf1 | -0.90 | 7.71E-05 | -5.59 | 1.62 |
| mmu-miR-26b-5p | Slc38a1 | -0.90 | 7.81E-05 | 3.80 | -3.08 |
| mmu-miR-22-3p | Clstn3 | -0.90 | 7.86E-05 | 2.95 | -5.05 |
| mmu-miR-30e-5p | Rabgap1l | -0.90 | 7.92E-05 | 3.60 | -2.04 |
| mmu-miR-210-5p | Epha2 | -0.90 | 8.05E-05 | -3.25 | 2.01 |
| mmu-miR-760-3p | Arid5a | -0.90 | 8.09E-05 | -7.15 | 1.86 |
| mmu-miR-200b-3p | Cdh26 | -0.90 | 8.11E-05 | 2.19 | -2.39 |
| mmu-miR-23b-3p | Ero1lb | -0.90 | 8.13E-05 | 2.62 | -1.73 |
| mmu-let-7b-5p | Dsg3 | -0.90 | 8.14E-05 | -3.08 | 1.91 |
| mmu-let-7d-3p | Hbegf | -0.90 | 8.32E-05 | -6.28 | 1.73 |
| mmu-miR-223-3p | Acsl3 | -0.90 | 8.33E-05 | 9.15 | -1.65 |
| mmu-miR-449c-5p | Notch1 | -0.90 | 8.34E-05 | -3.77 | 1.81 |
| mmu-miR-27a-3p | Fam13a | -0.90 | 8.36E-05 | 3.17 | -3.21 |
| mmu-miR-26a-5p | Rora | -0.90 | 8.40E-05 | 2.26 | -2.00 |
| mmu-miR-200a-3p | Msh2 | -0.90 | 8.43E-05 | 2.61 | -1.86 |
| mmu-miR-433-3p | Tpcn2 | -0.90 | 8.43E-05 | -4.72 | 2.16 |
| mmu-miR-27b-3p | Kitl | -0.90 | 8.44E-05 | 2.45 | -2.33 |
| mmu-miR-149-3p | Clcf1 | -0.90 | 8.45E-05 | -4.06 | 2.33 |
| mmu-miR-93-5p | 1110032A03Rik | -0.90 | 8.50E-05 | 2.30 | -1.79 |
| mmu-miR-29a-3p | Spag16 | -0.90 | 8.56E-05 | 2.71 | -3.82 |
| mmu-miR-23a-3p | Slc38a1 | -0.90 | 8.56E-05 | 3.01 | -3.08 |
| mmu-miR-181a-5p | Srgn | -0.90 | 8.58E-05 | -3.01 | 3.70 |
| mmu-let-7d-3p | Cdkn1a | -0.90 | 8.68E-05 | -6.28 | 1.79 |
| mmu-miR-30b-5p | Abi3bp | -0.90 | 8.70E-05 | 2.59 | -3.56 |
| mmu-miR-425-5p | Slitrk6 | -0.90 | 8.70E-05 | 1.93 | -3.34 |
| mmu-miR-181a-5p | Lilrb4 | -0.90 | 8.71E-05 | -3.01 | 3.54 |
| mmu-miR-16-5p | Pam | -0.90 | 8.71E-05 | 1.77 | -1.83 |
| mmu-miR-30b-5p | Rabgap1l | -0.90 | 8.75E-05 | 2.59 | -2.04 |
| mmu-miR-24-3p | Ccdc103 | -0.90 | 8.76E-05 | 1.73 | -2.01 |
| mmu-miR-182-5p | 5330417C22Rik | -0.90 | 8.76E-05 | 3.32 | -2.44 |
| mmu-miR-23a-3p | Ero1lb | -0.90 | 8.82E-05 | 3.01 | -1.73 |
| mmu-miR-203-3p | Clstn3 | -0.90 | 8.84E-05 | 4.73 | -5.05 |
| mmu-miR-34a-5p | Cdkn1c | -0.90 | 8.99E-05 | 3.60 | -2.19 |
| mmu-miR-433-3p | Eda2r | -0.90 | 9.15E-05 | -4.72 | 2.26 |
| mmu-miR-34a-5p | Scgb3a1 | -0.90 | 9.15E-05 | 3.60 | -3.85 |
| mmu-miR-31-5p | Chpt1 | -0.90 | 9.17E-05 | 4.65 | -2.38 |
| mmu-miR-141-3p | Msh2 | -0.90 | 9.28E-05 | 4.66 | -1.86 |
| mmu-miR-26b-5p | Rhoq | -0.90 | 9.35E-05 | 3.80 | -1.59 |
| mmu-miR-27a-3p | Fgf1 | -0.90 | 9.36E-05 | 3.17 | -1.67 |
| mmu-miR-181a-5p | Dusp5 | -0.90 | 9.45E-05 | -3.01 | 1.63 |
| mmu-miR-25-3p | Tbc1d30 | -0.90 | 9.48E-05 | 2.56 | -2.52 |
| mmu-miR-221-3p | Gna14 | -0.90 | 9.58E-05 | 2.18 | -1.90 |
| mmu-miR-27b-3p | Csrp2 | -0.90 | 9.59E-05 | 2.45 | -2.18 |
| mmu-miR-29a-3p | Col3a1 | -0.90 | 9.61E-05 | 2.71 | -2.61 |
| mmu-miR-141-3p | Rora | -0.90 | 9.70E-05 | 4.66 | -2.00 |
| mmu-miR-19b-3p | Calm2 | -0.90 | 9.73E-05 | 3.59 | -1.70 |
| mmu-miR-221-3p | Capsl | -0.90 | 9.76E-05 | 2.18 | -1.70 |
| mmu-miR-106b-5p | Galm | -0.90 | 9.77E-05 | 4.02 | -2.36 |
| mmu-miR-106b-5p | Csrp2 | -0.90 | 9.82E-05 | 4.02 | -2.18 |
| mmu-let-7i-5p | Gmnn | -0.89 | 9.90E-05 | 1.79 | -2.26 |
| mmu-miR-760-3p | Rin1 | -0.89 | 9.98E-05 | -7.15 | 2.03 |
| mmu-miR-375-3p | Psrc1 | -0.89 | 0.000100148 | -2.94 | 3.69 |
| mmu-miR-98-5p | Col3a1 | -0.89 | 0.000100758 | 3.72 | -2.61 |
| mmu-miR-181c-5p | Pxmp2 | -0.89 | 0.000101218 | 3.05 | -3.43 |
| mmu-miR-30a-5p | Rabgap1l | -0.89 | 0.000101894 | 2.87 | -2.04 |
| mmu-miR-93-5p | Elmod1 | -0.89 | 0.000102653 | 2.30 | -1.66 |
| mmu-miR-203-3p | Foxp2 | -0.89 | 0.000103908 | 4.73 | -2.72 |
| mmu-let-7g-5p | 5330417C22Rik | -0.89 | 0.000104221 | 2.05 | -2.44 |
| mmu-miR-22-3p | Cdh26 | -0.89 | 0.000104272 | 2.95 | -2.39 |
| mmu-miR-23b-3p | Aff3 | -0.89 | 0.000107366 | 2.62 | -1.84 |
| mmu-let-7d-3p | Cd300lf | -0.89 | 0.000107646 | -6.28 | 2.83 |
| mmu-miR-203-3p | Sparc | -0.89 | 0.000109658 | 4.73 | -1.95 |
| mmu-miR-27a-3p | Kitl | -0.89 | 0.000111178 | 3.17 | -2.33 |
| mmu-miR-20a-5p | Wdr19 | -0.89 | 0.000111203 | 3.70 | -1.81 |
| mmu-miR-26a-5p | Wnt5a | -0.89 | 0.000111388 | 2.26 | -2.52 |
| mmu-miR-30b-5p | Slc38a1 | -0.89 | 0.000111892 | 2.59 | -3.08 |
| mmu-miR-328-3p | Itga5 | -0.89 | 0.000112244 | -5.59 | 2.47 |
| mmu-miR-340-5p | Rab4a | -0.89 | 0.000113234 | 9.01 | -1.73 |
| mmu-miR-128-3p | Cyp39a1 | -0.89 | 0.000113464 | 2.80 | -1.84 |
| mmu-miR-93-5p | Fam13a | -0.89 | 0.000115812 | 2.30 | -3.21 |
| mmu-miR-26a-5p | Cd36 | -0.89 | 0.000115816 | 2.26 | -1.99 |
| mmu-miR-29a-3p | Sdc2 | -0.89 | 0.000115962 | 2.71 | -2.29 |
| mmu-miR-26a-5p | Col1a2 | -0.89 | 0.00011616 | 2.26 | -2.33 |
| mmu-miR-203-3p | Decr1 | -0.89 | 0.00011698 | 4.73 | -1.64 |
| mmu-miR-218-5p | Calm2 | -0.89 | 0.000117652 | 3.13 | -1.70 |
| mmu-miR-132-3p | Msh2 | -0.89 | 0.000118116 | 2.20 | -1.86 |
| mmu-miR-30e-5p | Msh2 | -0.89 | 0.000118377 | 3.60 | -1.86 |
| mmu-miR-340-5p | Sar1b | -0.89 | 0.000118495 | 9.01 | -1.65 |
| mmu-miR-24-3p | Gpr155 | -0.89 | 0.000118574 | 1.73 | -2.57 |
| mmu-miR-221-3p | Tacr1 | -0.89 | 0.000118711 | 2.18 | -5.28 |
| mmu-miR-30a-5p | Rora | -0.89 | 0.000118719 | 2.87 | -2.00 |
| mmu-miR-26a-5p | Pon1 | -0.89 | 0.000118774 | 2.26 | -3.35 |
| mmu-miR-31-5p | Mlf1 | -0.89 | 0.000119322 | 4.65 | -2.32 |
| mmu-let-7i-5p | Col3a1 | -0.89 | 0.000120071 | 1.79 | -2.61 |
| mmu-miR-23b-3p | Tspan12 | -0.89 | 0.000120479 | 2.62 | -1.95 |
| mmu-miR-92b-5p | Osr1 | -0.89 | 0.000120917 | -6.76 | 1.93 |
| mmu-miR-31-5p | Morn3 | -0.89 | 0.000121244 | 4.65 | -2.84 |
| mmu-miR-26b-5p | Ero1lb | -0.89 | 0.000121271 | 3.80 | -1.73 |
| mmu-miR-17-5p | Galm | -0.89 | 0.000122448 | 3.83 | -2.36 |
| mmu-miR-93-5p | Pfn2 | -0.89 | 0.000122978 | 2.30 | -1.75 |
| mmu-miR-24-3p | Gprasp1 | -0.89 | 0.000123864 | 1.73 | -2.36 |
| mmu-miR-17-5p | Stk33 | -0.89 | 0.000123901 | 3.83 | -2.78 |
| mmu-miR-449c-5p | Myc | -0.89 | 0.000123913 | -3.77 | 2.94 |
| mmu-miR-200b-3p | Adcy2 | -0.89 | 0.000127531 | 2.19 | -2.55 |
| mmu-miR-19b-3p | Slc27a2 | -0.89 | 0.00012916 | 3.59 | -3.23 |
| mmu-miR-26b-5p | Arhgap29 | -0.89 | 0.000130112 | 3.80 | -2.19 |
| mmu-miR-34a-5p | 1190002N15Rik | -0.89 | 0.000130751 | 3.60 | -1.84 |
| mmu-miR-181a-5p | H2-Q4 | -0.89 | 0.000131146 | -3.01 | 1.78 |
| mmu-miR-181a-5p | H2-Q4 | -0.89 | 0.000131146 | -3.01 | 1.78 |
| mmu-miR-155-5p | Fcgbp | -0.89 | 0.000131995 | 1.93 | -2.56 |
| mmu-miR-200b-3p | Pfn2 | -0.89 | 0.000132934 | 2.19 | -1.75 |
| mmu-miR-26a-5p | Arhgap29 | -0.89 | 0.000133049 | 2.26 | -2.19 |
| mmu-miR-93-5p | Stk33 | -0.89 | 0.000133196 | 2.30 | -2.78 |
| mmu-miR-20a-5p | Sar1b | -0.89 | 0.000133247 | 3.70 | -1.65 |
| mmu-miR-106b-5p | 9230110C19Rik | -0.89 | 0.000133627 | 4.02 | -2.63 |
| mmu-miR-25-3p | B3galt2 | -0.89 | 0.000134134 | 2.56 | -1.66 |
| mmu-miR-98-5p | Prkaa2 | -0.89 | 0.000134583 | 3.72 | -2.21 |
| mmu-miR-155-5p | Pam | -0.89 | 0.000136548 | 1.93 | -1.83 |
| mmu-miR-98-5p | Chpt1 | -0.89 | 0.000136802 | 3.72 | -2.38 |
| mmu-miR-23a-3p | Gpx7 | -0.89 | 0.000137454 | 3.01 | -2.02 |
| mmu-miR-23b-3p | Tbc1d30 | -0.89 | 0.000137474 | 2.62 | -2.52 |
| mmu-miR-182-5p | Rhoq | -0.89 | 0.000138409 | 3.32 | -1.59 |
| mmu-let-7b-5p | Plekho1 | -0.89 | 0.000139016 | -3.08 | 1.91 |
| mmu-miR-92b-3p | Itga5 | -0.89 | 0.000139767 | -4.78 | 2.47 |
| mmu-miR-26a-5p | Mme | -0.89 | 0.000140313 | 2.26 | -5.25 |
| mmu-miR-760-3p | Afap1l2 | -0.89 | 0.000140413 | -7.15 | 1.96 |
| mmu-let-7f-5p | Prkaa2 | -0.89 | 0.000140818 | 3.20 | -2.21 |
| mmu-miR-25-3p | Arhgap29 | -0.89 | 0.000141151 | 2.56 | -2.19 |
| mmu-miR-200b-3p | Fundc1 | -0.89 | 0.000141519 | 2.19 | -1.70 |
| mmu-miR-340-5p | Rora | -0.89 | 0.000142708 | 9.01 | -2.00 |
| mmu-miR-20a-5p | B3galt2 | -0.89 | 0.000142832 | 3.70 | -1.66 |
| mmu-miR-23a-3p | Pxmp2 | -0.89 | 0.000142881 | 3.01 | -3.43 |
| mmu-miR-20a-5p | Tmem17 | -0.89 | 0.000143317 | 3.70 | -1.99 |
| mmu-miR-421-3p | Kifap3 | -0.89 | 0.000143644 | 3.10 | -1.75 |
| mmu-miR-27a-3p | Fam69a | -0.89 | 0.00014408 | 3.17 | -2.33 |
| mmu-miR-181c-5p | Kitl | -0.89 | 0.000144185 | 3.05 | -2.33 |
| mmu-miR-200b-3p | Gprasp1 | -0.89 | 0.000146112 | 2.19 | -2.36 |
| mmu-miR-30a-5p | Slc38a1 | -0.88 | 0.000146848 | 2.87 | -3.08 |
| mmu-miR-132-3p | Crls1 | -0.88 | 0.000147034 | 2.20 | -1.62 |
| mmu-miR-22-3p | Padi2 | -0.88 | 0.00014714 | 2.95 | -2.11 |
| mmu-miR-200a-3p | Atp6v1c2 | -0.88 | 0.000147564 | 2.61 | -2.75 |
| mmu-miR-744-5p | Lime1 | -0.88 | 0.000147639 | -3.34 | 2.25 |
| mmu-miR-181c-5p | 9230110C19Rik | -0.88 | 0.0001482 | 3.05 | -2.63 |
| mmu-miR-19b-3p | Arhgap5 | -0.88 | 0.000148595 | 3.59 | -1.85 |
| mmu-miR-200b-3p | Slc27a2 | -0.88 | 0.000149224 | 2.19 | -3.23 |
| mmu-miR-210-5p | Slc4a11 | -0.88 | 0.000149227 | -3.25 | 3.42 |
| mmu-miR-141-3p | 4931406C07Rik | -0.88 | 0.000149864 | 4.66 | -2.42 |
| mmu-let-7f-5p | Chpt1 | -0.88 | 0.000149942 | 3.20 | -2.38 |
| mmu-miR-760-3p | S100a9 | -0.88 | 0.000150779 | -7.15 | 3.60 |
| mmu-miR-27b-3p | Foxp2 | -0.88 | 0.000151467 | 2.45 | -2.72 |
| mmu-miR-93-5p | Pam | -0.88 | 0.000151567 | 2.30 | -1.83 |
| mmu-let-7g-5p | Gmnn | -0.88 | 0.00015358 | 2.05 | -2.26 |
| mmu-miR-17-5p | Ero1lb | -0.88 | 0.000155382 | 3.83 | -1.73 |
| mmu-miR-34a-5p | Cntnap2 | -0.88 | 0.000156093 | 3.60 | -2.56 |
| mmu-miR-26a-5p | Rhoq | -0.88 | 0.000156912 | 2.26 | -1.59 |
| mmu-miR-328-3p | Rin1 | -0.88 | 0.000157264 | -5.59 | 2.03 |
| mmu-miR-106b-5p | Gstk1 | -0.88 | 0.00015778 | 4.02 | -1.71 |
| mmu-miR-15a-5p | Chpt1 | -0.88 | 0.000160449 | 2.29 | -2.38 |
| mmu-miR-23a-3p | Casd1 | -0.88 | 0.00016047 | 3.01 | -2.06 |
| mmu-miR-24-3p | Igfbp5 | -0.88 | 0.000160743 | 1.73 | -1.94 |
| mmu-miR-23a-5p | Gm8909 | -0.88 | 0.000160894 | -2.43 | 3.30 |
| mmu-miR-23b-3p | Eya1 | -0.88 | 0.000161322 | 2.62 | -1.69 |
| mmu-miR-26b-5p | Rora | -0.88 | 0.000161743 | 3.80 | -2.00 |
| mmu-let-7g-5p | Prkaa2 | -0.88 | 0.000163195 | 2.05 | -2.21 |
| mmu-miR-425-5p | Rabgap1l | -0.88 | 0.000164046 | 1.93 | -2.04 |
| mmu-miR-17-5p | Sar1b | -0.88 | 0.000164534 | 3.83 | -1.65 |
| mmu-miR-93-3p | Ero1lb | -0.88 | 0.000165537 | 4.39 | -1.73 |
| mmu-miR-340-5p | Cntnap2 | -0.88 | 0.000166096 | 9.01 | -2.56 |
| mmu-miR-106b-5p | Pfn2 | -0.88 | 0.000166443 | 4.02 | -1.75 |
| mmu-let-7d-3p | Edn1 | -0.88 | 0.000166894 | -6.28 | 2.51 |
| mmu-miR-27b-3p | Eya1 | -0.88 | 0.000167238 | 2.45 | -1.69 |
| mmu-miR-155-5p | Ogn | -0.88 | 0.000168122 | 1.93 | -3.66 |
| mmu-let-7b-5p | S100a9 | -0.88 | 0.000168781 | -3.08 | 3.60 |
| mmu-miR-27b-3p | Scn3b | -0.88 | 0.000168834 | 2.45 | -1.70 |
| mmu-miR-340-5p | Arhgap29 | -0.88 | 0.000169137 | 9.01 | -2.19 |
| mmu-miR-106b-5p | Fam13a | -0.88 | 0.000169835 | 4.02 | -3.21 |
| mmu-miR-340-5p | Ero1lb | -0.88 | 0.000170721 | 9.01 | -1.73 |
| mmu-miR-29a-3p | Rora | -0.88 | 0.000171853 | 2.71 | -2.00 |
| mmu-miR-23b-3p | B3galt2 | -0.88 | 0.000172174 | 2.62 | -1.66 |
| mmu-miR-29a-3p | Dcun1d1 | -0.88 | 0.000173336 | 2.71 | -1.64 |
| mmu-miR-27b-3p | Aff3 | -0.88 | 0.000173767 | 2.45 | -1.84 |
| mmu-miR-23a-3p | Col15a1 | -0.88 | 0.000173778 | 3.01 | -2.31 |
| mmu-miR-106b-5p | Arl4a | -0.88 | 0.000175055 | 4.02 | -1.95 |
| mmu-miR-31-5p | Fundc1 | -0.88 | 0.000176339 | 4.65 | -1.70 |
| mmu-miR-146a-5p | Atp6v1c2 | -0.88 | 0.000177456 | 4.19 | -2.75 |
| mmu-miR-27b-3p | Fam69a | -0.88 | 0.000177593 | 2.45 | -2.33 |
| mmu-miR-340-5p | Msh2 | -0.88 | 0.000177725 | 9.01 | -1.86 |
| mmu-miR-182-5p | Spag16 | -0.88 | 0.000177858 | 3.32 | -3.82 |
| mmu-miR-182-5p | Cadps2 | -0.88 | 0.000178152 | 3.32 | -1.61 |
| mmu-miR-27a-3p | Cyp39a1 | -0.88 | 0.000178502 | 3.17 | -1.84 |
| mmu-let-7d-3p | Lilrb4 | -0.88 | 0.00018045 | -6.28 | 3.54 |
| mmu-miR-193b-3p | Psrc1 | -0.88 | 0.000181258 | -2.01 | 3.69 |
| mmu-let-7g-5p | Chpt1 | -0.88 | 0.000181426 | 2.05 | -2.38 |
| mmu-miR-149-3p | Baiap2l1 | -0.88 | 0.000182573 | -4.06 | 1.93 |
| mmu-miR-26b-5p | Pon1 | -0.88 | 0.000182695 | 3.80 | -3.35 |
| mmu-miR-182-5p | Wnt5a | -0.88 | 0.000184278 | 3.32 | -2.52 |
| mmu-let-7b-5p | Prpf38b | -0.88 | 0.000185885 | -3.08 | 1.63 |
| mmu-miR-141-3p | Ccdc103 | -0.88 | 0.000186255 | 4.66 | -2.01 |
| mmu-miR-218-5p | Prkar2b | -0.88 | 0.000186704 | 3.13 | -2.31 |
| mmu-miR-425-5p | Crls1 | -0.88 | 0.000186831 | 1.93 | -1.62 |
| mmu-miR-106b-5p | Enpp5 | -0.88 | 0.000186886 | 4.02 | -1.89 |
| mmu-miR-30c-5p | Msh2 | -0.88 | 0.00018771 | 2.26 | -1.86 |
| mmu-miR-149-3p | Itga5 | -0.88 | 0.000187897 | -4.06 | 2.47 |
| mmu-miR-429-3p | Casd1 | -0.88 | 0.000188813 | 3.03 | -2.06 |
| mmu-miR-17-5p | Wdr19 | -0.88 | 0.000189026 | 3.83 | -1.81 |
| mmu-miR-23b-3p | Kitl | -0.88 | 0.000191187 | 2.62 | -2.33 |
| mmu-miR-29a-3p | Pxmp2 | -0.88 | 0.000194655 | 2.71 | -3.43 |
| mmu-miR-200a-3p | Sdc2 | -0.88 | 0.000195839 | 2.61 | -2.29 |
| mmu-miR-130a-3p | Acsl3 | -0.88 | 0.00019692 | 2.29 | -1.65 |
| mmu-miR-92a-3p | Sox4 | -0.88 | 0.000199603 | -2.41 | 1.76 |
| mmu-miR-181a-5p | Plau | -0.88 | 0.00020075 | -3.01 | 2.76 |
| mmu-miR-26a-5p | Cdkn1c | -0.88 | 0.000201523 | 2.26 | -2.19 |
| mmu-miR-141-3p | Enpp5 | -0.88 | 0.000201782 | 4.66 | -1.89 |
| mmu-miR-30a-5p | Ttc30a1 | -0.88 | 0.000202058 | 2.87 | -2.40 |
| mmu-let-7b-5p | Aen | -0.88 | 0.000202327 | -3.08 | 1.77 |
| mmu-miR-760-3p | Osr1 | -0.88 | 0.000203094 | -7.15 | 1.93 |
| mmu-miR-23b-3p | Rora | -0.88 | 0.000203146 | 2.62 | -2.00 |
| mmu-miR-203-3p | Tacr1 | -0.88 | 0.000203851 | 4.73 | -5.28 |
| mmu-miR-16-5p | Chpt1 | -0.88 | 0.000205486 | 1.77 | -2.38 |
| mmu-let-7b-5p | Ccnd2 | -0.88 | 0.000207061 | -3.08 | 2.09 |
| mmu-miR-26b-5p | Fmo5 | -0.88 | 0.000207478 | 3.80 | -1.61 |
| mmu-miR-181a-5p | C130026I21Rik | -0.88 | 0.000208222 | -3.01 | 4.65 |
| mmu-miR-181c-5p | Sema3c | -0.88 | 0.000208888 | 3.05 | -1.90 |
| mmu-miR-223-3p | Msh2 | -0.88 | 0.00020912 | 9.15 | -1.86 |
| mmu-miR-19b-3p | Ric3 | -0.87 | 0.000210889 | 3.59 | -2.30 |
| mmu-miR-30c-5p | Map3k1 | -0.87 | 0.000212023 | 2.26 | -1.61 |
| mmu-miR-203-3p | Gpr155 | -0.87 | 0.000212769 | 4.73 | -2.57 |
| mmu-miR-31-5p | Slc25a23 | -0.87 | 0.000213031 | 4.65 | -2.24 |
| mmu-miR-15a-5p | Slitrk6 | -0.87 | 0.000214474 | 2.29 | -3.34 |
| mmu-miR-26b-5p | Cdkn1c | -0.87 | 0.00021816 | 3.80 | -2.19 |
| mmu-miR-19b-3p | Stk33 | -0.87 | 0.000218214 | 3.59 | -2.78 |
| mmu-miR-26a-5p | Gpx7 | -0.87 | 0.000218299 | 2.26 | -2.02 |
| mmu-miR-17-5p | Tmem17 | -0.87 | 0.000219783 | 3.83 | -1.99 |
| mmu-miR-26b-5p | Mme | -0.87 | 0.000222212 | 3.80 | -5.25 |
| mmu-miR-210-5p | Prpf38b | -0.87 | 0.000222904 | -3.25 | 1.63 |
| mmu-miR-877-3p | Baz1a | -0.87 | 0.000224729 | -4.18 | 1.77 |
| mmu-miR-375-3p | Pfkfb3 | -0.87 | 0.000224789 | -2.94 | 1.73 |
| mmu-miR-106b-5p | Ero1lb | -0.87 | 0.000225257 | 4.02 | -1.73 |
| mmu-miR-30e-5p | Abi3bp | -0.87 | 0.000225662 | 3.60 | -3.56 |
| mmu-miR-340-5p | Casd1 | -0.87 | 0.000226531 | 9.01 | -2.06 |
| mmu-miR-185-3p | Has3 | -0.87 | 0.000226718 | -3.01 | 1.63 |
| mmu-let-7i-5p | Ttc26 | -0.87 | 0.000227131 | 1.79 | -1.81 |
| mmu-miR-93-5p | B3galt2 | -0.87 | 0.000227296 | 2.30 | -1.66 |
| mmu-miR-20a-5p | Rnf128 | -0.87 | 0.000227396 | 3.70 | -1.91 |
| mmu-let-7i-5p | Akap6 | -0.87 | 0.000228302 | 1.79 | -2.33 |
| mmu-let-7b-5p | Mesdc1 | -0.87 | 0.000228706 | -3.08 | 1.83 |
| mmu-miR-29a-3p | Col15a1 | -0.87 | 0.000228754 | 2.71 | -2.31 |
| mmu-miR-93-5p | Galm | -0.87 | 0.000230056 | 2.30 | -2.36 |
| mmu-miR-30e-5p | Slc38a1 | -0.87 | 0.000230101 | 3.60 | -3.08 |
| mmu-miR-200b-3p | Galm | -0.87 | 0.000230378 | 2.19 | -2.36 |
| mmu-miR-149-3p | Ptafr | -0.87 | 0.00023111 | -4.06 | 4.38 |
| mmu-miR-326-3p | Arid5a | -0.87 | 0.000231618 | -1.76 | 1.86 |
| mmu-miR-25-3p | Col1a2 | -0.87 | 0.000232095 | 2.56 | -2.33 |
| mmu-miR-182-5p | Prkaa2 | -0.87 | 0.000233347 | 3.32 | -2.21 |
| mmu-miR-200a-3p | Arl4a | -0.87 | 0.000234767 | 2.61 | -1.95 |
| mmu-miR-106b-5p | 4931406C07Rik | -0.87 | 0.00023524 | 4.02 | -2.42 |
| mmu-miR-23a-3p | Msrb2 | -0.87 | 0.000235898 | 3.01 | -2.49 |
| mmu-miR-425-5p | Cdh26 | -0.87 | 0.000237075 | 1.93 | -2.39 |
| mmu-miR-26a-5p | Ero1lb | -0.87 | 0.000237443 | 2.26 | -1.73 |
| mmu-miR-15a-5p | Fgf1 | -0.87 | 0.000239126 | 2.29 | -1.67 |
| mmu-miR-30b-5p | Rora | -0.87 | 0.000240453 | 2.59 | -2.00 |
| mmu-miR-203-3p | Cdhr3 | -0.87 | 0.000240986 | 4.73 | -1.83 |
| mmu-miR-25-3p | Wnt5a | -0.87 | 0.000242443 | 2.56 | -2.52 |
| mmu-let-7i-5p | Ghr | -0.87 | 0.000243481 | 1.79 | -2.23 |
| mmu-miR-200b-3p | Fmo3 | -0.87 | 0.000245251 | 2.19 | -2.11 |
| mmu-miR-27a-3p | Csrp2 | -0.87 | 0.000245337 | 3.17 | -2.18 |
| mmu-miR-203-3p | Setbp1 | -0.87 | 0.000245498 | 4.73 | -1.63 |
| mmu-miR-200b-3p | Myb | -0.87 | 0.000246713 | 2.19 | -1.98 |
| mmu-miR-34a-5p | Myl4 | -0.87 | 0.000247895 | 3.60 | -2.52 |
| mmu-miR-92b-5p | Ddit4 | -0.87 | 0.000248284 | -6.76 | 1.87 |
| mmu-miR-223-3p | Rabgap1l | -0.87 | 0.000249327 | 9.15 | -2.04 |
| mmu-miR-182-5p | Cdkn1c | -0.87 | 0.000249757 | 3.32 | -2.19 |
| mmu-miR-200a-3p | Wnt5a | -0.87 | 0.00024979 | 2.61 | -2.52 |
| mmu-let-7f-5p | Col3a1 | -0.87 | 0.000250102 | 3.20 | -2.61 |
| mmu-miR-23a-3p | Aldh1a1 | -0.87 | 0.00025137 | 3.01 | -1.70 |
| mmu-miR-31-5p | Csrp2 | -0.87 | 0.000252242 | 4.65 | -2.18 |
| mmu-miR-19b-3p | Dtna | -0.87 | 0.000252672 | 3.59 | -1.62 |
| mmu-miR-30e-5p | Ttc30a1 | -0.87 | 0.000254267 | 3.60 | -2.40 |
| mmu-miR-221-3p | Aldh1a1 | -0.87 | 0.000254482 | 2.18 | -1.70 |
| mmu-miR-24-3p | Msrb2 | -0.87 | 0.000255343 | 1.73 | -2.49 |
| mmu-miR-27a-3p | Elmod1 | -0.87 | 0.00025961 | 3.17 | -1.66 |
| mmu-miR-26b-5p | Wnt5a | -0.87 | 0.000260494 | 3.80 | -2.52 |
| mmu-miR-31-5p | Gpx7 | -0.87 | 0.000260712 | 4.65 | -2.02 |
| mmu-miR-24-3p | Tacr1 | -0.87 | 0.000261375 | 1.73 | -5.28 |
| mmu-miR-27b-3p | Mdh1b | -0.87 | 0.000261841 | 2.45 | -2.10 |
| mmu-miR-218-5p | Rabgap1l | -0.87 | 0.000262375 | 3.13 | -2.04 |
| mmu-miR-27a-3p | Myh10 | -0.87 | 0.000263154 | 3.17 | -1.69 |
| mmu-let-7d-3p | S100a9 | -0.87 | 0.000263253 | -6.28 | 3.60 |
| mmu-miR-148a-3p | Pgrmc1 | -0.87 | 0.00026364 | 2.94 | -2.17 |
| mmu-miR-30b-5p | Arl4a | -0.87 | 0.000266917 | 2.59 | -1.95 |
| mmu-miR-93-5p | Slc25a23 | -0.87 | 0.000267105 | 2.30 | -2.24 |
| mmu-miR-10a-5p | Msrb2 | -0.87 | 0.00026715 | 2.87 | -2.49 |
| mmu-miR-30e-5p | Rora | -0.87 | 0.000268126 | 3.60 | -2.00 |
| mmu-let-7b-5p | Prss22 | -0.87 | 0.000270522 | -3.08 | 2.11 |
| mmu-miR-22-3p | Fmo5 | -0.87 | 0.000273028 | 2.95 | -1.61 |
| mmu-miR-27b-3p | Cyp39a1 | -0.87 | 0.000273339 | 2.45 | -1.84 |
| mmu-miR-26b-5p | Gpx7 | -0.87 | 0.000273346 | 3.80 | -2.02 |
| mmu-miR-19b-3p | Enpp5 | -0.87 | 0.000275022 | 3.59 | -1.89 |
| mmu-miR-20a-5p | Slc4a5 | -0.87 | 0.000275317 | 3.70 | -3.03 |
| mmu-miR-210-5p | AA986860 | -0.87 | 0.000276026 | -3.25 | 1.81 |
| mmu-miR-106b-5p | Rora | -0.87 | 0.000277835 | 4.02 | -2.00 |
| mmu-miR-221-3p | Foxa2 | -0.87 | 0.000278771 | 2.18 | -2.09 |
| mmu-miR-182-5p | Aldh1a1 | -0.87 | 0.000282347 | 3.32 | -1.70 |
| mmu-let-7g-5p | Tnfsf10 | -0.87 | 0.000284716 | 2.05 | -1.92 |
| mmu-miR-200b-3p | Tmem17 | -0.87 | 0.000284748 | 2.19 | -1.99 |
| mmu-miR-421-3p | Ric3 | -0.87 | 0.000285013 | 3.10 | -2.30 |
| mmu-miR-183-3p | Cntnap2 | -0.87 | 0.000285171 | 4.74 | -2.56 |
| mmu-miR-203-3p | Dixdc1 | -0.87 | 0.000286105 | 4.73 | -1.73 |
| mmu-miR-27b-3p | Elmod1 | -0.87 | 0.000286995 | 2.45 | -1.66 |
| mmu-miR-181a-5p | Ddit4 | -0.87 | 0.00028767 | -3.01 | 1.87 |
| mmu-miR-130a-3p | Sar1b | -0.87 | 0.00028872 | 2.29 | -1.65 |
| mmu-miR-25-3p | Kcnrg | -0.87 | 0.000289548 | 2.56 | -2.07 |
| mmu-miR-30a-5p | 1190002N15Rik | -0.87 | 0.000289726 | 2.87 | -1.84 |
| mmu-miR-210-5p | Plcd3 | -0.87 | 0.000290516 | -3.25 | 1.97 |
| mmu-miR-23b-3p | Ulk4 | -0.87 | 0.000290675 | 2.62 | -1.85 |
| mmu-miR-143-3p | Calm2 | -0.87 | 0.000291318 | 2.79 | -1.70 |
| mmu-miR-26a-5p | Cadm1 | -0.86 | 0.000292981 | 2.26 | -1.72 |
| mmu-miR-27b-3p | Myh10 | -0.86 | 0.000295339 | 2.45 | -1.69 |
| mmu-miR-181c-5p | Akap6 | -0.86 | 0.000295551 | 3.05 | -2.33 |
| mmu-miR-218-5p | Ogn | -0.86 | 0.000295608 | 3.13 | -3.66 |
| mmu-miR-433-3p | Padi4 | -0.86 | 0.000298141 | -4.72 | 1.72 |
| mmu-miR-23b-3p | Dtna | -0.86 | 0.000299438 | 2.62 | -1.62 |
| mmu-miR-223-3p | Ptar1 | -0.86 | 0.000300497 | 9.15 | -3.27 |
| mmu-miR-19b-3p | Ptprg | -0.86 | 0.00030138 | 3.59 | -1.60 |
| mmu-miR-23a-5p | Nlrc5 | -0.86 | 0.000301447 | -2.43 | 4.26 |
| mmu-miR-155-5p | Fmo5 | -0.86 | 0.000303583 | 1.93 | -1.61 |
| mmu-miR-143-3p | Casc1 | -0.86 | 0.000306092 | 2.79 | -1.66 |
| mmu-miR-433-3p | Krt20 | -0.86 | 0.000306452 | -4.72 | 5.74 |
| mmu-miR-877-3p | Fes | -0.86 | 0.000308122 | -4.18 | 1.72 |
| mmu-miR-22-3p | Fstl1 | -0.86 | 0.000309045 | 2.95 | -2.02 |
| mmu-miR-183-3p | Rora | -0.86 | 0.000313404 | 4.74 | -2.00 |
| mmu-miR-141-3p | Arl4a | -0.86 | 0.000316802 | 4.66 | -1.95 |
| mmu-miR-30b-5p | 4931406C07Rik | -0.86 | 0.000317019 | 2.59 | -2.42 |
| mmu-miR-141-3p | Col15a1 | -0.86 | 0.000317866 | 4.66 | -2.31 |
| mmu-miR-146a-5p | Lum | -0.86 | 0.000318111 | 4.19 | -4.90 |
| mmu-miR-328-3p | Csf2rb2 | -0.86 | 0.000318336 | -5.59 | 2.57 |
| mmu-let-7i-5p | Col15a1 | -0.86 | 0.000319851 | 1.79 | -2.31 |
| mmu-miR-34a-5p | Elmod1 | -0.86 | 0.000320091 | 3.60 | -1.66 |
| mmu-miR-26b-5p | Tbc1d30 | -0.86 | 0.000321209 | 3.80 | -2.52 |
| mmu-miR-23a-3p | B3galt2 | -0.86 | 0.000321729 | 3.01 | -1.66 |
| mmu-miR-182-5p | Mlf1 | -0.86 | 0.000322888 | 3.32 | -2.32 |
| mmu-miR-29a-3p | Fstl1 | -0.86 | 0.000323474 | 2.71 | -2.02 |
| mmu-miR-93-5p | Tmem17 | -0.86 | 0.000324207 | 2.30 | -1.99 |
| mmu-miR-15a-5p | Sms | -0.86 | 0.000325115 | 2.29 | -2.09 |
| mmu-miR-17-5p | Gstk1 | -0.86 | 0.000328404 | 3.83 | -1.71 |
| mmu-miR-29a-3p | Col1a2 | -0.86 | 0.000330098 | 2.71 | -2.33 |
| mmu-miR-15a-5p | Ppic | -0.86 | 0.000330388 | 2.29 | -2.39 |
| mmu-miR-29a-3p | Rab4a | -0.86 | 0.000330389 | 2.71 | -1.73 |
| mmu-miR-22-3p | Crls1 | -0.86 | 0.000332052 | 2.95 | -1.62 |
| mmu-let-7d-3p | Apobec1 | -0.86 | 0.000332617 | -6.28 | 1.74 |
| mmu-miR-30e-5p | Pfn2 | -0.86 | 0.000334244 | 3.60 | -1.75 |
| mmu-miR-128-3p | Rora | -0.86 | 0.000335374 | 2.80 | -2.00 |
| mmu-miR-25-3p | Prkar1b | -0.86 | 0.000335451 | 2.56 | -1.97 |
| mmu-miR-16-5p | Fgf1 | -0.86 | 0.000336231 | 1.77 | -1.67 |
| mmu-miR-181a-5p | Csf3r | -0.86 | 0.000337453 | -3.01 | 3.75 |
| mmu-miR-429-3p | Adcy2 | -0.86 | 0.00033802 | 3.03 | -2.55 |
| mmu-miR-183-3p | Gp2 | -0.86 | 0.00033839 | 4.74 | -4.65 |
| mmu-miR-26a-5p | Foxp2 | -0.86 | 0.000339873 | 2.26 | -2.72 |
| mmu-miR-29a-3p | Ric3 | -0.86 | 0.000340561 | 2.71 | -2.30 |
| mmu-miR-132-3p | Rora | -0.86 | 0.000340729 | 2.20 | -2.00 |
| mmu-miR-30e-5p | Fam13a | -0.86 | 0.000341456 | 3.60 | -3.21 |
| mmu-miR-128-3p | Scn3b | -0.86 | 0.000344346 | 2.80 | -1.70 |
| mmu-miR-23a-3p | Kitl | -0.86 | 0.000344713 | 3.01 | -2.33 |
| mmu-miR-340-5p | Cyp4v3 | -0.86 | 0.000345637 | 9.01 | -1.71 |
| mmu-miR-223-3p | Dnajb13 | -0.86 | 0.00034881 | 9.15 | -1.82 |
| mmu-miR-19b-3p | Six4 | -0.86 | 0.000350037 | 3.59 | -1.65 |
| mmu-miR-221-3p | Acot1 | -0.86 | 0.000354981 | 2.18 | -2.12 |
| mmu-miR-221-3p | Acot1 | -0.86 | 0.000354981 | 2.18 | -2.12 |
| mmu-miR-877-3p | Sp110 | -0.86 | 0.000355729 | -4.18 | 2.64 |
| mmu-miR-200a-3p | Slc27a2 | -0.86 | 0.000356422 | 2.61 | -3.23 |
| mmu-miR-155-5p | Rcbtb2 | -0.86 | 0.000356601 | 1.93 | -1.59 |
| mmu-miR-20a-5p | Elmod1 | -0.86 | 0.000357571 | 3.70 | -1.66 |
| mmu-miR-24-3p | Cdkn1c | -0.86 | 0.000358515 | 1.73 | -2.19 |
| mmu-miR-141-3p | Arhgap29 | -0.86 | 0.000360487 | 4.66 | -2.19 |
| mmu-miR-30c-5p | Rabgap1l | -0.86 | 0.000361157 | 2.26 | -2.04 |
| mmu-miR-429-3p | Fundc1 | -0.86 | 0.000361366 | 3.03 | -1.70 |
| mmu-let-7b-5p | Myc | -0.86 | 0.000363883 | -3.08 | 2.94 |
| mmu-miR-17-5p | B3galt2 | -0.86 | 0.00036452 | 3.83 | -1.66 |
| mmu-miR-155-5p | Rhoq | -0.86 | 0.000367209 | 1.93 | -1.59 |
| mmu-miR-340-5p | Csrp2 | -0.86 | 0.000369466 | 9.01 | -2.18 |
| mmu-let-7d-3p | Prpf38b | -0.86 | 0.000371126 | -6.28 | 1.63 |
| mmu-miR-210-5p | Hk3 | -0.86 | 0.000372529 | -3.25 | 4.21 |
| mmu-miR-128-3p | Kitl | -0.86 | 0.000374381 | 2.80 | -2.33 |
| mmu-miR-182-5p | Dcn | -0.86 | 0.000374902 | 3.32 | -4.28 |
| mmu-miR-22-3p | Cyp39a1 | -0.86 | 0.00037689 | 2.95 | -1.84 |
| mmu-miR-429-3p | Fstl1 | -0.86 | 0.000380807 | 3.03 | -2.02 |
| mmu-miR-433-3p | C130026I21Rik | -0.86 | 0.000381057 | -4.72 | 4.65 |
| mmu-miR-92a-3p | Dusp5 | -0.86 | 0.000381668 | -2.41 | 1.63 |
| mmu-miR-27a-3p | Foxp2 | -0.86 | 0.000381838 | 3.17 | -2.72 |
| mmu-miR-340-5p | Arl4a | -0.86 | 0.00038352 | 9.01 | -1.95 |
| mmu-miR-30e-5p | Arl4a | -0.86 | 0.000383719 | 3.60 | -1.95 |
| mmu-miR-182-5p | Arhgap29 | -0.86 | 0.000384613 | 3.32 | -2.19 |
| mmu-miR-30a-5p | Arl4a | -0.86 | 0.000389653 | 2.87 | -1.95 |
| mmu-miR-181c-5p | Rgs22 | -0.86 | 0.000390499 | 3.05 | -2.80 |
| mmu-miR-141-3p | Fmo3 | -0.86 | 0.000392487 | 4.66 | -2.11 |
| mmu-miR-29a-3p | Nme5 | -0.86 | 0.000394419 | 2.71 | -2.53 |
| mmu-miR-182-5p | Adcy2 | -0.86 | 0.000395965 | 3.32 | -2.55 |
| mmu-miR-504-5p | Fscn1 | -0.86 | 0.000397002 | -4.12 | 2.28 |
| mmu-miR-155-5p | Atp1a2 | -0.86 | 0.00039732 | 1.93 | -4.08 |
| mmu-miR-25-3p | Abhd3 | -0.86 | 0.000397958 | 2.56 | -2.19 |
| mmu-miR-155-5p | Tspan13 | -0.85 | 0.000398677 | 1.93 | -2.22 |
| mmu-miR-425-5p | Dmbt1 | -0.85 | 0.000401253 | 1.93 | -3.59 |
| mmu-miR-340-5p | Fmo3 | -0.85 | 0.000402835 | 9.01 | -2.11 |
| mmu-miR-181c-5p | Igf2bp2 | -0.85 | 0.000403406 | 3.05 | -2.19 |
| mmu-miR-22-3p | Mlf1 | -0.85 | 0.000403454 | 2.95 | -2.32 |
| mmu-let-7i-5p | Col1a2 | -0.85 | 0.000404326 | 1.79 | -2.33 |
| mmu-miR-30a-5p | Pfn2 | -0.85 | 0.000405677 | 2.87 | -1.75 |
| mmu-miR-200a-3p | 4931406C07Rik | -0.85 | 0.000406885 | 2.61 | -2.42 |
| mmu-miR-106b-5p | Rnf128 | -0.85 | 0.000409421 | 4.02 | -1.91 |
| mmu-miR-181c-5p | Mdh1b | -0.85 | 0.000410086 | 3.05 | -2.10 |
| mmu-miR-25-3p | Gdpd2 | -0.85 | 0.000412998 | 2.56 | -1.78 |
| mmu-miR-29a-3p | Morn3 | -0.85 | 0.000413823 | 2.71 | -2.84 |
| mmu-miR-27a-3p | Mdh1b | -0.85 | 0.000415169 | 3.17 | -2.10 |
| mmu-miR-93-5p | Rnf128 | -0.85 | 0.000416081 | 2.30 | -1.91 |
| mmu-miR-181a-5p | Cpne2 | -0.85 | 0.000416867 | -3.01 | 2.13 |
| mmu-miR-218-5p | Mme | -0.85 | 0.000418921 | 3.13 | -5.25 |
| mmu-miR-31-5p | Elmod1 | -0.85 | 0.000419632 | 4.65 | -1.66 |
| mmu-miR-429-3p | Pfn2 | -0.85 | 0.00042162 | 3.03 | -1.75 |
| mmu-miR-25-3p | Aff3 | -0.85 | 0.000425378 | 2.56 | -1.84 |
| mmu-miR-93-5p | Csrp2 | -0.85 | 0.000425786 | 2.30 | -2.18 |
| mmu-miR-433-3p | Ccl3 | -0.85 | 0.00042691 | -4.72 | 3.93 |
| mmu-miR-200b-3p | Aff3 | -0.85 | 0.000427323 | 2.19 | -1.84 |
| mmu-let-7b-5p | Bax | -0.85 | 0.000430744 | -3.08 | 1.86 |
| mmu-miR-27b-3p | Dtna | -0.85 | 0.00043333 | 2.45 | -1.62 |
| mmu-miR-30b-5p | Pfn2 | -0.85 | 0.0004359 | 2.59 | -1.75 |
| mmu-miR-340-5p | Fundc1 | -0.85 | 0.000436728 | 9.01 | -1.70 |
| mmu-miR-182-5p | Myb | -0.85 | 0.000436833 | 3.32 | -1.98 |
| mmu-miR-34a-5p | Aff3 | -0.85 | 0.000439064 | 3.60 | -1.84 |
| mmu-miR-15a-5p | Calm2 | -0.85 | 0.000440519 | 2.29 | -1.70 |
| mmu-miR-433-3p | Csf3r | -0.85 | 0.000440751 | -4.72 | 3.75 |
| mmu-miR-143-3p | Rabgap1l | -0.85 | 0.000441308 | 2.79 | -2.04 |
| mmu-miR-19b-3p | Lclat1 | -0.85 | 0.000441504 | 3.59 | -1.84 |
| mmu-let-7i-5p | Gpx7 | -0.85 | 0.000443076 | 1.79 | -2.02 |
| mmu-miR-182-5p | Fam13a | -0.85 | 0.000445986 | 3.32 | -3.21 |
| mmu-miR-27b-3p | Sec14l3 | -0.85 | 0.000447331 | 2.45 | -2.04 |
| mmu-miR-26a-5p | B4galt4 | -0.85 | 0.000448952 | 2.26 | -2.19 |
| mmu-miR-26a-5p | Cfh | -0.85 | 0.000449188 | 2.26 | -3.58 |
| mmu-let-7i-5p | Foxp2 | -0.85 | 0.000449889 | 1.79 | -2.72 |
| mmu-miR-10a-5p | Dnajb13 | -0.85 | 0.000450631 | 2.87 | -1.82 |
| mmu-miR-23a-3p | Foxp2 | -0.85 | 0.000450832 | 3.01 | -2.72 |
| mmu-miR-98-5p | Gpx7 | -0.85 | 0.00045145 | 3.72 | -2.02 |
| mmu-miR-205-3p | Pam | -0.85 | 0.000452646 | 6.09 | -1.83 |
| mmu-miR-182-5p | Morn3 | -0.85 | 0.00045287 | 3.32 | -2.84 |
| mmu-miR-182-5p | Pcca | -0.85 | 0.000453348 | 3.32 | -1.94 |
| mmu-miR-760-3p | Ccrl2 | -0.85 | 0.000453631 | -7.15 | 2.47 |
| mmu-miR-26a-5p | Fmo5 | -0.85 | 0.000457119 | 2.26 | -1.61 |
| mmu-miR-130a-3p | Enpp5 | -0.85 | 0.000458852 | 2.29 | -1.89 |
| mmu-miR-504-5p | Slurp1 | -0.85 | 0.000459797 | -4.12 | 1.83 |
| mmu-let-7b-5p | Ccl3 | -0.85 | 0.000460788 | -3.08 | 3.93 |
| mmu-miR-181a-5p | Nrp2 | -0.85 | 0.000461053 | -3.01 | 1.65 |
| mmu-miR-27a-3p | Eya1 | -0.85 | 0.000462681 | 3.17 | -1.69 |
| mmu-miR-200b-3p | Scn3b | -0.85 | 0.000464385 | 2.19 | -1.70 |
| mmu-miR-326-3p | Ankrd11 | -0.85 | 0.000466089 | -1.76 | 1.76 |
| mmu-miR-340-5p | Mlf1 | -0.85 | 0.000470389 | 9.01 | -2.32 |
| mmu-let-7i-5p | Ccdc60 | -0.85 | 0.000471445 | 1.79 | -1.61 |
| mmu-miR-29a-3p | Eln | -0.85 | 0.000471773 | 2.71 | -2.85 |
| mmu-miR-200a-3p | Kifap3 | -0.85 | 0.000474611 | 2.61 | -1.75 |
| mmu-miR-26a-5p | Mdh1b | -0.85 | 0.000479187 | 2.26 | -2.10 |
| mmu-miR-106b-5p | Sar1b | -0.85 | 0.000483222 | 4.02 | -1.65 |
| mmu-miR-149-3p | Nrp2 | -0.85 | 0.000484727 | -4.06 | 1.65 |
| mmu-let-7d-3p | Socs1 | -0.85 | 0.000484734 | -6.28 | 1.90 |
| mmu-miR-27a-3p | Scn3b | -0.85 | 0.000484883 | 3.17 | -1.70 |
| mmu-miR-877-5p | Krt16 | -0.85 | 0.000485631 | -4.79 | 4.68 |
| mmu-miR-27b-3p | St3gal6 | -0.85 | 0.000487666 | 2.45 | -2.31 |
| mmu-miR-23b-3p | Intu | -0.85 | 0.000490888 | 2.62 | -1.73 |
| mmu-miR-200a-3p | Rora | -0.85 | 0.000491691 | 2.61 | -2.00 |
| mmu-miR-20a-5p | Pkia | -0.85 | 0.000491819 | 3.70 | -2.03 |
| mmu-miR-421-3p | Casd1 | -0.85 | 0.000493048 | 3.10 | -2.06 |
| mmu-miR-92a-3p | Has3 | -0.85 | 0.000494139 | -2.41 | 1.63 |
| mmu-miR-16-5p | Slitrk6 | -0.85 | 0.000494889 | 1.77 | -3.34 |
| mmu-miR-98-5p | Col15a1 | -0.85 | 0.000494944 | 3.72 | -2.31 |
| mmu-miR-23a-5p | Padi4 | -0.85 | 0.000494992 | -2.43 | 1.72 |
| mmu-miR-23a-3p | Tbc1d30 | -0.85 | 0.000497289 | 3.01 | -2.52 |
| mmu-miR-148a-3p | Msh2 | -0.85 | 0.000505949 | 2.94 | -1.86 |
| mmu-miR-182-5p | Dcun1d1 | -0.85 | 0.000506728 | 3.32 | -1.64 |
| mmu-miR-30b-5p | Enpp5 | -0.85 | 0.000508848 | 2.59 | -1.89 |
| mmu-miR-34a-5p | 5330417C22Rik | -0.85 | 0.000510066 | 3.60 | -2.44 |
| mmu-miR-141-3p | Foxa2 | -0.85 | 0.000512543 | 4.66 | -2.09 |
| mmu-miR-200a-3p | Fmo3 | -0.85 | 0.000513077 | 2.61 | -2.11 |
| mmu-let-7f-5p | Ttc30a1 | -0.85 | 0.000514504 | 3.20 | -2.40 |
| mmu-miR-205-3p | Msh2 | -0.85 | 0.000516616 | 6.09 | -1.86 |
| mmu-miR-26a-5p | Tbc1d30 | -0.85 | 0.000517822 | 2.26 | -2.52 |
| mmu-miR-98-5p | Col1a2 | -0.85 | 0.000517983 | 3.72 | -2.33 |
| mmu-miR-24-3p | Ppil6 | -0.85 | 0.000518784 | 1.73 | -2.27 |
| mmu-miR-200b-3p | Cadm1 | -0.85 | 0.000519275 | 2.19 | -1.72 |
| mmu-miR-128-3p | Foxp2 | -0.85 | 0.000522727 | 2.80 | -2.72 |
| mmu-miR-340-5p | Cdh26 | -0.85 | 0.000523724 | 9.01 | -2.39 |
| mmu-miR-30b-5p | Fam13a | -0.85 | 0.000524374 | 2.59 | -3.21 |
| mmu-miR-378c | Rora | -0.85 | 0.000525328 | 4.40 | -2.00 |
| mmu-miR-421-3p | Arhgap5 | -0.85 | 0.000526929 | 3.10 | -1.85 |
| mmu-miR-30a-3p | Lclat1 | -0.84 | 0.000530578 | 1.94 | -1.84 |
| mmu-miR-34a-5p | Rora | -0.84 | 0.000531471 | 3.60 | -2.00 |
| mmu-miR-31-5p | Ccdc60 | -0.84 | 0.000536009 | 4.65 | -1.61 |
| mmu-miR-30e-5p | 4931406C07Rik | -0.84 | 0.000539387 | 3.60 | -2.42 |
| mmu-miR-23b-3p | Tmem67 | -0.84 | 0.000541918 | 2.62 | -2.19 |
| mmu-miR-24-3p | Hp | -0.84 | 0.000544593 | 1.73 | -1.65 |
| mmu-miR-30a-5p | Tmem35 | -0.84 | 0.000546794 | 2.87 | -1.94 |
| mmu-miR-10a-5p | Mettl7a1 | -0.84 | 0.000547884 | 2.87 | -1.71 |
| mmu-miR-429-3p | Rwdd3 | -0.84 | 0.000549016 | 3.03 | -2.36 |
| mmu-let-7g-5p | Col3a1 | -0.84 | 0.000550667 | 2.05 | -2.61 |
| mmu-miR-429-3p | 6-Sep | -0.84 | 0.00055479 | 3.03 | -1.64 |
| mmu-miR-421-3p | Gmnn | -0.84 | 0.000555034 | 3.10 | -2.26 |
| mmu-miR-141-3p | Tmem17 | -0.84 | 0.000557381 | 4.66 | -1.99 |
| mmu-miR-221-3p | Kit | -0.84 | 0.00055894 | 2.18 | -1.91 |
| mmu-miR-98-5p | Decr1 | -0.84 | 0.000561261 | 3.72 | -1.64 |
| mmu-miR-141-3p | Myh10 | -0.84 | 0.000562759 | 4.66 | -1.69 |
| mmu-miR-19b-3p | Pfn2 | -0.84 | 0.00056303 | 3.59 | -1.75 |
| mmu-miR-128-3p | Csrp2 | -0.84 | 0.000564306 | 2.80 | -2.18 |
| mmu-miR-92a-3p | Ddit4 | -0.84 | 0.000566155 | -2.41 | 1.87 |
| mmu-miR-143-3p | Reg3g | -0.84 | 0.000566794 | 2.79 | -2.74 |
| mmu-miR-218-5p | Sdc2 | -0.84 | 0.000569413 | 3.13 | -2.29 |
| mmu-miR-181c-5p | Six4 | -0.84 | 0.000569848 | 3.05 | -1.65 |
| mmu-miR-149-3p | N4bp3 | -0.84 | 0.000571125 | -4.06 | 1.61 |
| mmu-miR-340-5p | 1190002N15Rik | -0.84 | 0.000572366 | 9.01 | -1.84 |
| mmu-miR-93-5p | Wdr19 | -0.84 | 0.000576297 | 2.30 | -1.81 |
| mmu-miR-19b-3p | Slc25a34 | -0.84 | 0.000576305 | 3.59 | -1.68 |
| mmu-miR-23a-5p | Plau | -0.84 | 0.000576729 | -2.43 | 2.76 |
| mmu-miR-26b-5p | Col1a2 | -0.84 | 0.000579265 | 3.80 | -2.33 |
| mmu-miR-132-3p | Prdx1 | -0.84 | 0.000582604 | 2.20 | -1.59 |
| mmu-miR-223-3p | Ogn | -0.84 | 0.000592058 | 9.15 | -3.66 |
| mmu-miR-141-3p | Lipf | -0.84 | 0.00059208 | 4.66 | -6.24 |
| mmu-miR-26a-5p | Tigd2 | -0.84 | 0.000592404 | 2.26 | -1.67 |
| mmu-miR-23a-3p | Aff3 | -0.84 | 0.000593098 | 3.01 | -1.84 |
| mmu-miR-26b-5p | Mdh1b | -0.84 | 0.000593331 | 3.80 | -2.10 |
| mmu-miR-181c-5p | Dtna | -0.84 | 0.000595859 | 3.05 | -1.62 |
| mmu-miR-31-5p | Prkar1b | -0.84 | 0.000596526 | 4.65 | -1.97 |
| mmu-miR-26b-5p | B4galt4 | -0.84 | 0.000598887 | 3.80 | -2.19 |
| mmu-miR-31-5p | Sez6l2 | -0.84 | 0.000600168 | 4.65 | -3.64 |
| mmu-miR-181a-5p | Tubb6 | -0.84 | 0.000603508 | -3.01 | 1.79 |
| mmu-miR-106b-5p | Zbtb33 | -0.84 | 0.000603713 | 4.02 | -1.68 |
| mmu-miR-200a-3p | Enpp5 | -0.84 | 0.000605135 | 2.61 | -1.89 |
| mmu-miR-340-5p | Ptar1 | -0.84 | 0.000606142 | 9.01 | -3.27 |
| mmu-let-7f-5p | Slc25a23 | -0.84 | 0.000606239 | 3.20 | -2.24 |
| mmu-miR-181c-5p | Sema3b | -0.84 | 0.000606987 | 3.05 | -1.61 |
| mmu-miR-182-5p | Sh3bgrl | -0.84 | 0.000611987 | 3.32 | -1.64 |
| mmu-miR-218-5p | Ric3 | -0.84 | 0.000614749 | 3.13 | -2.30 |
| mmu-miR-143-3p | Scgb1a1 | -0.84 | 0.00061544 | 2.79 | -2.57 |
| mmu-let-7b-5p | Muc4 | -0.84 | 0.000625406 | -3.08 | 1.94 |
| mmu-miR-182-5p | Rwdd3 | -0.84 | 0.000625497 | 3.32 | -2.36 |
| mmu-miR-200a-3p | Foxa2 | -0.84 | 0.000631297 | 2.61 | -2.09 |
| mmu-miR-26b-5p | Foxp2 | -0.84 | 0.000632287 | 3.80 | -2.72 |
| mmu-miR-30a-5p | Fam13a | -0.84 | 0.000634075 | 2.87 | -3.21 |
| mmu-miR-25-5p | Ripk3 | -0.84 | 0.000639549 | -2.35 | 1.70 |
| mmu-let-7d-3p | Plekho1 | -0.84 | 0.000639643 | -6.28 | 1.91 |
| mmu-miR-23a-3p | Rora | -0.84 | 0.000639905 | 3.01 | -2.00 |
| mmu-miR-218-5p | Dcn | -0.84 | 0.000642729 | 3.13 | -4.28 |
| mmu-miR-130a-3p | Prkaa2 | -0.84 | 0.000643785 | 2.29 | -2.21 |
| mmu-miR-20a-5p | St8sia2 | -0.84 | 0.000648115 | 3.70 | -1.66 |
| mmu-miR-30a-5p | Htra3 | -0.84 | 0.000652629 | 2.87 | -1.92 |
| mmu-miR-23a-5p | Rgl2 | -0.84 | 0.000652873 | -2.43 | 1.80 |
| mmu-miR-744-5p | Tsen54 | -0.84 | 0.000653734 | -3.34 | 1.59 |
| mmu-miR-200a-3p | Ccdc103 | -0.84 | 0.000659327 | 2.61 | -2.01 |
| mmu-miR-141-3p | Plekhb1 | -0.84 | 0.000663194 | 4.66 | -1.93 |
| mmu-miR-221-3p | Msrb2 | -0.84 | 0.000663612 | 2.18 | -2.49 |
| mmu-miR-425-5p | Ric3 | -0.84 | 0.000664292 | 1.93 | -2.30 |
| mmu-miR-29a-3p | Scn3b | -0.84 | 0.000664578 | 2.71 | -1.70 |
| mmu-let-7d-3p | Thbs1 | -0.84 | 0.000665103 | -6.28 | 2.52 |
| mmu-miR-340-5p | Kcnj16 | -0.84 | 0.00066607 | 9.01 | -4.54 |
| mmu-miR-210-5p | A430105I19Rik | -0.84 | 0.000667742 | -3.25 | 2.55 |
| mmu-miR-132-3p | Arhgap5 | -0.84 | 0.000671604 | 2.20 | -1.85 |
| mmu-miR-421-3p | Fmo5 | -0.84 | 0.000674163 | 3.10 | -1.61 |
| mmu-let-7b-5p | Bcat1 | -0.84 | 0.00067515 | -3.08 | 5.31 |
| mmu-miR-23b-3p | Tspyl4 | -0.84 | 0.000676806 | 2.62 | -2.33 |
| mmu-miR-421-3p | Gprasp1 | -0.84 | 0.000680931 | 3.10 | -2.36 |
| mmu-miR-143-3p | Dixdc1 | -0.84 | 0.000682554 | 2.79 | -1.73 |
| mmu-miR-148a-3p | Six4 | -0.84 | 0.000683612 | 2.94 | -1.65 |
| mmu-miR-429-3p | Scn3b | -0.84 | 0.00068491 | 3.03 | -1.70 |
| mmu-miR-27a-3p | Aff3 | -0.84 | 0.000689567 | 3.17 | -1.84 |
| mmu-miR-205-3p | Calm2 | -0.83 | 0.000692308 | 6.09 | -1.70 |
| mmu-miR-143-3p | Fgf1 | -0.83 | 0.000695638 | 2.79 | -1.67 |
| mmu-miR-429-3p | Mlf1 | -0.83 | 0.000695846 | 3.03 | -2.32 |
| mmu-miR-98-5p | Akap6 | -0.83 | 0.000696598 | 3.72 | -2.33 |
| mmu-miR-26a-5p | Ppil6 | -0.83 | 0.000698549 | 2.26 | -2.27 |
| mmu-miR-93-5p | St8sia2 | -0.83 | 0.000700715 | 2.30 | -1.66 |
| mmu-miR-210-5p | Ptafr | -0.83 | 0.000702854 | -3.25 | 4.38 |
| mmu-miR-19b-3p | Dcun1d1 | -0.83 | 0.000704536 | 3.59 | -1.64 |
| mmu-miR-200b-3p | Myh10 | -0.83 | 0.000706638 | 2.19 | -1.69 |
| mmu-miR-200b-3p | Chn2 | -0.83 | 0.000706917 | 2.19 | -1.90 |
| mmu-miR-328-3p | Ptafr | -0.83 | 0.000707127 | -5.59 | 4.38 |
| mmu-miR-877-3p | Ccl3 | -0.83 | 0.000709725 | -4.18 | 3.93 |
| mmu-miR-877-3p | Ccl3 | -0.83 | 0.000709725 | -4.18 | 3.93 |
| mmu-miR-877-3p | Ccl3 | -0.83 | 0.000709725 | -4.18 | 3.93 |
| mmu-miR-141-3p | Foxp2 | -0.83 | 0.000715761 | 4.66 | -2.72 |
| mmu-miR-185-5p | Padi2 | -0.83 | 0.000716655 | 2.50 | -2.11 |
| mmu-let-7g-5p | Foxa2 | -0.83 | 0.000717524 | 2.05 | -2.09 |
| mmu-miR-23a-5p | Plaur | -0.83 | 0.000718486 | -2.43 | 2.60 |
| mmu-miR-19b-3p | Acsl3 | -0.83 | 0.00071853 | 3.59 | -1.65 |
| mmu-miR-148a-3p | Sms | -0.83 | 0.00072096 | 2.94 | -2.09 |
| mmu-let-7i-5p | Dnah7b | -0.83 | 0.000722356 | 1.79 | -1.97 |
| mmu-miR-149-3p | Ltb4r1 | -0.83 | 0.000725269 | -4.06 | 2.94 |
| mmu-miR-106b-5p | Stk33 | -0.83 | 0.000726256 | 4.02 | -2.78 |
| mmu-miR-30b-5p | 1190002N15Rik | -0.83 | 0.000738348 | 2.59 | -1.84 |
| mmu-miR-26b-5p | Ppil6 | -0.83 | 0.00074075 | 3.80 | -2.27 |
| mmu-miR-146a-5p | Dcn | -0.83 | 0.00074221 | 4.19 | -4.28 |
| mmu-miR-24-3p | Bmpr1b | -0.83 | 0.000743112 | 1.73 | -2.84 |
| mmu-miR-375-3p | Specc1 | -0.83 | 0.000743657 | -2.94 | 1.91 |
| mmu-miR-17-5p | Pkia | -0.83 | 0.000744532 | 3.83 | -2.03 |
| mmu-miR-181c-5p | Zbtb33 | -0.83 | 0.000746621 | 3.05 | -1.68 |
| mmu-miR-143-3p | Morn3 | -0.83 | 0.000747843 | 2.79 | -2.84 |
| mmu-miR-218-5p | 4931406C07Rik | -0.83 | 0.000749135 | 3.13 | -2.42 |
| mmu-miR-23b-3p | Pkia | -0.83 | 0.00075003 | 2.62 | -2.03 |
| mmu-miR-375-3p | H2-Q10 | -0.83 | 0.000751765 | -2.94 | 3.66 |
| mmu-miR-141-3p | Igf2bp2 | -0.83 | 0.000752773 | 4.66 | -2.19 |
| mmu-miR-98-5p | Pon1 | -0.83 | 0.000756156 | 3.72 | -3.35 |
| mmu-miR-30a-5p | Rab4a | -0.83 | 0.000757644 | 2.87 | -1.73 |
| mmu-miR-155-5p | Myb | -0.83 | 0.00076041 | 1.93 | -1.98 |
| mmu-miR-17-5p | Slc4a5 | -0.83 | 0.000762584 | 3.83 | -3.03 |
| mmu-miR-17-5p | Rnf128 | -0.83 | 0.000762973 | 3.83 | -1.91 |
| mmu-miR-30c-5p | Slc38a1 | -0.83 | 0.000763501 | 2.26 | -3.08 |
| mmu-miR-429-3p | Gprasp1 | -0.83 | 0.000767282 | 3.03 | -2.36 |
| mmu-miR-185-3p | Hspa1b | -0.83 | 0.000770007 | -3.01 | 2.24 |
| mmu-miR-92b-5p | Slc7a11 | -0.83 | 0.000770188 | -6.76 | 1.80 |
| mmu-let-7f-5p | Col15a1 | -0.83 | 0.000770783 | 3.20 | -2.31 |
| mmu-miR-29a-3p | Gpx7 | -0.83 | 0.000771526 | 2.71 | -2.02 |
| mmu-let-7b-5p | Sfn | -0.83 | 0.000771976 | -3.08 | 1.63 |
| mmu-let-7i-5p | Dync2li1 | -0.83 | 0.000776017 | 1.79 | -1.85 |
| mmu-miR-205-3p | Cyp4a12a | -0.83 | 0.000777294 | 6.09 | -3.14 |
| mmu-miR-200b-3p | Bche | -0.83 | 0.000783922 | 2.19 | -2.78 |
| mmu-let-7f-5p | Gpx7 | -0.83 | 0.000786383 | 3.20 | -2.02 |
| mmu-miR-218-5p | Kifap3 | -0.83 | 0.00078894 | 3.13 | -1.75 |
| mmu-miR-429-3p | Myb | -0.83 | 0.000791487 | 3.03 | -1.98 |
| mmu-miR-92b-3p | Hmha1 | -0.83 | 0.000792434 | -4.78 | 2.54 |
| mmu-miR-15a-5p | Arhgap5 | -0.83 | 0.000792694 | 2.29 | -1.85 |
| mmu-let-7i-5p | Pkia | -0.83 | 0.00079633 | 1.79 | -2.03 |
| mmu-miR-182-5p | Foxp2 | -0.83 | 0.000796687 | 3.32 | -2.72 |
| mmu-miR-181a-5p | Socs3 | -0.83 | 0.000799264 | -3.01 | 2.61 |
| mmu-miR-182-5p | Sez6l2 | -0.83 | 0.00080577 | 3.32 | -3.64 |
| mmu-miR-30e-5p | Rab4a | -0.83 | 0.000807294 | 3.60 | -1.73 |
| mmu-let-7b-5p | C130026I21Rik | -0.83 | 0.000808069 | -3.08 | 4.65 |
| mmu-miR-26a-5p | Myh10 | -0.83 | 0.000808227 | 2.26 | -1.69 |
| mmu-miR-92b-5p | Fam20c | -0.83 | 0.000815129 | -6.76 | 2.32 |
| mmu-miR-34a-5p | Akap6 | -0.83 | 0.000815399 | 3.60 | -2.33 |
| mmu-miR-181a-5p | Tnfrsf11b | -0.83 | 0.000816117 | -3.01 | 2.53 |
| mmu-let-7d-3p | Dsg3 | -0.83 | 0.000817353 | -6.28 | 1.91 |
| mmu-miR-10a-5p | Rora | -0.83 | 0.000818639 | 2.87 | -2.00 |
| mmu-miR-30e-5p | 1190002N15Rik | -0.83 | 0.000819328 | 3.60 | -1.84 |
| mmu-miR-22-3p | 2410004P03Rik | -0.83 | 0.000821518 | 2.95 | -2.16 |
| mmu-miR-181c-5p | Rora | -0.83 | 0.000822214 | 3.05 | -2.00 |
| mmu-miR-16-5p | Calm2 | -0.83 | 0.000823269 | 1.77 | -1.70 |
| mmu-miR-326-3p | Nbeal2 | -0.83 | 0.000825806 | -1.76 | 2.56 |
| mmu-miR-708-5p | Il13ra1 | -0.83 | 0.000825831 | 2.00 | -1.76 |
| mmu-let-7d-3p | Ptafr | -0.83 | 0.000826193 | -6.28 | 4.38 |
| mmu-miR-34a-5p | Tbc1d30 | -0.83 | 0.00082654 | 3.60 | -2.52 |
| mmu-miR-30b-5p | Lclat1 | -0.83 | 0.00082782 | 2.59 | -1.84 |
| mmu-miR-25-5p | Atg9b | -0.83 | 0.000830772 | -2.35 | 4.72 |
| mmu-miR-23b-3p | Six4 | -0.83 | 0.000831098 | 2.62 | -1.65 |
| mmu-miR-421-3p | Aff3 | -0.83 | 0.000847183 | 3.10 | -1.84 |
| mmu-miR-203-3p | Elmod1 | -0.83 | 0.000847636 | 4.73 | -1.66 |
| mmu-miR-185-5p | Aldoc | -0.83 | 0.000848846 | 2.50 | -2.23 |
| mmu-miR-200a-3p | Ppt1 | -0.83 | 0.000849706 | 2.61 | -1.73 |
| mmu-miR-203-3p | Tigd2 | -0.83 | 0.00085244 | 4.73 | -1.67 |
| mmu-miR-26b-5p | Myh10 | -0.83 | 0.000856345 | 3.80 | -1.69 |
| mmu-miR-218-5p | Cntnap2 | -0.83 | 0.000856652 | 3.13 | -2.56 |
| mmu-miR-148a-3p | Slc25a23 | -0.83 | 0.000859191 | 2.94 | -2.24 |
| mmu-miR-221-3p | Foxp2 | -0.83 | 0.000864753 | 2.18 | -2.72 |
| mmu-miR-221-3p | 1700007G11Rik | -0.83 | 0.000865292 | 2.18 | -1.74 |
| mmu-miR-92b-3p | Zfp276 | -0.83 | 0.000867498 | -4.78 | 1.74 |
| mmu-miR-149-3p | Ccl3 | -0.83 | 0.000867754 | -4.06 | 3.93 |
| mmu-miR-326-3p | Mfi2 | -0.83 | 0.000869285 | -1.76 | 2.43 |
| mmu-miR-23a-3p | Col6a1 | -0.83 | 0.000869482 | 3.01 | -2.27 |
| mmu-miR-23a-5p | Ptk2b | -0.83 | 0.000872707 | -2.43 | 1.83 |
| mmu-miR-421-3p | Tigd2 | -0.83 | 0.000872969 | 3.10 | -1.67 |
| mmu-miR-20a-5p | Zbtb33 | -0.83 | 0.000873593 | 3.70 | -1.68 |
| mmu-miR-34a-5p | Kitl | -0.83 | 0.000874132 | 3.60 | -2.33 |
| mmu-miR-16-5p | Cdh26 | -0.83 | 0.000874887 | 1.77 | -2.39 |
| mmu-miR-200b-3p | Lipf | -0.83 | 0.000877939 | 2.19 | -6.24 |
| mmu-miR-106b-5p | Slc4a5 | -0.82 | 0.000888865 | 4.02 | -3.03 |
| mmu-miR-340-5p | Fam69a | -0.82 | 0.00088897 | 9.01 | -2.33 |
| mmu-miR-146a-5p | Scn3b | -0.82 | 0.000891832 | 4.19 | -1.70 |
| mmu-let-7i-5p | Igf2bp2 | -0.82 | 0.000893376 | 1.79 | -2.19 |
| mmu-miR-23a-3p | Ulk4 | -0.82 | 0.000898399 | 3.01 | -1.85 |
| mmu-miR-877-3p | Nlrp3 | -0.82 | 0.000903675 | -4.18 | 3.80 |
| mmu-miR-19b-3p | Pcca | -0.82 | 0.000905369 | 3.59 | -1.94 |
| mmu-miR-106b-5p | Tmem17 | -0.82 | 0.000906491 | 4.02 | -1.99 |
| mmu-miR-27a-3p | Sec14l3 | -0.82 | 0.000908324 | 3.17 | -2.04 |
| mmu-miR-185-5p | Igfbp5 | -0.82 | 0.000911836 | 2.50 | -1.94 |
| mmu-miR-30a-5p | Myh10 | -0.82 | 0.000912145 | 2.87 | -1.69 |
| mmu-miR-106b-5p | Ttc26 | -0.82 | 0.000912486 | 4.02 | -1.81 |
| mmu-miR-185-3p | Cpne2 | -0.82 | 0.000912558 | -3.01 | 2.13 |
| mmu-miR-16-5p | Six4 | -0.82 | 0.00091545 | 1.77 | -1.65 |
| mmu-miR-200b-3p | Dtna | -0.82 | 0.000916017 | 2.19 | -1.62 |
| mmu-let-7b-5p | Edn1 | -0.82 | 0.000919875 | -3.08 | 2.51 |
| mmu-miR-182-5p | Acot1 | -0.82 | 0.000921729 | 3.32 | -2.12 |
| mmu-miR-182-5p | Acot1 | -0.82 | 0.000921729 | 3.32 | -2.12 |
| mmu-miR-218-5p | Rhoq | -0.82 | 0.000923733 | 3.13 | -1.59 |
| mmu-miR-182-5p | Cadm1 | -0.82 | 0.000926891 | 3.32 | -1.72 |
| mmu-miR-149-3p | Lrg1 | -0.82 | 0.000927064 | -4.06 | 2.65 |
| mmu-miR-193b-3p | Trim15 | -0.82 | 0.000929681 | -2.01 | 2.14 |
| mmu-miR-155-5p | Elmod1 | -0.82 | 0.000930279 | 1.93 | -1.66 |
| mmu-miR-23a-5p | Dusp5 | -0.82 | 0.000933821 | -2.43 | 1.63 |
| mmu-miR-504-5p | Pde1b | -0.82 | 0.000934897 | -4.12 | 1.80 |
| mmu-miR-218-5p | Slc38a1 | -0.82 | 0.000935299 | 3.13 | -3.08 |
| mmu-miR-15a-5p | Mlf1 | -0.82 | 0.000936238 | 2.29 | -2.32 |
| mmu-miR-23a-3p | Ptar1 | -0.82 | 0.000939869 | 3.01 | -3.27 |
| mmu-let-7f-5p | Col1a2 | -0.82 | 0.000939986 | 3.20 | -2.33 |
| mmu-miR-30e-5p | Lclat1 | -0.82 | 0.000942927 | 3.60 | -1.84 |
| mmu-miR-30a-5p | Dynlrb2 | -0.82 | 0.000950659 | 2.87 | -2.96 |
| mmu-miR-30c-5p | Abi3bp | -0.82 | 0.000951105 | 2.26 | -3.56 |
| mmu-miR-218-5p | Smtnl2 | -0.82 | 0.000954182 | 3.13 | -2.05 |
| mmu-miR-221-3p | 1700026D08Rik | -0.82 | 0.000957982 | 2.18 | -2.36 |
| mmu-miR-744-5p | Fmnl1 | -0.82 | 0.000964136 | -3.34 | 2.24 |
| mmu-miR-429-3p | Tmem17 | -0.82 | 0.000967204 | 3.03 | -1.99 |
| mmu-miR-27a-3p | Dtna | -0.82 | 0.000979752 | 3.17 | -1.62 |
| mmu-miR-155-5p | Aldh1a1 | -0.82 | 0.000985806 | 1.93 | -1.70 |
| mmu-miR-23b-3p | Slc4a8 | -0.82 | 0.000988207 | 2.62 | -1.64 |
| mmu-miR-877-5p | Padi4 | -0.82 | 0.000991157 | -4.79 | 1.72 |
| mmu-miR-221-3p | Igf2bp2 | -0.82 | 0.00099258 | 2.18 | -2.19 |
| mmu-miR-744-5p | Sfn | -0.82 | 0.000993524 | -3.34 | 1.63 |
| mmu-miR-326-3p | Ptprs | -0.82 | 0.001001716 | -1.76 | 1.75 |
| mmu-miR-203-3p | Sox5 | -0.82 | 0.001004633 | 4.73 | -2.90 |
| mmu-miR-181c-5p | Myh10 | -0.82 | 0.001009021 | 3.05 | -1.69 |
| mmu-miR-182-5p | Ptprg | -0.82 | 0.00100921 | 3.32 | -1.60 |
| mmu-miR-744-5p | Pik3ap1 | -0.82 | 0.001009951 | -3.34 | 2.92 |
| mmu-let-7b-5p | Lcp2 | -0.82 | 0.001010487 | -3.08 | 2.21 |
| mmu-miR-34a-5p | Atp1a2 | -0.82 | 0.001010788 | 3.60 | -4.08 |
| mmu-miR-25-5p | Dusp5 | -0.82 | 0.001010802 | -2.35 | 1.63 |
| mmu-miR-24-3p | Dnah9 | -0.82 | 0.001013832 | 1.73 | -2.00 |
| mmu-miR-223-3p | Myh10 | -0.82 | 0.001017622 | 9.15 | -1.69 |
| mmu-miR-421-3p | Arl4a | -0.82 | 0.001021239 | 3.10 | -1.95 |
| mmu-miR-200b-3p | Elmod1 | -0.82 | 0.001021987 | 2.19 | -1.66 |
| mmu-miR-146a-5p | B3galt2 | -0.82 | 0.001025281 | 4.19 | -1.66 |
| mmu-miR-143-3p | Six4 | -0.82 | 0.001026815 | 2.79 | -1.65 |
| mmu-miR-200a-3p | Ptprg | -0.82 | 0.001035504 | 2.61 | -1.60 |
| mmu-miR-98-5p | Slc25a23 | -0.82 | 0.001035989 | 3.72 | -2.24 |
| mmu-miR-760-3p | Socs1 | -0.82 | 0.001037543 | -7.15 | 1.90 |
| mmu-miR-148a-3p | Pxmp2 | -0.82 | 0.001037613 | 2.94 | -3.43 |
| mmu-miR-128-3p | Adcy2 | -0.82 | 0.001038626 | 2.80 | -2.55 |
| mmu-miR-340-5p | Myh10 | -0.82 | 0.001038671 | 9.01 | -1.69 |
| mmu-let-7g-5p | Gpx7 | -0.82 | 0.001042445 | 2.05 | -2.02 |
| mmu-miR-19b-3p | Kit | -0.82 | 0.001042715 | 3.59 | -1.91 |
| mmu-miR-135b-5p | Rabgap1l | -0.82 | 0.001044546 | 4.89 | -2.04 |
| mmu-miR-155-5p | Rora | -0.82 | 0.00104693 | 1.93 | -2.00 |
| mmu-miR-29a-3p | Kcnrg | -0.82 | 0.001050215 | 2.71 | -2.07 |
| mmu-miR-30b-5p | Myh10 | -0.82 | 0.001050708 | 2.59 | -1.69 |
| mmu-miR-132-3p | Pfn2 | -0.82 | 0.001051089 | 2.20 | -1.75 |
| mmu-miR-92b-5p | Sox4 | -0.82 | 0.001052309 | -6.76 | 1.76 |
| mmu-miR-218-5p | Cd34 | -0.82 | 0.001052738 | 3.13 | -3.73 |
| mmu-miR-15a-5p | Tbc1d19 | -0.82 | 0.00105365 | 2.29 | -2.63 |
| mmu-miR-20a-5p | Dnah6 | -0.82 | 0.001057767 | 3.70 | -1.93 |
| mmu-miR-30b-5p | Rab4a | -0.82 | 0.001065734 | 2.59 | -1.73 |
| mmu-miR-200a-3p | Mdm1 | -0.82 | 0.001066174 | 2.61 | -1.68 |
| mmu-miR-218-5p | Mlf1 | -0.82 | 0.00107538 | 3.13 | -2.32 |
| mmu-miR-200a-3p | Plekhb1 | -0.82 | 0.001077188 | 2.61 | -1.93 |
| mmu-miR-429-3p | Cadm1 | -0.82 | 0.001079171 | 3.03 | -1.72 |
| mmu-miR-27b-3p | Sema3b | -0.82 | 0.001082641 | 2.45 | -1.61 |
| mmu-let-7d-3p | Pik3ap1 | -0.82 | 0.001083278 | -6.28 | 2.92 |
| mmu-let-7b-5p | Thbs1 | -0.82 | 0.00108981 | -3.08 | 2.52 |
| mmu-miR-27b-3p | Dync2li1 | -0.82 | 0.001095195 | 2.45 | -1.85 |
| mmu-miR-106b-5p | B3galt2 | -0.82 | 0.001101828 | 4.02 | -1.66 |
| mmu-miR-218-5p | Arhgap5 | -0.82 | 0.001106499 | 3.13 | -1.85 |
| mmu-miR-23a-3p | Dtna | -0.82 | 0.001106692 | 3.01 | -1.62 |
| mmu-miR-27b-3p | Smoc2 | -0.82 | 0.001112046 | 2.45 | -2.15 |
| mmu-miR-17-5p | St8sia2 | -0.82 | 0.001112946 | 3.83 | -1.66 |
| mmu-miR-30e-3p | Slc25a34 | -0.82 | 0.001113145 | 4.97 | -1.68 |
| mmu-miR-19b-3p | Sar1b | -0.82 | 0.001117598 | 3.59 | -1.65 |
| mmu-miR-218-5p | Rora | -0.81 | 0.001127721 | 3.13 | -2.00 |
| mmu-let-7b-5p | Ccl6 | -0.81 | 0.001132263 | -3.08 | 2.24 |
| mmu-miR-29c-3p | Ttc30a1 | -0.81 | 0.001140661 | 5.21 | -2.40 |
| mmu-miR-340-5p | Dtna | -0.81 | 0.00114078 | 9.01 | -1.62 |
| mmu-miR-25-3p | Fstl1 | -0.81 | 0.001145737 | 2.56 | -2.02 |
| mmu-miR-340-5p | Bbox1 | -0.81 | 0.001145787 | 9.01 | -2.18 |
| mmu-miR-132-3p | Stk33 | -0.81 | 0.001151898 | 2.20 | -2.78 |
| mmu-let-7g-5p | Col15a1 | -0.81 | 0.001152723 | 2.05 | -2.31 |
| mmu-let-7f-5p | Foxp2 | -0.81 | 0.001154086 | 3.20 | -2.72 |
| mmu-miR-203-3p | Akap6 | -0.81 | 0.001154696 | 4.73 | -2.33 |
| mmu-miR-30b-5p | Htra3 | -0.81 | 0.001162278 | 2.59 | -1.92 |
| mmu-miR-410-3p | Arhgap5 | -0.81 | 0.001166013 | 3.62 | -1.85 |
| mmu-miR-24-3p | Armc4 | -0.81 | 0.001166782 | 1.73 | -3.02 |
| mmu-miR-23b-3p | Col6a1 | -0.81 | 0.001168095 | 2.62 | -2.27 |
| mmu-miR-141-3p | Scara5 | -0.81 | 0.001168865 | 4.66 | -2.63 |
| mmu-miR-128-3p | Myh10 | -0.81 | 0.00116889 | 2.80 | -1.69 |
| mmu-miR-182-5p | Cfh | -0.81 | 0.001170811 | 3.32 | -3.58 |
| mmu-miR-223-3p | Ccdc60 | -0.81 | 0.001174347 | 9.15 | -1.61 |
| mmu-miR-340-5p | Ppil6 | -0.81 | 0.001181638 | 9.01 | -2.27 |
| mmu-miR-183-3p | Ppic | -0.81 | 0.001182124 | 4.74 | -2.39 |
| mmu-miR-200b-3p | Tcp11 | -0.81 | 0.001182301 | 2.19 | -1.73 |
| mmu-let-7i-5p | Col4a6 | -0.81 | 0.001182717 | 1.79 | -1.94 |
| mmu-miR-429-3p | Lipf | -0.81 | 0.001189016 | 3.03 | -6.24 |
| mmu-miR-29a-3p | Dixdc1 | -0.81 | 0.001192453 | 2.71 | -1.73 |
| mmu-miR-130a-3p | Igfbp5 | -0.81 | 0.001193699 | 2.29 | -1.94 |
| mmu-miR-16-5p | Stk33 | -0.81 | 0.001196167 | 1.77 | -2.78 |
| mmu-miR-23a-3p | Slc4a8 | -0.81 | 0.001200867 | 3.01 | -1.64 |
| mmu-miR-16-5p | Dixdc1 | -0.81 | 0.001205461 | 1.77 | -1.73 |
| mmu-miR-200a-3p | Msrb2 | -0.81 | 0.001208064 | 2.61 | -2.49 |
| mmu-miR-375-3p | Gabbr1 | -0.81 | 0.001208391 | -2.94 | 2.09 |
| mmu-miR-223-3p | Cyp4v3 | -0.81 | 0.001210807 | 9.15 | -1.71 |
| mmu-let-7b-5p | S100a8 | -0.81 | 0.001210941 | -3.08 | 2.52 |
| mmu-miR-218-5p | Ugt8a | -0.81 | 0.001212687 | 3.13 | -4.94 |
| mmu-miR-132-3p | Col4a6 | -0.81 | 0.001215275 | 2.20 | -1.94 |
| mmu-miR-26b-5p | Tigd2 | -0.81 | 0.001219645 | 3.80 | -1.67 |
| mmu-let-7d-3p | Gmip | -0.81 | 0.00122055 | -6.28 | 2.22 |
| mmu-miR-877-3p | Slurp1 | -0.81 | 0.001226079 | -4.18 | 1.83 |
| mmu-miR-203-3p | Rpgrip1l | -0.81 | 0.00123396 | 4.73 | -1.64 |
| mmu-miR-223-3p | Slc27a2 | -0.81 | 0.001238212 | 9.15 | -3.23 |
| mmu-miR-26b-5p | Ccdc60 | -0.81 | 0.001241275 | 3.80 | -1.61 |
| mmu-miR-130a-3p | Lclat1 | -0.81 | 0.001246353 | 2.29 | -1.84 |
| mmu-miR-26a-5p | Meig1 | -0.81 | 0.001256513 | 2.26 | -2.50 |
| mmu-miR-421-3p | Ppt1 | -0.81 | 0.001256612 | 3.10 | -1.73 |
| mmu-miR-27b-3p | Pkia | -0.81 | 0.001258603 | 2.45 | -2.03 |
| mmu-miR-16-5p | Mlf1 | -0.81 | 0.001262262 | 1.77 | -2.32 |
| mmu-miR-182-5p | Slc23a2 | -0.81 | 0.001269164 | 3.32 | -1.79 |
| mmu-miR-132-3p | Ric3 | -0.81 | 0.001275065 | 2.20 | -2.30 |
| mmu-miR-181c-5p | Ccdc60 | -0.81 | 0.001277164 | 3.05 | -1.61 |
| mmu-miR-92a-3p | Pusl1 | -0.81 | 0.001277815 | -2.41 | 1.89 |
| mmu-miR-182-5p | Dixdc1 | -0.81 | 0.00128435 | 3.32 | -1.73 |
| mmu-miR-20a-5p | Rragd | -0.81 | 0.001288081 | 3.70 | -2.21 |
| mmu-let-7g-5p | Akap6 | -0.81 | 0.001292366 | 2.05 | -2.33 |
| mmu-miR-23b-3p | Bmpr1b | -0.81 | 0.001297059 | 2.62 | -2.84 |
| mmu-miR-328-3p | Sema6b | -0.81 | 0.001301759 | -5.59 | 2.27 |
| mmu-let-7d-3p | Nat8l | -0.81 | 0.001303392 | -6.28 | 2.43 |
| mmu-miR-205-3p | Serpinf1 | -0.81 | 0.001309012 | 6.09 | -4.36 |
| mmu-miR-143-3p | Chek2 | -0.81 | 0.001312391 | 2.79 | -1.80 |
| mmu-miR-130a-3p | Casd1 | -0.81 | 0.001313378 | 2.29 | -2.06 |
| mmu-miR-132-3p | Dpyd | -0.81 | 0.001318104 | 2.20 | -1.87 |
| mmu-miR-29a-3p | Lgi2 | -0.81 | 0.001319831 | 2.71 | -2.32 |
| mmu-miR-182-5p | Ppil6 | -0.81 | 0.001327506 | 3.32 | -2.27 |
| mmu-miR-30e-5p | Dynlrb2 | -0.81 | 0.001333391 | 3.60 | -2.96 |
| mmu-miR-130a-3p | Fam13a | -0.81 | 0.001342538 | 2.29 | -3.21 |
| mmu-miR-23a-5p | Slc16a6 | -0.81 | 0.001346786 | -2.43 | 3.02 |
| mmu-miR-22-3p | Akap6 | -0.81 | 0.001353485 | 2.95 | -2.33 |
| mmu-miR-429-3p | Dtna | -0.81 | 0.001362325 | 3.03 | -1.62 |
| mmu-miR-132-3p | Dynlrb2 | -0.81 | 0.001363884 | 2.20 | -2.96 |
| mmu-let-7b-5p | Nat8l | -0.81 | 0.001367219 | -3.08 | 2.43 |
| mmu-miR-25-3p | Dpyd | -0.81 | 0.001368422 | 2.56 | -1.87 |
| mmu-miR-223-3p | Ttc26 | -0.81 | 0.001369687 | 9.15 | -1.81 |
| mmu-miR-16-5p | Eya1 | -0.81 | 0.001370457 | 1.77 | -1.69 |
| mmu-miR-429-3p | Elmod1 | -0.81 | 0.00137163 | 3.03 | -1.66 |
| mmu-miR-421-3p | Tbc1d30 | -0.81 | 0.001377939 | 3.10 | -2.52 |
| mmu-miR-19b-3p | Prkaa2 | -0.81 | 0.001379068 | 3.59 | -2.21 |
| mmu-miR-19b-3p | Rora | -0.81 | 0.001379434 | 3.59 | -2.00 |
| mmu-miR-203-3p | Casc1 | -0.81 | 0.001384624 | 4.73 | -1.66 |
| mmu-miR-205-3p | Cldn8 | -0.81 | 0.00138728 | 6.09 | -2.86 |
| mmu-miR-130a-3p | Adcy2 | -0.81 | 0.001392401 | 2.29 | -2.55 |
| mmu-miR-744-5p | Trim15 | -0.81 | 0.001393422 | -3.34 | 2.14 |
| mmu-miR-744-5p | Mfi2 | -0.81 | 0.001394211 | -3.34 | 2.43 |
| mmu-miR-132-3p | Kitl | -0.81 | 0.001394642 | 2.20 | -2.33 |
| mmu-miR-15a-5p | Ghr | -0.81 | 0.001395949 | 2.29 | -2.23 |
| mmu-miR-210-3p | Tigd2 | -0.81 | 0.00139731 | 1.86 | -1.67 |
| mmu-miR-221-3p | Bche | -0.81 | 0.001398367 | 2.18 | -2.78 |
| mmu-let-7f-5p | Akap6 | -0.80 | 0.001400074 | 3.20 | -2.33 |
| mmu-miR-24-3p | 6820408C15Rik | -0.80 | 0.001400312 | 1.73 | -2.61 |
| mmu-miR-132-3p | Pam | -0.80 | 0.001401938 | 2.20 | -1.83 |
| mmu-miR-30a-5p | Slc25a34 | -0.80 | 0.001418017 | 2.87 | -1.68 |
| mmu-miR-30c-5p | Lclat1 | -0.80 | 0.001422946 | 2.26 | -1.84 |
| mmu-miR-130a-3p | Rwdd3 | -0.80 | 0.001429043 | 2.29 | -2.36 |
| mmu-miR-200a-3p | Scara5 | -0.80 | 0.001431971 | 2.61 | -2.63 |
| mmu-miR-141-3p | Mdm1 | -0.80 | 0.001433538 | 4.66 | -1.68 |
| mmu-miR-30a-5p | Fancl | -0.80 | 0.001435847 | 2.87 | -2.07 |
| mmu-miR-98-5p | Igf2bp2 | -0.80 | 0.001438659 | 3.72 | -2.19 |
| mmu-miR-92b-3p | Pik3ap1 | -0.80 | 0.0014409 | -4.78 | 2.92 |
| mmu-miR-30b-5p | Fancl | -0.80 | 0.00144991 | 2.59 | -2.07 |
| mmu-miR-106b-5p | Wdr19 | -0.80 | 0.00145063 | 4.02 | -1.81 |
| mmu-miR-30b-5p | Ugt8a | -0.80 | 0.001457166 | 2.59 | -4.94 |
| mmu-miR-26b-5p | Intu | -0.80 | 0.00145999 | 3.80 | -1.73 |
| mmu-miR-92a-3p | Fam20c | -0.80 | 0.001462699 | -2.41 | 2.32 |
| mmu-miR-221-3p | Dnah7b | -0.80 | 0.001465701 | 2.18 | -1.97 |
| mmu-miR-17-5p | Dnah6 | -0.80 | 0.001471623 | 3.83 | -1.93 |
| mmu-miR-30c-5p | Pfn2 | -0.80 | 0.001474605 | 2.26 | -1.75 |
| mmu-miR-132-3p | Nov | -0.80 | 0.001476277 | 2.20 | -2.34 |
| mmu-miR-193b-3p | Rassf1 | -0.80 | 0.001480472 | -2.01 | 1.62 |
| mmu-miR-410-3p | Prkar2b | -0.80 | 0.00148089 | 3.62 | -2.31 |
| mmu-miR-26a-5p | Tspyl4 | -0.80 | 0.001483854 | 2.26 | -2.33 |
| mmu-miR-425-5p | Mdm1 | -0.80 | 0.001489217 | 1.93 | -1.68 |
| mmu-miR-200a-3p | Col15a1 | -0.80 | 0.001490686 | 2.61 | -2.31 |
| mmu-miR-27a-3p | Scara5 | -0.80 | 0.001492277 | 3.17 | -2.63 |
| mmu-miR-155-5p | Ptar1 | -0.80 | 0.001494257 | 1.93 | -3.27 |
| mmu-let-7b-5p | Padi4 | -0.80 | 0.001495804 | -3.08 | 1.72 |
| mmu-miR-16-5p | Kcnrg | -0.80 | 0.001498218 | 1.77 | -2.07 |
| mmu-let-7i-5p | Eln | -0.80 | 0.001501628 | 1.79 | -2.85 |
| mmu-miR-15a-5p | Stk33 | -0.80 | 0.001503663 | 2.29 | -2.78 |
| mmu-miR-504-5p | Trim15 | -0.80 | 0.00151191 | -4.12 | 2.14 |
| mmu-miR-16-5p | Tbc1d19 | -0.80 | 0.001521812 | 1.77 | -2.63 |
| mmu-miR-433-3p | Ptafr | -0.80 | 0.001525328 | -4.72 | 4.38 |
| mmu-miR-181a-5p | B3galt5 | -0.80 | 0.00152617 | -3.01 | 2.28 |
| mmu-miR-340-5p | Aff3 | -0.80 | 0.001527365 | 9.01 | -1.84 |
| mmu-miR-26b-5p | C1s1 | -0.80 | 0.001534335 | 3.80 | -2.40 |
| mmu-miR-223-3p | Pfn2 | -0.80 | 0.001536631 | 9.15 | -1.75 |
| mmu-let-7d-3p | Sfn | -0.80 | 0.001537472 | -6.28 | 1.63 |
| mmu-let-7g-5p | Foxp2 | -0.80 | 0.001548946 | 2.05 | -2.72 |
| mmu-miR-30b-5p | Pvrl3 | -0.80 | 0.001549916 | 2.59 | -4.33 |
| mmu-miR-183-3p | Acsm1 | -0.80 | 0.001551254 | 4.74 | -2.87 |
| mmu-miR-30b-5p | Manea | -0.80 | 0.001557376 | 2.59 | -2.60 |
| mmu-let-7f-5p | Ppil6 | -0.80 | 0.00155764 | 3.20 | -2.27 |
| mmu-miR-200b-3p | Slc23a2 | -0.80 | 0.001558866 | 2.19 | -1.79 |
| mmu-miR-19b-3p | Fbxo36 | -0.80 | 0.001560121 | 3.59 | -1.72 |
| mmu-miR-132-3p | Gmnn | -0.80 | 0.001563809 | 2.20 | -2.26 |
| mmu-miR-200b-3p | Dixdc1 | -0.80 | 0.001566618 | 2.19 | -1.73 |
| mmu-miR-30e-5p | Dtna | -0.80 | 0.001571733 | 3.60 | -1.62 |
| mmu-miR-200a-3p | Igf2bp2 | -0.80 | 0.00157327 | 2.61 | -2.19 |
| mmu-miR-221-3p | Sema3b | -0.80 | 0.001575511 | 2.18 | -1.61 |
| mmu-miR-328-3p | Nkd2 | -0.80 | 0.001584525 | -5.59 | 3.66 |
| mmu-miR-16-5p | Arhgap5 | -0.80 | 0.001592103 | 1.77 | -1.85 |
| mmu-miR-23a-5p | Arg1 | -0.80 | 0.001595116 | -2.43 | 2.48 |
| mmu-miR-93-5p | Pkia | -0.80 | 0.001598007 | 2.30 | -2.03 |
| mmu-miR-192-5p | Fcgbp | -0.80 | 0.001603624 | 5.95 | -2.56 |
| mmu-miR-433-3p | Msr1 | -0.80 | 0.001606563 | -4.72 | 3.60 |
| mmu-miR-181c-5p | Mme | -0.80 | 0.001607247 | 3.05 | -5.25 |
| mmu-miR-155-5p | Slc27a2 | -0.80 | 0.001614804 | 1.93 | -3.23 |
| mmu-miR-205-3p | Casd1 | -0.80 | 0.001621079 | 6.09 | -2.06 |
| mmu-miR-212-3p | Cdkn1c | -0.80 | 0.00162814 | 3.43 | -2.19 |
| mmu-miR-421-3p | Dcn | -0.80 | 0.001632763 | 3.10 | -4.28 |
| mmu-let-7g-5p | Col1a2 | -0.80 | 0.001632898 | 2.05 | -2.33 |
| mmu-miR-23a-3p | Intu | -0.80 | 0.001637301 | 3.01 | -1.73 |
| mmu-miR-200a-3p | Dixdc1 | -0.80 | 0.001650359 | 2.61 | -1.73 |
| mmu-miR-192-5p | Gna14 | -0.80 | 0.001654718 | 5.95 | -1.90 |
| mmu-miR-429-3p | Chn2 | -0.80 | 0.001657908 | 3.03 | -1.90 |
| mmu-miR-132-3p | Abhd3 | -0.80 | 0.001662972 | 2.20 | -2.19 |
| mmu-miR-27a-3p | Smoc2 | -0.80 | 0.001667279 | 3.17 | -2.15 |
| mmu-miR-26a-5p | C1s1 | -0.80 | 0.001668577 | 2.26 | -2.40 |
| mmu-miR-181a-5p | Slc7a11 | -0.80 | 0.001668628 | -3.01 | 1.80 |
| mmu-miR-205-3p | Cdh26 | -0.80 | 0.00167049 | 6.09 | -2.39 |
| mmu-miR-143-3p | Zfp474 | -0.80 | 0.001678398 | 2.79 | -2.17 |
| mmu-miR-23b-3p | Dnah9 | -0.80 | 0.001679178 | 2.62 | -2.00 |
| mmu-let-7b-5p | Apobec1 | -0.80 | 0.001680058 | -3.08 | 1.74 |
| mmu-miR-143-3p | Ccdc60 | -0.80 | 0.001685243 | 2.79 | -1.61 |
| mmu-miR-155-5p | Dynlrb2 | -0.80 | 0.001685832 | 1.93 | -2.96 |
| mmu-miR-26b-5p | Tspyl4 | -0.80 | 0.001686586 | 3.80 | -2.33 |
| mmu-miR-29c-3p | Mlf1 | -0.80 | 0.001690451 | 5.21 | -2.32 |
| mmu-miR-98-5p | Foxp2 | -0.80 | 0.00169261 | 3.72 | -2.72 |
| mmu-miR-16-5p | Ulk4 | -0.80 | 0.001699795 | 1.77 | -1.85 |
| mmu-miR-130a-3p | Myb | -0.80 | 0.0017059 | 2.29 | -1.98 |
| mmu-miR-15a-5p | Rora | -0.80 | 0.001707379 | 2.29 | -2.00 |
| mmu-miR-130a-3p | Capsl | -0.80 | 0.001710019 | 2.29 | -1.70 |
| mmu-let-7i-5p | Pm20d2 | -0.80 | 0.00171095 | 1.79 | -2.00 |
| mmu-miR-200a-3p | Elmod1 | -0.80 | 0.001717978 | 2.61 | -1.66 |
| mmu-let-7f-5p | Igf2bp2 | -0.80 | 0.001718178 | 3.20 | -2.19 |
| mmu-miR-30a-5p | Dtna | -0.80 | 0.001719043 | 2.87 | -1.62 |
| mmu-miR-203-3p | Slc16a7 | -0.79 | 0.001724638 | 4.73 | -3.51 |
| mmu-miR-30e-5p | Rgs22 | -0.79 | 0.001732594 | 3.60 | -2.80 |
| mmu-miR-92b-5p | Pusl1 | -0.79 | 0.001736982 | -6.76 | 1.89 |
| mmu-miR-31-5p | Cfh | -0.79 | 0.001739868 | 4.65 | -3.58 |
| mmu-miR-29a-3p | Hpgd | -0.79 | 0.001750497 | 2.71 | -2.36 |
| mmu-miR-93-5p | Slc4a5 | -0.79 | 0.001760296 | 2.30 | -3.03 |
| mmu-miR-223-3p | Sntn | -0.79 | 0.001763747 | 9.15 | -2.73 |
| mmu-miR-20a-5p | Capn13 | -0.79 | 0.001778477 | 3.70 | -3.68 |
| mmu-miR-185-3p | Csf2rb2 | -0.79 | 0.001786441 | -3.01 | 2.57 |
| mmu-miR-183-3p | Pfn2 | -0.79 | 0.001788156 | 4.74 | -1.75 |
| mmu-miR-98-5p | Pm20d2 | -0.79 | 0.001789136 | 3.72 | -2.00 |
| mmu-miR-429-3p | Aff3 | -0.79 | 0.001793641 | 3.03 | -1.84 |
| mmu-miR-16-5p | Rgs22 | -0.79 | 0.001798082 | 1.77 | -2.80 |
| mmu-miR-25-3p | Foxp2 | -0.79 | 0.001799407 | 2.56 | -2.72 |
| mmu-miR-29c-3p | Msh2 | -0.79 | 0.001802116 | 5.21 | -1.86 |
| mmu-miR-29c-3p | Scn3b | -0.79 | 0.001807524 | 5.21 | -1.70 |
| mmu-miR-210-5p | Rassf1 | -0.79 | 0.001814393 | -3.25 | 1.62 |
| mmu-miR-15a-5p | Arhgap18 | -0.79 | 0.001815737 | 2.29 | -1.77 |
| mmu-miR-200a-3p | Rwdd3 | -0.79 | 0.001819932 | 2.61 | -2.36 |
| mmu-miR-143-3p | Tacr1 | -0.79 | 0.00182122 | 2.79 | -5.28 |
| mmu-let-7f-5p | Ghr | -0.79 | 0.001823508 | 3.20 | -2.23 |
| mmu-miR-26b-5p | Gdpd2 | -0.79 | 0.001825059 | 3.80 | -1.78 |
| mmu-miR-30c-5p | Rora | -0.79 | 0.001826117 | 2.26 | -2.00 |
| mmu-miR-30e-5p | Slc23a2 | -0.79 | 0.001826136 | 3.60 | -1.79 |
| mmu-miR-340-5p | Kit | -0.79 | 0.001834569 | 9.01 | -1.91 |
| mmu-miR-223-3p | Kcnrg | -0.79 | 0.001834859 | 9.15 | -2.07 |
| mmu-miR-181c-5p | Dcn | -0.79 | 0.001837691 | 3.05 | -4.28 |
| mmu-miR-130a-3p | Pcca | -0.79 | 0.001841545 | 2.29 | -1.94 |
| mmu-miR-25-5p | Myo5a | -0.79 | 0.001848253 | -2.35 | 1.84 |
| mmu-miR-30c-5p | Enpp5 | -0.79 | 0.001856079 | 2.26 | -1.89 |
| mmu-miR-326-3p | Id1 | -0.79 | 0.001856082 | -1.76 | 1.65 |
| mmu-miR-26a-5p | Hpgd | -0.79 | 0.001861575 | 2.26 | -2.36 |
| mmu-miR-182-5p | 1700007G11Rik | -0.79 | 0.001868632 | 3.32 | -1.74 |
| mmu-miR-22-3p | Fbxo36 | -0.79 | 0.001869074 | 2.95 | -1.72 |
| mmu-let-7b-5p | Msr1 | -0.79 | 0.001871194 | -3.08 | 3.60 |
| mmu-miR-93-5p | Smoc2 | -0.79 | 0.001884724 | 2.30 | -2.15 |
| mmu-miR-433-3p | Stra6 | -0.79 | 0.001886527 | -4.72 | 2.33 |
| mmu-miR-16-5p | Kifap3 | -0.79 | 0.001889109 | 1.77 | -1.75 |
| mmu-miR-760-3p | B3galt5 | -0.79 | 0.001890769 | -7.15 | 2.28 |
| mmu-miR-29c-3p | Ric3 | -0.79 | 0.001893367 | 5.21 | -2.30 |
| mmu-miR-92a-3p | Hmha1 | -0.79 | 0.001894523 | -2.41 | 2.54 |
| mmu-miR-10a-5p | Six4 | -0.79 | 0.001902602 | 2.87 | -1.65 |
| mmu-miR-30b-5p | Slc25a34 | -0.79 | 0.001902845 | 2.59 | -1.68 |
| mmu-miR-135b-5p | Dram2 | -0.79 | 0.001903426 | 4.89 | -1.92 |
| mmu-miR-30a-5p | Aff3 | -0.79 | 0.001905478 | 2.87 | -1.84 |
| mmu-miR-15a-5p | Six4 | -0.79 | 0.001911762 | 2.29 | -1.65 |
| mmu-miR-98-5p | Ghr | -0.79 | 0.001913882 | 3.72 | -2.23 |
| mmu-miR-30c-5p | Fam13a | -0.79 | 0.001915622 | 2.26 | -3.21 |
| mmu-miR-429-3p | Slc23a2 | -0.79 | 0.001915796 | 3.03 | -1.79 |
| mmu-miR-34a-5p | Dixdc1 | -0.79 | 0.001919831 | 3.60 | -1.73 |
| mmu-miR-218-5p | Dcun1d1 | -0.79 | 0.001920901 | 3.13 | -1.64 |
| mmu-miR-181a-5p | Lama1 | -0.79 | 0.001922519 | -3.01 | 4.14 |
| mmu-miR-30c-5p | Slc23a2 | -0.79 | 0.001925234 | 2.26 | -1.79 |
| mmu-miR-29c-3p | Ppic | -0.79 | 0.001934121 | 5.21 | -2.39 |
| mmu-miR-200a-3p | Tmem17 | -0.79 | 0.001938923 | 2.61 | -1.99 |
| mmu-let-7d-3p | Osm | -0.79 | 0.001949936 | -6.28 | 4.34 |
| mmu-miR-15a-5p | Eya1 | -0.79 | 0.001956271 | 2.29 | -1.69 |
| mmu-miR-429-3p | Abhd3 | -0.79 | 0.001957737 | 3.03 | -2.19 |
| mmu-miR-92a-3p | Slx4 | -0.79 | 0.001959742 | -2.41 | 1.68 |
| mmu-miR-203-3p | Scara5 | -0.79 | 0.001965079 | 4.73 | -2.63 |
| mmu-miR-98-5p | Apoo | -0.79 | 0.001965114 | 3.72 | -3.38 |
| mmu-miR-30c-5p | 1190002N15Rik | -0.79 | 0.001973585 | 2.26 | -1.84 |
| mmu-miR-30e-3p | Actc1 | -0.79 | 0.001974672 | 4.97 | -2.17 |
| mmu-miR-26b-5p | Meig1 | -0.79 | 0.001980261 | 3.80 | -2.50 |
| mmu-let-7f-5p | 1700026D08Rik | -0.79 | 0.001981493 | 3.20 | -2.36 |
| mmu-miR-205-3p | Wnt5a | -0.79 | 0.001983994 | 6.09 | -2.52 |
| mmu-miR-130a-3p | Avil | -0.79 | 0.001984505 | 2.29 | -1.67 |
| mmu-miR-30c-5p | Ttc30a1 | -0.79 | 0.001988634 | 2.26 | -2.40 |
| mmu-miR-19b-3p | Rragd | -0.79 | 0.002016054 | 3.59 | -2.21 |
| mmu-miR-27b-3p | Scara5 | -0.79 | 0.002016393 | 2.45 | -2.63 |
| mmu-miR-200a-3p | Tspyl4 | -0.79 | 0.002018187 | 2.61 | -2.33 |
| mmu-miR-30a-5p | Scara5 | -0.79 | 0.002022464 | 2.87 | -2.63 |
| mmu-miR-340-5p | Slco4c1 | -0.79 | 0.00202338 | 9.01 | -2.76 |
| mmu-miR-27a-3p | St3gal6 | -0.79 | 0.002028433 | 3.17 | -2.31 |
| mmu-miR-92a-3p | Ripk3 | -0.79 | 0.002028804 | -2.41 | 1.70 |
| mmu-miR-22-3p | Sema3b | -0.79 | 0.002044964 | 2.95 | -1.61 |
| mmu-miR-30b-5p | Dtna | -0.79 | 0.002050708 | 2.59 | -1.62 |
| mmu-miR-30a-5p | C1s1 | -0.79 | 0.002051939 | 2.87 | -2.40 |
| mmu-miR-29a-3p | Efhc1 | -0.79 | 0.00205209 | 2.71 | -2.17 |
| mmu-let-7g-5p | Ppil6 | -0.79 | 0.002057687 | 2.05 | -2.27 |
| mmu-miR-205-3p | Atp2a3 | -0.79 | 0.002068629 | 6.09 | -3.03 |
| mmu-miR-93-5p | Dnah6 | -0.79 | 0.002069792 | 2.30 | -1.93 |
| mmu-miR-218-5p | Fam13a | -0.79 | 0.002070445 | 3.13 | -3.21 |
| mmu-miR-148a-3p | Wdr19 | -0.79 | 0.002076449 | 2.94 | -1.81 |
| mmu-miR-23a-3p | Tspyl4 | -0.79 | 0.002078174 | 3.01 | -2.33 |
| mmu-miR-203-3p | Bche | -0.79 | 0.002081716 | 4.73 | -2.78 |
| mmu-miR-504-5p | Ripk3 | -0.79 | 0.002081757 | -4.12 | 1.70 |
| mmu-miR-149-3p | Psca | -0.79 | 0.002085913 | -4.06 | 1.94 |
| mmu-miR-200b-3p | Dync2h1 | -0.78 | 0.00210805 | 2.19 | -1.72 |
| mmu-let-7f-5p | Gdpd2 | -0.78 | 0.002115502 | 3.20 | -1.78 |
| mmu-miR-223-3p | B4galt4 | -0.78 | 0.002121598 | 9.15 | -2.19 |
| mmu-miR-421-3p | 1700026D08Rik | -0.78 | 0.00212201 | 3.10 | -2.36 |
| mmu-miR-17-5p | Zbtb33 | -0.78 | 0.00212688 | 3.83 | -1.68 |
| mmu-miR-130a-3p | Arl4a | -0.78 | 0.002127537 | 2.29 | -1.95 |
| mmu-miR-30b-5p | Capn13 | -0.78 | 0.002128536 | 2.59 | -3.68 |
| mmu-miR-26a-5p | Intu | -0.78 | 0.002138128 | 2.26 | -1.73 |
| mmu-miR-34a-5p | Manea | -0.78 | 0.00213939 | 3.60 | -2.60 |
| mmu-miR-25-3p | Col4a6 | -0.78 | 0.002140192 | 2.56 | -1.94 |
| mmu-miR-32-5p | Adi1 | -0.78 | 0.002141636 | 4.39 | -1.73 |
| mmu-miR-193b-3p | Zc3h12a | -0.78 | 0.002143906 | -2.01 | 1.67 |
| mmu-miR-326-3p | 9430015G10Rik | -0.78 | 0.002144445 | -1.76 | 1.93 |
| mmu-miR-16-5p | Zbtb33 | -0.78 | 0.002144835 | 1.77 | -1.68 |
| mmu-miR-421-3p | Htra3 | -0.78 | 0.002150964 | 3.10 | -1.92 |
| mmu-miR-182-5p | Tmem67 | -0.78 | 0.002151073 | 3.32 | -2.19 |
| mmu-miR-19b-3p | Atp1a2 | -0.78 | 0.002152558 | 3.59 | -4.08 |
| mmu-miR-32-5p | Sdc2 | -0.78 | 0.002153155 | 4.39 | -2.29 |
| mmu-miR-205-3p | Ccdc121 | -0.78 | 0.002173308 | 6.09 | -1.71 |
| mmu-miR-141-3p | Slc16a7 | -0.78 | 0.00217554 | 4.66 | -3.51 |
| mmu-miR-155-5p | Arhgap18 | -0.78 | 0.002177819 | 1.93 | -1.77 |
| mmu-miR-26a-5p | Casc1 | -0.78 | 0.002179346 | 2.26 | -1.66 |
| mmu-let-7d-3p | Tnfrsf1b | -0.78 | 0.0021921 | -6.28 | 1.68 |
| mmu-miR-141-3p | Ptprg | -0.78 | 0.002204226 | 4.66 | -1.60 |
| mmu-miR-30e-5p | Tspyl4 | -0.78 | 0.002204994 | 3.60 | -2.33 |
| mmu-miR-34a-5p | Dnaic2 | -0.78 | 0.002212676 | 3.60 | -2.03 |
| mmu-miR-30b-5p | Scara5 | -0.78 | 0.002214081 | 2.59 | -2.63 |
| mmu-miR-326-3p | Gmip | -0.78 | 0.002219012 | -1.76 | 2.22 |
| mmu-let-7d-3p | S100a8 | -0.78 | 0.002227305 | -6.28 | 2.52 |
| mmu-miR-30e-5p | Scara5 | -0.78 | 0.002228598 | 3.60 | -2.63 |
| mmu-miR-93-5p | Rragd | -0.78 | 0.002231167 | 2.30 | -2.21 |
| mmu-miR-30b-5p | Rgs22 | -0.78 | 0.002235725 | 2.59 | -2.80 |
| mmu-miR-23a-3p | Bmpr1b | -0.78 | 0.002237276 | 3.01 | -2.84 |
| mmu-miR-31-5p | Dnaic1 | -0.78 | 0.002243927 | 4.65 | -1.71 |
| mmu-miR-221-3p | Pkia | -0.78 | 0.002247679 | 2.18 | -2.03 |
| mmu-miR-141-5p | Gpm6b | -0.78 | 0.002262839 | 1.68 | -1.96 |
| mmu-miR-15a-5p | Kcnrg | -0.78 | 0.002267629 | 2.29 | -2.07 |
| mmu-miR-15a-5p | Ppt1 | -0.78 | 0.002271903 | 2.29 | -1.73 |
| mmu-let-7f-5p | Pkia | -0.78 | 0.002275349 | 3.20 | -2.03 |
| mmu-miR-23a-3p | Six4 | -0.78 | 0.002278213 | 3.01 | -1.65 |
| mmu-miR-143-3p | Rpgr | -0.78 | 0.002284159 | 2.79 | -2.01 |
| mmu-miR-210-5p | B3galt5 | -0.78 | 0.002284685 | -3.25 | 2.28 |
| mmu-miR-203-3p | Dysf | -0.78 | 0.002285896 | 4.73 | -2.88 |
| mmu-miR-30e-5p | Six4 | -0.78 | 0.00229681 | 3.60 | -1.65 |
| mmu-miR-135b-5p | Msh2 | -0.78 | 0.00230046 | 4.89 | -1.86 |
| mmu-miR-132-3p | Smoc2 | -0.78 | 0.002300954 | 2.20 | -2.15 |
| mmu-miR-15a-5p | Dixdc1 | -0.78 | 0.002305058 | 2.29 | -1.73 |
| mmu-miR-30e-5p | Myh10 | -0.78 | 0.002306522 | 3.60 | -1.69 |
| mmu-miR-676-3p | Mafg | -0.78 | 0.002316514 | -4.64 | 1.84 |
| mmu-miR-146a-5p | Sox5 | -0.78 | 0.002319634 | 4.19 | -2.90 |
| mmu-miR-22-3p | Igfbp4 | -0.78 | 0.002334559 | 2.95 | -1.67 |
| mmu-miR-34a-5p | Smtnl2 | -0.78 | 0.002343925 | 3.60 | -2.05 |
| mmu-let-7d-3p | Lcp2 | -0.78 | 0.00235452 | -6.28 | 2.21 |
| mmu-miR-27b-3p | Rpgrip1l | -0.78 | 0.002355106 | 2.45 | -1.64 |
| mmu-miR-200a-3p | Slc16a7 | -0.78 | 0.002356354 | 2.61 | -3.51 |
| mmu-miR-183-3p | Dmbt1 | -0.78 | 0.002357834 | 4.74 | -3.59 |
| mmu-miR-421-3p | Intu | -0.78 | 0.002362081 | 3.10 | -1.73 |
| mmu-miR-98-5p | Pkia | -0.78 | 0.002370743 | 3.72 | -2.03 |
| mmu-miR-10a-5p | Spib | -0.78 | 0.002375253 | 2.87 | -2.22 |
| mmu-miR-30b-5p | Aff3 | -0.78 | 0.002379342 | 2.59 | -1.84 |
| mmu-miR-26a-5p | Pex12 | -0.78 | 0.002379824 | 2.26 | -1.77 |
| mmu-miR-193b-3p | Plau | -0.78 | 0.002385542 | -2.01 | 2.76 |
| mmu-miR-30c-5p | Dynlrb2 | -0.78 | 0.002386144 | 2.26 | -2.96 |
| mmu-miR-27b-3p | Atp1a2 | -0.78 | 0.002387133 | 2.45 | -4.08 |
| mmu-miR-340-5p | Tmem35 | -0.78 | 0.002387371 | 9.01 | -1.94 |
| mmu-miR-221-3p | 2310057J18Rik | -0.78 | 0.002394406 | 2.18 | -4.65 |
| mmu-miR-182-5p | Gdpd2 | -0.78 | 0.002399894 | 3.32 | -1.78 |
| mmu-miR-146a-5p | Armc4 | -0.78 | 0.002406721 | 4.19 | -3.02 |
| mmu-miR-30a-5p | Slc23a2 | -0.78 | 0.002408234 | 2.87 | -1.79 |
| mmu-miR-141-3p | Fsd1l | -0.78 | 0.002409351 | 4.66 | -2.48 |
| mmu-miR-27a-3p | Pkia | -0.78 | 0.002409448 | 3.17 | -2.03 |
| mmu-miR-429-3p | Dixdc1 | -0.78 | 0.002410173 | 3.03 | -1.73 |
| mmu-miR-93-5p | Atp1a2 | -0.78 | 0.002412017 | 2.30 | -4.08 |
| mmu-miR-26b-5p | Slc4a8 | -0.78 | 0.002413243 | 3.80 | -1.64 |
| mmu-miR-504-5p | Slfn2 | -0.78 | 0.002414014 | -4.12 | 1.73 |
| mmu-miR-20a-5p | Atp1a2 | -0.78 | 0.002416793 | 3.70 | -4.08 |
| mmu-miR-31-5p | Tsnaxip1 | -0.78 | 0.002417486 | 4.65 | -1.85 |
| mmu-miR-182-5p | Ptar1 | -0.78 | 0.002419583 | 3.32 | -3.27 |
| mmu-miR-17-5p | Smoc2 | -0.78 | 0.002420777 | 3.83 | -2.15 |
| mmu-let-7g-5p | Ghr | -0.78 | 0.002424494 | 2.05 | -2.23 |
| mmu-miR-218-5p | Cetn2 | -0.78 | 0.002429657 | 3.13 | -1.85 |
| mmu-miR-29c-3p | Morn3 | -0.78 | 0.002438712 | 5.21 | -2.84 |
| mmu-let-7d-3p | Krt16 | -0.78 | 0.002442383 | -6.28 | 4.68 |
| mmu-let-7g-5p | Pkia | -0.78 | 0.002442729 | 2.05 | -2.03 |
| mmu-miR-221-3p | Dnah9 | -0.78 | 0.002466023 | 2.18 | -2.00 |
| mmu-miR-32-5p | Ptar1 | -0.78 | 0.002469452 | 4.39 | -3.27 |
| mmu-miR-26b-5p | Atp1a2 | -0.78 | 0.002473799 | 3.80 | -4.08 |
| mmu-miR-30b-5p | Slc23a2 | -0.78 | 0.002486898 | 2.59 | -1.79 |
| mmu-miR-205-3p | Mlf1 | -0.78 | 0.002492048 | 6.09 | -2.32 |
| mmu-miR-181c-5p | Lrguk | -0.78 | 0.002492603 | 3.05 | -2.08 |
| mmu-miR-32-5p | Prkar2b | -0.78 | 0.002494949 | 4.39 | -2.31 |
| mmu-miR-34a-5p | Svip | -0.78 | 0.002496959 | 3.60 | -1.64 |
| mmu-miR-141-3p | Adhfe1 | -0.78 | 0.002499467 | 4.66 | -2.39 |
| mmu-miR-10a-5p | Fbxo36 | -0.78 | 0.002500264 | 2.87 | -1.72 |
| mmu-miR-141-3p | Slc23a2 | -0.78 | 0.002501706 | 4.66 | -1.79 |
| mmu-miR-218-5p | Bche | -0.78 | 0.002502643 | 3.13 | -2.78 |
| mmu-miR-146a-5p | Dnaic1 | -0.78 | 0.002504323 | 4.19 | -1.71 |
| mmu-miR-106b-5p | Capn13 | -0.78 | 0.002504634 | 4.02 | -3.68 |
| mmu-miR-421-3p | Abca13 | -0.78 | 0.00252285 | 3.10 | -1.78 |
| mmu-miR-130a-3p | Cacna2d1 | -0.78 | 0.002522942 | 2.29 | -4.55 |
| mmu-miR-17-5p | Capn13 | -0.78 | 0.002523538 | 3.83 | -3.68 |
| mmu-miR-30c-5p | Ugt8a | -0.78 | 0.002526807 | 2.26 | -4.94 |
| mmu-miR-449c-5p | Sox4 | -0.78 | 0.002527279 | -3.77 | 1.76 |
| mmu-miR-15a-5p | Zbtb33 | -0.78 | 0.002530404 | 2.29 | -1.68 |
| mmu-miR-421-3p | Tmem67 | -0.78 | 0.002532988 | 3.10 | -2.19 |
| mmu-miR-34a-5p | Pkia | -0.78 | 0.002538494 | 3.60 | -2.03 |
| mmu-miR-23a-5p | Alpk1 | -0.77 | 0.002543775 | -2.43 | 1.89 |
| mmu-miR-30e-5p | Wdr19 | -0.77 | 0.002553824 | 3.60 | -1.81 |
| mmu-miR-15a-5p | Zmynd10 | -0.77 | 0.002554419 | 2.29 | -2.36 |
| mmu-miR-200b-3p | Intu | -0.77 | 0.002562202 | 2.19 | -1.73 |
| mmu-miR-182-5p | Kcnmb2 | -0.77 | 0.002562225 | 3.32 | -2.63 |
| mmu-miR-93-5p | Cybrd1 | -0.77 | 0.002570183 | 2.30 | -2.98 |
| mmu-miR-181a-5p | Lif | -0.77 | 0.002570966 | -3.01 | 2.93 |
| mmu-miR-425-5p | Ugt8a | -0.77 | 0.002574063 | 1.93 | -4.94 |
| mmu-miR-877-5p | Sncg | -0.77 | 0.002576254 | -4.79 | 2.33 |
| mmu-miR-106b-5p | Elmod1 | -0.77 | 0.002578014 | 4.02 | -1.66 |
| mmu-miR-218-5p | Dynlrb2 | -0.77 | 0.002579084 | 3.13 | -2.96 |
| mmu-let-7i-5p | Ptar1 | -0.77 | 0.002579085 | 1.79 | -3.27 |
| mmu-miR-92b-5p | Atf3 | -0.77 | 0.002580456 | -6.76 | 2.02 |
| mmu-miR-214-3p | Banp | -0.77 | 0.002581093 | -1.90 | 1.83 |
| mmu-miR-30b-5p | Tspyl4 | -0.77 | 0.00258233 | 2.59 | -2.33 |
| mmu-miR-30a-5p | Rgs22 | -0.77 | 0.002586901 | 2.87 | -2.80 |
| mmu-miR-15a-5p | Myb | -0.77 | 0.002589609 | 2.29 | -1.98 |
| mmu-miR-26b-5p | Slc16a7 | -0.77 | 0.00259171 | 3.80 | -3.51 |
| mmu-miR-92b-5p | Has3 | -0.77 | 0.002592649 | -6.76 | 1.63 |
| mmu-miR-141-3p | Dixdc1 | -0.77 | 0.00261951 | 4.66 | -1.73 |
| mmu-let-7d-3p | Clec4e | -0.77 | 0.002620879 | -6.28 | 2.52 |
| mmu-let-7i-5p | Lrguk | -0.77 | 0.002627891 | 1.79 | -2.08 |
| mmu-miR-34a-5p | Scgb1a1 | -0.77 | 0.002627979 | 3.60 | -2.57 |
| mmu-miR-183-3p | Prdx1 | -0.77 | 0.002640076 | 4.74 | -1.59 |
| mmu-miR-149-3p | Trim15 | -0.77 | 0.002644507 | -4.06 | 2.14 |
| mmu-miR-32-5p | Abi3bp | -0.77 | 0.00264656 | 4.39 | -3.56 |
| mmu-miR-34a-5p | Foxp2 | -0.77 | 0.002659624 | 3.60 | -2.72 |
| mmu-miR-26a-5p | Ttc29 | -0.77 | 0.002665891 | 2.26 | -2.35 |
| mmu-miR-183-3p | Ptar1 | -0.77 | 0.002670243 | 4.74 | -3.27 |
| mmu-miR-16-5p | Myb | -0.77 | 0.002673647 | 1.77 | -1.98 |
| mmu-miR-23a-5p | Tigit | -0.77 | 0.002681755 | -2.43 | 4.12 |
| mmu-miR-20a-5p | Ascl3 | -0.77 | 0.002682069 | 3.70 | -2.07 |
| mmu-let-7g-5p | Col4a6 | -0.77 | 0.002682976 | 2.05 | -1.94 |
| mmu-miR-328-3p | Ltb4r1 | -0.77 | 0.002685925 | -5.59 | 2.94 |
| mmu-miR-421-3p | Dmbt1 | -0.77 | 0.002690376 | 3.10 | -3.59 |
| mmu-miR-149-3p | Stra6 | -0.77 | 0.002691898 | -4.06 | 2.33 |
| mmu-miR-16-5p | Wdr19 | -0.77 | 0.002697054 | 1.77 | -1.81 |
| mmu-miR-92a-3p | Itga5 | -0.77 | 0.00269748 | -2.41 | 2.47 |
| mmu-miR-32-5p | Fstl1 | -0.77 | 0.002713923 | 4.39 | -2.02 |
| mmu-let-7b-5p | Olfm4 | -0.77 | 0.002716396 | -3.08 | 1.70 |
| mmu-miR-130a-3p | Ptprg | -0.77 | 0.002718204 | 2.29 | -1.60 |
| mmu-miR-30c-5p | Arl4a | -0.77 | 0.002724679 | 2.26 | -1.95 |
| mmu-miR-143-3p | Igfbp5 | -0.77 | 0.002727268 | 2.79 | -1.94 |
| mmu-miR-27a-3p | Dync2li1 | -0.77 | 0.002728699 | 3.17 | -1.85 |
| mmu-let-7g-5p | Igf2bp2 | -0.77 | 0.002729588 | 2.05 | -2.19 |
| mmu-miR-708-5p | Dync2li1 | -0.77 | 0.002730167 | 2.00 | -1.85 |
| mmu-miR-22-3p | Dnaic2 | -0.77 | 0.002733721 | 2.95 | -2.03 |
| mmu-miR-181c-5p | Lgi2 | -0.77 | 0.002746457 | 3.05 | -2.32 |
| mmu-miR-106b-5p | Pkia | -0.77 | 0.002753944 | 4.02 | -2.03 |
| mmu-miR-27a-3p | Sema3b | -0.77 | 0.002754244 | 3.17 | -1.61 |
| mmu-let-7d-3p | Dok2 | -0.77 | 0.002774411 | -6.28 | 2.80 |
| mmu-miR-26b-5p | Hpgd | -0.77 | 0.002775989 | 3.80 | -2.36 |
| mmu-miR-26a-5p | Slc16a10 | -0.77 | 0.002782145 | 2.26 | -1.94 |
| mmu-miR-421-3p | Fbxo36 | -0.77 | 0.002789796 | 3.10 | -1.72 |
| mmu-miR-29a-3p | Slc4a8 | -0.77 | 0.002797667 | 2.71 | -1.64 |
| mmu-let-7g-5p | Ccdc60 | -0.77 | 0.002798007 | 2.05 | -1.61 |
| mmu-miR-29a-3p | Dusp28 | -0.77 | 0.002799064 | 2.71 | -1.71 |
| mmu-miR-149-3p | Pde1b | -0.77 | 0.00279961 | -4.06 | 1.80 |
| mmu-miR-200b-3p | Tmem67 | -0.77 | 0.002851013 | 2.19 | -2.19 |
| mmu-miR-128-3p | Aff3 | -0.77 | 0.002859297 | 2.80 | -1.84 |
| mmu-miR-182-5p | Meig1 | -0.77 | 0.00286713 | 3.32 | -2.50 |
| mmu-miR-19b-3p | Agr3 | -0.77 | 0.002879811 | 3.59 | -2.60 |
| mmu-miR-411-5p | Map3k1 | -0.77 | 0.00289551 | 4.43 | -1.61 |
| mmu-miR-30e-5p | Htra3 | -0.77 | 0.002904248 | 3.60 | -1.92 |
| mmu-let-7f-5p | Col4a6 | -0.77 | 0.002913161 | 3.20 | -1.94 |
| mmu-miR-34a-5p | Gdpd2 | -0.77 | 0.00291589 | 3.60 | -1.78 |
| mmu-miR-143-3p | Akap6 | -0.77 | 0.002920163 | 2.79 | -2.33 |
| mmu-miR-181c-5p | Apoo | -0.77 | 0.002936227 | 3.05 | -3.38 |
| mmu-miR-16-5p | Rora | -0.77 | 0.002943077 | 1.77 | -2.00 |
| mmu-miR-328-3p | Mybl2 | -0.77 | 0.002952919 | -5.59 | 1.71 |
| mmu-let-7b-5p | Gmip | -0.77 | 0.002968595 | -3.08 | 2.22 |
| mmu-miR-181c-5p | Osbpl6 | -0.77 | 0.002970123 | 3.05 | -1.73 |
| mmu-miR-26a-5p | Slc4a8 | -0.77 | 0.002984407 | 2.26 | -1.64 |
| mmu-miR-29c-3p | Pxmp2 | -0.77 | 0.002997082 | 5.21 | -3.43 |
| mmu-miR-19b-3p | Fundc1 | -0.77 | 0.003022205 | 3.59 | -1.70 |
| mmu-miR-708-5p | Tcp11 | -0.77 | 0.003023197 | 2.00 | -1.73 |
| mmu-let-7i-5p | Ccdc81 | -0.77 | 0.003030943 | 1.79 | -1.59 |
| mmu-miR-205-3p | Pcca | -0.77 | 0.003037384 | 6.09 | -1.94 |
| mmu-let-7f-5p | Apoo | -0.77 | 0.003041938 | 3.20 | -3.38 |
| mmu-miR-223-3p | Tmem67 | -0.76 | 0.003054981 | 9.15 | -2.19 |
| mmu-miR-26a-5p | Atp1a2 | -0.76 | 0.003061911 | 2.26 | -4.08 |
| mmu-miR-30a-5p | Ccdc60 | -0.76 | 0.003067577 | 2.87 | -1.61 |
| mmu-miR-130a-3p | Smoc2 | -0.76 | 0.003078771 | 2.29 | -2.15 |
| mmu-miR-16-5p | Ghr | -0.76 | 0.003081759 | 1.77 | -2.23 |
| mmu-miR-141-3p | Tcp11 | -0.76 | 0.003086191 | 4.66 | -1.73 |
| mmu-miR-30b-5p | Six4 | -0.76 | 0.003088126 | 2.59 | -1.65 |
| mmu-miR-218-5p | Acyp2 | -0.76 | 0.003101782 | 3.13 | -2.40 |
| mmu-miR-132-3p | Slc23a2 | -0.76 | 0.003105485 | 2.20 | -1.79 |
| mmu-miR-24-3p | Dcn | -0.76 | 0.00311212 | 1.73 | -4.28 |
| mmu-miR-214-5p | Foxp2 | -0.76 | 0.003112464 | 3.66 | -2.72 |
| mmu-miR-223-3p | Gpr155 | -0.76 | 0.003114044 | 9.15 | -2.57 |
| mmu-miR-15a-5p | Elmod1 | -0.76 | 0.003118522 | 2.29 | -1.66 |
| mmu-miR-760-3p | Mfi2 | -0.76 | 0.003125783 | -7.15 | 2.43 |
| mmu-miR-183-3p | Cyp4b1 | -0.76 | 0.003141539 | 4.74 | -1.87 |
| mmu-miR-30b-5p | Intu | -0.76 | 0.003148596 | 2.59 | -1.73 |
| mmu-miR-200a-3p | Adhfe1 | -0.76 | 0.003150958 | 2.61 | -2.39 |
| mmu-miR-17-5p | Atp1a2 | -0.76 | 0.003175237 | 3.83 | -4.08 |
| mmu-miR-17-5p | Cybrd1 | -0.76 | 0.003183437 | 3.83 | -2.98 |
| mmu-miR-24-3p | Cd34 | -0.76 | 0.003188694 | 1.73 | -3.73 |
| mmu-let-7d-3p | Sulf2 | -0.76 | 0.003190959 | -6.28 | 2.72 |
| mmu-miR-135b-5p | Slitrk6 | -0.76 | 0.003197032 | 4.89 | -3.34 |
| mmu-miR-200a-5p | Pfkm | -0.76 | 0.003198738 | 2.31 | -1.75 |
| mmu-miR-421-3p | Prkaa2 | -0.76 | 0.003199068 | 3.10 | -2.21 |
| mmu-miR-23b-3p | Apoo | -0.76 | 0.003200195 | 2.62 | -3.38 |
| mmu-miR-200a-3p | Bmpr1b | -0.76 | 0.003207252 | 2.61 | -2.84 |
| mmu-miR-421-3p | Myh10 | -0.76 | 0.003214328 | 3.10 | -1.69 |
| mmu-miR-99b-5p | Slc15a2 | -0.76 | 0.003216672 | 1.97 | -3.27 |
| mmu-miR-19b-3p | Rwdd3 | -0.76 | 0.003218551 | 3.59 | -2.36 |
| mmu-miR-149-3p | Mmp9 | -0.76 | 0.003230233 | -4.06 | 2.37 |
| mmu-miR-23b-3p | Ptar1 | -0.76 | 0.003239343 | 2.62 | -3.27 |
| mmu-miR-200a-3p | Slc23a2 | -0.76 | 0.003240637 | 2.61 | -1.79 |
| mmu-miR-26b-5p | Ttc29 | -0.76 | 0.003243839 | 3.80 | -2.35 |
| mmu-miR-15a-5p | Fundc1 | -0.76 | 0.003245929 | 2.29 | -1.70 |
| mmu-miR-98-5p | Dnah7b | -0.76 | 0.003259394 | 3.72 | -1.97 |
| mmu-miR-30c-5p | Dtna | -0.76 | 0.003262324 | 2.26 | -1.62 |
| mmu-miR-194-5p | Chad | -0.76 | 0.003273952 | 4.92 | -6.22 |
| mmu-miR-17-5p | Rragd | -0.76 | 0.003282118 | 3.83 | -2.21 |
| mmu-miR-31-5p | 6820408C15Rik | -0.76 | 0.003282882 | 4.65 | -2.61 |
| mmu-miR-340-5p | Tmem67 | -0.76 | 0.003284978 | 9.01 | -2.19 |
| mmu-miR-155-5p | Csrp2 | -0.76 | 0.003298036 | 1.93 | -2.18 |
| mmu-miR-32-5p | Sar1b | -0.76 | 0.003310048 | 4.39 | -1.65 |
| mmu-miR-328-3p | Sncg | -0.76 | 0.003313904 | -5.59 | 2.33 |
| mmu-miR-23a-5p | Ms4a6d | -0.76 | 0.003317162 | -2.43 | 2.29 |
| mmu-miR-148a-3p | Adcy2 | -0.76 | 0.003321348 | 2.94 | -2.55 |
| mmu-miR-708-5p | Rora | -0.76 | 0.003325742 | 2.00 | -2.00 |
| mmu-miR-760-3p | Ereg | -0.76 | 0.003329831 | -7.15 | 2.33 |
| mmu-miR-421-3p | Cyp39a1 | -0.76 | 0.003332167 | 3.10 | -1.84 |
| mmu-miR-32-5p | Arhgap29 | -0.76 | 0.003333483 | 4.39 | -2.19 |
| mmu-miR-20a-5p | Cybrd1 | -0.76 | 0.003342018 | 3.70 | -2.98 |
| mmu-miR-29a-3p | Smtnl2 | -0.76 | 0.003350574 | 2.71 | -2.05 |
| mmu-miR-340-5p | Trf | -0.76 | 0.003363362 | 9.01 | -2.46 |
| mmu-let-7i-5p | Rragd | -0.76 | 0.003364163 | 1.79 | -2.21 |
| mmu-miR-15a-5p | Nme5 | -0.76 | 0.003366481 | 2.29 | -2.53 |
| mmu-miR-30e-5p | Aff3 | -0.76 | 0.003378501 | 3.60 | -1.84 |
| mmu-miR-223-3p | Ttll9 | -0.76 | 0.00338472 | 9.15 | -1.73 |
| mmu-miR-181c-5p | Eln | -0.76 | 0.003387407 | 3.05 | -2.85 |
| mmu-miR-30a-3p | Intu | -0.76 | 0.003391737 | 1.94 | -1.73 |
| mmu-miR-205-3p | Pfkm | -0.76 | 0.003416427 | 6.09 | -1.75 |
| mmu-let-7b-5p | Pik3ap1 | -0.76 | 0.003421637 | -3.08 | 2.92 |
| mmu-miR-223-3p | Setbp1 | -0.76 | 0.003424438 | 9.15 | -1.63 |
| mmu-miR-106b-5p | Adhfe1 | -0.76 | 0.003426725 | 4.02 | -2.39 |
| mmu-miR-200a-3p | Fsd1l | -0.76 | 0.003443201 | 2.61 | -2.48 |
| mmu-miR-192-5p | Tacr1 | -0.76 | 0.003449336 | 5.95 | -5.28 |
| mmu-miR-181a-5p | Cyp4f18 | -0.76 | 0.003450635 | -3.01 | 4.07 |
| mmu-miR-93-5p | Zbtb33 | -0.76 | 0.003463241 | 2.30 | -1.68 |
| mmu-miR-185-5p | Ric3 | -0.76 | 0.003465393 | 2.50 | -2.30 |
| mmu-miR-32-5p | Wnt5a | -0.76 | 0.003465607 | 4.39 | -2.52 |
| mmu-miR-210-5p | Cpne2 | -0.76 | 0.003470837 | -3.25 | 2.13 |
| mmu-miR-192-5p | Cyp4a12b | -0.76 | 0.00347428 | 5.95 | -2.32 |
| mmu-miR-223-3p | 6-Sep | -0.76 | 0.00349284 | 9.15 | -1.64 |
| mmu-miR-29c-3p | 4931406C07Rik | -0.76 | 0.003510122 | 5.21 | -2.42 |
| mmu-miR-326-3p | Cep250 | -0.76 | 0.003512778 | -1.76 | 1.60 |
| mmu-miR-410-3p | Myh10 | -0.76 | 0.003517592 | 3.62 | -1.69 |
| mmu-miR-25-3p | Ptar1 | -0.76 | 0.003533421 | 2.56 | -3.27 |
| mmu-miR-16-5p | Elmod1 | -0.76 | 0.00354137 | 1.77 | -1.66 |
| mmu-miR-98-5p | Ptar1 | -0.76 | 0.003555498 | 3.72 | -3.27 |
| mmu-miR-135b-5p | Il13ra1 | -0.76 | 0.003556057 | 4.89 | -1.76 |
| mmu-miR-183-3p | Scara5 | -0.76 | 0.003559932 | 4.74 | -2.63 |
| mmu-miR-185-5p | Spag16 | -0.76 | 0.003564891 | 2.50 | -3.82 |
| mmu-miR-200a-3p | Myh10 | -0.76 | 0.003565418 | 2.61 | -1.69 |
| mmu-miR-218-5p | Hpgd | -0.76 | 0.003573768 | 3.13 | -2.36 |
| mmu-miR-221-3p | Axin2 | -0.76 | 0.003606492 | 2.18 | -1.66 |
| mmu-miR-205-3p | Cadm1 | -0.76 | 0.003610473 | 6.09 | -1.72 |
| mmu-miR-132-3p | Aldh1a1 | -0.75 | 0.003642317 | 2.20 | -1.70 |
| mmu-miR-30e-5p | Tmem35 | -0.75 | 0.003660806 | 3.60 | -1.94 |
| mmu-miR-29a-3p | Ccdc81 | -0.75 | 0.00367116 | 2.71 | -1.59 |
| mmu-miR-30a-5p | Spa17 | -0.75 | 0.003675957 | 2.87 | -1.63 |
| mmu-miR-148a-3p | Dnah11 | -0.75 | 0.003700374 | 2.94 | -1.85 |
| mmu-miR-200a-3p | Tcp11 | -0.75 | 0.003709927 | 2.61 | -1.73 |
| mmu-miR-23a-5p | Fam20c | -0.75 | 0.003711701 | -2.43 | 2.32 |
| mmu-miR-106b-5p | Atp1a2 | -0.75 | 0.003733733 | 4.02 | -4.08 |
| mmu-miR-144-5p | Eln | -0.75 | 0.003740457 | 5.17 | -2.85 |
| mmu-miR-19b-3p | Foxp2 | -0.75 | 0.003741728 | 3.59 | -2.72 |
| mmu-miR-132-3p | Cdhr3 | -0.75 | 0.003755441 | 2.20 | -1.83 |
| mmu-miR-223-3p | 1700026D08Rik | -0.75 | 0.003756067 | 9.15 | -2.36 |
| mmu-miR-410-3p | Sar1b | -0.75 | 0.003766747 | 3.62 | -1.65 |
| mmu-miR-429-3p | Adhfe1 | -0.75 | 0.003768431 | 3.03 | -2.39 |
| mmu-miR-132-3p | Ephx2 | -0.75 | 0.003776877 | 2.20 | -1.99 |
| mmu-miR-34a-5p | Fgfr1 | -0.75 | 0.003789339 | 3.60 | -1.71 |
| mmu-miR-212-3p | Rora | -0.75 | 0.003803298 | 3.43 | -2.00 |
| mmu-miR-23a-3p | Apoo | -0.75 | 0.003812246 | 3.01 | -3.38 |
| mmu-let-7b-5p | Tnfrsf1b | -0.75 | 0.003815316 | -3.08 | 1.68 |
| mmu-miR-30b-5p | C1s1 | -0.75 | 0.003820864 | 2.59 | -2.40 |
| mmu-miR-200a-3p | Foxp2 | -0.75 | 0.003834194 | 2.61 | -2.72 |
| mmu-let-7f-5p | Dync2li1 | -0.75 | 0.003845299 | 3.20 | -1.85 |
| mmu-miR-92b-5p | Ripk3 | -0.75 | 0.003845729 | -6.76 | 1.70 |
| mmu-miR-92a-3p | Slc7a11 | -0.75 | 0.003853011 | -2.41 | 1.80 |
| mmu-miR-19b-3p | Dnah6 | -0.75 | 0.003855172 | 3.59 | -1.93 |
| mmu-miR-200b-3p | Pkia | -0.75 | 0.003856673 | 2.19 | -2.03 |
| mmu-miR-410-3p | Acsl3 | -0.75 | 0.003859576 | 3.62 | -1.65 |
| mmu-miR-25-5p | Arntl2 | -0.75 | 0.003874663 | -2.35 | 3.18 |
| mmu-miR-181c-5p | Sox5 | -0.75 | 0.003879126 | 3.05 | -2.90 |
| mmu-miR-27a-3p | Atp1a2 | -0.75 | 0.00388924 | 3.17 | -4.08 |
| mmu-let-7d-3p | Cep250 | -0.75 | 0.003903517 | -6.28 | 1.60 |
| mmu-miR-23b-3p | 1700084C01Rik | -0.75 | 0.003912509 | 2.62 | -3.29 |
| mmu-miR-19b-3p | Fam69a | -0.75 | 0.003915989 | 3.59 | -2.33 |
| mmu-miR-23a-3p | Pkia | -0.75 | 0.003916036 | 3.01 | -2.03 |
| mmu-let-7d-3p | Msr1 | -0.75 | 0.003921937 | -6.28 | 3.60 |
| mmu-let-7g-5p | Dync2li1 | -0.75 | 0.003944515 | 2.05 | -1.85 |
| mmu-miR-182-5p | Bmpr1b | -0.75 | 0.003952916 | 3.32 | -2.84 |
| mmu-miR-504-5p | Has3 | -0.75 | 0.003957602 | -4.12 | 1.63 |
| mmu-miR-340-5p | Six4 | -0.75 | 0.003973763 | 9.01 | -1.65 |
| mmu-miR-203-3p | C1s1 | -0.75 | 0.003982239 | 4.73 | -2.40 |
| mmu-miR-425-5p | B4galt4 | -0.75 | 0.003982424 | 1.93 | -2.19 |
| mmu-miR-98-5p | Col4a6 | -0.75 | 0.003985389 | 3.72 | -1.94 |
| mmu-miR-183-3p | Ttc26 | -0.75 | 0.003990718 | 4.74 | -1.81 |
| mmu-miR-429-3p | Intu | -0.75 | 0.003997196 | 3.03 | -1.73 |
| mmu-miR-192-5p | Bgn | -0.75 | 0.00400704 | 5.95 | -1.97 |
| mmu-miR-141-3p | Elmod1 | -0.75 | 0.004014486 | 4.66 | -1.66 |
| mmu-miR-32-5p | Col1a2 | -0.75 | 0.004025347 | 4.39 | -2.33 |
| mmu-miR-132-3p | Cdkl4 | -0.75 | 0.004040118 | 2.20 | -2.06 |
| mmu-miR-152-3p | Trf | -0.75 | 0.004042365 | 1.92 | -2.46 |
| mmu-let-7i-5p | Galc | -0.75 | 0.004042381 | 1.79 | -1.71 |
| mmu-miR-185-3p | Faim2 | -0.75 | 0.004046337 | -3.01 | 1.65 |
| mmu-miR-340-5p | Dixdc1 | -0.75 | 0.00405312 | 9.01 | -1.73 |
| mmu-miR-130a-3p | Aff3 | -0.75 | 0.004056202 | 2.29 | -1.84 |
| mmu-miR-34a-5p | Gfra1 | -0.75 | 0.00407507 | 3.60 | -3.67 |
| mmu-miR-182-5p | Gfra1 | -0.75 | 0.004080037 | 3.32 | -3.67 |
| mmu-miR-30a-5p | Wdr19 | -0.75 | 0.004109248 | 2.87 | -1.81 |
| mmu-miR-106b-5p | Smoc2 | -0.75 | 0.004127102 | 4.02 | -2.15 |
| mmu-miR-375-3p | Fam20c | -0.75 | 0.00413836 | -2.94 | 2.32 |
| mmu-miR-223-3p | Meig1 | -0.75 | 0.004143753 | 9.15 | -2.50 |
| mmu-let-7d-3p | Ccl6 | -0.75 | 0.004149808 | -6.28 | 2.24 |
| mmu-miR-24-3p | Fam45a | -0.75 | 0.004156962 | 1.73 | -1.61 |
| mmu-miR-30b-5p | Agr3 | -0.75 | 0.004175347 | 2.59 | -2.60 |
| mmu-miR-141-3p | Bmpr1b | -0.75 | 0.004175808 | 4.66 | -2.84 |
| mmu-miR-155-5p | Ccdc89 | -0.75 | 0.004206987 | 1.93 | -1.73 |
| mmu-let-7g-5p | Pm20d2 | -0.75 | 0.004223129 | 2.05 | -2.00 |
| mmu-miR-19b-3p | Dnaic1 | -0.75 | 0.004237214 | 3.59 | -1.71 |
| mmu-miR-877-5p | Cd33 | -0.75 | 0.004239684 | -4.79 | 1.91 |
| mmu-miR-183-3p | Pam | -0.74 | 0.004258385 | 4.74 | -1.83 |
| mmu-let-7f-5p | Galc | -0.74 | 0.004268646 | 3.20 | -1.71 |
| mmu-miR-200b-3p | Apoo | -0.74 | 0.00428832 | 2.19 | -3.38 |
| mmu-miR-429-3p | Tmem67 | -0.74 | 0.004289842 | 3.03 | -2.19 |
| mmu-miR-326-3p | 5730559C18Rik | -0.74 | 0.004292003 | -1.76 | 1.64 |
| mmu-miR-200b-3p | Ppp2r2b | -0.74 | 0.004298438 | 2.19 | -1.83 |
| mmu-miR-212-3p | Gmnn | -0.74 | 0.004302361 | 3.43 | -2.26 |
| mmu-miR-135b-5p | Tbc1d30 | -0.74 | 0.004338769 | 4.89 | -2.52 |
| mmu-miR-16-5p | Dnah9 | -0.74 | 0.004346882 | 1.77 | -2.00 |
| mmu-miR-340-5p | St8sia2 | -0.74 | 0.004350182 | 9.01 | -1.66 |
| mmu-miR-19b-3p | Smoc2 | -0.74 | 0.004351608 | 3.59 | -2.15 |
| mmu-miR-32-5p | Kifap3 | -0.74 | 0.00435381 | 4.39 | -1.75 |
| mmu-miR-130a-3p | Ulk4 | -0.74 | 0.004375569 | 2.29 | -1.85 |
| mmu-miR-210-3p | Dnah11 | -0.74 | 0.004377932 | 1.86 | -1.85 |
| mmu-miR-29c-3p | Dixdc1 | -0.74 | 0.004378558 | 5.21 | -1.73 |
| mmu-miR-421-3p | Rora | -0.74 | 0.004378906 | 3.10 | -2.00 |
| mmu-miR-30e-5p | C1s1 | -0.74 | 0.004378968 | 3.60 | -2.40 |
| mmu-miR-214-5p | Cyp4a12a | -0.74 | 0.004381313 | 3.66 | -3.14 |
| mmu-miR-200b-3p | Adhfe1 | -0.74 | 0.004391766 | 2.19 | -2.39 |
| mmu-let-7i-5p | Scd1 | -0.74 | 0.00442833 | 1.79 | -1.90 |
| mmu-miR-326-3p | Sesn2 | -0.74 | 0.004446168 | -1.76 | 1.67 |
| mmu-miR-30c-5p | Rgs22 | -0.74 | 0.004454522 | 2.26 | -2.80 |
| mmu-miR-183-3p | Arhgap18 | -0.74 | 0.004468216 | 4.74 | -1.77 |
| mmu-miR-98-5p | Dync2li1 | -0.74 | 0.004493478 | 3.72 | -1.85 |
| mmu-miR-25-5p | Osr1 | -0.74 | 0.004504539 | -2.35 | 1.93 |
| mmu-miR-34a-5p | Slc23a2 | -0.74 | 0.004506557 | 3.60 | -1.79 |
| mmu-miR-30a-5p | Tspyl4 | -0.74 | 0.004511472 | 2.87 | -2.33 |
| mmu-miR-92a-3p | Pik3ap1 | -0.74 | 0.004535937 | -2.41 | 2.92 |
| mmu-miR-27b-3p | Slc16a10 | -0.74 | 0.004551638 | 2.45 | -1.94 |
| mmu-miR-148a-3p | 1190002N15Rik | -0.74 | 0.004565806 | 2.94 | -1.84 |
| mmu-miR-30b-5p | Spa17 | -0.74 | 0.004591275 | 2.59 | -1.63 |
| mmu-miR-340-5p | Casc1 | -0.74 | 0.004593855 | 9.01 | -1.66 |
| mmu-miR-29c-3p | Sms | -0.74 | 0.004607247 | 5.21 | -2.09 |
| mmu-miR-375-3p | Slc7a11 | -0.74 | 0.004618106 | -2.94 | 1.80 |
| mmu-miR-378c | Slc38a1 | -0.74 | 0.00464065 | 4.40 | -3.08 |
| mmu-miR-29a-3p | 6820408C15Rik | -0.74 | 0.004647552 | 2.71 | -2.61 |
| mmu-miR-29c-3p | Arl4a | -0.74 | 0.00465092 | 5.21 | -1.95 |
| mmu-miR-429-3p | Apoo | -0.74 | 0.004658766 | 3.03 | -3.38 |
| mmu-miR-340-5p | Dync2li1 | -0.74 | 0.004659485 | 9.01 | -1.85 |
| mmu-miR-22-3p | Gfra1 | -0.74 | 0.004693851 | 2.95 | -3.67 |
| mmu-miR-183-3p | Dtna | -0.74 | 0.004708701 | 4.74 | -1.62 |
| mmu-miR-30a-5p | Acyp2 | -0.74 | 0.004714354 | 2.87 | -2.40 |
| mmu-miR-19b-3p | Fbxo16 | -0.74 | 0.004738989 | 3.59 | -2.41 |
| mmu-let-7f-5p | Pm20d2 | -0.74 | 0.004760187 | 3.20 | -2.00 |
| mmu-miR-15a-5p | Oscp1 | -0.74 | 0.00476052 | 2.29 | -1.84 |
| mmu-miR-149-3p | Arc | -0.74 | 0.004787872 | -4.06 | 1.92 |
| mmu-miR-429-3p | Pkia | -0.74 | 0.004796465 | 3.03 | -2.03 |
| mmu-miR-410-3p | Foxp2 | -0.74 | 0.004805704 | 3.62 | -2.72 |
| mmu-miR-26b-5p | Rpgr | -0.74 | 0.004840456 | 3.80 | -2.01 |
| mmu-let-7g-5p | Galc | -0.74 | 0.004875859 | 2.05 | -1.71 |
| mmu-miR-425-5p | Scara5 | -0.74 | 0.004881918 | 1.93 | -2.63 |
| mmu-miR-16-5p | Oscp1 | -0.74 | 0.004888647 | 1.77 | -1.84 |
| mmu-miR-183-3p | Pcca | -0.74 | 0.004905136 | 4.74 | -1.94 |
| mmu-miR-25-5p | Notch1 | -0.74 | 0.004907643 | -2.35 | 1.81 |
| mmu-miR-26a-5p | Rpgr | -0.74 | 0.004925367 | 2.26 | -2.01 |
| mmu-miR-744-5p | Rab20 | -0.74 | 0.004945129 | -3.34 | 2.03 |
| mmu-miR-205-3p | Intu | -0.74 | 0.004967183 | 6.09 | -1.73 |
| mmu-miR-30e-5p | Ccdc60 | -0.73 | 0.004985955 | 3.60 | -1.61 |
| mmu-miR-106b-5p | St8sia2 | -0.73 | 0.005004756 | 4.02 | -1.66 |
| mmu-miR-200b-3p | Scd1 | -0.73 | 0.005021459 | 2.19 | -1.90 |
| mmu-miR-194-5p | Tbc1d30 | -0.73 | 0.005029028 | 4.92 | -2.52 |
| mmu-miR-152-3p | Sms | -0.73 | 0.005038517 | 1.92 | -2.09 |
| mmu-miR-223-3p | Slc23a2 | -0.73 | 0.005047146 | 9.15 | -1.79 |
| mmu-miR-10a-5p | Efhc1 | -0.73 | 0.005054861 | 2.87 | -2.17 |
| mmu-miR-32-5p | Rora | -0.73 | 0.005057689 | 4.39 | -2.00 |
| mmu-miR-30a-5p | Six4 | -0.73 | 0.005059296 | 2.87 | -1.65 |
| mmu-miR-24-3p | Htra3 | -0.73 | 0.005079917 | 1.73 | -1.92 |
| mmu-miR-148a-3p | Grb14 | -0.73 | 0.00508448 | 2.94 | -1.73 |
| mmu-miR-16-5p | Hydin | -0.73 | 0.005114291 | 1.77 | -2.12 |
| mmu-miR-326-3p | Ecm1 | -0.73 | 0.005130747 | -1.76 | 2.00 |
| mmu-miR-10a-5p | Smtnl2 | -0.73 | 0.005144695 | 2.87 | -2.05 |
| mmu-let-7i-5p | Nxt2 | -0.73 | 0.005146683 | 1.79 | -2.84 |
| mmu-miR-185-5p | Cyp4v3 | -0.73 | 0.005155587 | 2.50 | -1.71 |
| mmu-miR-30e-5p | Spa17 | -0.73 | 0.005171011 | 3.60 | -1.63 |
| mmu-miR-181a-5p | Zbp1 | -0.73 | 0.00519264 | -3.01 | 2.54 |
| mmu-miR-135b-5p | Aldh6a1 | -0.73 | 0.005204229 | 4.89 | -2.30 |
| mmu-miR-340-5p | Pm20d2 | -0.73 | 0.005233406 | 9.01 | -2.00 |
| mmu-miR-205-3p | Aff3 | -0.73 | 0.005244505 | 6.09 | -1.84 |
| mmu-miR-106b-5p | Rragd | -0.73 | 0.00524962 | 4.02 | -2.21 |
| mmu-miR-30b-5p | Ccdc60 | -0.73 | 0.005274851 | 2.59 | -1.61 |
| mmu-miR-218-5p | Foxp2 | -0.73 | 0.005278249 | 3.13 | -2.72 |
| mmu-miR-27a-3p | Rpgrip1l | -0.73 | 0.005293582 | 3.17 | -1.64 |
| mmu-miR-29c-5p | Sparc | -0.73 | 0.005295679 | 3.44 | -1.95 |
| mmu-miR-135b-5p | Lrp11 | -0.73 | 0.005297276 | 4.89 | -1.71 |
| mmu-miR-155-5p | Wdr19 | -0.73 | 0.005298977 | 1.93 | -1.81 |
| mmu-miR-410-3p | Slc4a8 | -0.73 | 0.005300651 | 3.62 | -1.64 |
| mmu-miR-504-5p | Lst1 | -0.73 | 0.005304149 | -4.12 | 2.38 |
| mmu-miR-20a-5p | Smoc2 | -0.73 | 0.005318276 | 3.70 | -2.15 |
| mmu-miR-128-3p | Dtna | -0.73 | 0.005362324 | 2.80 | -1.62 |
| mmu-miR-130a-3p | Stk33 | -0.73 | 0.005387228 | 2.29 | -2.78 |
| mmu-miR-25-3p | Actc1 | -0.73 | 0.005441587 | 2.56 | -2.17 |
| mmu-miR-194-5p | Adhfe1 | -0.73 | 0.005443124 | 4.92 | -2.39 |
| mmu-miR-98-5p | Galc | -0.73 | 0.005444638 | 3.72 | -1.71 |
| mmu-miR-205-3p | 1700024G13Rik | -0.73 | 0.005447698 | 6.09 | -3.00 |
| mmu-miR-106b-5p | Dnah6 | -0.73 | 0.005480145 | 4.02 | -1.93 |
| mmu-miR-155-5p | Pcca | -0.73 | 0.00551379 | 1.93 | -1.94 |
| mmu-miR-218-5p | Spa17 | -0.73 | 0.005529405 | 3.13 | -1.63 |
| mmu-miR-23a-5p | Csf1 | -0.73 | 0.005548385 | -2.43 | 2.24 |
| mmu-miR-152-3p | Six4 | -0.73 | 0.005594168 | 1.92 | -1.65 |
| mmu-miR-200a-3p | St3gal6 | -0.73 | 0.005601044 | 2.61 | -2.31 |
| mmu-miR-132-3p | 1700007G11Rik | -0.73 | 0.005609427 | 2.20 | -1.74 |
| mmu-miR-27b-3p | Nxt2 | -0.73 | 0.005622179 | 2.45 | -2.84 |
| mmu-miR-193b-3p | Cd53 | -0.73 | 0.005623061 | -2.01 | 1.78 |
| mmu-miR-760-3p | Fgr | -0.73 | 0.005623897 | -7.15 | 2.06 |
| mmu-miR-193b-3p | Csf2ra | -0.73 | 0.005664384 | -2.01 | 1.64 |
| mmu-miR-203-3p | Mrpl1 | -0.73 | 0.005668521 | 4.73 | -2.74 |
| mmu-miR-30c-5p | Rab4a | -0.73 | 0.005672941 | 2.26 | -1.73 |
| mmu-miR-23a-5p | Sesn2 | -0.73 | 0.005690037 | -2.43 | 1.67 |
| mmu-miR-429-3p | Setbp1 | -0.73 | 0.005701702 | 3.03 | -1.63 |
| mmu-miR-148a-3p | Rora | -0.73 | 0.00572369 | 2.94 | -2.00 |
| mmu-miR-181a-5p | Lmo1 | -0.73 | 0.005775576 | -3.01 | 2.34 |
| mmu-miR-29a-3p | Col1a1 | -0.72 | 0.005806249 | 2.71 | -1.71 |
| mmu-miR-130a-3p | Atp1a2 | -0.72 | 0.005806841 | 2.29 | -4.08 |
| mmu-miR-30c-5p | Htra3 | -0.72 | 0.005807354 | 2.26 | -1.92 |
| mmu-miR-32-5p | Srpx2 | -0.72 | 0.005812016 | 4.39 | -2.81 |
| mmu-miR-141-3p | St8sia2 | -0.72 | 0.005824443 | 4.66 | -1.66 |
| mmu-miR-421-3p | Dync2h1 | -0.72 | 0.005954431 | 3.10 | -1.72 |
| mmu-miR-15a-5p | Hydin | -0.72 | 0.005956037 | 2.29 | -2.12 |
| mmu-miR-183-3p | Htra3 | -0.72 | 0.005973647 | 4.74 | -1.92 |
| mmu-miR-200a-3p | Srpx2 | -0.72 | 0.005984322 | 2.61 | -2.81 |
| mmu-miR-15a-5p | Slc4a8 | -0.72 | 0.005988582 | 2.29 | -1.64 |
| mmu-miR-132-3p | Wdr19 | -0.72 | 0.005998712 | 2.20 | -1.81 |
| mmu-miR-130a-3p | Abhd3 | -0.72 | 0.006007702 | 2.29 | -2.19 |
| mmu-miR-375-3p | Lcp2 | -0.72 | 0.006017564 | -2.94 | 2.21 |
| mmu-let-7b-5p | Socs1 | -0.72 | 0.006023472 | -3.08 | 1.90 |
| mmu-miR-23a-5p | Atf3 | -0.72 | 0.006026662 | -2.43 | 2.02 |
| mmu-miR-17-5p | Adhfe1 | -0.72 | 0.006047688 | 3.83 | -2.39 |
| mmu-miR-141-3p | Col4a6 | -0.72 | 0.006075038 | 4.66 | -1.94 |
| mmu-miR-425-5p | Casc1 | -0.72 | 0.006120049 | 1.93 | -1.66 |
| mmu-miR-30a-5p | Gfra1 | -0.72 | 0.006130607 | 2.87 | -3.67 |
| mmu-miR-193b-3p | Rbp1 | -0.72 | 0.006170824 | -2.01 | 2.21 |
| mmu-miR-144-5p | Rora | -0.72 | 0.006180208 | 5.17 | -2.00 |
| mmu-miR-15a-5p | Foxp2 | -0.72 | 0.006198869 | 2.29 | -2.72 |
| mmu-miR-30c-5p | Myh10 | -0.72 | 0.006208291 | 2.26 | -1.69 |
| mmu-miR-425-5p | Spag17 | -0.72 | 0.006230773 | 1.93 | -2.77 |
| mmu-miR-340-5p | Smtnl2 | -0.72 | 0.0062343 | 9.01 | -2.05 |
| mmu-miR-23a-5p | Ptger4 | -0.72 | 0.006312223 | -2.43 | 2.20 |
| mmu-miR-98-5p | Ccdc81 | -0.72 | 0.006372061 | 3.72 | -1.59 |
| mmu-miR-194-5p | Aldh6a1 | -0.72 | 0.006387333 | 4.92 | -2.30 |
| mmu-miR-148a-3p | Cacna2d1 | -0.72 | 0.006430804 | 2.94 | -4.55 |
| mmu-miR-30c-5p | Aff3 | -0.72 | 0.006440308 | 2.26 | -1.84 |
| mmu-miR-214-5p | Tigd2 | -0.72 | 0.006441816 | 3.66 | -1.67 |
| mmu-miR-500-3p | Ptar1 | -0.72 | 0.006448113 | 3.74 | -3.27 |
| mmu-miR-155-5p | Fundc1 | -0.72 | 0.006468015 | 1.93 | -1.70 |
| mmu-miR-149-3p | Sh3bp2 | -0.72 | 0.006468608 | -4.06 | 2.98 |
| mmu-miR-99b-5p | Tacr1 | -0.72 | 0.006471004 | 1.97 | -5.28 |
| mmu-miR-22-3p | Osbpl6 | -0.72 | 0.006491936 | 2.95 | -1.73 |
| mmu-miR-148a-3p | Chn2 | -0.72 | 0.006507642 | 2.94 | -1.90 |
| mmu-miR-181c-5p | Ugt8a | -0.72 | 0.006516912 | 3.05 | -4.94 |
| mmu-miR-30c-5p | Intu | -0.72 | 0.00655216 | 2.26 | -1.73 |
| mmu-miR-130a-3p | Bmpr1b | -0.72 | 0.006554017 | 2.29 | -2.84 |
| mmu-let-7i-5p | Dysf | -0.72 | 0.006560415 | 1.79 | -2.88 |
| mmu-miR-205-3p | St3gal6 | -0.72 | 0.006564155 | 6.09 | -2.31 |
| mmu-miR-23b-3p | Aldh1a2 | -0.72 | 0.006574884 | 2.62 | -3.23 |
| mmu-miR-29c-3p | Rab4a | -0.72 | 0.006599719 | 5.21 | -1.73 |
| mmu-miR-410-3p | Hibch | -0.72 | 0.00660582 | 3.62 | -2.10 |
| mmu-miR-218-5p | Kit | -0.72 | 0.006606491 | 3.13 | -1.91 |
| mmu-miR-27a-3p | Nxt2 | -0.72 | 0.006629699 | 3.17 | -2.84 |
| mmu-miR-152-3p | Slc25a23 | -0.72 | 0.006638502 | 1.92 | -2.24 |
| mmu-miR-425-5p | Rpgr | -0.72 | 0.006645614 | 1.93 | -2.01 |
| mmu-miR-155-5p | St8sia2 | -0.72 | 0.006667763 | 1.93 | -1.66 |
| mmu-let-7i-5p | Slc16a10 | -0.72 | 0.006676275 | 1.79 | -1.94 |
| mmu-miR-504-5p | Nrp2 | -0.72 | 0.006678239 | -4.12 | 1.65 |
| mmu-miR-410-3p | Rab4a | -0.72 | 0.006690088 | 3.62 | -1.73 |
| mmu-miR-218-5p | Smyd3 | -0.71 | 0.006702593 | 3.13 | -1.91 |
| mmu-miR-106b-5p | Ascl3 | -0.71 | 0.006709238 | 4.02 | -2.07 |
| mmu-miR-200b-3p | Actc1 | -0.71 | 0.006712811 | 2.19 | -2.17 |
| mmu-miR-30c-5p | C1s1 | -0.71 | 0.006722102 | 2.26 | -2.40 |
| mmu-miR-23a-3p | 1700084C01Rik | -0.71 | 0.006748417 | 3.01 | -3.29 |
| mmu-miR-433-3p | 9430015G10Rik | -0.71 | 0.006765394 | -4.72 | 1.93 |
| mmu-miR-34a-5p | Lars2 | -0.71 | 0.006790477 | 3.60 | -2.44 |
| mmu-miR-411-5p | Aldh1a1 | -0.71 | 0.00679179 | 4.43 | -1.70 |
| mmu-miR-29c-5p | Col15a1 | -0.71 | 0.006831242 | 3.44 | -2.31 |
| mmu-miR-155-5p | Ephx2 | -0.71 | 0.006841315 | 1.93 | -1.99 |
| mmu-miR-194-5p | Elmod1 | -0.71 | 0.006865151 | 4.92 | -1.66 |
| mmu-miR-27a-3p | Slc16a10 | -0.71 | 0.006870103 | 3.17 | -1.94 |
| mmu-miR-200a-3p | St8sia2 | -0.71 | 0.006892941 | 2.61 | -1.66 |
| mmu-miR-106b-5p | Zfp850 | -0.71 | 0.006917921 | 4.02 | -2.34 |
| mmu-miR-205-3p | 6-Sep | -0.71 | 0.00694251 | 6.09 | -1.64 |
| mmu-miR-223-3p | Spag17 | -0.71 | 0.006970416 | 9.15 | -2.77 |
| mmu-let-7f-5p | Ptar1 | -0.71 | 0.006982609 | 3.20 | -3.27 |
| mmu-miR-25-3p | Fbn1 | -0.71 | 0.007011201 | 2.56 | -3.25 |
| mmu-miR-17-5p | Ascl3 | -0.71 | 0.007017404 | 3.83 | -2.07 |
| mmu-miR-214-5p | Spdef | -0.71 | 0.007026085 | 3.66 | -1.94 |
| mmu-miR-181d-5p | Rcbtb2 | -0.71 | 0.007042739 | 4.07 | -1.59 |
| mmu-miR-19b-3p | Sox5 | -0.71 | 0.007067321 | 3.59 | -2.90 |
| mmu-miR-149-3p | Cmklr1 | -0.71 | 0.007069459 | -4.06 | 1.61 |
| mmu-miR-708-5p | Cd34 | -0.71 | 0.00708939 | 2.00 | -3.73 |
| mmu-miR-192-5p | Ccdc103 | -0.71 | 0.007095844 | 5.95 | -2.01 |
| mmu-miR-328-3p | Trim12c | -0.71 | 0.007095919 | -5.59 | 2.09 |
| mmu-miR-132-3p | Foxp2 | -0.71 | 0.007097514 | 2.20 | -2.72 |
| mmu-miR-192-5p | Dync2h1 | -0.71 | 0.007111704 | 5.95 | -1.72 |
| mmu-miR-15a-5p | Dusp28 | -0.71 | 0.007116688 | 2.29 | -1.71 |
| mmu-miR-30a-5p | Actc1 | -0.71 | 0.007150943 | 2.87 | -2.17 |
| mmu-miR-181d-5p | Kifap3 | -0.71 | 0.007177602 | 4.07 | -1.75 |
| mmu-miR-130a-3p | C1s1 | -0.71 | 0.007185937 | 2.29 | -2.40 |
| mmu-miR-26b-5p | Slc16a10 | -0.71 | 0.007211361 | 3.80 | -1.94 |
| mmu-miR-218-5p | Six4 | -0.71 | 0.007213874 | 3.13 | -1.65 |
| mmu-miR-130a-3p | Meig1 | -0.71 | 0.007220533 | 2.29 | -2.50 |
| mmu-miR-26a-5p | Fam45a | -0.71 | 0.007228574 | 2.26 | -1.61 |
| mmu-miR-29c-3p | Sdc2 | -0.71 | 0.007303179 | 5.21 | -2.29 |
| mmu-miR-421-3p | Smyd3 | -0.71 | 0.007336941 | 3.10 | -1.91 |
| mmu-miR-500-3p | Dpt | -0.71 | 0.007366032 | 3.74 | -3.73 |
| mmu-miR-185-5p | Morn3 | -0.71 | 0.007378594 | 2.50 | -2.84 |
| mmu-miR-203-3p | G630090E17Rik | -0.71 | 0.007398577 | 4.73 | -2.14 |
| mmu-miR-433-3p | Map3k6 | -0.71 | 0.007398997 | -4.72 | 1.65 |
| mmu-miR-181c-5p | Dpyd | -0.71 | 0.007407264 | 3.05 | -1.87 |
| mmu-miR-26b-5p | Osbpl6 | -0.71 | 0.00744112 | 3.80 | -1.73 |
| mmu-miR-16-5p | Slc4a8 | -0.71 | 0.007443089 | 1.77 | -1.64 |
| mmu-miR-30c-5p | Tspyl4 | -0.71 | 0.007444932 | 2.26 | -2.33 |
| mmu-miR-141-3p | Fkbp1b | -0.71 | 0.007530603 | 4.66 | -2.34 |
| mmu-miR-500-3p | 6-Sep | -0.71 | 0.007541244 | 3.74 | -1.64 |
| mmu-miR-146a-5p | Cfh | -0.71 | 0.007545559 | 4.19 | -3.58 |
| mmu-miR-32-5p | Foxp2 | -0.71 | 0.007559084 | 4.39 | -2.72 |
| mmu-miR-30c-5p | Spa17 | -0.71 | 0.007568996 | 2.26 | -1.63 |
| mmu-miR-99b-5p | Spdef | -0.71 | 0.007640979 | 1.97 | -1.94 |
| mmu-miR-30c-5p | Six4 | -0.71 | 0.007656543 | 2.26 | -1.65 |
| mmu-miR-218-5p | Ptar1 | -0.71 | 0.007660528 | 3.13 | -3.27 |
| mmu-miR-194-5p | Ulk4 | -0.71 | 0.007682121 | 4.92 | -1.85 |
| mmu-let-7i-5p | Ppargc1a | -0.71 | 0.007684972 | 1.79 | -1.61 |
| mmu-miR-25-3p | Fam92b | -0.70 | 0.007699859 | 2.56 | -2.31 |
| mmu-miR-429-3p | Actc1 | -0.70 | 0.0077019 | 3.03 | -2.17 |
| mmu-miR-34a-5p | Nxt2 | -0.70 | 0.007709719 | 3.60 | -2.84 |
| mmu-miR-26a-5p | Gng11 | -0.70 | 0.007711027 | 2.26 | -2.20 |
| mmu-miR-152-3p | Cetn2 | -0.70 | 0.007712601 | 1.92 | -1.85 |
| mmu-miR-200b-3p | Fkbp1b | -0.70 | 0.007730842 | 2.19 | -2.34 |
| mmu-miR-25-5p | Sox4 | -0.70 | 0.007734378 | -2.35 | 1.76 |
| mmu-miR-31-5p | Dusp28 | -0.70 | 0.007744173 | 4.65 | -1.71 |
| mmu-miR-200b-3p | Rpgr | -0.70 | 0.007746062 | 2.19 | -2.01 |
| mmu-miR-214-5p | Dnajb13 | -0.70 | 0.007750005 | 3.66 | -1.82 |
| mmu-miR-181c-5p | Bche | -0.70 | 0.007790038 | 3.05 | -2.78 |
| mmu-miR-32-5p | Casd1 | -0.70 | 0.007803362 | 4.39 | -2.06 |
| mmu-miR-155-5p | Gpm6b | -0.70 | 0.007835274 | 1.93 | -1.96 |
| mmu-miR-193b-3p | Plekho1 | -0.70 | 0.007873093 | -2.01 | 1.91 |
| mmu-miR-98-5p | Slc16a10 | -0.70 | 0.007876342 | 3.72 | -1.94 |
| mmu-let-7f-5p | Ccdc81 | -0.70 | 0.007903111 | 3.20 | -1.59 |
| mmu-let-7g-5p | Ptar1 | -0.70 | 0.007968691 | 2.05 | -3.27 |
| mmu-miR-410-3p | Rpgr | -0.70 | 0.007978439 | 3.62 | -2.01 |
| mmu-miR-26b-5p | Fam45a | -0.70 | 0.007992859 | 3.80 | -1.61 |
| mmu-miR-30b-5p | Actc1 | -0.70 | 0.007995424 | 2.59 | -2.17 |
| mmu-miR-205-3p | Rora | -0.70 | 0.00800146 | 6.09 | -2.00 |
| mmu-miR-128-3p | Rpgrip1l | -0.70 | 0.008002612 | 2.80 | -1.64 |
| mmu-miR-148b-3p | Cadm1 | -0.70 | 0.00802619 | 4.32 | -1.72 |
| mmu-miR-128-3p | Pkia | -0.70 | 0.00804486 | 2.80 | -2.03 |
| mmu-miR-214-5p | Atp2a3 | -0.70 | 0.008060521 | 3.66 | -3.03 |
| mmu-miR-218-5p | Sema3b | -0.70 | 0.008061147 | 3.13 | -1.61 |
| mmu-miR-92b-5p | Slx4 | -0.70 | 0.008136288 | -6.76 | 1.68 |
| mmu-miR-135b-5p | Six4 | -0.70 | 0.008154175 | 4.89 | -1.65 |
| mmu-miR-29c-3p | Nme5 | -0.70 | 0.008161811 | 5.21 | -2.53 |
| mmu-miR-141-3p | Ppp2r2b | -0.70 | 0.008190746 | 4.66 | -1.83 |
| mmu-miR-183-3p | Ttll9 | -0.70 | 0.008201374 | 4.74 | -1.73 |
| mmu-miR-32-5p | Crls1 | -0.70 | 0.008207107 | 4.39 | -1.62 |
| mmu-miR-340-5p | Lrrc34 | -0.70 | 0.008264513 | 9.01 | -2.14 |
| mmu-miR-130a-3p | Fam45a | -0.70 | 0.008281379 | 2.29 | -1.61 |
| mmu-miR-212-3p | Nov | -0.70 | 0.00831421 | 3.43 | -2.34 |
| mmu-miR-218-5p | Col1a1 | -0.70 | 0.008332598 | 3.13 | -1.71 |
| mmu-let-7f-5p | Slc16a10 | -0.70 | 0.008365013 | 3.20 | -1.94 |
| mmu-let-7b-5p | Ptafr | -0.70 | 0.008376883 | -3.08 | 4.38 |
| mmu-miR-214-3p | Ccl4 | -0.70 | 0.00839251 | -1.90 | 4.77 |
| mmu-miR-194-5p | Arhgap5 | -0.70 | 0.008405171 | 4.92 | -1.85 |
| mmu-miR-205-3p | Axin2 | -0.70 | 0.00841766 | 6.09 | -1.66 |
| mmu-miR-24-3p | Lipt2 | -0.70 | 0.0084418 | 1.73 | -1.62 |
| mmu-miR-328-3p | Sh3bp2 | -0.70 | 0.008444947 | -5.59 | 2.98 |
| mmu-miR-212-3p | Pfn2 | -0.70 | 0.008522971 | 3.43 | -1.75 |
| mmu-miR-192-5p | Dnah5 | -0.70 | 0.008546601 | 5.95 | -2.51 |
| mmu-miR-328-3p | Csf2ra | -0.70 | 0.008559273 | -5.59 | 1.64 |
| mmu-miR-214-3p | Tnfrsf10b | -0.70 | 0.00856529 | -1.90 | 2.36 |
| mmu-miR-340-5p | Rpgr | -0.70 | 0.008569229 | 9.01 | -2.01 |
| mmu-miR-148a-5p | Kit | -0.70 | 0.00859303 | 2.62 | -1.91 |
| mmu-miR-504-5p | Sh2d3c | -0.70 | 0.008634825 | -4.12 | 1.76 |
| mmu-miR-185-5p | Acot1 | -0.70 | 0.008656219 | 2.50 | -2.12 |
| mmu-miR-185-5p | Acot1 | -0.70 | 0.008656219 | 2.50 | -2.12 |
| mmu-miR-182-5p | Sox5 | -0.70 | 0.008676467 | 3.32 | -2.90 |
| mmu-miR-23b-3p | Cybrd1 | -0.70 | 0.00868114 | 2.62 | -2.98 |
| mmu-miR-29a-3p | Fbn1 | -0.70 | 0.008694221 | 2.71 | -3.25 |
| mmu-miR-421-3p | Zfp474 | -0.70 | 0.008704501 | 3.10 | -2.17 |
| mmu-miR-135b-5p | Atp6v1c2 | -0.70 | 0.008712631 | 4.89 | -2.75 |
| mmu-miR-218-5p | Setbp1 | -0.70 | 0.008713891 | 3.13 | -1.63 |
| mmu-miR-410-3p | Col15a1 | -0.70 | 0.008724797 | 3.62 | -2.31 |
| mmu-miR-10a-5p | Fgfr1 | -0.70 | 0.00875717 | 2.87 | -1.71 |
| mmu-miR-425-5p | Dync2h1 | -0.70 | 0.00879815 | 1.93 | -1.72 |
| mmu-miR-29c-3p | Gpx7 | -0.69 | 0.008808199 | 5.21 | -2.02 |
| mmu-miR-29c-3p | Dusp28 | -0.69 | 0.008877201 | 5.21 | -1.71 |
| mmu-miR-183-5p | Ccdc121 | -0.69 | 0.00888495 | 2.64 | -1.71 |
| mmu-miR-214-3p | Pim1 | -0.69 | 0.008899067 | -1.90 | 1.62 |
| mmu-let-7g-5p | Slc16a10 | -0.69 | 0.008906754 | 2.05 | -1.94 |
| mmu-miR-148b-3p | Cacna2d1 | -0.69 | 0.008909611 | 4.32 | -4.55 |
| mmu-miR-17-5p | Fam45a | -0.69 | 0.008911736 | 3.83 | -1.61 |
| mmu-miR-29c-5p | Eln | -0.69 | 0.008914531 | 3.44 | -2.85 |
| mmu-miR-194-5p | Cntnap2 | -0.69 | 0.008921891 | 4.92 | -2.56 |
| mmu-miR-30c-5p | Slc25a34 | -0.69 | 0.00893199 | 2.26 | -1.68 |
| mmu-miR-135b-5p | Ghr | -0.69 | 0.008936627 | 4.89 | -2.23 |
| mmu-miR-29a-3p | Col4a6 | -0.69 | 0.008940119 | 2.71 | -1.94 |
| mmu-miR-152-3p | Wdr19 | -0.69 | 0.008945966 | 1.92 | -1.81 |
| mmu-let-7f-5p | Zfp474 | -0.69 | 0.00895328 | 3.20 | -2.17 |
| mmu-miR-340-5p | Rragd | -0.69 | 0.008996746 | 9.01 | -2.21 |
| mmu-miR-130a-3p | Six4 | -0.69 | 0.009002484 | 2.29 | -1.65 |
| mmu-miR-98-5p | Rragd | -0.69 | 0.009008779 | 3.72 | -2.21 |
| mmu-miR-433-3p | Slco1a5 | -0.69 | 0.009017281 | -4.72 | 2.46 |
| mmu-miR-135b-5p | Dtna | -0.69 | 0.009028305 | 4.89 | -1.62 |
| mmu-miR-210-5p | G0s2 | -0.69 | 0.009059962 | -3.25 | 3.68 |
| mmu-miR-193b-3p | Nbeal2 | -0.69 | 0.009071751 | -2.01 | 2.56 |
| mmu-miR-193b-3p | Cebpb | -0.69 | 0.009109044 | -2.01 | 1.62 |
| mmu-miR-185-5p | Loxl1 | -0.69 | 0.00911816 | 2.50 | -2.18 |
| mmu-miR-210-3p | Armc4 | -0.69 | 0.009129154 | 1.86 | -3.02 |
| mmu-miR-200a-3p | Col4a6 | -0.69 | 0.009135529 | 2.61 | -1.94 |
| mmu-miR-20a-5p | Fam45a | -0.69 | 0.009148486 | 3.70 | -1.61 |
| mmu-miR-135b-5p | Eya1 | -0.69 | 0.009179215 | 4.89 | -1.69 |
| mmu-miR-29c-5p | Col3a1 | -0.69 | 0.009240247 | 3.44 | -2.61 |
| mmu-miR-210-3p | Tsnaxip1 | -0.69 | 0.009261984 | 1.86 | -1.85 |
| mmu-miR-326-3p | Dok2 | -0.69 | 0.009288557 | -1.76 | 2.80 |
| mmu-miR-223-3p | Fbxo16 | -0.69 | 0.009295137 | 9.15 | -2.41 |
| mmu-miR-144-5p | Mlf1 | -0.69 | 0.009300952 | 5.17 | -2.32 |
| mmu-miR-326-3p | Fscn1 | -0.69 | 0.009313136 | -1.76 | 2.28 |
| mmu-miR-181d-5p | Eln | -0.69 | 0.009313239 | 4.07 | -2.85 |
| mmu-miR-182-5p | Osbpl6 | -0.69 | 0.009317763 | 3.32 | -1.73 |
| mmu-miR-148b-3p | Slc25a23 | -0.69 | 0.009343077 | 4.32 | -2.24 |
| mmu-miR-433-3p | Trim30a | -0.69 | 0.009383943 | -4.72 | 2.33 |
| mmu-miR-135b-5p | Ramp2 | -0.69 | 0.009391948 | 4.89 | -2.72 |
| mmu-miR-130a-3p | Nov | -0.69 | 0.009396171 | 2.29 | -2.34 |
| mmu-miR-29c-5p | Fstl1 | -0.69 | 0.009412619 | 3.44 | -2.02 |
| mmu-let-7b-5p | 9430015G10Rik | -0.69 | 0.009427363 | -3.08 | 1.93 |
| mmu-miR-200a-3p | Fkbp1b | -0.69 | 0.009429489 | 2.61 | -2.34 |
| mmu-miR-760-3p | Sh3bp2 | -0.69 | 0.009435096 | -7.15 | 2.98 |
| mmu-miR-193b-3p | Plaur | -0.69 | 0.009443245 | -2.01 | 2.60 |
| mmu-miR-141-3p | Pfkm | -0.69 | 0.009488858 | 4.66 | -1.75 |
| mmu-miR-128-3p | Scd1 | -0.69 | 0.009496283 | 2.80 | -1.90 |
| mmu-miR-152-3p | Pxmp2 | -0.69 | 0.009508469 | 1.92 | -3.43 |
| mmu-miR-411-5p | Slc16a7 | -0.69 | 0.00955467 | 4.43 | -3.51 |
| mmu-miR-27b-3p | Ccdc89 | -0.69 | 0.009569237 | 2.45 | -1.73 |
| mmu-miR-29c-3p | Slc4a8 | -0.69 | 0.009585183 | 5.21 | -1.64 |
| mmu-miR-135b-5p | Dynlrb2 | -0.69 | 0.00958954 | 4.89 | -2.96 |
| mmu-miR-421-3p | Elmod1 | -0.69 | 0.009591158 | 3.10 | -1.66 |
| mmu-let-7g-5p | Ccdc81 | -0.69 | 0.009591343 | 2.05 | -1.59 |
| mmu-miR-340-5p | Fbn1 | -0.69 | 0.009617104 | 9.01 | -3.25 |
| mmu-miR-210-3p | Chad | -0.69 | 0.009623149 | 1.86 | -6.22 |
| mmu-miR-193b-3p | Phlda2 | -0.69 | 0.009629079 | -2.01 | 2.34 |
| mmu-miR-25-5p | Has3 | -0.69 | 0.009674763 | -2.35 | 1.63 |
| mmu-miR-425-5p | Fbn1 | -0.69 | 0.009691261 | 1.93 | -3.25 |
| mmu-miR-183-3p | Ric3 | -0.69 | 0.009708607 | 4.74 | -2.30 |
| mmu-miR-148b-3p | Dnah11 | -0.69 | 0.009730723 | 4.32 | -1.85 |
| mmu-miR-30c-5p | Ccdc60 | -0.69 | 0.009744924 | 2.26 | -1.61 |
| mmu-miR-181d-5p | Calm2 | -0.69 | 0.009751369 | 4.07 | -1.70 |
| mmu-miR-25-3p | Ccdc89 | -0.69 | 0.009775195 | 2.56 | -1.73 |
| mmu-miR-192-5p | Arhgap29 | -0.69 | 0.009818114 | 5.95 | -2.19 |
| mmu-miR-99b-5p | Kifap3 | -0.69 | 0.009849066 | 1.97 | -1.75 |
| mmu-miR-708-5p | Rpgrip1l | -0.69 | 0.009872624 | 2.00 | -1.64 |
| mmu-miR-93-5p | Ascl3 | -0.69 | 0.009891538 | 2.30 | -2.07 |
| mmu-miR-185-5p | Lclat1 | -0.69 | 0.009895025 | 2.50 | -1.84 |
| mmu-miR-212-3p | Col1a1 | -0.69 | 0.009922582 | 3.43 | -1.71 |
| mmu-let-7f-5p | Lrguk | -0.69 | 0.009938748 | 3.20 | -2.08 |
| mmu-miR-16-5p | Fkbp1b | -0.69 | 0.00994499 | 1.77 | -2.34 |
| mmu-miR-200a-3p | Ppp2r2b | -0.68 | 0.010035268 | 2.61 | -1.83 |
| mmu-miR-29a-3p | Osbpl6 | -0.68 | 0.01006044 | 2.71 | -1.73 |
| mmu-miR-29c-5p | Dcun1d1 | -0.68 | 0.010091704 | 3.44 | -1.64 |
| mmu-miR-500-3p | Dmbt1 | -0.68 | 0.010095245 | 3.74 | -3.59 |
| mmu-miR-214-3p | Csf1 | -0.68 | 0.010125159 | -1.90 | 2.24 |
| mmu-miR-185-3p | 9430015G10Rik | -0.68 | 0.010139244 | -3.01 | 1.93 |
| mmu-miR-32-5p | Cdkn1c | -0.68 | 0.010162128 | 4.39 | -2.19 |
| mmu-miR-155-5p | Cfh | -0.68 | 0.010165931 | 1.93 | -3.58 |
| mmu-miR-181d-5p | Igf2bp2 | -0.68 | 0.010170138 | 4.07 | -2.19 |
| mmu-miR-27a-5p | Ccdc89 | -0.68 | 0.010179078 | 4.90 | -1.73 |
| mmu-miR-205-3p | Bmpr1b | -0.68 | 0.010201882 | 6.09 | -2.84 |
| mmu-miR-23b-3p | Ppargc1a | -0.68 | 0.010207473 | 2.62 | -1.61 |
| mmu-miR-185-5p | Fbxo36 | -0.68 | 0.010336378 | 2.50 | -1.72 |
| mmu-miR-182-5p | Fbn1 | -0.68 | 0.010349224 | 3.32 | -3.25 |
| mmu-miR-212-3p | Msh2 | -0.68 | 0.010354254 | 3.43 | -1.86 |
| mmu-miR-676-5p | B3galt2 | -0.68 | 0.010409162 | 2.82 | -1.66 |
| mmu-miR-410-3p | Rora | -0.68 | 0.010436685 | 3.62 | -2.00 |
| mmu-miR-152-3p | Dlec1 | -0.68 | 0.010437259 | 1.92 | -1.65 |
| mmu-miR-182-5p | Fam45a | -0.68 | 0.010438644 | 3.32 | -1.61 |
| mmu-miR-16-5p | Foxp2 | -0.68 | 0.010447808 | 1.77 | -2.72 |
| mmu-miR-411-5p | Spag16 | -0.68 | 0.010470663 | 4.43 | -3.82 |
| mmu-miR-99a-5p | 8430408G22Rik | -0.68 | 0.010506828 | 3.47 | -2.53 |
| mmu-miR-429-3p | Ppp2r2b | -0.68 | 0.010541958 | 3.03 | -1.83 |
| mmu-miR-10a-5p | Dmbt1 | -0.68 | 0.010560657 | 2.87 | -3.59 |
| mmu-miR-504-5p | Lif | -0.68 | 0.010589841 | -4.12 | 2.93 |
| mmu-miR-218-5p | Dnah7b | -0.68 | 0.010594264 | 3.13 | -1.97 |
| mmu-miR-375-3p | Trim15 | -0.68 | 0.010599645 | -2.94 | 2.14 |
| mmu-miR-143-3p | Fmo5 | -0.68 | 0.010609927 | 2.79 | -1.61 |
| mmu-miR-210-3p | Rabgap1l | -0.68 | 0.010616284 | 1.86 | -2.04 |
| mmu-miR-26b-5p | Mrpl1 | -0.68 | 0.010622912 | 3.80 | -2.74 |
| mmu-miR-148a-3p | Cadm1 | -0.68 | 0.010668052 | 2.94 | -1.72 |
| mmu-miR-410-3p | Fam45a | -0.68 | 0.010724865 | 3.62 | -1.61 |
| mmu-miR-24-3p | Dmbt1 | -0.68 | 0.010793919 | 1.73 | -3.59 |
| mmu-miR-130a-3p | Dnaic1 | -0.68 | 0.010795251 | 2.29 | -1.71 |
| mmu-miR-152-3p | Galm | -0.68 | 0.010851459 | 1.92 | -2.36 |
| mmu-miR-32-5p | Tbc1d30 | -0.68 | 0.010891331 | 4.39 | -2.52 |
| mmu-miR-132-3p | Six4 | -0.68 | 0.010926598 | 2.20 | -1.65 |
| mmu-miR-135b-5p | Gpr155 | -0.68 | 0.010929352 | 4.89 | -2.57 |
| mmu-miR-194-5p | Kitl | -0.68 | 0.010945028 | 4.92 | -2.33 |
| mmu-miR-148b-3p | Sms | -0.68 | 0.010956584 | 4.32 | -2.09 |
| mmu-miR-19b-3p | Osbpl6 | -0.68 | 0.010970238 | 3.59 | -1.73 |
| mmu-miR-27a-3p | Gpam | -0.68 | 0.010977699 | 3.17 | -1.76 |
| mmu-miR-193b-3p | Trim30a | -0.68 | 0.010987319 | -2.01 | 2.33 |
| mmu-miR-135b-5p | Fbxo36 | -0.68 | 0.010995509 | 4.89 | -1.72 |
| mmu-miR-375-3p | Clcn2 | -0.68 | 0.011004341 | -2.94 | 1.73 |
| mmu-miR-148a-3p | Atp1a2 | -0.68 | 0.011006001 | 2.94 | -4.08 |
| mmu-miR-183-3p | Tspyl4 | -0.68 | 0.011021332 | 4.74 | -2.33 |
| mmu-miR-326-3p | Faim2 | -0.68 | 0.011023214 | -1.76 | 1.65 |
| mmu-miR-16-5p | Casc1 | -0.68 | 0.011023614 | 1.77 | -1.66 |
| mmu-miR-16-5p | 6820408C15Rik | -0.68 | 0.011035975 | 1.77 | -2.61 |
| mmu-let-7g-5p | Rragd | -0.68 | 0.011037212 | 2.05 | -2.21 |
| mmu-miR-130a-3p | Kit | -0.68 | 0.011040847 | 2.29 | -1.91 |
| mmu-miR-135b-5p | Kcnrg | -0.68 | 0.011047774 | 4.89 | -2.07 |
| mmu-miR-744-5p | Dok3 | -0.68 | 0.011097525 | -3.34 | 3.00 |
| mmu-miR-29c-3p | Kcnrg | -0.68 | 0.011128142 | 5.21 | -2.07 |
| mmu-miR-193b-3p | Gdf15 | -0.68 | 0.011238793 | -2.01 | 5.48 |
| mmu-miR-205-3p | Armc4 | -0.68 | 0.011242529 | 6.09 | -3.02 |
| mmu-miR-92b-5p | Cxcr4 | -0.68 | 0.011255931 | -6.76 | 1.97 |
| mmu-miR-214-3p | Phlda3 | -0.68 | 0.011259169 | -1.90 | 2.39 |
| mmu-miR-218-5p | Gfra1 | -0.68 | 0.0112717 | 3.13 | -3.67 |
| mmu-miR-205-3p | Dnah9 | -0.68 | 0.011277791 | 6.09 | -2.00 |
| mmu-miR-143-3p | Adhfe1 | -0.68 | 0.011299629 | 2.79 | -2.39 |
| mmu-miR-29a-3p | Zfp791 | -0.68 | 0.011322868 | 2.71 | -2.85 |
| mmu-miR-130a-3p | Sox5 | -0.67 | 0.011359427 | 2.29 | -2.90 |
| mmu-miR-500-3p | Gpx7 | -0.67 | 0.011443882 | 3.74 | -2.02 |
| mmu-miR-144-5p | Arhgap5 | -0.67 | 0.011456468 | 5.17 | -1.85 |
| mmu-miR-155-5p | Gfra1 | -0.67 | 0.011470004 | 1.93 | -3.67 |
| mmu-miR-194-5p | Pfn2 | -0.67 | 0.011541362 | 4.92 | -1.75 |
| mmu-miR-29c-3p | Aqp1 | -0.67 | 0.011559784 | 5.21 | -1.87 |
| mmu-miR-205-3p | Gfra1 | -0.67 | 0.011595774 | 6.09 | -3.67 |
| mmu-miR-218-5p | Bmpr1b | -0.67 | 0.011674297 | 3.13 | -2.84 |
| mmu-miR-326-3p | Ccrl2 | -0.67 | 0.011702822 | -1.76 | 2.47 |
| mmu-miR-411-5p | Lrrc34 | -0.67 | 0.011745057 | 4.43 | -2.14 |
| mmu-miR-23a-3p | Aldh1a2 | -0.67 | 0.011775859 | 3.01 | -3.23 |
| mmu-miR-99a-5p | Sez6l2 | -0.67 | 0.011795711 | 3.47 | -3.64 |
| mmu-miR-144-5p | Tacr1 | -0.67 | 0.011806441 | 5.17 | -5.28 |
| mmu-miR-411-5p | Arhgap5 | -0.67 | 0.011826282 | 4.43 | -1.85 |
| mmu-miR-429-3p | Rpgr | -0.67 | 0.011861608 | 3.03 | -2.01 |
| mmu-miR-152-3p | Sox5 | -0.67 | 0.011880234 | 1.92 | -2.90 |
| mmu-miR-141-3p | Sox5 | -0.67 | 0.011892191 | 4.66 | -2.90 |
| mmu-miR-99b-5p | 8430408G22Rik | -0.67 | 0.011926774 | 1.97 | -2.53 |
| mmu-miR-300-3p | Fgf1 | -0.67 | 0.011941097 | 2.34 | -1.67 |
| mmu-miR-429-3p | Scd1 | -0.67 | 0.011984359 | 3.03 | -1.90 |
| mmu-miR-152-3p | Rora | -0.67 | 0.011989905 | 1.92 | -2.00 |
| mmu-miR-98-5p | Col1a1 | -0.67 | 0.012051251 | 3.72 | -1.71 |
| mmu-miR-212-3p | Kitl | -0.67 | 0.012109765 | 3.43 | -2.33 |
| mmu-miR-411-5p | Hp | -0.67 | 0.012110821 | 4.43 | -1.65 |
| mmu-miR-183-3p | Spag17 | -0.67 | 0.012116771 | 4.74 | -2.77 |
| mmu-miR-99a-5p | Dtna | -0.67 | 0.012120144 | 3.47 | -1.62 |
| mmu-miR-29c-3p | Efhc1 | -0.67 | 0.012151207 | 5.21 | -2.17 |
| mmu-miR-326-3p | Irf7 | -0.67 | 0.012158529 | -1.76 | 1.81 |
| mmu-miR-194-5p | Ccdc103 | -0.67 | 0.012172912 | 4.92 | -2.01 |
| mmu-let-7f-5p | Rragd | -0.67 | 0.012217069 | 3.20 | -2.21 |
| mmu-miR-132-3p | Rfx3 | -0.67 | 0.012236603 | 2.20 | -2.30 |
| mmu-miR-15a-5p | 6820408C15Rik | -0.67 | 0.012278231 | 2.29 | -2.61 |
| mmu-miR-223-3p | Fkbp1b | -0.67 | 0.012287801 | 9.15 | -2.34 |
| mmu-miR-93-3p | Zfp791 | -0.67 | 0.012292105 | 4.39 | -2.85 |
| mmu-miR-149-3p | Dmrta2 | -0.67 | 0.012301041 | -4.06 | 1.77 |
| mmu-let-7i-5p | Col1a1 | -0.67 | 0.012327268 | 1.79 | -1.71 |
| mmu-miR-411-5p | Abi3bp | -0.67 | 0.012366107 | 4.43 | -3.56 |
| mmu-miR-32-5p | B3galt2 | -0.67 | 0.012378189 | 4.39 | -1.66 |
| mmu-miR-99b-5p | Spag6 | -0.67 | 0.012421898 | 1.97 | -2.67 |
| mmu-miR-181a-5p | Slc26a4 | -0.67 | 0.012488829 | -3.01 | 2.61 |
| mmu-miR-30e-5p | Acyp2 | -0.67 | 0.012505274 | 3.60 | -2.40 |
| mmu-miR-26a-5p | Mrpl1 | -0.67 | 0.012516339 | 2.26 | -2.74 |
| mmu-miR-203-3p | Fbxo16 | -0.67 | 0.012576168 | 4.73 | -2.41 |
| mmu-miR-135b-5p | Sec14l4 | -0.67 | 0.012582162 | 4.89 | -2.09 |
| mmu-miR-192-5p | Bmpr1b | -0.67 | 0.012594258 | 5.95 | -2.84 |
| mmu-miR-148a-3p | Crls1 | -0.67 | 0.012680229 | 2.94 | -1.62 |
| mmu-miR-375-3p | Fgr | -0.67 | 0.012727151 | -2.94 | 2.06 |
| mmu-miR-300-3p | Zbtb33 | -0.67 | 0.012760098 | 2.34 | -1.68 |
| mmu-miR-143-3p | Decr1 | -0.67 | 0.01276727 | 2.79 | -1.64 |
| mmu-miR-192-5p | Cfh | -0.66 | 0.012801957 | 5.95 | -3.58 |
| mmu-miR-429-3p | Nkd1 | -0.66 | 0.012832011 | 3.03 | -2.32 |
| mmu-miR-210-5p | F7 | -0.66 | 0.012834141 | -3.25 | 2.64 |
| mmu-miR-135b-5p | Crls1 | -0.66 | 0.012899968 | 4.89 | -1.62 |
| mmu-miR-328-3p | Tnfrsf10b | -0.66 | 0.012906082 | -5.59 | 2.36 |
| mmu-miR-152-3p | Dnah11 | -0.66 | 0.012917191 | 1.92 | -1.85 |
| mmu-miR-135b-5p | Spag17 | -0.66 | 0.012929152 | 4.89 | -2.77 |
| mmu-miR-32-5p | Kcnmb2 | -0.66 | 0.01302548 | 4.39 | -2.63 |
| mmu-miR-148b-3p | Adcy2 | -0.66 | 0.013114069 | 4.32 | -2.55 |
| mmu-miR-146a-5p | Pm20d2 | -0.66 | 0.013150253 | 4.19 | -2.00 |
| mmu-miR-15a-5p | Fgfr1 | -0.66 | 0.013175524 | 2.29 | -1.71 |
| mmu-miR-192-5p | Msrb2 | -0.66 | 0.013218872 | 5.95 | -2.49 |
| mmu-miR-29a-3p | Gpam | -0.66 | 0.013251284 | 2.71 | -1.76 |
| mmu-miR-99b-5p | Ttc30a1 | -0.66 | 0.01325644 | 1.97 | -2.40 |
| mmu-miR-214-5p | Cadm1 | -0.66 | 0.013328098 | 3.66 | -1.72 |
| mmu-miR-99a-5p | Ttc30a1 | -0.66 | 0.013375298 | 3.47 | -2.40 |
| mmu-miR-135b-5p | Dnah1 | -0.66 | 0.013382724 | 4.89 | -1.82 |
| mmu-miR-326-3p | Csrnp1 | -0.66 | 0.01338406 | -1.76 | 2.09 |
| mmu-miR-877-3p | Col27a1 | -0.66 | 0.013410904 | -4.18 | 3.31 |
| mmu-miR-148b-3p | Pxmp2 | -0.66 | 0.013452382 | 4.32 | -3.43 |
| mmu-miR-135b-5p | Slc4a8 | -0.66 | 0.013502526 | 4.89 | -1.64 |
| mmu-let-7b-5p | Cd33 | -0.66 | 0.013558356 | -3.08 | 1.91 |
| mmu-miR-326-3p | Itga5 | -0.66 | 0.01356065 | -1.76 | 2.47 |
| mmu-miR-34a-5p | Fbxo16 | -0.66 | 0.013611583 | 3.60 | -2.41 |
| mmu-miR-504-5p | Col7a1 | -0.66 | 0.013707025 | -4.12 | 3.75 |
| mmu-miR-183-3p | 6820408C15Rik | -0.66 | 0.013711134 | 4.74 | -2.61 |
| mmu-miR-210-5p | Tnip3 | -0.66 | 0.013740706 | -3.25 | 3.56 |
| mmu-miR-708-5p | Srpx2 | -0.66 | 0.013744432 | 2.00 | -2.81 |
| mmu-miR-504-5p | Hk3 | -0.66 | 0.013752229 | -4.12 | 4.21 |
| mmu-miR-30e-5p | Ak7 | -0.66 | 0.01379672 | 3.60 | -1.79 |
| mmu-miR-152-3p | Grb14 | -0.66 | 0.013844433 | 1.92 | -1.73 |
| mmu-miR-27b-3p | Gpam | -0.66 | 0.013854281 | 2.45 | -1.76 |
| mmu-miR-182-5p | Nxt2 | -0.66 | 0.013855664 | 3.32 | -2.84 |
| mmu-miR-19b-3p | Chek2 | -0.66 | 0.013905432 | 3.59 | -1.80 |
| mmu-miR-17-5p | Zfp791 | -0.66 | 0.013934042 | 3.83 | -2.85 |
| mmu-miR-92b-3p | Tnip3 | -0.66 | 0.014024355 | -4.78 | 3.56 |
| mmu-miR-106b-5p | Cybrd1 | -0.66 | 0.014080801 | 4.02 | -2.98 |
| mmu-miR-27a-3p | Tpsb2 | -0.66 | 0.0140992 | 3.17 | -2.56 |
| mmu-miR-194-5p | Cacna2d1 | -0.66 | 0.014101176 | 4.92 | -4.55 |
| mmu-miR-30c-5p | Scara5 | -0.66 | 0.014121357 | 2.26 | -2.63 |
| mmu-miR-135b-5p | Meig1 | -0.66 | 0.014167635 | 4.89 | -2.50 |
| mmu-miR-194-5p | B4galt4 | -0.66 | 0.014178934 | 4.92 | -2.19 |
| mmu-miR-200a-3p | Gpm6b | -0.66 | 0.014250668 | 2.61 | -1.96 |
| mmu-miR-181c-5p | Scd1 | -0.66 | 0.014280207 | 3.05 | -1.90 |
| mmu-miR-23a-3p | Zfp791 | -0.66 | 0.014298602 | 3.01 | -2.85 |
| mmu-miR-29c-3p | Smtnl2 | -0.66 | 0.014322075 | 5.21 | -2.05 |
| mmu-miR-708-5p | Dysf | -0.65 | 0.014429877 | 2.00 | -2.88 |
| mmu-miR-144-5p | Tnfrsf19 | -0.65 | 0.014475726 | 5.17 | -3.09 |
| mmu-miR-92b-5p | Lrrc25 | -0.65 | 0.014520682 | -6.76 | 2.40 |
| mmu-miR-92b-5p | Slc16a6 | -0.65 | 0.014538751 | -6.76 | 3.02 |
| mmu-miR-152-3p | Adcy2 | -0.65 | 0.014550265 | 1.92 | -2.55 |
| mmu-miR-340-5p | Osbpl6 | -0.65 | 0.014561414 | 9.01 | -1.73 |
| mmu-miR-221-3p | Gnb3 | -0.65 | 0.014609446 | 2.18 | -1.89 |
| mmu-miR-141-3p | Osbpl6 | -0.65 | 0.014626824 | 4.66 | -1.73 |
| mmu-miR-205-3p | Armc3 | -0.65 | 0.014651544 | 6.09 | -2.25 |
| mmu-miR-144-5p | Kcnmb2 | -0.65 | 0.014678442 | 5.17 | -2.63 |
| mmu-miR-212-3p | Pam | -0.65 | 0.014848858 | 3.43 | -1.83 |
| mmu-miR-212-3p | Arhgap5 | -0.65 | 0.014910918 | 3.43 | -1.85 |
| mmu-miR-17-5p | Plscr4 | -0.65 | 0.014916898 | 3.83 | -2.48 |
| mmu-miR-26b-5p | Fbxo16 | -0.65 | 0.014930491 | 3.80 | -2.41 |
| mmu-miR-130a-3p | Ttll9 | -0.65 | 0.014941607 | 2.29 | -1.73 |
| mmu-miR-340-5p | Plscr4 | -0.65 | 0.014993339 | 9.01 | -2.48 |
| mmu-miR-130a-3p | Zfp850 | -0.65 | 0.015005984 | 2.29 | -2.34 |
| mmu-miR-144-5p | Rab4a | -0.65 | 0.015011958 | 5.17 | -1.73 |
| mmu-miR-218-5p | Dnah6 | -0.65 | 0.015019359 | 3.13 | -1.93 |
| mmu-miR-410-3p | Wnt5a | -0.65 | 0.015043629 | 3.62 | -2.52 |
| mmu-miR-23a-3p | Fbn1 | -0.65 | 0.015071352 | 3.01 | -3.25 |
| mmu-miR-214-3p | Trem1 | -0.65 | 0.015212532 | -1.90 | 4.63 |
| mmu-miR-148b-3p | Msh2 | -0.65 | 0.015252921 | 4.32 | -1.86 |
| mmu-miR-214-5p | 1110017D15Rik | -0.65 | 0.01527091 | 3.66 | -2.15 |
| mmu-miR-212-3p | Crls1 | -0.65 | 0.015318185 | 3.43 | -1.62 |
| mmu-miR-411-5p | Mettl7a1 | -0.65 | 0.015327033 | 4.43 | -1.71 |
| mmu-miR-200b-3p | Nkd1 | -0.65 | 0.015348109 | 2.19 | -2.32 |
| mmu-miR-135b-5p | Adh1 | -0.65 | 0.01535944 | 4.89 | -2.15 |
| mmu-miR-410-3p | Rfx3 | -0.65 | 0.015468813 | 3.62 | -2.30 |
| mmu-miR-181d-5p | Akap6 | -0.65 | 0.015510207 | 4.07 | -2.33 |
| mmu-miR-16-5p | Actc1 | -0.65 | 0.015529482 | 1.77 | -2.17 |
| mmu-miR-143-3p | Fbxo16 | -0.65 | 0.0156775 | 2.79 | -2.41 |
| mmu-let-7i-5p | Lipt2 | -0.65 | 0.015682848 | 1.79 | -1.62 |
| mmu-miR-210-3p | 1700084C01Rik | -0.65 | 0.01571169 | 1.86 | -3.29 |
| mmu-miR-15a-5p | Actc1 | -0.65 | 0.015836189 | 2.29 | -2.17 |
| mmu-miR-98-5p | Scd1 | -0.65 | 0.015844813 | 3.72 | -1.90 |
| mmu-miR-194-5p | Wdr19 | -0.65 | 0.015862585 | 4.92 | -1.81 |
| mmu-miR-152-3p | Cdkl4 | -0.65 | 0.015872353 | 1.92 | -2.06 |
| mmu-miR-99b-5p | Cd248 | -0.65 | 0.015942419 | 1.97 | -4.04 |
| mmu-miR-203-3p | Papss2 | -0.65 | 0.015942869 | 4.73 | -1.73 |
| mmu-miR-30c-5p | Actc1 | -0.65 | 0.015956415 | 2.26 | -2.17 |
| mmu-miR-181d-5p | Cntnap2 | -0.65 | 0.01596451 | 4.07 | -2.56 |
| mmu-miR-326-3p | Rassf1 | -0.65 | 0.015995281 | -1.76 | 1.62 |
| mmu-miR-143-3p | Scara5 | -0.65 | 0.016013506 | 2.79 | -2.63 |
| mmu-miR-181d-5p | Sema3c | -0.64 | 0.016113383 | 4.07 | -1.90 |
| mmu-miR-210-3p | Cdkn1c | -0.64 | 0.016145691 | 1.86 | -2.19 |
| mmu-miR-345-3p | Rabgap1l | -0.64 | 0.016232706 | 2.14 | -2.04 |
| mmu-miR-93-3p | Fam45a | -0.64 | 0.016250321 | 4.39 | -1.61 |
| mmu-miR-148a-3p | Sox5 | -0.64 | 0.016254779 | 2.94 | -2.90 |
| mmu-miR-32-5p | Fbn1 | -0.64 | 0.016268682 | 4.39 | -3.25 |
| mmu-miR-106b-5p | Fam45a | -0.64 | 0.016387225 | 4.02 | -1.61 |
| mmu-miR-23a-3p | Cybrd1 | -0.64 | 0.016412222 | 3.01 | -2.98 |
| mmu-let-7f-5p | Col1a1 | -0.64 | 0.016481606 | 3.20 | -1.71 |
| mmu-miR-410-3p | Slc16a7 | -0.64 | 0.016511123 | 3.62 | -3.51 |
| mmu-miR-185-5p | Actc1 | -0.64 | 0.01651479 | 2.50 | -2.17 |
| mmu-miR-200a-3p | Sox5 | -0.64 | 0.016519799 | 2.61 | -2.90 |
| mmu-miR-411-5p | Galm | -0.64 | 0.016527844 | 4.43 | -2.36 |
| mmu-miR-25-5p | Ddit4 | -0.64 | 0.016539811 | -2.35 | 1.87 |
| mmu-miR-192-5p | Manea | -0.64 | 0.016542302 | 5.95 | -2.60 |
| mmu-miR-375-3p | Slc15a3 | -0.64 | 0.016681563 | -2.94 | 3.44 |
| mmu-miR-130a-3p | Chek2 | -0.64 | 0.016730756 | 2.29 | -1.80 |
| mmu-miR-214-5p | Dusp28 | -0.64 | 0.016738856 | 3.66 | -1.71 |
| mmu-miR-218-5p | Gng11 | -0.64 | 0.016812097 | 3.13 | -2.20 |
| mmu-miR-218-5p | Armc3 | -0.64 | 0.01682604 | 3.13 | -2.25 |
| mmu-miR-193b-3p | Clec2d | -0.64 | 0.016859086 | -2.01 | 1.65 |
| mmu-miR-148a-3p | Dusp28 | -0.64 | 0.016920346 | 2.94 | -1.71 |
| mmu-miR-183-3p | 1700084C01Rik | -0.64 | 0.016927642 | 4.74 | -3.29 |
| mmu-miR-106b-5p | Plscr4 | -0.64 | 0.017041318 | 4.02 | -2.48 |
| mmu-miR-130a-3p | Osbpl6 | -0.64 | 0.017063278 | 2.29 | -1.73 |
| mmu-miR-210-3p | C1s1 | -0.64 | 0.017078204 | 1.86 | -2.40 |
| mmu-miR-16-5p | Axin2 | -0.64 | 0.017187334 | 1.77 | -1.66 |
| mmu-miR-210-3p | Fhit | -0.64 | 0.017190111 | 1.86 | -3.20 |
| mmu-miR-300-3p | Hibch | -0.64 | 0.017305767 | 2.34 | -2.10 |
| mmu-miR-16-5p | Fgfr1 | -0.64 | 0.017318041 | 1.77 | -1.71 |
| mmu-let-7d-3p | Olfm4 | -0.64 | 0.017324488 | -6.28 | 1.70 |
| mmu-miR-214-5p | Ccdc103 | -0.64 | 0.017380093 | 3.66 | -2.01 |
| mmu-miR-205-3p | Ccdc81 | -0.64 | 0.017384777 | 6.09 | -1.59 |
| mmu-miR-29c-5p | Col1a2 | -0.64 | 0.017415202 | 3.44 | -2.33 |
| mmu-miR-181d-5p | Abi3bp | -0.64 | 0.01744727 | 4.07 | -3.56 |
| mmu-miR-30e-5p | Fabp4 | -0.64 | 0.017472818 | 3.60 | -1.95 |
| mmu-miR-92b-5p | Col27a1 | -0.64 | 0.017514101 | -6.76 | 3.31 |
| mmu-miR-135b-5p | Hydin | -0.64 | 0.017514273 | 4.89 | -2.12 |
| mmu-miR-93-5p | Adhfe1 | -0.64 | 0.017522702 | 2.30 | -2.39 |
| mmu-miR-152-3p | 1190002N15Rik | -0.64 | 0.01754137 | 1.92 | -1.84 |
| mmu-miR-181a-5p | Spp1 | -0.64 | 0.017546046 | -3.01 | 3.09 |
| mmu-miR-29c-5p | Rora | -0.64 | 0.017630618 | 3.44 | -2.00 |
| mmu-miR-30a-5p | Ak7 | -0.64 | 0.017784408 | 2.87 | -1.79 |
| mmu-miR-24-3p | Lars2 | -0.64 | 0.017795332 | 1.73 | -2.44 |
| mmu-miR-200a-3p | Osbpl6 | -0.64 | 0.017796873 | 2.61 | -1.73 |
| mmu-miR-194-5p | Acot1 | -0.64 | 0.017799749 | 4.92 | -2.12 |
| mmu-miR-194-5p | Acot1 | -0.64 | 0.017799749 | 4.92 | -2.12 |
| mmu-miR-130a-3p | Nxt2 | -0.64 | 0.017860412 | 2.29 | -2.84 |
| mmu-miR-500-3p | Ppic | -0.63 | 0.017935211 | 3.74 | -2.39 |
| mmu-miR-212-3p | Col4a6 | -0.63 | 0.017948763 | 3.43 | -1.94 |
| mmu-miR-32-5p | Prkar1b | -0.63 | 0.01796756 | 4.39 | -1.97 |
| mmu-miR-210-5p | Nfil3 | -0.63 | 0.0180308 | -3.25 | 1.86 |
| mmu-miR-194-5p | Bmpr1b | -0.63 | 0.018054721 | 4.92 | -2.84 |
| mmu-miR-30e-5p | Ppargc1a | -0.63 | 0.01805884 | 3.60 | -1.61 |
| mmu-miR-16-5p | Chek2 | -0.63 | 0.018064783 | 1.77 | -1.80 |
| mmu-miR-214-5p | Slc23a2 | -0.63 | 0.018085396 | 3.66 | -1.79 |
| mmu-miR-20a-5p | Plscr4 | -0.63 | 0.018140817 | 3.70 | -2.48 |
| mmu-miR-183-3p | Ephx2 | -0.63 | 0.018262086 | 4.74 | -1.99 |
| mmu-miR-326-3p | Psrc1 | -0.63 | 0.018319281 | -1.76 | 3.69 |
| mmu-miR-98-5p | Nkd1 | -0.63 | 0.018490777 | 3.72 | -2.32 |
| mmu-miR-98-5p | Nxt2 | -0.63 | 0.018535139 | 3.72 | -2.84 |
| mmu-miR-194-5p | Aff3 | -0.63 | 0.018614828 | 4.92 | -1.84 |
| mmu-miR-26b-5p | Fsip1 | -0.63 | 0.018633171 | 3.80 | -2.52 |
| mmu-miR-15a-5p | Sox5 | -0.63 | 0.018656071 | 2.29 | -2.90 |
| mmu-miR-192-5p | Osbpl6 | -0.63 | 0.018695857 | 5.95 | -1.73 |
| mmu-miR-27b-3p | Papss2 | -0.63 | 0.018715634 | 2.45 | -1.73 |
| mmu-miR-30b-5p | Rpgr | -0.63 | 0.018731615 | 2.59 | -2.01 |
| mmu-let-7f-5p | Scd1 | -0.63 | 0.018765773 | 3.20 | -1.90 |
| mmu-miR-410-3p | Six4 | -0.63 | 0.0187929 | 3.62 | -1.65 |
| mmu-miR-676-5p | Sox5 | -0.63 | 0.018816449 | 2.82 | -2.90 |
| mmu-miR-223-3p | Osbpl6 | -0.63 | 0.018842992 | 9.15 | -1.73 |
| mmu-miR-26b-5p | Gng11 | -0.63 | 0.01892862 | 3.80 | -2.20 |
| mmu-let-7g-5p | Fkbp1b | -0.63 | 0.018952927 | 2.05 | -2.34 |
| mmu-miR-98-5p | Fkbp1b | -0.63 | 0.01896119 | 3.72 | -2.34 |
| mmu-miR-200b-3p | Fsip1 | -0.63 | 0.018973023 | 2.19 | -2.52 |
| mmu-miR-92b-5p | Havcr2 | -0.63 | 0.019021976 | -6.76 | 1.80 |
| mmu-miR-411-5p | Slc16a10 | -0.63 | 0.019124254 | 4.43 | -1.94 |
| mmu-miR-15a-5p | Spdef | -0.63 | 0.019225086 | 2.29 | -1.94 |
| mmu-let-7f-5p | Fkbp1b | -0.63 | 0.019246513 | 3.20 | -2.34 |
| mmu-miR-210-3p | Cdh26 | -0.63 | 0.0193417 | 1.86 | -2.39 |
| mmu-let-7f-5p | Ppargc1a | -0.63 | 0.019360208 | 3.20 | -1.61 |
| mmu-let-7g-5p | Nxt2 | -0.63 | 0.019394809 | 2.05 | -2.84 |
| mmu-miR-27a-3p | Papss2 | -0.63 | 0.019397925 | 3.17 | -1.73 |
| mmu-let-7f-5p | Nkd1 | -0.63 | 0.019471776 | 3.20 | -2.32 |
| mmu-let-7f-5p | Nxt2 | -0.63 | 0.019480044 | 3.20 | -2.84 |
| mmu-miR-27b-3p | Tpsb2 | -0.63 | 0.019480452 | 2.45 | -2.56 |
| mmu-miR-32-5p | Aff3 | -0.63 | 0.019717918 | 4.39 | -1.84 |
| mmu-miR-340-5p | Chek2 | -0.63 | 0.019737087 | 9.01 | -1.80 |
| mmu-miR-212-3p | Smoc2 | -0.63 | 0.01974756 | 3.43 | -2.15 |
| mmu-miR-22-3p | Chil3 | -0.63 | 0.019787672 | 2.95 | -2.14 |
| mmu-miR-22-3p | Nxt2 | -0.63 | 0.019811234 | 2.95 | -2.84 |
| mmu-miR-212-3p | Slc27a2 | -0.63 | 0.019864127 | 3.43 | -3.23 |
| mmu-miR-340-5p | Actc1 | -0.62 | 0.019939361 | 9.01 | -2.17 |
| mmu-miR-345-3p | Rora | -0.62 | 0.020100068 | 2.14 | -2.00 |
| mmu-miR-16-5p | Ccdc81 | -0.62 | 0.020129125 | 1.77 | -1.59 |
| mmu-miR-135b-5p | Armc4 | -0.62 | 0.020177788 | 4.89 | -3.02 |
| mmu-miR-214-3p | Ptgs2 | -0.62 | 0.020203538 | -1.90 | 3.28 |
| mmu-miR-128-3p | Nxt2 | -0.62 | 0.020212936 | 2.80 | -2.84 |
| mmu-miR-92b-3p | Ptger4 | -0.62 | 0.020248635 | -4.78 | 2.20 |
| mmu-miR-20a-5p | Zfp791 | -0.62 | 0.020274007 | 3.70 | -2.85 |
| mmu-miR-181d-5p | Mlf1 | -0.62 | 0.020277424 | 4.07 | -2.32 |
| mmu-miR-185-5p | St8sia2 | -0.62 | 0.020348507 | 2.50 | -1.66 |
| mmu-miR-425-5p | Nxt2 | -0.62 | 0.020398282 | 1.93 | -2.84 |
| mmu-miR-205-3p | Fbxo16 | -0.62 | 0.020426564 | 6.09 | -2.41 |
| mmu-let-7i-5p | Islr | -0.62 | 0.020448817 | 1.79 | -2.28 |
| mmu-miR-203-3p | Fkbp1b | -0.62 | 0.020633831 | 4.73 | -2.34 |
| mmu-miR-15a-5p | Axin2 | -0.62 | 0.020727244 | 2.29 | -1.66 |
| mmu-let-7b-5p | Plb1 | -0.62 | 0.020798701 | -3.08 | 2.01 |
| mmu-miR-23b-3p | Fbn1 | -0.62 | 0.020828969 | 2.62 | -3.25 |
| mmu-miR-132-3p | Sox5 | -0.62 | 0.020852084 | 2.20 | -2.90 |
| mmu-miR-15a-5p | Apoo | -0.62 | 0.02089595 | 2.29 | -3.38 |
| mmu-miR-185-5p | 1700084C01Rik | -0.62 | 0.021000851 | 2.50 | -3.29 |
| mmu-miR-99b-5p | Capn13 | -0.62 | 0.02106072 | 1.97 | -3.68 |
| mmu-miR-24-3p | Pde2a | -0.62 | 0.021071248 | 1.73 | -1.61 |
| mmu-miR-214-5p | Ccdc81 | -0.62 | 0.021114057 | 3.66 | -1.59 |
| mmu-miR-15a-5p | Ccdc81 | -0.62 | 0.021116563 | 2.29 | -1.59 |
| mmu-miR-340-5p | Aldh1a2 | -0.62 | 0.021129132 | 9.01 | -3.23 |
| mmu-let-7g-5p | Scd1 | -0.62 | 0.021214633 | 2.05 | -1.90 |
| mmu-miR-744-5p | Phlda2 | -0.62 | 0.021223832 | -3.34 | 2.34 |
| mmu-miR-25-5p | Slc7a11 | -0.62 | 0.021226871 | -2.35 | 1.80 |
| mmu-miR-29c-3p | Dnah7b | -0.62 | 0.021267001 | 5.21 | -1.97 |
| mmu-let-7i-5p | Nkd1 | -0.62 | 0.021294434 | 1.79 | -2.32 |
| mmu-miR-135b-5p | Ctxn1 | -0.62 | 0.02129547 | 4.89 | -1.66 |
| mmu-miR-23b-3p | Zfp791 | -0.62 | 0.021412631 | 2.62 | -2.85 |
| mmu-miR-214-5p | Sez6l2 | -0.62 | 0.021487242 | 3.66 | -3.64 |
